# Lipid-lowering effect of combined therapy with high-intensity statins and CETP inhibitors: a Systematic Review and meta-analysis

**DOI:** 10.3389/fendo.2025.1512670

**Published:** 2025-05-01

**Authors:** Liubo Xiang, Huan Wu, Zhihao Zhao, Tingchun Wu, Dawei Lv, Ping Wu, Yuhua Zheng, Qianqian Huang, Tao Xu

**Affiliations:** ^1^ Second Clinical Medical College, Guizhou University of Traditional Chinese Medicine, Gui Yang, China; ^2^ Cardiovascular Medicine, The Second Affiliated Hospital of Guizhou University of Traditional Chinese Medicine, Gui Yang, China

**Keywords:** high-intensity, statins, CETP inhibitors, combination therapy, lipid-lowering

## Abstract

**Background:**

This study aimed to evaluate the impact of combining high-intensity statins with CETP inhibitors on lipid levels, as well as to explore their potential clinical significance.

**Methods:**

We conducted a comprehensive search of relevant studies in the PubMed, Embase, Cochrane Library, and Web of Science databases. The Cochrane Risk of Bias Tool RoB 2.0 was employed to evaluate the quality of the included studies. Statistical analyses were carried out using STATA 15 software, with primary outcomes being high-density lipoprotein cholesterol (HDL-C) and low-density lipoprotein cholesterol (LDL-C).

**Results:**

Out of 2,552 records, 7 studies were included in the final analysis. The findings revealed that the combination of high-intensity statins with CETP inhibitors significantly raised HDL-C levels (SMD 2.47 [1.77, 3.18], p < 0.001) and lowered LDL-C levels (SMD -1.75 [-2.19, -1.31], p < 0.001).

**Conclusion:**

Compared to statin monotherapy, the combination of high-intensity statins and CETP inhibitors resulted in a more pronounced increase in HDL-C and ApoAI, while reducing LDL-C, triglycerides (TG), and ApoB levels, without increasing the incidence of adverse events.

## 1 Introduction

Controlling lipid levels is one of the key strategies in the prevention and management of cardiovascular diseases, as it reduces the risk of these conditions (1). Hyperlipidemia is a prevalent metabolic disorder characterized by elevated levels of lipids in the blood, resulting from a combination of genetic predisposition and environmental factors such as unhealthy diet and sedentary lifestyle (2). It is a significant contributor to the global burden of cardiovascular diseases, with studies estimating that over 50% of adults in the United States exhibit elevated low-density lipoprotein (LDL) levels (3). This condition is strongly associated with the development of atherosclerotic cardiovascular disease (ASCVD), significantly increasing the risk of ASCVD (4). Beyond ASCVD, hyperlipidemia also contributes to other complications such as pancreatitis, non-alcoholic fatty liver disease, and metabolic syndrome, further underscoring the need for effective management (5–8).

Currently, the treatment of hyperlipidemia primarily relies on statins, especially high-intensity statins (such as atorvastatin and rosuvastatin), which are the first-line treatment due to their excellent effect in lowering LDL cholesterol (LDL-C) and reducing cardiovascular events (9). However, despite the effectiveness of high-intensity statins in most patients, some individuals fail to achieve optimal LDL-C levels, a phenomenon referred to as the “residual risk after statin therapy” (10). Additionally, some patients cannot tolerate high-dose statins due to statin intolerance or side effects. These clinical challenges have prompted researchers and clinicians to explore additional lipid-lowering agents that can be used in combination with statins to further optimize lipid control.

Cholesteryl ester transfer protein (CETP) inhibitors are a new class of lipid-lowering drugs. By inhibiting CETP activity, they prevent the transfer of cholesterol from high-density lipoprotein cholesterol (HDL-C) to other lipoproteins, thereby raising HDL-C levels and lowering LDL-C levels (11, 12). This mechanism offers a potential adjunctive treatment option for hyperlipidemic patients, particularly those who are unresponsive to or intolerant of statin therapy. However, despite the favorable lipid-lowering effects observed in early studies, CETP inhibitors may increase mortality and cardiovascular disease risks (13). Based on current research, there is growing evidence supporting combination lipid-lowering strategies to optimally reduce lipid levels (14) he combination of high-intensity statins and CETP inhibitors has garnered widespread attention and is considered a potential comprehensive lipid management option for hyperlipidemic patients. Nevertheless, a systematic review of the combined effects of high-intensity statins and CETP inhibitors on lipid metabolism is still lacking. Therefore, this study aims to synthesize the available data through a systematic review and meta-analysis to evaluate the effects of combined therapy with high-intensity statins and CETP inhibitors on lipid levels in patients. By performing a comprehensive analysis of these study results, it aims to provide more reliable evidence to guide clinical practice and help physicians make more informed decisions when developing individualized treatment plans for hyperlipidemic patients.

## 2 Methods

This study follows the guidelines of the 2015 Preferred Reporting Items for Systematic Review and Meta-Analysis Protocols (PRISMA-P) statement (15). The protocol for our review was registered in PROSPERO under the registration number CRD42024581470. Ethical approval and patient consent were not required for this study.

### 2.1 Literature search and selection

Two independent researchers performed a systematic search of the PubMed, Cochrane Library, Embase, and Web of Science databases, covering studies from the databases’ inception until May 9, 2024. The search strategies were adapted for each database, using MeSH terms “Hydroxymethylglutaryl-CoA Reductase Inhibitors” and “Cholesterol Ester Transfer Proteins” along with relevant free-text terms. Boolean operators “OR” and “AND” were applied to build the search queries (details of the search strategy can be found in the appendix). In addition, we reviewed the reference lists of the retrieved studies to identify other relevant articles.

Two researchers (XLB and WH) independently reviewed the titles and abstracts of the initially identified studies. Studies were included if they met the following criteria (1): randomized controlled trials or crossover trials involving human participants; (2) published in English; (3) the intervention group received combination therapy with high-intensity statins and cholesteryl ester transfer protein (CETP) inhibitors, while the control group received either placebo or monotherapy; (4) studies included at least one of the following outcomes: high-density lipoprotein cholesterol (HDL-C), low-density lipoprotein cholesterol (LDL-C), total cholesterol (TC), triglycerides (TG), apolipoprotein AI (ApoAI), apolipoprotein B (ApoB), and adverse events.

Studies were excluded if they met any of the following criteria: (1) the type or dose of the drugs used was not clearly specified; (2) complete outcome data were not available; (3) they were letters, editorials, case reports, conference abstracts, meta-analyses, reviews, or patents.

According to the American College of Cardiology (ACC) and American Heart Association (AHA) guidelines (16), high-intensity statins were defined as daily doses of 20 or 40 mg of rosuvastatin or 40–80 mg of atorvastatin.

### 2.2 Quality assessment

The methodological quality of all included studies was independently evaluated by two researchers using the Cochrane Risk of Bias tool RoB 2.0. The assessment focused on five key domains: bias arising from the randomization process, bias due to deviations from intended interventions, bias due to missing outcome data, bias in outcome measurement, and bias in selective outcome reporting.

### 2.3 Data extraction

Data extraction was performed independently by two researchers (XLB and WH). Discrepancies were resolved through discussion with a third researcher until consensus was reached. The following information was extracted from the full texts of the included studies: basic information (first author, year of publication, country of study, study design), study cohort information (sample size, age, treatment drugs, dosage, follow-up duration), and outcome measures.

The primary outcome measures included: (1) HDL-C; (2) LDL-C. Secondary outcome measures were: (1) TC; (2) TG; (3) ApoAI; (4) ApoB; (5) adverse events.

### 2.4 Statistical analysis

Statistical analysis was performed using STATA 16.0. In studies with more than one intervention group, the control group was split between the two intervention groups to avoid double-counting in the meta-analysis. For continuous variables, normally distributed data were presented as mean ± standard deviation, while non-normally distributed data were presented as median (interquartile range). We calculated the standardized mean difference (SMD) and 95% confidence intervals (CIs) for HDL-C, LDL-C, TC, TG, ApoAI, and ApoB. For dichotomous variables, we calculated the risk ratio (RR) and 95% CIs for adverse events.

To assess heterogeneity between studies, we used the chi-squared test and the I² statistic. I² values of 0%, 25%, 50%, and 75% were interpreted as no heterogeneity, low heterogeneity, moderate heterogeneity, and high heterogeneity, respectively. A fixed-effects model was used if I² was <50% and clinical and methodological heterogeneity among studies was minimal; otherwise, a random-effects model was applied. Furthermore, to investigate the sources of heterogeneity and ascertain the relationship between the types of CETP inhibitors and lipid-lowering effects, we conducted a subgroup analysis based on the categories of CETP inhibitors. All meta-analysis results were displayed in forest plots.

Publication bias was assessed using Egger’s test and a visual inspection of funnel plots. Additionally, we performed sensitivity analysis using a “leave-one-out” method to assess the robustness of the results by comparing meta-analyses including all studies with those after removing one study at a time.

## 3 Results

### 3.1 Search results and study selection

**Figure 1:**
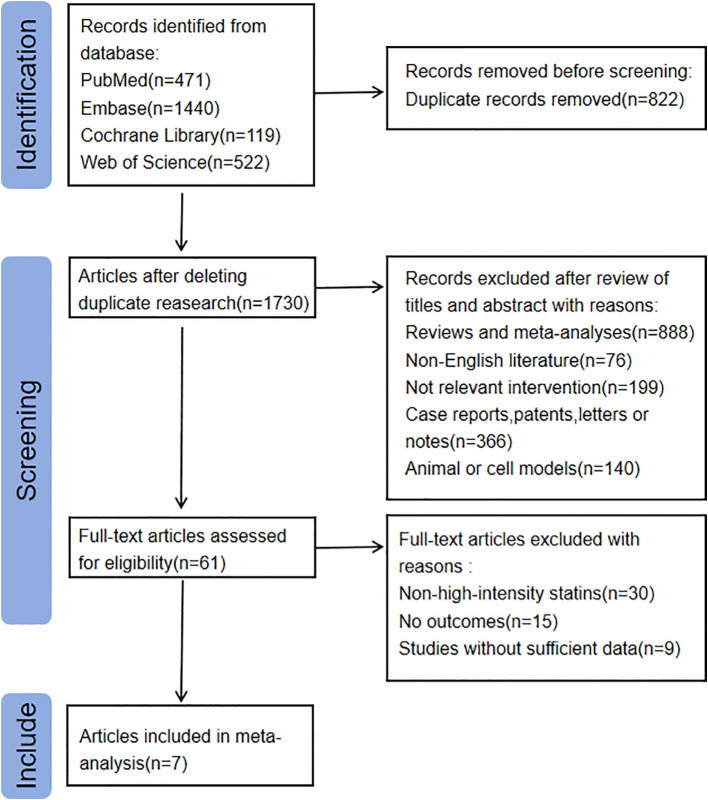
Literature search and screening flowchart.

The flowchart for the literature search and screening process is shown in **Figure 1**. A total of 2,552 relevant records were identified from the following databases: PubMed (n = 471), Embase (n = 1,440), Cochrane Library (n = 119), and Web of Science (n = 522). After removing duplicate articles, 1,730 articles were screened based on their titles and abstracts. We limited the language of the articles to English, resulting in the exclusion of 76 articles. Additionally, 1,254 articles were excluded due to inappropriate article types. Another 140 articles were excluded because their studies were conducted on animals or cells. Moreover, 199 articles were excluded as their interventions were not relevant to this study.

Subsequently, we conducted a full-text review and screening of 61 articles, excluding 54 of them. Ultimately, 7 articles were included in the study (17–23). Among the excluded articles, 30 did not meet the definition of high-intensity statin interventions, and 24 either lacked relevant outcomes or had incomplete data. The main characteristics of the included studies are presented in **Supplementary Material** (**Supplementary Table 2**).

### 3.2 Risk of bias

**Figure 2:**
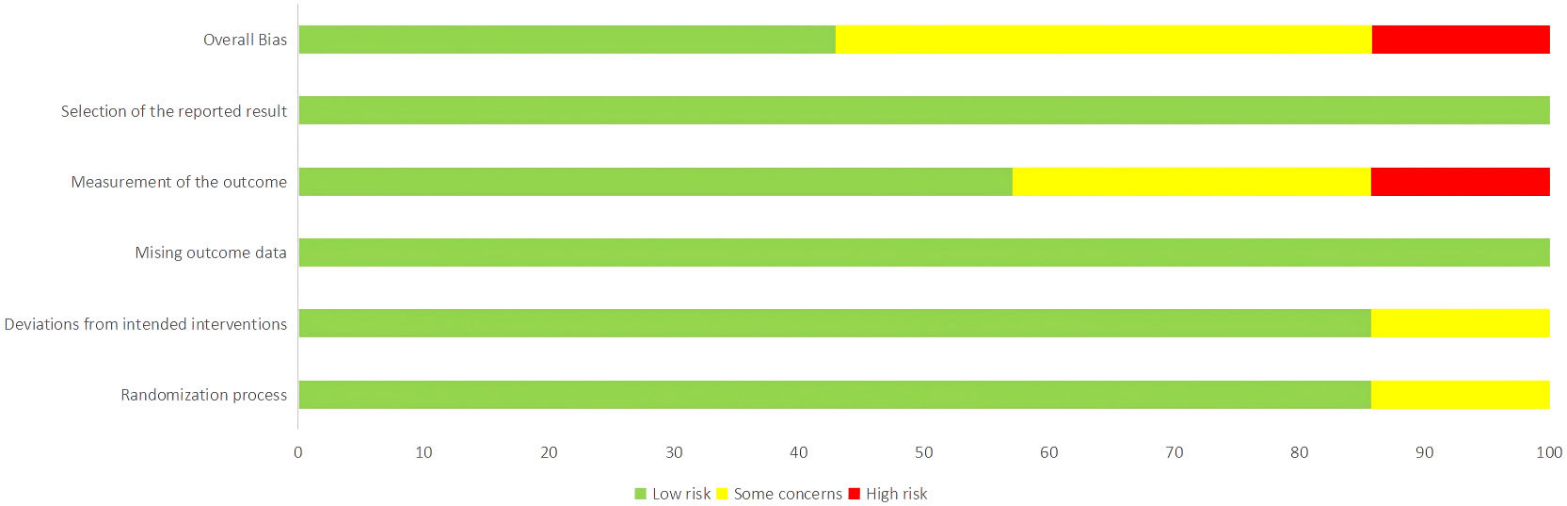
Proportion of risk of bias.

**Figure 3:**
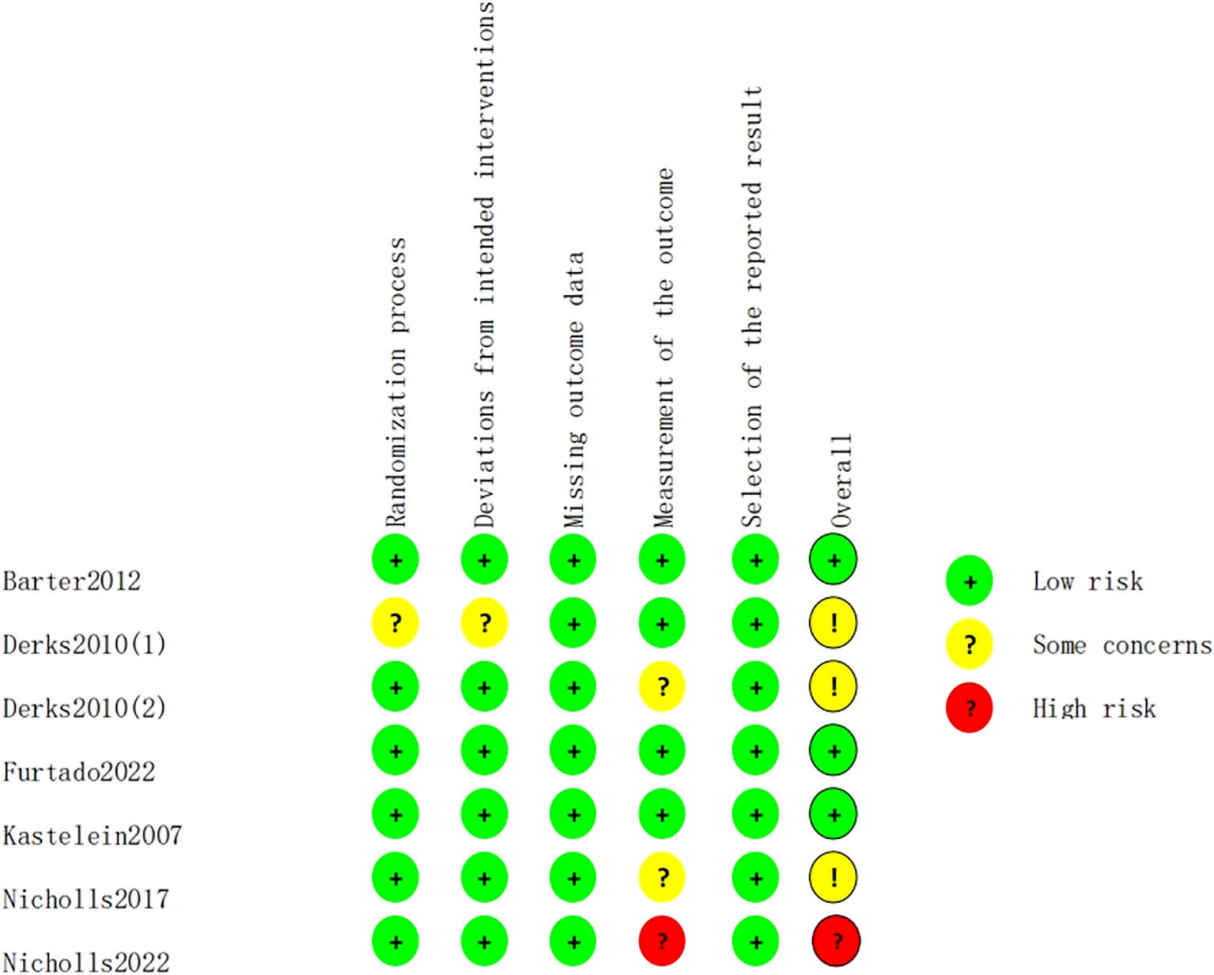
Overall methodological quality assessment.

The risk of bias for the 7 included studies was assessed using the Cochrane risk of bias tool, RoB 2.0. Among the included studies, there were 5 randomized controlled trials and 2 crossover trials. The proportion of risk of bias and the overall methodological quality assessment are shown in **Figures 2** and **3**. Of these, 3 studies were of high quality, with all five domains assessed by the risk assessment tool rated as low risk. However, the study by Nicholls et al. (22) did not provide a description of the outcome measurement methods, leading to a high rivsk of bias for outcome measurement. Overall, the quality of the included studies was high.

### 3.3 Meta-analysis results

#### 3.3.1 HDL-C

**Figure 4:**
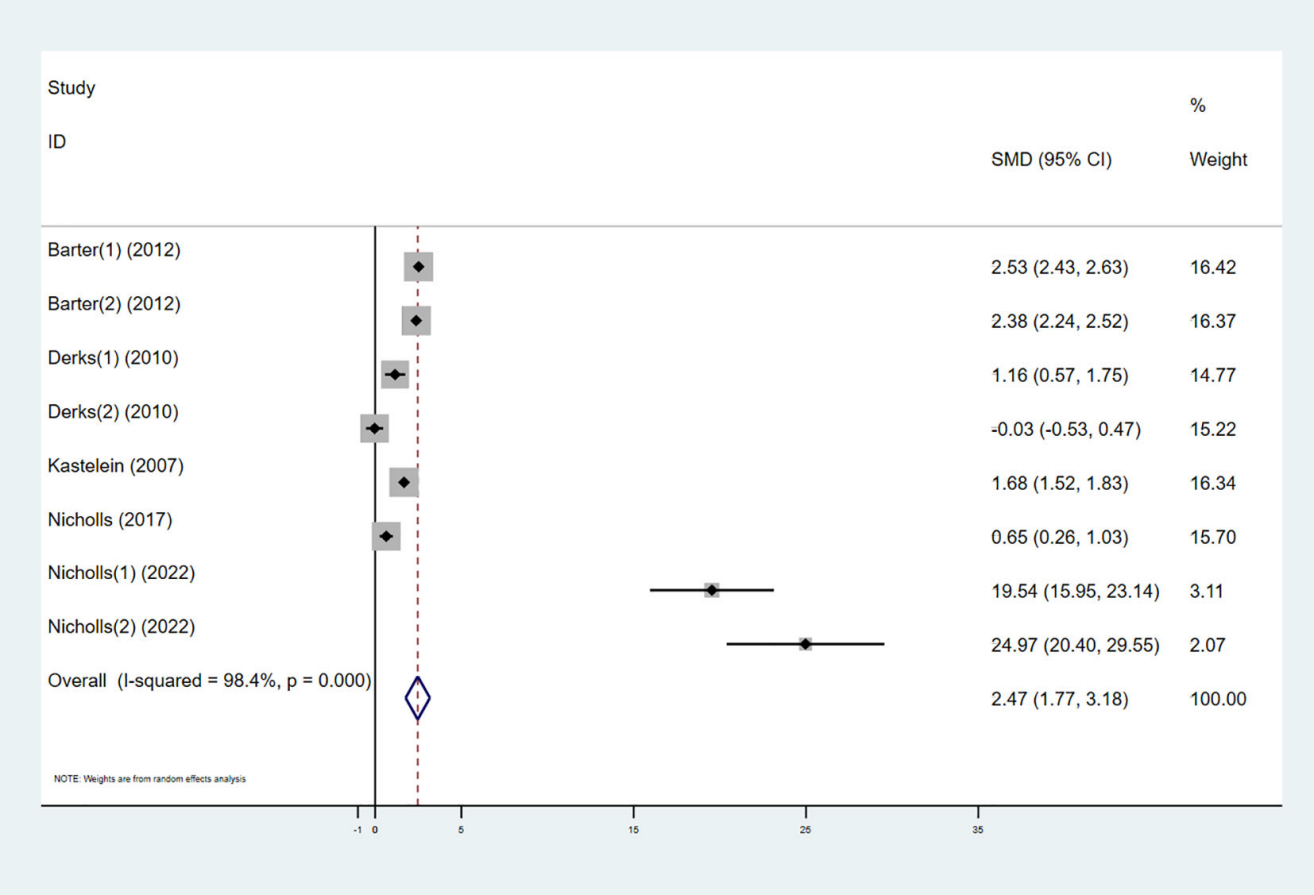
Forest plot for HDL-C.

Six studies investigated the effect of combining high-intensity statins with CETP inhibitors on HDL-C, including 5,575 participants (2,838 in the experimental group and 2,737 in the control group). The results showed that the combination therapy significantly increased HDL-C levels (SMD 2.47 [1.77, 3.18], I² = 98.4%, p < 0.001) (**Figure 4**).

#### 3.3.2 LDL-C

**Figure 5:**
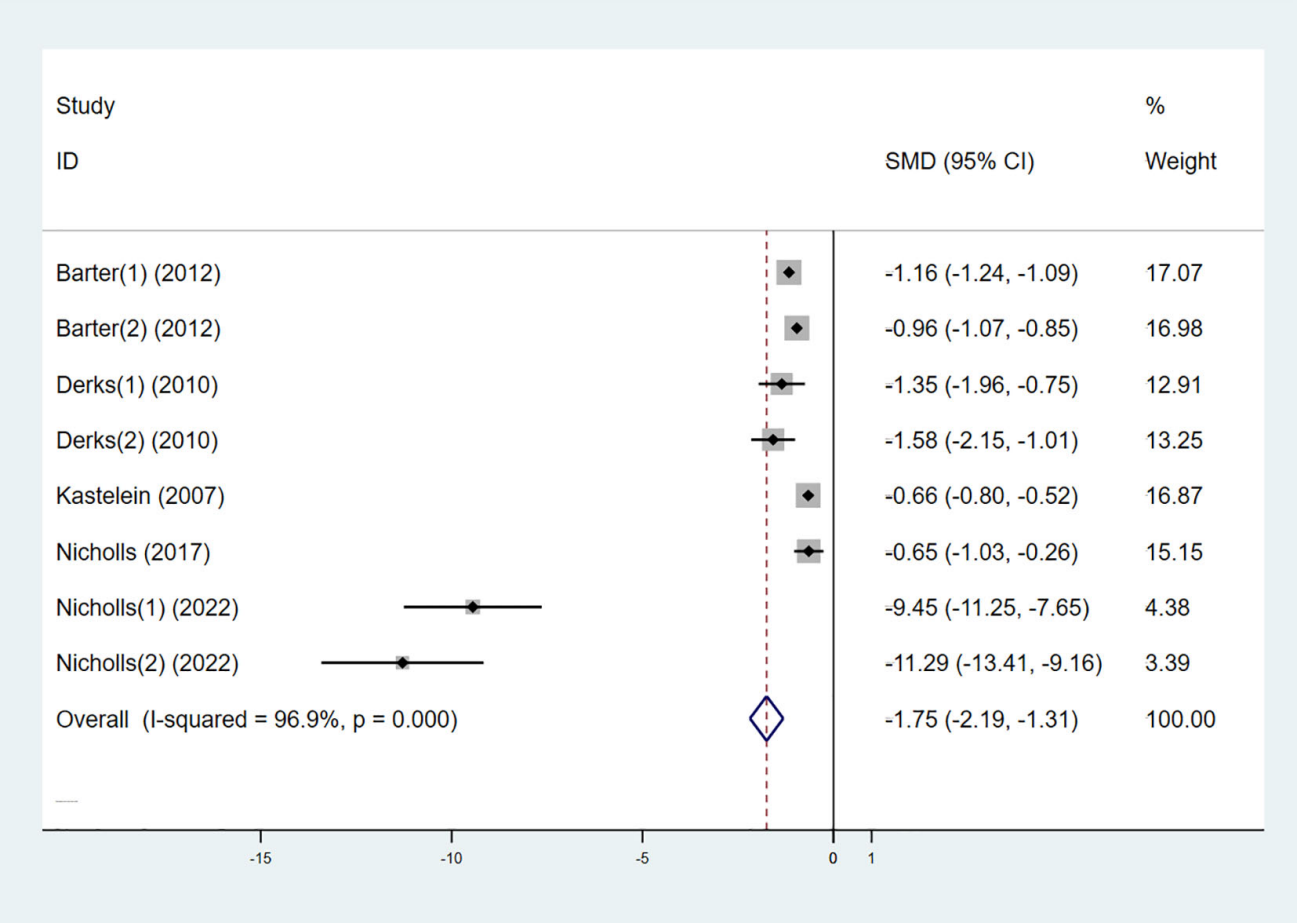
Forest plot for LDL-C.

Six studies also examined the effect of high-intensity statins combined with CETP inhibitors on LDL-C, with 5,556 participants (2,829 in the experimental group and 2,727 in the control group). The results indicated that LDL-C levels were significantly reduced in the experimental group compared to the control group (SMD -1.75 [-2.19, -1.31], I² = 96.9%, p < 0.001) (**Figure 5**).

#### 3.3.3 Total cholesterol

**Figure 6:**
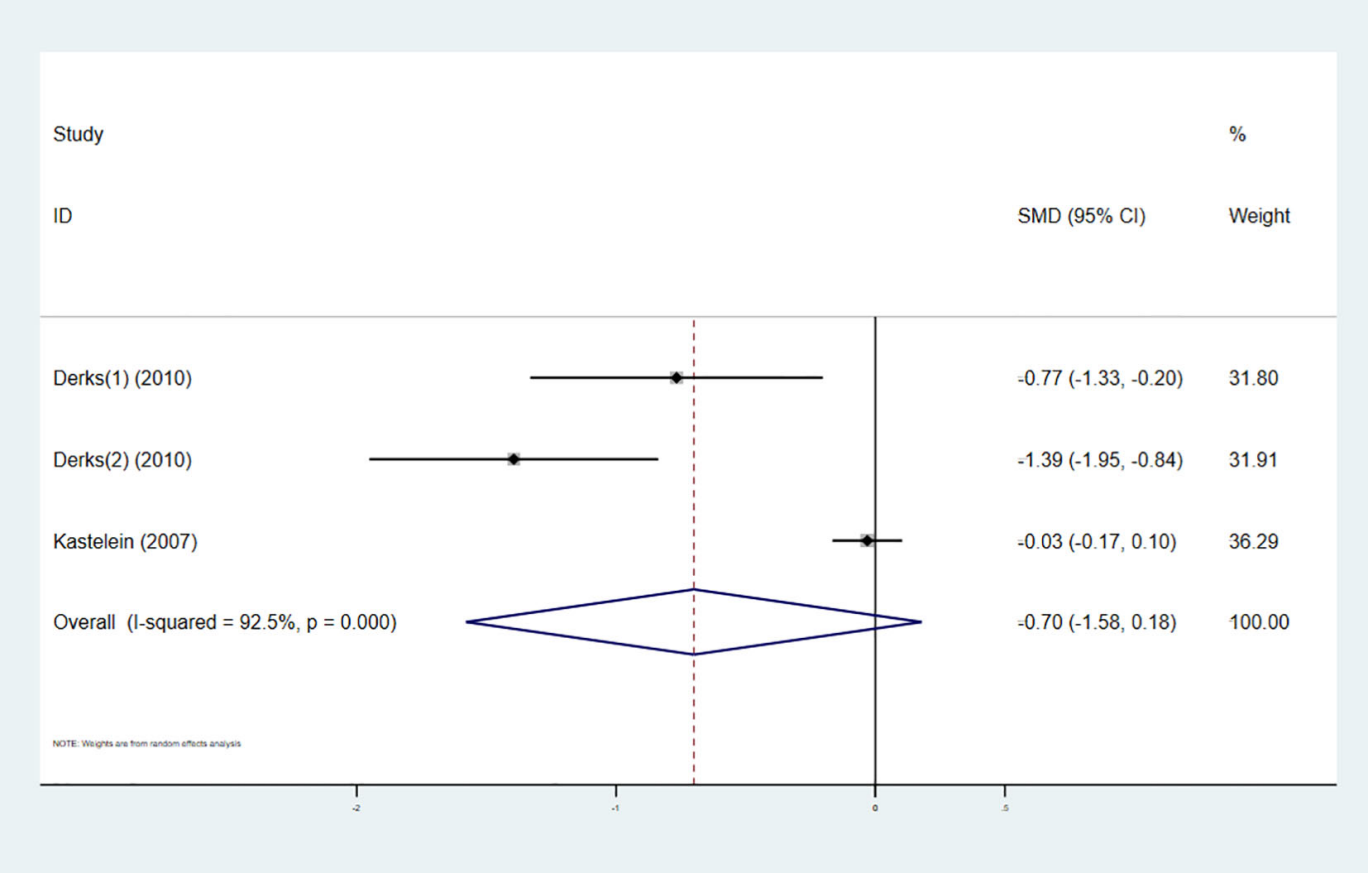
Forest plot for TC.

Three studies investigated the effect of combining high-intensity statins with CETP inhibitors on TC, involving 964 participants (480 in the experimental group and 484 in the control group). The pooled analysis showed no statistically significant difference in TC changes between the experimental and control groups (SMD -0.70 [-1.58, 0.18], I² = 92.5%, p < 0.001) (**Figure 6**).

#### 3.3.4 Triglycerides

**Figure 7:**
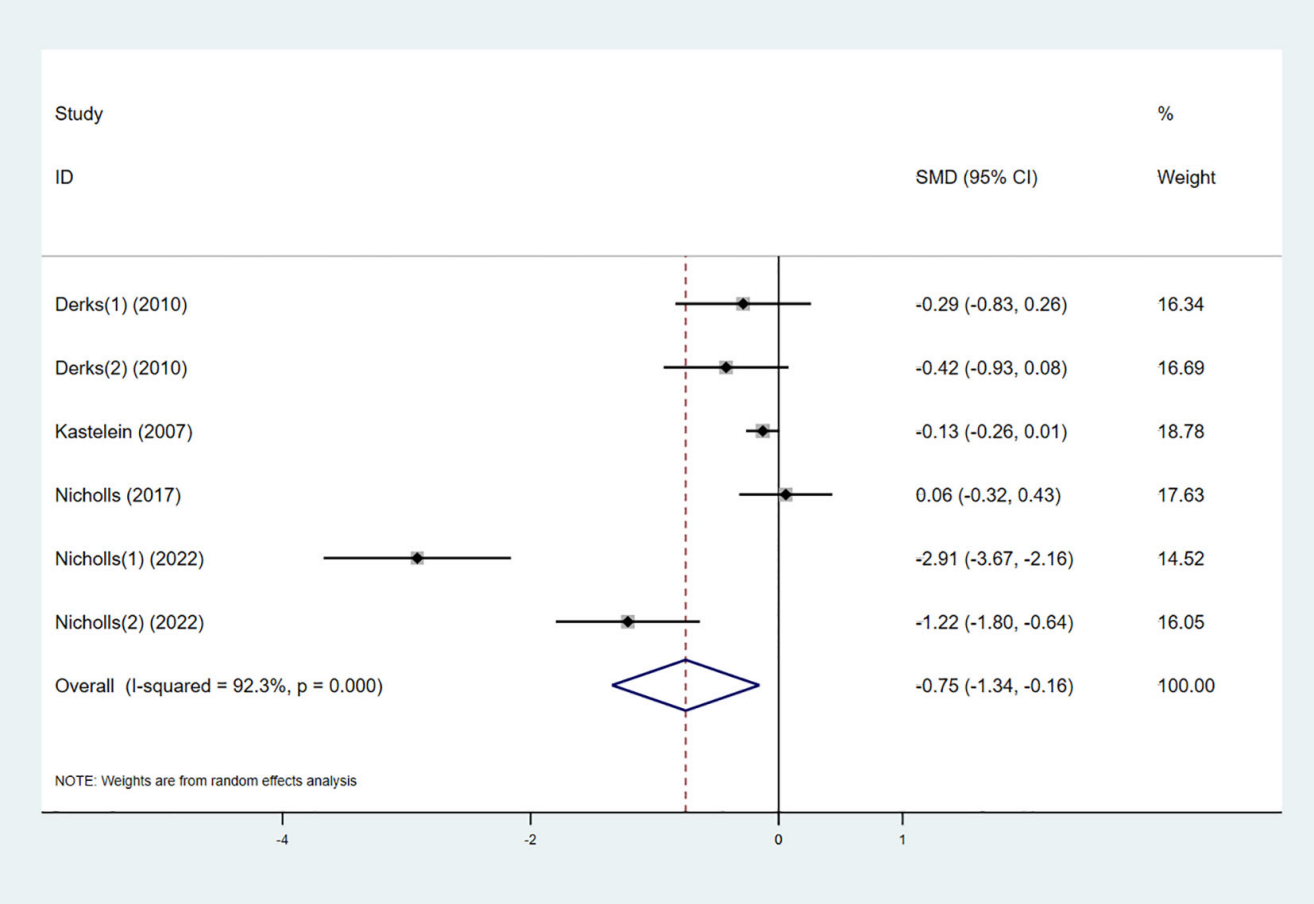
Forest plot for TG.

Five studies examined the effect of the combination therapy on TG. The results indicated that TG levels significantly decreased in the combination therapy group compared to the control group (SMD -0.75 [-1.34, -0.16], I² = 92.3%, p < 0.001) (**Figure 7**).

#### 3.3.5 ApoAI

**Figure 8:**
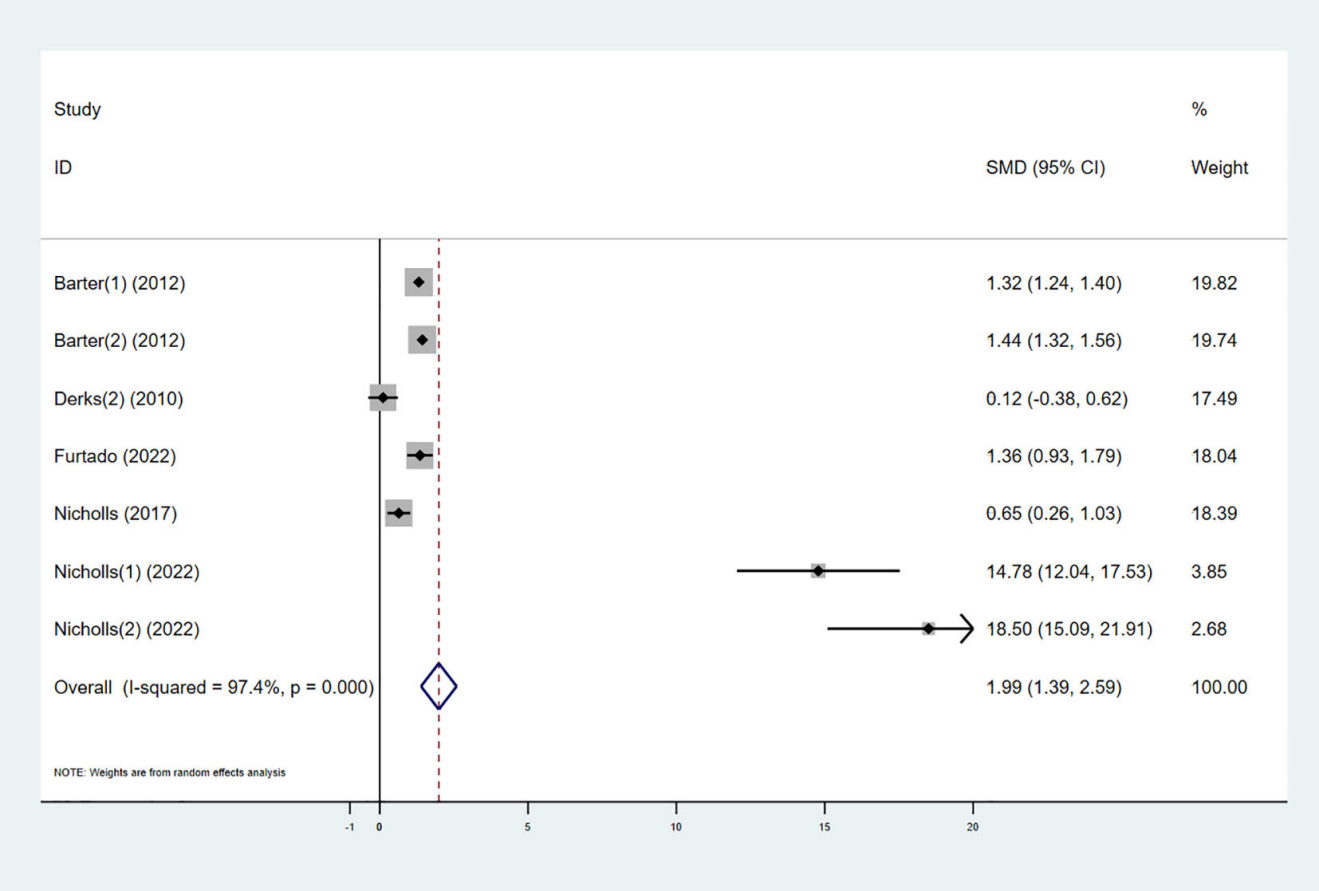
Forest plot for ApoAI.

Five studies explored the effect of combining high-intensity statins with CETP inhibitors on ApoAI levels. The results showed that ApoAI levels in the experimental group significantly increased (SMD 1.99 [1.39, 2.59], I² = 92.3%, p < 0.001) (**Figure 8**).

#### 3.3.6 ApoB

**Figure 9:**
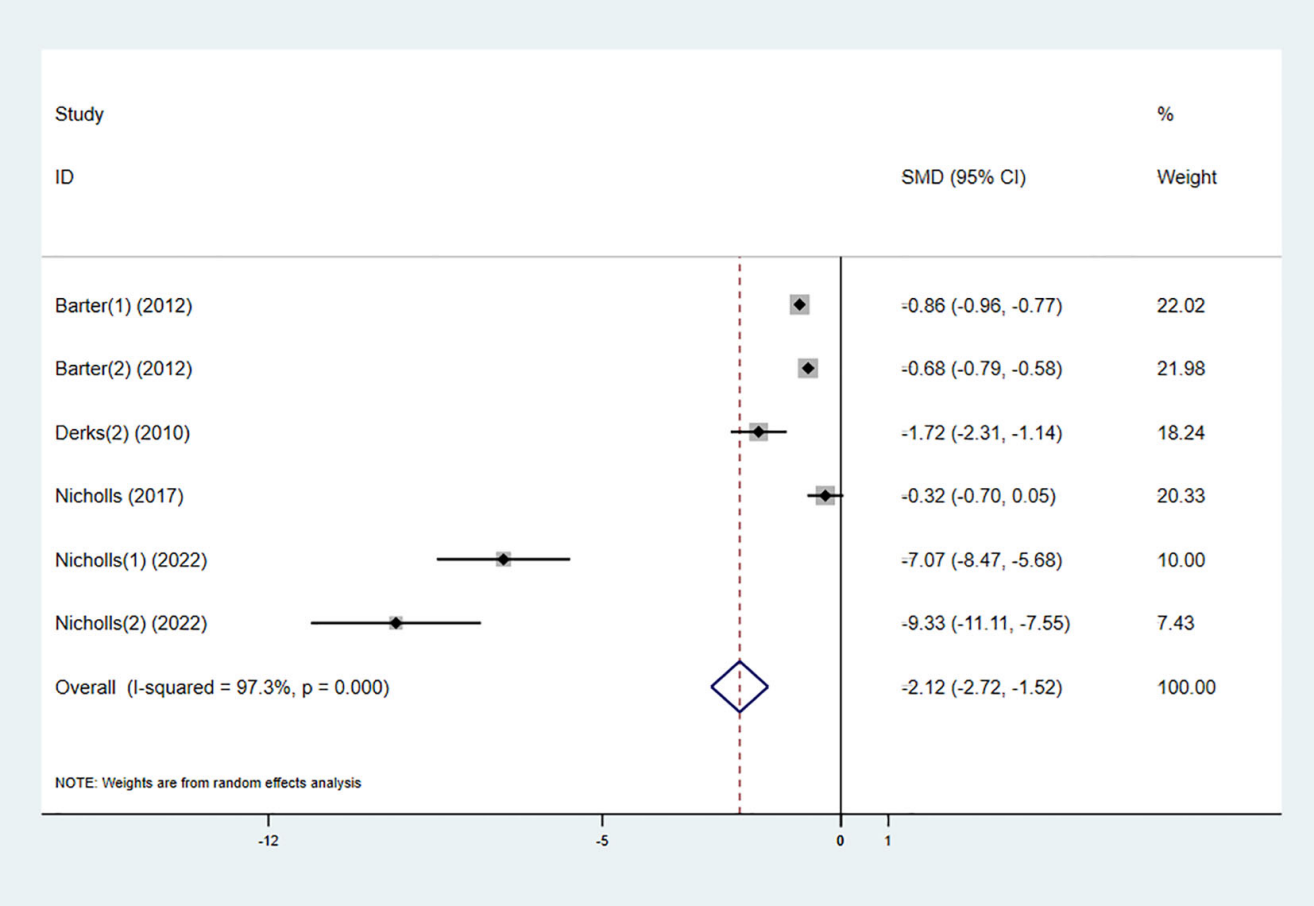
Forest plot for ApoB.

Four studies investigated the effect of the combination therapy on ApoB levels, with the results indicating a significant reduction in ApoB levels in the experimental group (SMD -2.12 [-2.72, -1.52], I² = 97.3%, p < 0.001) (**Figure 9**).

#### 3.3.7 Adverse reactions

**Figure 10:**
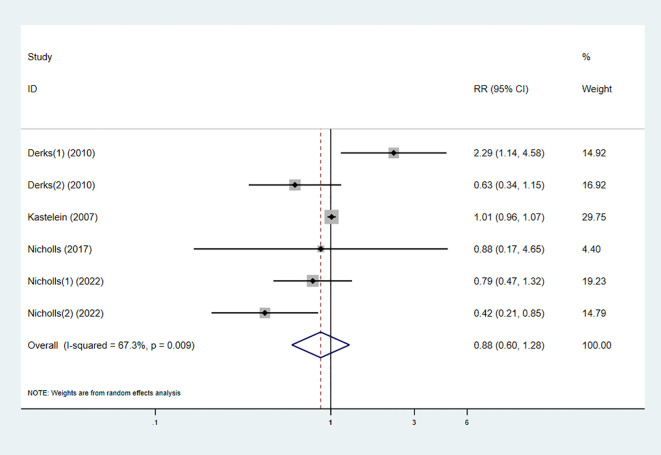
Forest plot for AEs.

Five studies reported the occurrence of adverse reactions in both the experimental and control groups. The pooled results showed no statistically significant difference in the risk of adverse reactions between the combination therapy and statin monotherapy (RR 0.88 [0.60, 1.28], I² = 67.3%, p = 0.009) (**Figure 10**).

### 3.4 Subgroup analysis

To determine the relationship between different types of CETP inhibitors and lipid-lowering effects, a subgroup analysis was conducted on the included studies. Based on the type of CETP inhibitor used in the combination therapy, the studies were categorized into the “torcetrapib” group, “dalcetrapib” group, “evacetrapib” group, and “obicetrapib” group.

**Figure 11:**
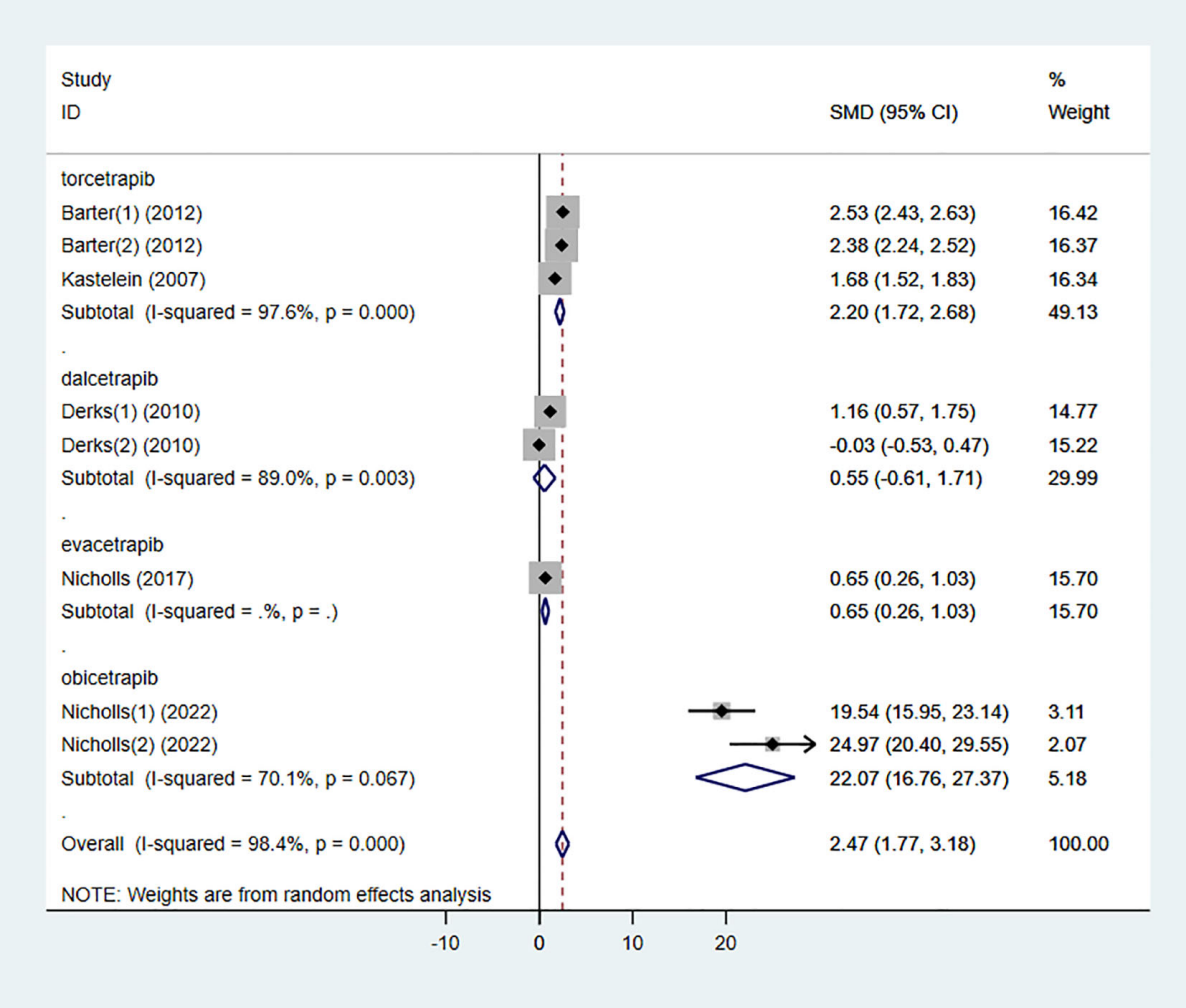
Subgroup analysis for HDL-C.

The results showed that the “dalcetrapib” group did not demonstrate a significant difference in HDL-C elevation compared to the control group (SMD 0.55 [-0.61, 1.71], I² = 89.0%, p = 0.003) (**Figure 11**). However, other types of CETP inhibitors combined with high-intensity statins significantly increased HDL-C levels in the participants.

**Figure 12:**
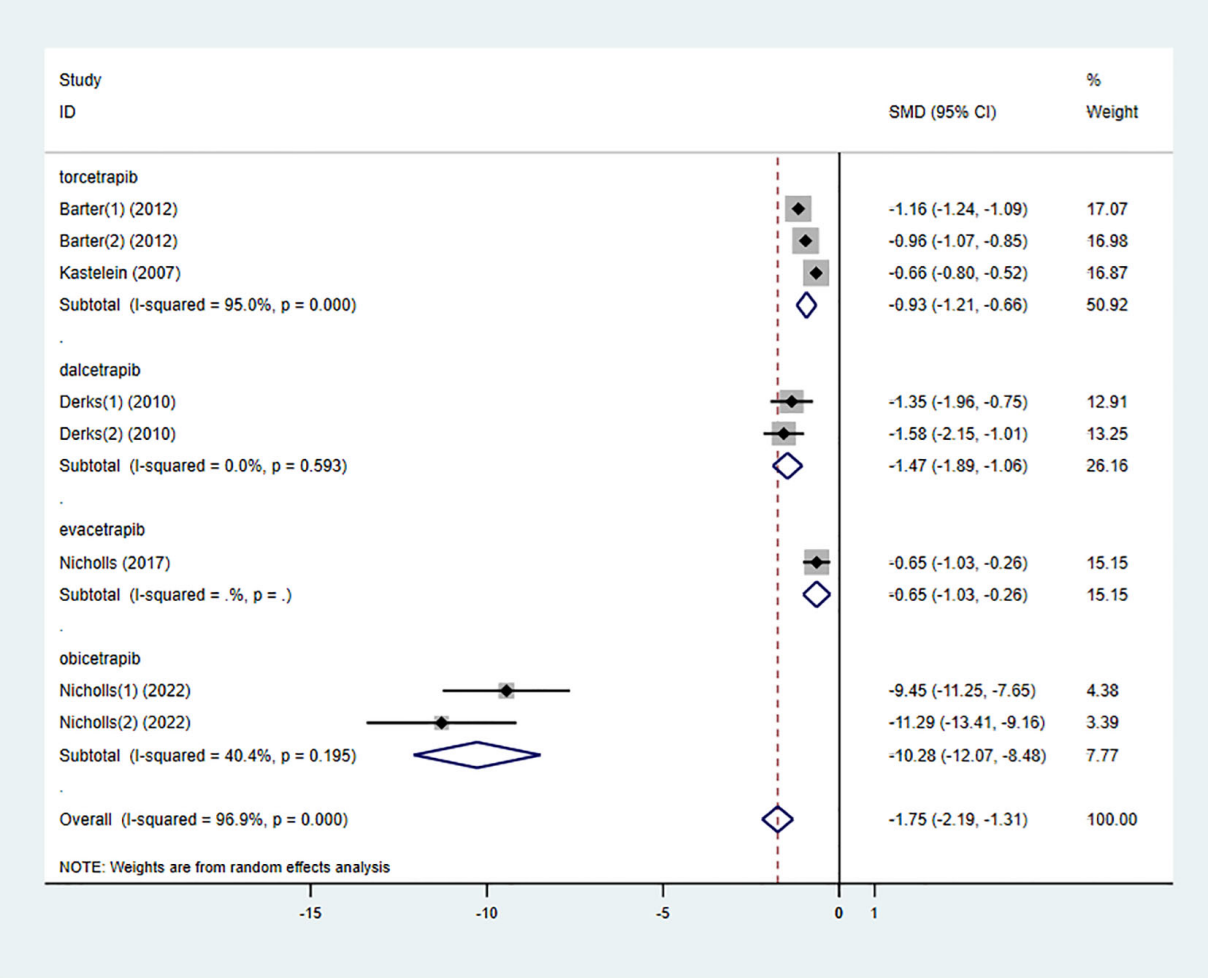
Subgroup analysis for LDL-C.

On the other hand, all types of CETP inhibitors combined with high-intensity statins significantly reduced LDL-C levels in participants. Among them, “obicetrapib” had the most pronounced effect in lowering LDL-C levels (SMD -10.28 [-12.07, -8.48], I² = 40.4%, p = 0.195) (**Figure 12**), while there was no significant difference between the “torcetrapib,” “dalcetrapib,” and “evacetrapib” groups.

### 3.5 Publication bias and sensitivity analysis

**Figure 13:**
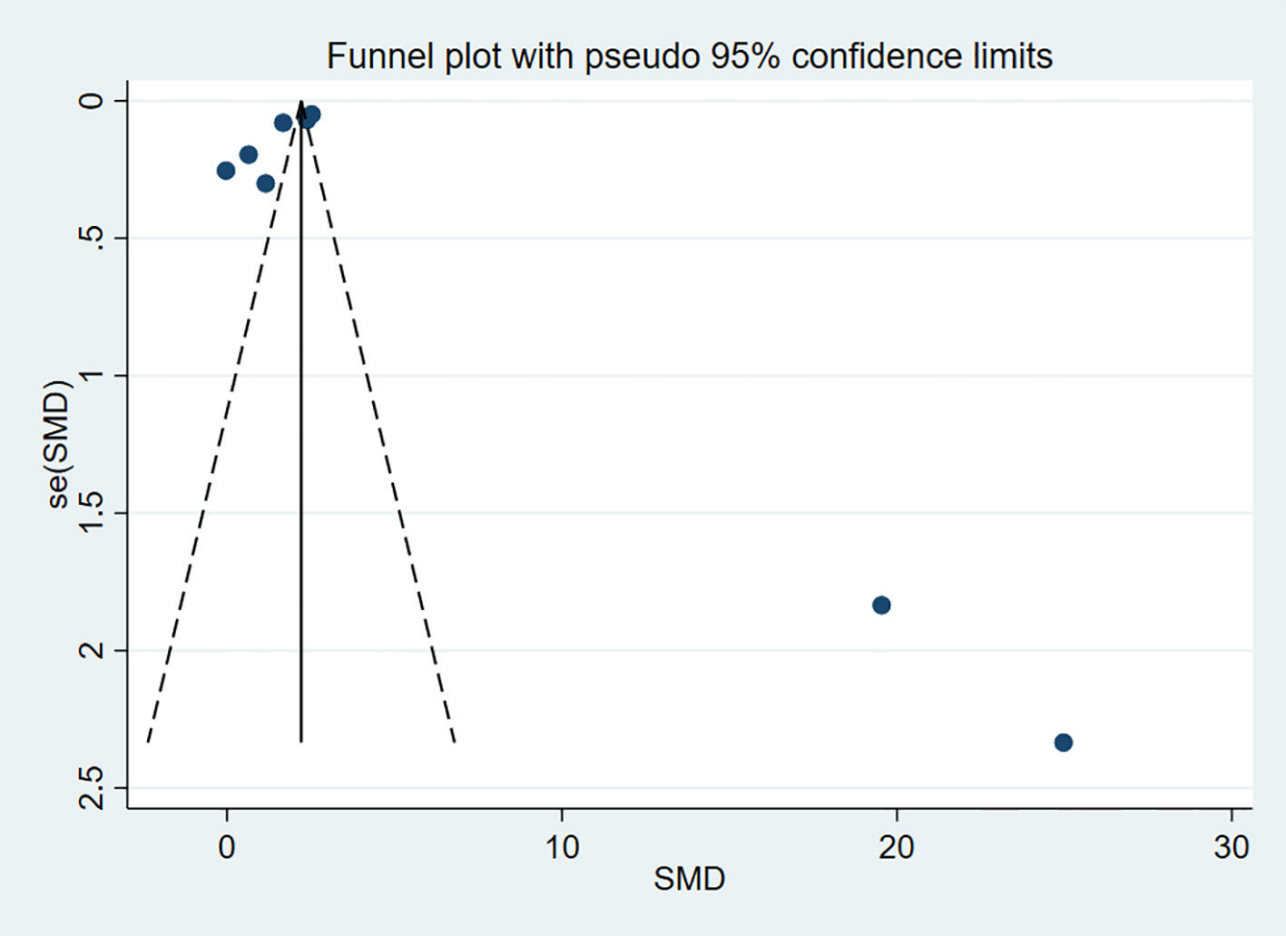
Funnel plot for HDL-C.

**Figure 14:**
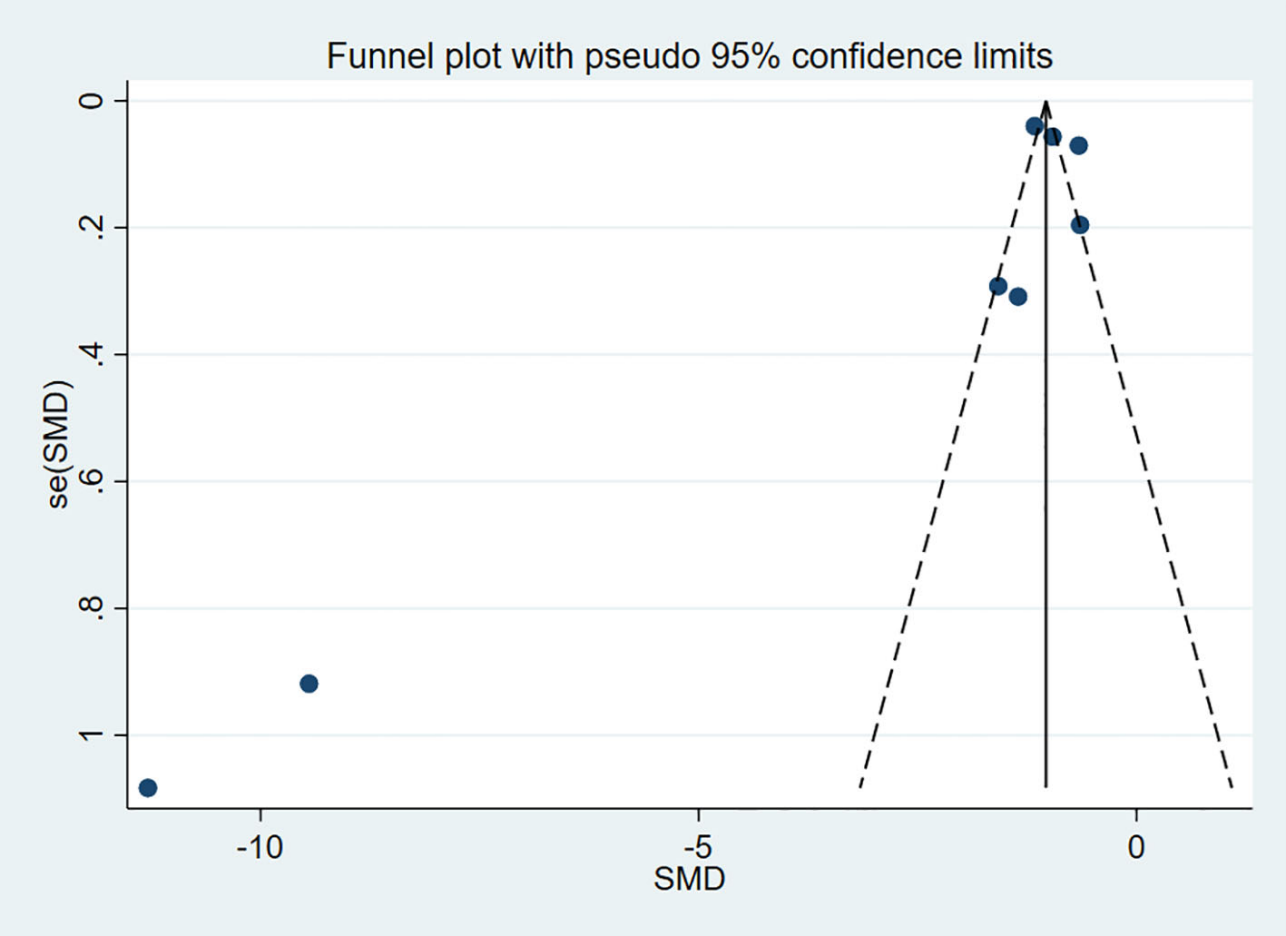
Funnel plot for LDL-C.

To assess publication bias for the outcome indicators in the included studies, funnel plots were used for visual representation, and Egger’s test was applied for further analysis. A P-value > 0.05 was considered indicative of no publication bias. The results indicated no publication bias for HDL-C (P = 0.963) and LDL-C (P = 0.155) (**Figures 13**, **14**).

For the sensitivity analysis, each study was systematically excluded one by one, and the remaining studies were reanalyzed to determine whether any single study had an excessive impact on the results. The sensitivity analysis showed that no single study excessively influenced the overall results, confirming that the meta-analysis results were stable and reliable.

## 4 Discussion

This study evaluated the lipid-modulating effects of combining CETP inhibitors with high-intensity statins, with particular focus on changes in HDL-C and LDL-C levels. LDL-C is typically considered the primary target for intervention in patients with hypercholesterolemia (24). Statins are the first-line therapy for lowering LDL-C, but their use, whether as monotherapy or in combination with other lipid-lowering agents, has not been fully optimized. For patients requiring further LDL-C reduction to reach their therapeutic goals, statin monotherapy is often insufficient. Consequently, there is a pressing need for additional lipid-lowering therapies for many patients whose LDL-C levels exceed ideal standards.

Cholesterol in plasma primarily exists in the form of cholesterol esters. CETP, a plasma glycoprotein secreted by the liver, is present in humans, non-human primates, and certain other animals. It facilitates the transfer of cholesterol esters and triglycerides between HDL, VLDL, and LDL particles (25). CETP activity typically facilitates the transfer of cholesterol esters from HDL to VLDL and LDL, while triglycerides are transferred from VLDL to LDL and HDL (26). CETP inhibition disrupts the transfer of cholesteryl esters from HDL to apoB-containing lipoproteins, augments transintestinal cholesterol excretion, upregulates the expression of scavenger receptor class B type 1 and hepatic LDL receptors, and enhances the catabolic efficiency of both LDL particles and apoB (27). When used in combination with statins, statins reduce intracellular cholesterol levels by inhibiting HMG-CoA reductase, thereby upregulating LDL receptor expression and enhancing the clearance of LDL particles from the circulation (28). CETP inhibitors complement this mechanism by increasing HDL-C levels and reducing LDL-C levels, which further stimulates LDL receptor expression (29). Early clinical trials demonstrated encouraging outcomes in patients with dyslipidemia treated with CETP inhibitors (30–33), but the development of these inhibitors encountered several disappointing challenges in later-stage trials.

The first CETP inhibitor evaluated in phase 3 clinical trials was torcetrapib, but the trial results revealed an increased risk of cardiovascular events and death, leading to the premature termination of the trial and cessation of torcetrapib’s development (13). Dalcetrapib had relatively modest effects, raising HDL-C levels, but its cardioprotective effects were not significant in the phase 3 dal-OUTCOMES trial, resulting in the termination of the project in 2012 (34). However, dalcetrapib may be effective in patients with specific genotypes, such as variations in the ADCY9 gene, and related research is ongoing. Evacetrapib showed promising results in raising HDL-C and lowering LDL-C in early trials, but the phase 3 ACCELERATE trial failed to demonstrate a significant reduction in cardiovascular events, leading to the discontinuation of the project in 2015 (35). Due to the premature termination of these clinical trials, significant gaps remain in our understanding. The mechanisms of adverse reactions observed in the later stages of the trials have not been fully elucidated. For example, the excess mortality associated with torcetrapib has been linked to off-target drug effects related to its unique structure (13), but the reasons for the electrolyte imbalance are still under question. Moreover, the early discontinuation of these trials has left us without long-term data on both efficacy and safety. Over time, such studies could have provided critical insights into the sustainability of treatment benefits and the potential for delayed adverse effects (36).

Despite these past challenges, current research is increasingly focused on the potential of CETP inhibitors to reduce cardiovascular risk by lowering LDL-C levels. Obicetrapib, an oral, once-daily, low-dose CETP inhibitor, is still under development. It has shown promise not only in treating dyslipidemia and lowering cardiovascular risk but also in the treatment of Alzheimer’s disease (37). Regarding lipid modulation, it effectively lowers LDL-C, non-HDL-C, ApoB, and LDL particle concentrations, particularly small LDL particles and lipoprotein(a), while raising mature HDL particles and ApoAI levels (38–41).

These findings align with our meta-analysis results. In Nicholls et al.’s study (16), the experimental group treated with obicetrapib combined with high-intensity statins demonstrated superior efficacy in raising HDL-C and ApoAI and lowering LDL-C and ApoB compared to other CETP inhibitors combined with statins. This suggests that obicetrapib holds potential for helping patients who have not reached their LDL-C goals, filling a gap in current therapies (42).

Another critical aspect of CETP inhibitors is their ability to lower ApoB. A study found that torcetrapib lowers VLDL, IDL, LDL, and ApoB levels primarily by increasing the clearance of ApoB100, while in combination with atorvastatin, torcetrapib reduces ApoB levels by enhancing VLDL ApoB100 clearance and decreasing IDL and LDL ApoB100 production (43). This study also showed that combining high-intensity statins with CETP inhibitors significantly reduced ApoB, indicating that obicetrapib may currently be the most effective CETP inhibitor for lowering both LDL-C and ApoB, particularly when combined with high-intensity statins.

Regarding safety, the Nicholls et al. study (22) did not report any significant differences in adverse events or off-target effects with obicetrapib. Similarly, this meta-analysis showed that combining high-intensity statins with CETP inhibitors did not increase adverse event rates. Furthermore, Harada et al. (44) found in their clinical study in Japan that the safety profile of obicetrapib as an adjunct to statin therapy was not influenced by racial differences. This also provides evidence for the potential safety of using this more potent CETP inhibitor either alone or in combination.

Our meta-analysis has several limitations that warrant consideration. First, the included studies exhibited significant heterogeneity, particularly in HDL-C and LDL-C outcomes, with I² values of 98.4% and 96.9%, respectively. This substantial heterogeneity may stem from variations in study design, patient populations, or the types of CETP inhibitors used. Although subgroup analysis by CETP inhibitor type did not fully resolve this issue, it highlights the complexity of interpreting pooled results. Additionally, some of the included studies employed cross-sectional designs, which limit the ability to draw causal conclusions. While these studies provide valuable insights into the association between statins, CETP inhibitors, and lipid-lowering effects, they cannot confirm a direct causal relationship. Future longitudinal studies are needed to establish whether the observed benefits are directly attributable to the combination therapy. Furthermore, due to our stringent restriction on the daily dose of statin therapy, the relatively small sample sizes of some included studies may limit the statistical power and generalizability of our findings.

Another limitation is the potential for bias in some studies due to insufficient reporting of outcome measurement methods. It has been noted that the indirect measurement of LDL-C using the Friedewald equation may underestimate true levels, potentially exaggerating the effects of CETP inhibitors, while the beta-quantification method, a more accurate approach, has not been widely adopted (45, 46). This may introduce variability in the interpretation of lipid-lowering effects and safety outcomes. Future studies should strive for standardized measurement protocols and transparent reporting to enhance the reliability of findings.

Finally, existing research has shown that combining obicetrapib with ezetimibe, in addition to high-intensity statin therapy, can significantly reduce atherogenic lipid and lipoprotein parameters while maintaining good safety and tolerability (38). Although we did not include studies investigating triple-drug combinations, it is important to recognize the potential of these combination therapies, such as CETP inhibitors used alongside ezetimibe or PCSK9 inhibitors in conjunction with high-intensity statins. These combinations may further reduce LDL-C and other atherogenic lipid parameters, offering a more comprehensive approach to lipid management.

Despite the less-than-ideal results of CETP inhibitors in early clinical trials, recent studies highlight their potential in reducing LDL-C levels and cardiovascular risk. Obicetrapib, a new CETP inhibitor, has demonstrated significant lipid-lowering effects and good safety profiles when used alongside statin therapy. While the effects of different CETP inhibitors vary, and evidence for the combination of CETP inhibitors with high-intensity statins has been lacking, this meta-analysis fills this gap. However, to further strengthen the evidence base for personalized lipid-lowering strategies and improve cardiovascular disease prevention, future large-scale, multi-center randomized controlled trials are needed. These studies should focus on long-term follow-up to comprehensively evaluate the safety and efficacy of CETP inhibitors in combination with high-intensity statins, particularly in diverse patient populations and under varying clinical conditions.

## 5 Conclusion

This meta-analysis demonstrates that combining high-intensity statins with CETP inhibitors significantly increases HDL-C and reduces LDL-C, TG, and ApoB levels without increasing adverse events. Obicetrapib showed the most pronounced lipid-lowering effects, while dalcetrapib had limited HDL-C elevation. Despite high heterogeneity and small sample sizes in some studies, these findings suggest that combination therapy, particularly with obicetrapib, may benefit patients not achieving optimal lipid control with statin monotherapy. Further large-scale trials are needed to confirm long-term efficacy and safety.

## Data Availability

The original contributions presented in the study are included in the article/**Supplementary Material**, further inquiries can be directed to the corresponding author/s.
