# Supplementary material for: Lipid-lowering effect of combined therapy with high-intensity statins and CETP inhibitors: a Systematic Review and meta-analysis
Source: Front Endocrinol (Lausanne). 2025 May 1;16:1512670. doi: 10.3389/fendo.2025.1512670 (PMC12078159; doi:10.3389/fendo.2025.1512670)
Supplement: Supplementary file 1 [file DataSheet1.zip › Raw Data/Raw Data/5. Risk of bias/ROB-2复件/随机对照试验偏倚风险评价工...B2(2019修订版)解读_刘津池.pdf]

· 方法学 ·

随机对照试验偏倚风险评价工具 RoB2（2019 修订版）解读

刘津池，刘畅，华成舸

口腔疾病研究国家重点实验室，国家口腔疾病临床医学研究中心，四川大学华西口腔医院口腔颌面外科，循证口腔医学教研室（成都 610041）

【摘要】 随机对照试验偏倚风险的工具 RoB2（2019 修订版）在原有版本的基础上进行了更新和改进。本文从 RoB2（2019 修订版）的制订背景、主要内容及新增软件的操作过程进行详细阐述和解读。与上一版 RoB2（2018 修订版）相比，RoB2（2019 修订版）具有内容丰富、细节完善、问题精准、操作简便等优点，且对于研究人员及初学者更加友好，对于不同类型随机对照试验的偏倚风险评价更为全面和准确，是权威的、值得信赖和推广的医疗实践中随机对照试验偏倚风险的评价工具。

【关键词】 随机对照试验；偏倚风险；评价工具；系统评价

Risk bias assessment tool RoB2 (revised version 2019) for randomized controlled trial : an interpretation

LIU Jinchi, LIU Chang, HUA Chengge

State Key Laboratory of Oral Diseases & National Clinical Research Center for Oral Diseases & Dept. of Oral and Maxillofacial Surgery & Dept. of Evidence-Based Dentistry, West China Hospital of Stomatology, Sichuan University, Chengdu 610041, P.R.China

Corresponding author: HUA Chengge, Email: huacg@163.com

【Abstract】 RoB2 (revised version 2019), an authoritative tool for assessing the risk of bias in randomized controlled trials, has been updated and improved based on the original version. This article elaborated and interpreted the background and main content of RoB2 (revised version 2019), as well as the operation process of the new software. Compared with the previous version of RoB2 (revised version 2018), RoB2 (revised version 2019) has the advantages of rich content, complete details, accurate questions, and simple operation, etc. Additionally, it is more user-friendly for researchers and beginners. The risk bias assessment of randomized controlled trials is more comprehensive and accurate, and it is an authoritative, trustworthy, and popular tool for evaluating the risk of bias in randomized controlled studies in medical practice.

【Key words】 Randomized controlled trial; Risk of bias; Tool for assessment; Systematic review

1 RoB2（2019 修订版）的制订背景

在医疗卫生实践中评价某种药物或治疗方法等干预措施最可靠的临床研究设计方案是随机对照试验（randomized controlled trial，RCT）[1]。高质量的 RCT 能够最大限度地平衡组间基线上可能存在的混杂因素，提高数据分析的有效性，提供真实的医学证据。然而，随机化过程的不严谨、盲法实施的失败、设置对照参考的失当、随访过程中失访率过高等各种情况，会造成 RCT 方法学质量较低、偏倚风险较大，进而影响研究结论的内部真实性。因此，应用权威的评价工具对 RCT 的偏倚风险做出全面客观的评价对循证医学的重要性不言而喻。

在上个世纪，针对 RCT 偏倚风险的评价工具主要采用两种形式[2]：一是量表式（scale），例如 Jadad 量表[3]；二是清单式（checklist），例如 Weintraub 清单[4]。Cochrane 协作网 RCT 偏倚风险评价工具（Cochrane collaboration’s tool for assessing risk of bias in randomized trial，RoB1）作为第三种形式—领域式评价工具（domain-based evaluation）于 2008 年[5]问世，2011 年更新[6, 7]，解决了以往评价工

DOI：10.7507/1672-2531.202011144
基金项目：四川大学华西口腔医院科研项目（编号：LCYJ2019-7）
通信作者：华成舸，Email：huacg@163.com

(C)1994-2022 China Academic Journal Electronic Publishing House. All rights reserved. http://www.cnki.net

具的常见问题，如量表式评分权重不合理、清单式评价条目过于冗杂等，成为了过去十余年系统评价的主流评价工具。

但 RoB1 在应用中也暴露出一些问题，例如：没有考虑整群随机设计、交叉设计等特殊类型的 RCT，未正视组间基线的均衡性，未能明确界定干预措施分配的效果及干预措施依从的效果，遗漏了组间沾染等问题[8]。为此，Cochrane 方法学组于 2016 年 10 月 20 日重新推出新版 RCT 偏倚风险评价工具（Revised Cochrane risk-of-bias tool for randomized trials，又名 Version 2 of the Cochrane tool for assessing risk of bias in randomised trial，RoB2）[9, 10]。RoB2 相较于 RoB1 覆盖了包括整群 RCT、交叉试验等更多设计类型的 RCT，注重采用恰当的统计分析方法控制偏倚。RoB2 在 RoB1 基础上变得更加详尽而全面，因而操作也相对复杂[11]。对于 RoB2 的完善工作从未停止，于 2018 年 10 月 9 日[1, 12]、2019 年 8 月 22 日[13]进行了两次重要更新，分别收录于 2019 年第 6.0 版[14]和 2020 年第 6.1 版[15] Cochrane 手册中。杨智荣等[8]针对 RoB2（2016 初版）、刘括等[1]针对 RoB2（2018 修订版）均进行了系统解读。而最新版本即 2019 年 8 月 22 日修订版 RoB2 除了部分信号问题及评价标准有所调整外，还发布了一个带有宏的可运行 Excel 文件作为评价软件。除了针对个体平行设计 RCT 的 RoB2 工具，该工具研发团队在其网站于 2020 年 11 月 10 日[16]和 2020 年 12 月 8 日[17]，还更新了评价整群随机试验及交叉试验偏倚风险的 RoB2 扩展版本，作为对 RoB2（2019 修订版）的补充，拓宽了该工具的适用范围。本文旨在解读 RoB2（2019 修订版）的信号问题及评价标准，并简单介绍软件操作过程。

2　RoB2（2019 修订版）的解读

与 2016 版 RoB2 相同，2019 修订版 RoB2 设置了 5 个评价领域：随机化过程中的偏倚、偏离既定干预措施的偏倚、结局数据缺失的偏倚、结局测量的偏倚和选择性报告结果的偏倚。其中，偏离既定干预措施的偏倚领域按照不同的研究目的分为了两种情况：一是研究干预措施分配的效果，二是干预措施依从的效果。

每个领域下有多个不同信号问题。研究人员在评价 RCT 的偏倚风险时，需做出判断并客观地回答这些问题。信号问题一般有五种供选答案：是（Yes，Y）、很可能是（Probably Yes，PY）、很可能否（Probably No，PN）、否（No，N）、没有信息（No Information，NI）。个别信号问题不允许回答 NI。有些信号问题之间有逻辑关联，即可能因为前面的信号问题选了某个选项而跳过后面的信号问题；如果信号问题因为这种逻辑设置而被跳过了，则被记为不适用（Not Applicable，NA）。当分析多位评阅者对某一研究的偏倚风险评价是否一致时，需将每道信号问题中的 Y、PY、N、PN、NI、NA 作为问题的同一性质答案。各领域的具体评价方法详见表 1。

RoB2 每个领域偏倚风险的决策路径见图 1～6。根据评阅者对信号问题的回答，每个领域的偏倚风险可分为三个等级：“低风险”（low risk of bias）、“有一定风险”（some concerns）及“高风险”（high risk of bias）。如果所有领域的偏倚风险评价结果都是“低风险”，那么整体偏倚风险（overall risk of bias）就是“低风险”；如果有的领域的偏倚风险评价结果为“有一定风险”且不存在“高风险”的领域，那么整体偏倚风险为“有一定风险”；只要有一个领域偏倚风险评价结果是“高风险”，那么整体偏倚风险就是“高风险”。

此外，RoB2 对每个领域还给出了预计偏倚方向（predicted direction of bias）的选项，以此来评价偏倚的大小和方向：对试验组有利（favours experimental）、对对照组有利（favours comparator）、趋于零（towards null）、远离零（away from null）、无法预计（unpredictable）、不适用。值得注意的是，如果选择特定的效应量大小（趋于零、远离零）或方向（对试验组有利、对对照组有利）需要敏感性分析等严谨的统计分析结果来支撑对偏倚风险评价的判断，如果无法评价偏倚的大小和方向则应该选择“无法预计”。

3　RoB2（2019 修订版）Excel 文件操作过程

RoB2（2019 修订版）新增了一个启用宏的 Excel 文件。这份官方提供的文件使得评价工作更加程序化、标准化，同时也大大降低了人为因素的干扰。为了方便研究人员更好地利用这款软件，笔者结合一篇已发表的系统评价[18]对 RoB2（2019 修订版）操作过程予以介绍。

3.1　偏倚风险评价流程

① 在 Risk of bias 官网（https://www.riskofbias.info/）下载并打开 Excel 文件，运行宏，单击“Rob 2 Assessment Form”（RoB2 评价窗体）按钮以初始化用户表格；② 为方便日后查找评价结果，给每一次评价设置一个“Unique ID”（唯一 ID），然后在

(C)1994-2022 China Academic Journal Electronic Publishing House. All rights reserved.　http://www.cnki.net

表 1 各领域评价的信号问题及供选答案

领域	信号问题	供选答案*
随机化过程中的偏倚	1.1 分配序列是否随机？	Y/PY/PN/N/NI
	1.2 直至受试者参加并分配到干预措施，分配序列是否隐藏？	Y/PY/PN/N/NI
	1.3 组间基线差异是否提示随机化过程中有问题？	Y/PY/PN/N/NI
偏离既定干预措施的偏倚（干预措施分配的效果）	2.1 在试验中受试者是否知道他们分配到哪种干预措施？	Y/PY/PN/N/NI
	2.2 在试验中护理人员和干预措施提供者是否知道受试者分配到哪种干预措施？	Y/PY/PN/N/NI
	2.3 若 2.1 或 2.2 回答 Y/PY/NI：是否存在由于研究环境造成的偏离既定干预措施的情况？**	NA/Y/PY/PN/N/NI
	2.4 若 2.3 回答 Y/PY：偏离既定干预措施的情况是否很可能影响结局？	NA/Y/PY/PN/N/NI
	2.5 若 2.4 回答 Y/PY/NI：偏离既定干预措施的情况是否在组间均衡？	NA/Y/PY/PN/N/NI
	2.6 是否采用了恰当的分析方法估计干预措施分配的效果？	Y/PY/PN/N/NI
	2.7 若 2.6 回答 N/PN/NI：分析受试者时分组错误是否有（对结果）造成实质影响的潜在可能？	NA/Y/PY/PN/N/NI
偏离既定干预措施的偏倚（干预措施依从的效果）	2.1 在试验中受试者是否知道他们分配到哪种干预措施？	Y/PY/PN/N/NI
	2.2 在试验中护理人员和干预措施提供者是否知道受试者分配到哪种干预措施？	Y/PY/PN/N/NI
	2.3［如果适用］若 2.1 或 2.2 回答 Y/PY/NI：重要的计划外的干预措施是否在组间均衡？	NA/Y/PY/PN/N/NI
	2.4［如果适用］未完成干预措施的情况是否有可能影响结局？	NA/Y/PY/PN/N/NI
	2.5［如果适用］不依从干预措施的情况是否有可能影响受试者结局？	NA/Y/PY/PN/N/NI
	2.6 若 2.3 回答 N/PN/NI，或 2.4 或 2.5 回答 Y/PY/NI：是否采用了恰当的分析方法估计干预措施依从的效果？	NA/Y/PY/PN/N/NI
结局数据缺失的偏倚	3.1 是否可以获取全部或者几乎全部受试者的结局数据？	Y/PY/PN/N/NI
	3.2 若 3.1 回答 N/PN/NI：是否有证据证明结局数据的缺失没有对结果造成偏倚？	NA/Y/PY/PN/N
	3.3 若 3.2 回答 N/PN：结局数据的缺失是否有可能依赖于其真值？	NA/Y/PY/PN/N/NI
	3.4 若 3.3 回答 Y/PY/NI：结局数据的缺失是否很可能依赖于其真值	NA/Y/PY/PN/N/NI
结局测量的偏倚	4.1 结局测量方法是否不恰当？	Y/PY/PN/N/NI
	4.2 结局测量或认定是否有可能有组间差异？	Y/PY/PN/N/NI
	4.3 若 4.1 回答 N/PN/NI：结局测量者是否知道受试者接受到哪种干预措施？	NA/Y/PY/PN/N/NI
	4.4 若 4.3 回答 Y/PY/NI：如果知道接受哪种干预措施，是否有可能影响结局测量？	NA/Y/PY/PN/N/NI
	4.5 若 4.4 回答 Y/PY/NI：如果知道接受哪种干预措施，是否很可能影响结局测量？	NA/Y/PY/PN/N/NI
选择性报告结果的偏倚	5.1 结果的数据分析是否与在获取揭盲的结局数据之前就已预先确定的分析计划相一致？	Y/PY/PN/N/NI
	5.2 正在评价的数值结果是否很可能是从多个合格的结局测量（例如：多个分值、多个定义标准、多个时间点）的结果中选择性报告的？	Y/PY/PN/N/NI
	5.3 正在评价的数值结果是否很可能是从多个合格的数据分析的结果中选择性报告的？	Y/PY/PN/N/NI

*：绿色下划线的答案是低偏倚风险的潜在标志，红色标识的答案是偏倚风险的潜在标志。如果问题仅与其他问题相关，则不使用标记；**：未采用盲法或盲法失败造成的偏离属于由于研究环境造成的偏离既定干预措施，例如：对照组的受试者因知道自己是对照组而沾染试验组的干预措施，实施者因知道分组情况而破坏研究计划。但是注意，因为受试者发生严重毒副作用而按照规范流程提前破盲不属于由于研究环境造成的偏离既定干预措施。

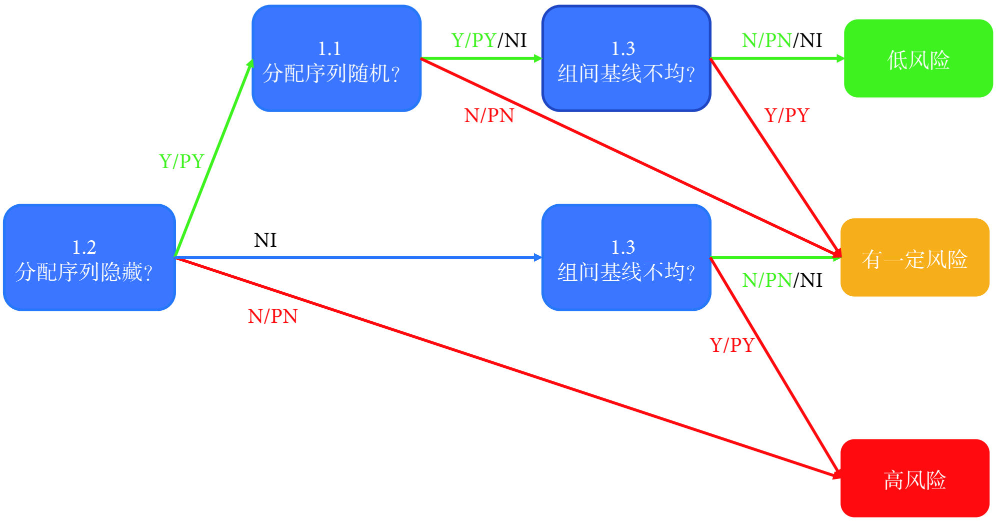

图 1 随机化过程中的偏倚领域决策路径图

(C)1994-2022 China Academic Journal Electronic Publishing House. All rights reserved. http://www.cnki.net

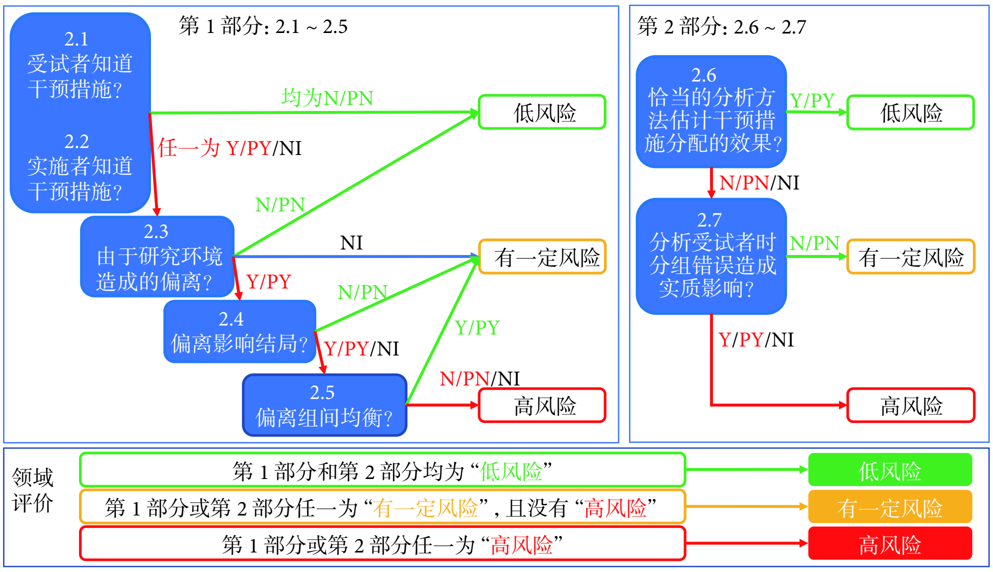

图 2　偏离既定干预措施的偏倚（干预措施分配的效果）领域决策路径图

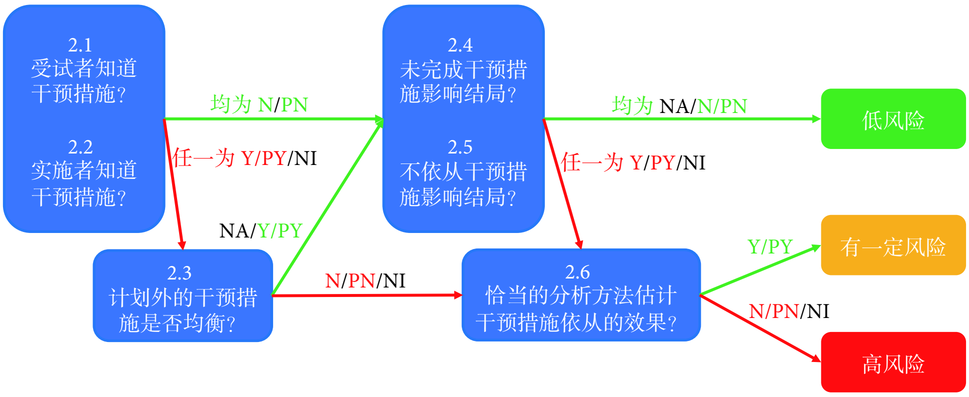

图 3　偏离既定干预措施的偏倚（干预措施依从的效果）领域决策路径图

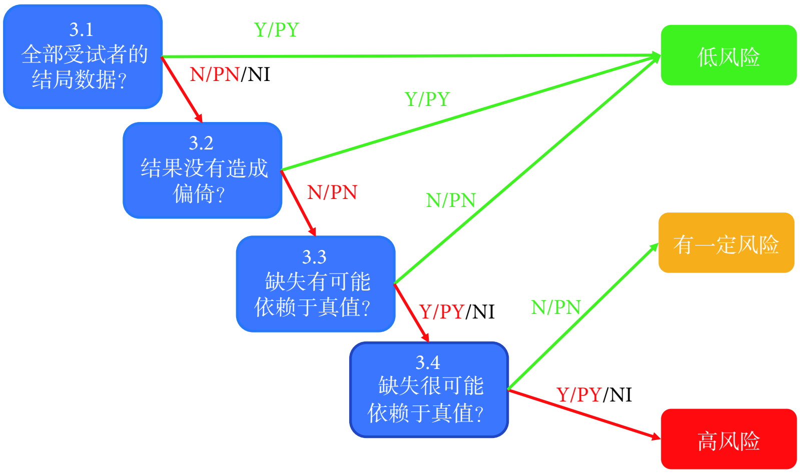

图 4　结局数据缺失的偏倚领域决策路径图

表格相应位置（图 7a）输入该 ID，ID 可以为字母或数字及组合；③ 在完成与评价相关信息录入后，需回答每个领域下信号问题（图 7b），信号问题的答案可以从下拉菜单中选择，并可以在后面“Description”一栏中阐述相应理由，双击信号问题可出现相应问题的解释；④ RoB2 软件可以根据信号问题的答案自动判断每个领域的偏倚风险，点击“Algorithm”（运算）按钮（图 7c），系统即可自动得出判断结果；⑤ 若两位评阅者要独立进行评价，则需使用相同的 ID 进入同一个评价中，最后

(C)1994-2022 China Academic Journal Electronic Publishing House. All rights reserved.　http://www.cnki.net

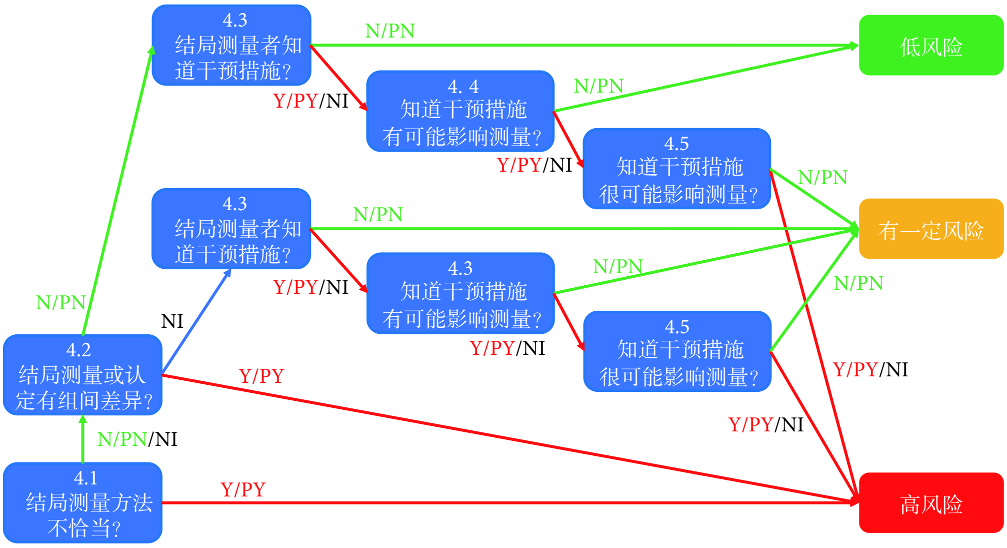

图 5　结局测量的偏倚决策路径图

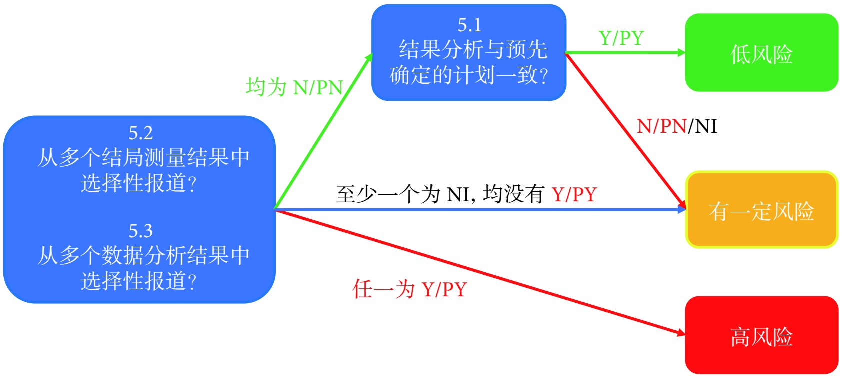

图 6　选择性报告结果的偏倚决策路径图

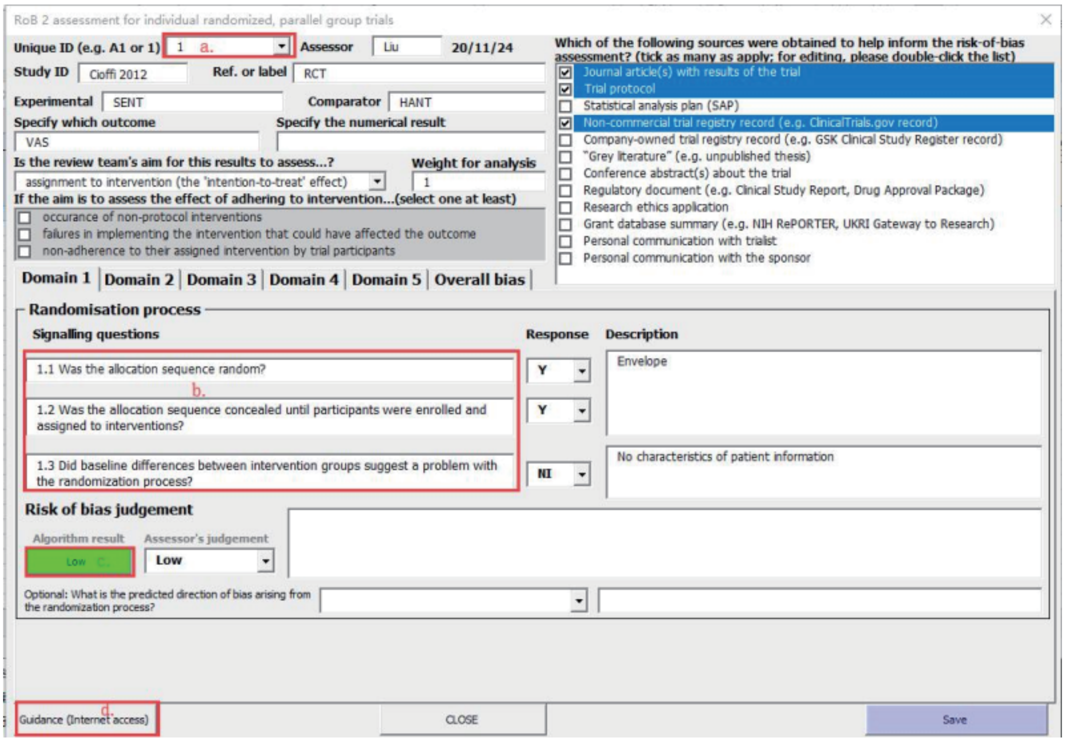

图 7　RoB2（2019 修订版）Excel 偏倚风险评价的宏对话框

(C)1994-2022 China Academic Journal Electronic Publishing House. All rights reserved.　http://www.cnki.net

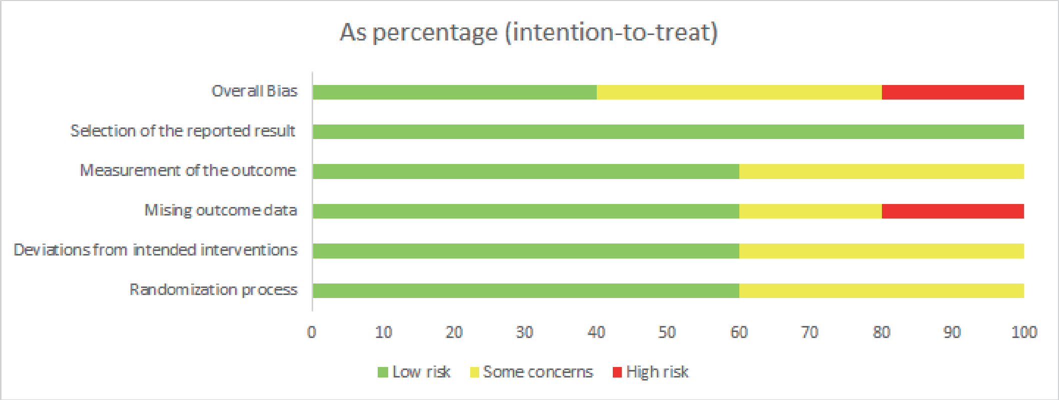

图 8　RoB2（2019 修订版）Excel 输出的偏倚风险图

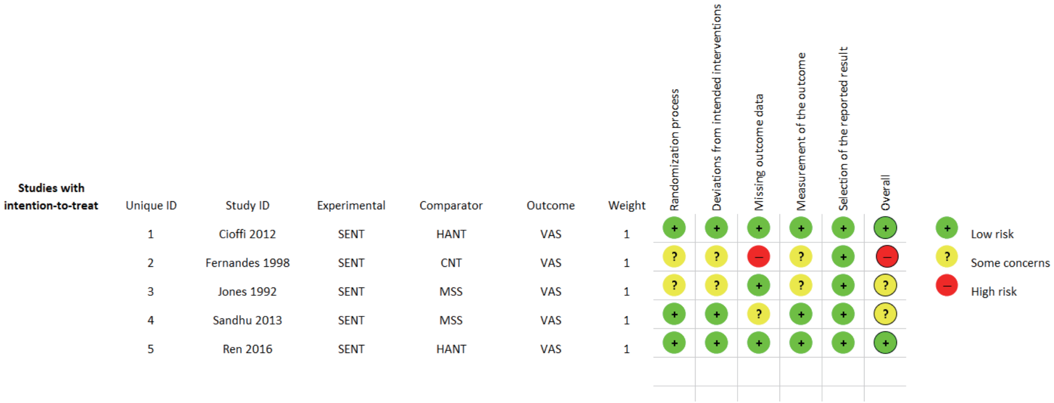

图 9　RoB2（2019 修订版）Excel 输出的偏倚风险总结图

还要检查两者答案的差异；⑥ 点击“保存”按钮，完成评价；⑦ 如有疑问还可以点击“Guidance”按钮（图 7d）以获取更多信息。

若要编辑或删除现有评价，则在交互式表单及结果表中直接编辑或删除即可。但系统对领域的偏倚风险判断不会自动更新，如果已对信号问题的答案进行了修改，则需再次单击“Algorithm”（运算）按钮，完成偏倚风险判断。

3.2　偏倚风险图和偏倚风险总结图的绘制

完成偏倚风险评价后，单击“Intro”（介绍）页上的“Summary”（总结）按钮，所有的评价结果都将出现在“Summary”（总结）表中，并且在工作表的右侧系统将自动绘制出偏倚风险图（图 8），更加直观地展示所有纳入研究在各个领域中的不同风险的构成比例。单击“Intro”（介绍）页中的“Figures”（图表）按钮，即可在“Figures”（图表）表中自动绘制出偏倚风险总结图（图 9），该图总结了每个纳入研究在每个领域的偏倚风险及整体偏倚风险的等级评价结果。此外，可以使用 Excel 常规功能如添加边框和线条等，可以进一步编辑图表，使其更加美观。

3.3　双人核查

检查 2 个评价者的评价结果，并输出最终的一致判断。在使用此功能之前，需务必备份评价结果（评阅人 1 的结果），因为第一评阅人的答案将被最后的结果覆盖。其具体步骤如下：① 将两个评阅者的结果复制粘贴到“Check”（核查）表中；② 确保“Unique ID”（唯一 ID）与所要差异检查的评价相对应；③ 点击“Intro”（介绍）页上的“Discrepancy Check”（差异核查）按钮，会出现一个交互表格；④ 评阅者的答案并排显示。若两位评阅人做出的判断不同，则判断将以红色显示；⑤ 编辑最终的判断结果并保存。然后可以使用如前所述的“Summary”（总结）、“Figures”（图表）等功能生成所需结果。

4　讨论

2016 年，RoB2 一经发布，就吸引了大量的目光。相比于 RoB1，RoB2 具有内容丰富、细节完善等优点。同时，RoB2 完善了评价流程和细节，还删减了大量模糊的内容及专业的术语，对于问题的描述更加准确和直接。因此，RoB2 比 RoB1 更加适用

(C)1994-2022 China Academic Journal Electronic Publishing House. All rights reserved.　http://www.cnki.net

于 RCT 的偏倚风险评价。然而，RoB2 仍有可靠性较低[11]等缺点，其修订工作也从未停止。对比 RoB2（2018 修订版），RoB2（2019 修订版）主要有以下两个显著特点。

4.1 对部分信号问题进行了调整和改良

对信号问题进行简化：RoB2（2019 修订版）对“结局数据缺失偏倚”领域的信号问题 3.3 ~ 3.5 进行了变动，删除了原版 3.4 问题；对“偏离既定干预措施的偏倚（干预措施依从的效果）”领域的信号问题 2.3 ~ 2.5 进行变动，除了调整信号问题的措辞与答案外，评价者还可以依据研究内容的适用情况而酌情跳过或保留相关信号问题。这样的变动去除了冗杂的问题，使信号问题变得更加清晰和明确，简化了评价过程。

对部分术语进行了严谨地修改或限制，比如：在“偏离既定干预措施的偏倚（干预措施依从的效果）”领域，将“co-intervention”（干扰，辅干扰）换成“non-protocol intervention”（计划外的干预措施）；在“选择性报告结果的偏倚”领域，在 5.2 ~ 5.3 中添加“eligible”（合格的）等限制。这些修改使得 RoB2 更加严谨而通俗，同时也避免了不必要误解。

4.2 增加了启用宏的 Excel 文件

以往的量表只给予评价的方法，但是没有提供一个进行评价的平台。这一次 RoB2（2019 修订版）提供了启用宏的 Excel 文件，为评价人员减少了工作量。在经过实际操作后，笔者所感受到的具体优点如下：

① 操作简便快捷：只需要按照启用宏的 Excel 文件上的提示，输入相应的内容，即可完成评价流程。减少了评价者以往的评价时间，使评价工作变得高效，同时也提高了评价的质量。

② 流程标准规范：对于研究者尤其是刚接触偏倚风险评价的人来说，该份启用宏的 Excel 文件提供了一个标准的流程，且对于每个操作步骤及流程都做了详细的解释说明，大大减小了人为因素对评价结果的干扰。

③ 功能丰富强大：该 Excel 宏对于研究者的操作系统和软件要求低，并且其功能强大，集分析、汇总、绘图、打印、核查等功能于一身。在进行评价偏倚风险的同时，该操作系统即可完成绘制图表工作，使得评价结果更加直观，同时也省去了评价者在多个软件进行分析的时间。

最新版的 RoB2 对于 2018 版及更早的 RoB1 有了较大的飞跃。新一版的量表提供并整合了评价的软件工具，经过测试，完成新版量表的时间比旧版时间明显减少，大大提高评阅者评价工作的效率也增强了 RoB2 的可操作性。对于相当一部分刚接触评价工作的初学者，该软件也降低了评价工作的难度。尽管如此，在应用 RoB2 量表之前，评阅者仍需进行培训和练习以提高评价结果的可靠性。另外对于前一版的部分冗杂且模糊的信号问题，新一版的量表也做出了删改，减少了评价者的困扰及出错的机会。

综上，最新一版的 RoB2 对于研究人员及初学者比较友好，对于 RCT 的偏倚风险评价较为全面和准确，是权威的，值得信赖和推广的评价医疗实践中随机对照研究设计偏倚风险的工具。

参考文献

1 刘括, 孙殿钦, 廖星, 等. 随机对照试验偏倚风险评估工具 2.0 修订版解读. 中国循证心血管医学杂志, 2019, 11(3): 284-291.

2 Moher D, Jadad AR, Tugwell P. Assessing the quality of randomized controlled trials. Current issues and future directions. Int J Technol Assess Health Care, 1996, 12(2): 195-208.

3 Jadad AR, Moore RA, Carroll D, *et al.* Assessing the quality of reports of randomized clinical trials: is blinding necessary? Control Clin Trials, 1996, 17(1): 1-12.

4 Weintraub M. How to critically assess clinical drug trials. Drug Ther, 1982, 12: 131-148.

5 Higgins JPT, Altman DG. Chapter 8: Assessing risk of bias in included studies. In: Higgins JPT, Green S. Cochrane handbook for systematic reviews of interventions version 5.0. 0 (updated February 2008). The Cochrane Collaboration, 2008. Available at: https://training.cochrane.org/handbook/archive/v5.0.0/.

6 Higgins JPT, Altman DG, Sterne JAC, *et al.* Chapter 8: Assessing risk of bias in included studies. In: Higgins JPT, Green S. Cochrane handbook for systematic reviews of interventions version 5.1. 0 (updated March 2011). The Cochrane Collaboration, 2011. Available at: https://training.cochrane.org/handbook/archive/v5.1/.

7 Higgins JPT, Altman DG, Gøtzsche PC, *et al.* The Cochrane Collaboration's tool for assessing risk of bias in randomised trials. BMJ, 2011, 343: d5928.

8 杨智荣, 孙凤, 詹思延. 偏倚风险评估系列: (二)平行设计随机对照试验偏倚评估工具 2.0 介绍. 中华流行病学杂志, 2017, 38(9): 1285-1291.

9 Higgins JPT, Sterne JAC, Savović J, *et al.* A revised tool for assessing risk of bias in randomized trials. In: Chandler J, McKenzie J, Boutron I, *et al.* Cochrane methods. Cochrane Database Syst Rev, 2016, 10(Supp 1): CD201601.

10 Higgins JPT, Sterne JAC, Savović J, *et al.* Revised Cochrane risk of bias tool for randomized trials (RoB 2.0), 2016-10-20. Available at: https://www.riskofbias.info/welcome/rob-2-0-tool/archive-rob-2-0-2016/.

11 Minozzi S, Cinquini M, Gianola S, *et al.* The revised Cochrane risk of bias tool for randomized trials (RoB 2) showed low interrater reliability and challenges in its application. J Clin Epidemiol, 2020, 126: 37-44.

(C)1994-2022 China Academic Journal Electronic Publishing House. All rights reserved. http://www.cnki.net

12 Higgins JPT, Sterne JAC, Savović J, *et al*. Revised Cochrane risk-of-bias tool for randomized trials (RoB 2), 2018-10-09. Available at: https://www.riskofbias.info/welcome/rob-2-0-tool/current-version-of-rob-2/.
13 Sterne JAC, Savović J, Page MJ, *et al*. RoB 2: a revised tool for assessing risk of bias in randomised trials. BMJ, 2019, 366: l4898.
14 Higgins JPT, Savović J, Page MJ, *et al*. Chapter 8: Assessing risk of bias in a randomized trial. In: Higgins JPT, Thomas J, Chandler J, *et al*. Cochrane handbook for systematic reviews of interventions version 6.0 (updated July 2019). The Cochrane Collaboration, 2019. Available at: ttps://training.cochrane.org/handbook/archive/v6/chapter-08/.
15 Higgins JPT, Savović J, Page MJ, *et al*. Chapter 8: Assessing risk of bias in a randomized trial. In: Higgins JPT, Thomas J, Chandler J, *et al*. Cochrane handbook for systematic reviews of interventions version 6.1 (updated September 2020). The Cochrane Collaboration, 2020. Available at: https://training.cochrane.org/handbook/current/chapter-08/.
16 Higgins JPT, Campbell MK, Campbell MJ, *et al*. Additional considerations for cluster-randomized trials (RoB 2 CRT), 2020-11-10. Available at: https://www.riskofbias.info/welcome/rob-2-0-tool/current-version-of-rob-2/.
17 Higgins JPT, Tianjing L, Jonathan S, *et al*. Additional consideration for crossover trials, 2020-12-08. Available at: https://www.riskofbias.info/welcome/rob-2-0-tool/current-version-of-rob-2/.
18 刘畅, 王艳, 潘韦霖, 等. 初始弓丝材料对正畸治疗初始疼痛影响的系统评价与网状 Meta 分析. 华西口腔医学杂志, 2018, 36(3): 296-300.

收稿日期：2020-11-25　修回日期：2021-02-05
本文编辑：熊鹰

(C)1994-2022 China Academic Journal Electronic Publishing House. All rights reserved.　http://www.cnki.net
