# Supplementary material for: Lipid-lowering effect of combined therapy with high-intensity statins and CETP inhibitors: a Systematic Review and meta-analysis
Source: Front Endocrinol (Lausanne). 2025 May 1;16:1512670. doi: 10.3389/fendo.2025.1512670 (PMC12078159; doi:10.3389/fendo.2025.1512670)
Supplement: Supplementary file 1 [file DataSheet1.zip › Raw Data/Raw Data/5. Risk of bias/ROB-2复件/Risk of Bias 2.0具体项目.pdf]

随机对照研究的偏倚风险评价工具2.0		
**偏倚域**	**信号问题**	**判断结果**
**1. 随机化过程中产生的偏倚**		
1.1 研究对象是否随机分配?	“Y”：在序列产生过程中描述了随机化方法。例如：使用计算机生成随机数字；使用随机数字表；抛硬币；洗牌或信封法；掷骰子；抽签。“最小化法”也是基于随机原理的分组方法，属于随机分配“N”：在分组过程中没有使用随机分组方法，或者分组可以被预测。例如：有选择性入组；基于日期(如生日，就诊日期、就诊顺序)、病历号的选择方法；由医生或患者自行决定组别；根据干预的可及性进行分组；或其他造成偏倚的分组方法“NI”：原文仅陈述了“随机”，但没有进一步描述随机化方法“PY”“PN”：对于一项大型研究，如果是一个独立的中心承担或研究是以监管为目的，则可认为分配过程是随机的。如果同一研究团队的其他试验(同期)明确未使用随机化分配，当前试验也可认为未随机化	Y/PY/PN/N/NI
1.2 在受试者入组前是否实施分配隐藏?	“Y”：使用远程或集中管理方法分配干预措施，并且分配过程是由与入组实施者无关的第三方机构完成(如独立的药企，电话或网络随机过程的服务提供者)“Y”：信封和药品包装使用恰当。信封应该是按顺序编码，要求不透光密封包装，并有防撕毁封口。药物包装也应该顺序编码，且外观一致。如果本部分未在原文中详细提及，可酌情判断为“可能是”或“可能否”“N”：入组实施者或研究对象可以预知分组	Y/PY/PN/N/NI
1.3 随机化过程是否导致了基线间的不均衡?	由随机误差导致的组间差异不会造成偏倚“N”：基线组间均衡或组间差异是由随机误差所致(组间可比)“Y”：由随机过程问题导致了基线组间不可比，包括：两组间样本量差异较大，超出了预计的分配比例；两组间基线特征差异有统计学意义；一个或多个重要的预后因素或结局变量的基线测量间有差异，且不是由随机误差所致；或组间差异大到会给干预效应评价带来偏倚；基线特征间过于相似，无法用随机误差解释；应报告的重要基线特征未被提及“NI”：原文中未陈述有效的基线信息本问题的答案不应影响1.1和1.2的答案。如：一项试验的基线组间有较大的不均衡，但作者报告使用了恰当的随机化方法，问题1.1和1.2应该按照作者报告的随机化方法来评判，所有关于组间均衡的问题均在1.3中评价，并反映到随机过程偏倚的整体评价中研究者也许会通过统计分析方法控制基线组间不均衡，以此弥补随机化过程中出现的问题。为了去除随机化过程带来的偏倚风险，应该识别和测量所有在基线时不均衡的预后因素。但是，由于无法对所有重要的预后因素进行识别和测量，所以最多只能降低偏倚风险。如果综述作者希望在控制基线不均衡的试验研究中评估偏倚风险，需要用ROBINS-I工具评估	Y/PY/PN/N/NI
偏倚风险评价(低风险/高风险/可能存在风险)		
**2. 偏离既定干预的偏倚——(1)干预分配**		
2.1 研究对象是否在试验过程中知晓自己的分组?	如果研究对象知晓了自己的分组，其健康相关行为会有所改变。对研究对象采用盲法(安慰剂)可避免这种健康相关行为的改变“Y/PY”：研究对象通过不良反应等判断出了自己处于某个干预组	Y/PY/PN/N/NI
2.2 医护人员或试验实施者是否在试验过程中知晓分组?	如果医护人员知晓分组信息，会对干预的实施和管理产生影响。对医护人员设盲(安慰剂)可避免该种影响“Y/PY”：医护人员通过不良反应等判断出研究对象处于某个干预组。如果随机化方案中没有隐藏分组，则医护人员很有可能知晓研究对象的分组	Y/PY/PN/N/NI
**2.3如果2.1或者2.2回答“Y/PY/NI”，是否由于试验环境而导致出现了偏离预期干预的情况?**	当评价关注干预分配时，只有与试验内容有关的偏差才能造成偏倚。“Y”：研究对象觉得被分配到对照组很“不幸”，进而寻求与试验组相同的干预，或其他干预措施。由于患者对试验组和对照组的预期不同，而这种预期不同带来的偏差并不是常规医疗实践中的一部分，因而，在评估干预效果时并不能反映干预在实践中的真实效果“N/PN”：试验中发生的偏离在常规医疗中也会出现。①由于药物的不良反应导致的停药；②干预措施的不依从；③针对干预结果做出的“联合干预措施”。由于某些干预措施有特殊的不良反应，因此在试验中不能施行盲法。在这种情况下，除非干预方式的偏离与试验内容相关，否则本条目判断为“N”或“PN”。不良反应造成的终止干预或换组一般不算偏离既定干预。研究者没有报告偏离是否与试验内容相关时应该回答“NI”，但如果可以判断出很有可能发生与试验内容相关的偏离，答案应该是“PY”	NA/Y/PY/PN/N/NI
**2.4 如果2.3回答“Y/PY/NI”**，这些偏离是否会影响结局?	如果偏离既定干预与常规医疗实践无关，组间的偏离影响结局时需要引起重视	NA/Y/PY/PN/N/NI
**2.5 如果2.4回答了“Y/PY”**，偏离既定干预的情况在各组间是否均衡?	如果偏离既定干预与常规医疗实践无关(2.3回答“Y/PY”)，组间的偏离存在差异时需要引起重视	NA/Y/PY/PN/N/NI
2.6 评价干预效果的分析方法是否恰当?	应用意向性分析(ITT)和修正的意向性分析(mITT)将缺失结局资料的研究对象不予分析可认为是合理的。不恰当的分析方法包括接受干预分析(“as treated”analysis)和遵循研究方案分析(“per-protocol”analysis)。随机化后再分组时，不应对合格研究对象进行排除，但可以排除不合格的研究对象	Y/PY/PN/N/NI
**2.7 如果2.6回答“N/PN/NI”**，无法按照事先随机分组对研究对象进行分析是否可能会对结局产生较大影响?	本问题主要关注没有按照事先随机分组进行分析或者未纳入分析的研究对象的数目是否足以对结果产生重要影响。对于数目多少没有明确的界定：当结局是罕见事件或错分与预后因素有关时，即使少于5%的研究对象被纳人了错误组别进行分析，也有可能对结局产生影响	NA/Y/PY/PN/N/NI
**2. 偏离既定干预的偏倚——(2)干预依从**		
2.1 研究对象是否在试验过程中知晓自己的分组?	如果研究对象知晓了自己的分组，其健康相关行为会有所改变。对研究对象采用盲法(安慰剂)可避免这种健康相关行为的改变	Y/PY/PN/N/NI
2.2 医护人员或试验实施者是否在试验过程中知晓分组?	如果医护人员知晓分组信息，会对干预的实施和管理产生影响。对医护人员设盲(安慰剂)可避免该种影响	Y/PY/PN/N/NI
**2.3 如果2.1或者2.2回答“Y/PY/NI”**时，重要的协同干预措施组间是否均衡?	重要的协同干预措施是指某种干预措施或暴露属于下列情况：①与研究的实施方案不一致；②本研究所用干预正在进行或结束后进行的其他干预；③与研究所用干预相关的其他干预；④与结局预后相关的干预。如果以上类型的协同干预在组间不均衡，则会产生偏倚	NA/Y/PY/PN/N/NI
2.4 是否因未完成既定干预而影响了结局?	“N”“PN”：如果所有或绝大部分研究对象都完成了干预	Y/PY/PN/N/NI
2.5 研究对象是否依从了分配的干预措施?	不依从包括没有完全服从既定干预，终止干预，换到对照组或寻求其他的干预措施。留意随访时有多少研究对象仍然遵循原干预方案，如果不遵循原方案的比例过高，本项选择“N”“PN”。如果干预是一过性的或大部分研究对象都服从原干预，本项选择“Y”	Y/PY/PN/N/NI
**2.6 如果2.3或者2.5回答“N/PN/NI”或2.4回答“Y/PY/NI”**：是否使用了恰当的统计学方法对依从干预的研究对象进行分析?	接受干预分析和遵循研究方案分析为不恰当的方法。如果实际干预与原干预计划有偏，可以通过适当的统计学方法进行处理。包括：①当比较单次干预和标准疗法的差异时，通过工具变量法分析来估计接受既定干预的效应。②逆概率加权法可以用于持续性干预中，以调整由于研究对象不依从原干预造成的偏差。但这些方法有严格的适用条件，如果本项回答“是”或“可能是”时，需要谨慎评估适用条件。如果某一个干预组中所有的研究对象都有很重要的“协同干预”，则这种偏倚无法通过分析避免。如下方法可能并不适用于估计依从干预者的干预效果：ITT分析，接受干预分析，遵循研究方案分析	NA/Y/PY/PN/N/NI
**3. 结局数据缺失偏倚**		
3.1 是否所有或几乎所有随机化分组的研究对象都获得了结局数据?	ITT分析适用于所有随机化分组的研究对象均可纳入分析的情况。注意：填补数据也属于缺失数据，并且在本项中不认为是“结局数据”。“NI”：原文中没有提到结局数据缺失的程度，这种情况一般会存在由于缺失结局数据导致的高偏倚风险。“几乎所有”研究对象是指结局缺失的研究对象人数较少，无论缺失的这部分人是何种结局，均不影响对干预效果的估计方向。对于连续性变量，如果有结局的研究对象占95%(或90%)即可认为是足够的。如果结局是二分类变量，则这个比例与结局事件发生的概率有关。如果发生结局事件的研究对象数目远远大于缺失结局数据的研究对象数目，则只产生较小的偏倚	Y/PY/PN/N/NI
**3.2 如果3.1回答“N/PN/NI”**，是否有证据表明结果不受到缺失的结局数据的影响?	该类证据包括：①使用正确的分析方法来校正偏倚；②敏感性分析的结果表明：在结局缺失情况下的分析结果与真实的结果相比，二者差异局限在可接受的较小范围。但是当我们填补结局变量时，无论是用“末次观察转入”法还是仅基于干预组的多重填补，均不是弥补缺失结局所致的偏倚的有效方法	NA/Y/PY/PN/N
**3.3 如果3.2回答“N/PN”**，结局变量的缺失与结局本身是否相关?	如果失访或退出试验是由于参与者的健康状态所致，则结局变量的缺失很可能是与结局本身相关。如果所有的结局变量缺失均与结局本身无关，则由于缺失所造成的偏倚风险较小(例如测量仪器故障，数据收集被中断等)	NA/Y/PY/PN/N/NI
**3.4 如果3.3回答“Y/PY/NI”**，结局变量的缺失是否可能与结局本身相关?	本问题分如下两种情况：①如果结局缺失有可能与结局本身有关，评价为“可能(some conserns)”；②如果结局变量缺失很可能与结局本身相关，评价为“很可能(high conserns)”以下情况回答“Y”；①两组间结局缺失比例不同的最可能原因是结局变量的缺失与结局本身相关；②结局变量缺失原因的报告提示其与结局本身相关；③两组结局变量缺失的报告原因不同；④研究的实际情况导致结局变量的缺失很可能与结局本身相关。如：从精神分裂症相关研究中退出的最主要的原因是患者的后续症状	NA/Y/PY/PN/N/NI
**4. 结局测量偏倚**		
4.1 结局测量方法是否不恰当?	本题旨在评估数据收集过程中的结局测量是否妥当，而不是为了评价结局指标的选择是否合理。一般来说，对于事先规定好的结局，本题的答案为“N”“PN”。如果结局测方法不恰当则回答“Y”“PY”，如：①目前的测量方法无法将干预效果可靠的测量出来，如结局指标超出测量方法的检出范围；②测量工具可靠性较差	Y/PY/PN/N/NI
4.2 结局的测量或确证方法是否在两组间存在差异?	对两组的结局变量采用的测量方式应该是可比的，包括在可比的时点采用相同的测量方法和具有相同的测量阈值。两组间测量存在差异会造成在结局数据采集过程中的“诊断检出偏倚”，如果干预组的就诊次数更多，可导致该组被识别到发生结局事件的可能性更大	Y/PY/PN/N/NI
**4.3 如果4.1或4.2回答“N/PN/NI”**，结局测量者是否知晓研究对象接受的干预?	如果对干预状态实施了盲法，本题回答“N”。对于研究对象自报结局的研究，结局测量者就是研究对象本人	Y/PY/PN/N/NI
**4.4 如果4.3回答“Y/PY/NI”**，知晓干预措施是否影响了结局变量的测量?	预先知晓干预措施会影响研究对象自报结局(如疼痛等级)，会使得研究者在报告结局时掺入主观判断，影响由干预实施者主观判断的结局指标。如果研究结局不涉及到主观判断则不会影响对结局的测量评价，如结局是死亡、发病等一些客观判断指标时	NA/Y/PY/PN/N/NI
**4.5 如果4.4回答“Y/PY/NI”**，知晓干预措施是否可能影响结局变量的测量?	本问题分如下两种情况：①如果知晓干预措施有可能影响结局测量但是并无证据表明确实影响了结局测量，评价为“可能”；②如果知晓干预措施很可能影响结局测量，评价为“很可能”。当研究对象能够预计干预措施的效果时，无论干预效果被预期为有益的或是有害的，都很有可能影响到结局的测量。例如：在顺势疗法中患者自报症状，或者物理治疗师评价机体功能的康复	NA/Y/PY/PN/N/NI
**5. 结果选择性报告偏倚**		
5.1 试验分析方法是否与数据对分析者揭盲前所制订的研究计划一致?	如果研究者事先制订的研究计划已经有详细报道，则可以将计划的结果测量方法和分析与之前报道的方案进行比较。为了避免研究结果的选择性报告，最终的研究分析方案需要在数据对分析者揭盲之前制订如果在揭盲前更改分析方案，或者能够明确方案更改与结果无关(如仪器损坏导致无法继续收集数据)，此种情况不存在结果选择性报告的偏倚风险	Y/PY/PN/N/NI
5.2 是否进行了多种结局测量(如量表、不同的定义、不同时点)?	是否从以下来源得到多种结果，基于结果进行了选择性报告。为了获得某类结局指标，可能会采用多种测量方法。例如，疼痛程度可能会涉及到使用多个量表评估(直观模拟标度尺或 McGill 疼痛问卷)，或评估多个时点(如治疗后的 3，6，12 周)。如果进行了多种测量，但只是报告了一次或某几次的结果，则会产生很高的选择性报告偏倚风险“Y”“PY”：有明确的证据(如研究方案或统计分析方案)显示对结局进行了多种测量，但只有一个或几个测量结果被全面报告，则认为这些全面报告的结果可能是基于分析结果进行了选择性报报告。选择性报告结果的原因可能是希望结果更有利于发表或更有利于研究假设的验证。例如研究者更愿意证明试验组或干预组有益时，会更倾向于报告试验组有效的结果“N”“PN”：有明确的证据(如研究方案或统计分析方案)表明所有与结局相关的结果测量均采用预定方案：或者结局只有一种可能的测量方式(因此无法选择性报告)，或者在同一个试验的不同报告中，结局测量方法不一致，但研究者对此给出了解释，且这种不一致对结果的性质无影响“NI”：不知道分析方案，或者分析方案的报告不全，且结局指标的测量方式有多种	Y/PY/PN/N/NI
5.3 是否采取了多种分析方式?	某种研究结局可能对应多种分析方法。例如：调整协变量及未调整协变量的模型；最终值 vs 与基线的差值 vs 协方差分析；变量的变换；结局组分的不同定义(如“主要不良反应”)；对连续型变量使用不用的截断值转换为分类变量；不同的协变量调整方式；不同的缺失数据处理方法。不同的分析方法对某种特定结局产生的不同分析结果。如果有多个分析结果，但只报告了一次或几次的结果，则会产生很高的选择性报告偏倚风险“Y”“PY”：有明确的证据(如研究方案或统计分析方案)显示结果进行了多种方式的分析，但只有一个或几个分析结果被全面报告，则认为这些全面报告的结果可能是基于分析结果选择性报告的。选择性报告结果的原因可能是希望结果更有利于发表或更有利于研究假设的验证。例如，研究者想证明干预有益时，会更倾向于报告试验组有效的结果“N”“PN”：有明确的证据(如研究方案或统计分析方案)表明所有与结局相关的结果均与预期分析方法一致：或者结局只有一种可能的分析方式(因此无法选择性报告)，或者在同一个试验中不同的分析方法所得的结果不同，但研究者对此给出了解释，且与结果的自然属性无关“NI”： 不知道分析方案，或者分析方案的报告不全，且结局指标的分析方式有多种	Y/PY/PN/N/NI
