# Supplementary material for: Lipid-lowering effect of combined therapy with high-intensity statins and CETP inhibitors: a Systematic Review and meta-analysis
Source: Front Endocrinol (Lausanne). 2025 May 1;16:1512670. doi: 10.3389/fendo.2025.1512670 (PMC12078159; doi:10.3389/fendo.2025.1512670)
Supplement: Supplementary file 1 [file DataSheet1.zip › Raw Data/Raw Data/5. Risk of bias/ROB-2复件/20190814_RoB_2.0_cribsheet_parallel_trial .pdf]

Revised Cochrane risk-of-bias tool for randomized trials (RoB 2)
SHORT VERSION (CRIBSHEET)

Edited by Julian PT Higgins, Jelena Savović, Matthew J Page, Jonathan AC Sterne
on behalf of the RoB 2 Development Group

Version of 22 August 2019

The development of the RoB 2 tool was supported by the MRC Network of Hubs for Trials Methodology Research (MR/L004933/2- N61), with the support of the host MRC ConDuCT-II Hub (Collaboration and innovation for Difficult and Complex randomised controlled Trials In Invasive procedures - MR/K025643/1), by MRC research grant MR/M025209/1, and by a grant from The Cochrane Collaboration.

This work is licensed under a Creative Commons Attribution-NonCommercial-NoDerivatives 4.0 International License.

Preliminary considerations

Study design

☐ Individually-randomized parallel-group trial
☐ Cluster-randomized parallel-group trial
☐ Individually randomized cross-over (or other matched) trial

For the purposes of this assessment, the interventions being compared are defined as

Experimental: ☐ Comparator: ☐

Specify which outcome is being assessed for risk of bias

Specify the numerical result being assessed. In case of multiple alternative analyses being presented, specify the numeric result (e.g. RR = 1.52 (95% CI 0.83 to 2.77) and/or a reference (e.g. to a table, figure or paragraph) that uniquely defines the result being assessed.

Is the review team's aim for this result…?

☐ to assess the effect of *assignment to intervention* (the 'intention-to-treat' effect)
☐ to assess the effect of *adhering to intervention* (the 'per-protocol' effect)

If the aim is to assess the effect of *adhering to intervention*, select the deviations from intended intervention that should be addressed (at least one must be checked):

☐ occurrence of non-protocol interventions
☐ failures in implementing the intervention that could have affected the outcome
☐ non-adherence to their assigned intervention by trial participants

Which of the following sources were <u>obtained</u> to help inform the risk-of-bias assessment? (tick as many as apply)

☐ Journal article(s)
☐ Trial protocol
☐ Statistical analysis plan (SAP)
☐ Non-commercial trial registry record (e.g. ClinicalTrials.gov record)
☐ Company-owned trial registry record (e.g. GSK Clinical Study Register record)
☐ “Grey literature” (e.g. unpublished thesis)
☐ Conference abstract(s) about the trial
☐ Regulatory document (e.g. Clinical Study Report, Drug Approval Package)
☐ Research ethics application
☐ Grant database summary (e.g. NIH RePORTER or Research Councils UK Gateway to Research)
☐ Personal communication with trialist
☐ Personal communication with the sponsor

Domain 1: Risk of bias arising from the randomization process

Signalling questions	Elaboration	Response options
**1.1 Was the allocation sequence random?**	Answer 'Yes' if a random component was used in the sequence generation process. Examples include computer-generated random numbers; reference to a random number table; coin tossing; shuffling cards or envelopes; throwing dice; or drawing lots. Minimization is generally implemented with a random element (at least when the scores are equal), so an allocation sequence that is generated using minimization should generally be considered to be random.<br><br>Answer 'No' if no random element was used in generating the allocation sequence or the sequence is predictable. Examples include alternation; methods based on dates (of birth or admission); patient record numbers; allocation decisions made by clinicians or participants; allocation based on the availability of the intervention; or any other systematic or haphazard method.<br><br>Answer 'No information' if the only information about randomization methods is a statement that the study is randomized.<br><br>In some situations a judgement may be made to answer 'Probably no' or 'Probably yes'. For example, , in the context of a large trial run by an experienced clinical trials unit, absence of specific information about generation of the randomization sequence, in a paper published in a journal with rigorously enforced word count limits, is likely to result in a response of 'Probably yes' rather than 'No information'. Alternatively, if other (contemporary) trials by the same investigator team have clearly used non-random sequences, it might be reasonable to assume that the current study was done using similar methods.	Y/PY/PN/N/NI
**1.2 Was the allocation sequence concealed until participants were enrolled and assigned to interventions?**	Answer 'Yes' if the trial used any form of remote or centrally administered method to allocate interventions to participants, where the process of allocation is controlled by an external unit or organization, independent of the enrolment personnel (e.g. independent central pharmacy, telephone or internet-based randomization service providers).<br><br>Answer 'Yes' if envelopes or drug containers were used appropriately. Envelopes should be opaque, sequentially numbered, sealed with a tamper-proof seal and opened only after the envelope has been irreversibly assigned to the participant. Drug containers should be sequentially numbered and of identical appearance, and dispensed or administered only after they have been irreversibly assigned to the participant. This level of detail is rarely provided in reports, and a judgement may be required to justify an answer of 'Probably yes' or 'Probably no'.<br><br>Answer 'No' if there is reason to suspect that the enrolling investigator or the participant had knowledge of the forthcoming allocation.	Y/PY/PN/N/NI

**1.3 Did baseline differences between intervention groups suggest a problem with the randomization process?**	*Note that differences that are compatible with chance do not lead to a risk of bias. A small number of differences identified as 'statistically significant' at the conventional 0.05 threshold should usually be considered to be compatible with chance.*<br><br>Answer 'No' if no imbalances are apparent or if any observed imbalances are compatible with chance.<br><br>Answer 'Yes' if there are imbalances that indicate problems with the randomization process, including:<br>(1) substantial differences between intervention group sizes, compared with the intended allocation ratio;<br>or<br>(2) a substantial excess in statistically significant differences in baseline characteristics between intervention groups, beyond that expected by chance; or<br>(3) imbalance in one or more key prognostic factors, or baseline measures of outcome variables, that is very unlikely to be due to chance and for which the between-group difference is big enough to result in bias in the intervention effect estimate.<br><br>Also answer 'Yes' if there are other reasons to suspect that the randomization process was problematic:<br>(4) excessive similarity in baseline characteristics that is not compatible with chance.<br><br>Answer 'No information' when there is no *useful* baseline information available (e.g. abstracts, or studies that reported only baseline characteristics of participants in the final analysis).<br><br>The answer to this question should not influence answers to questions 1.1 or 1.2. For example, if the trial has large baseline imbalances, but authors report adequate randomization methods, questions 1.1 and 1.2 should still be answered on the basis of the reported adequate methods, and any concerns about the imbalance should be raised in the answer to the question 1.3 and reflected in the domain-level risk-of-bias judgement.<br><br>Trialists may undertake analyses that attempt to deal with flawed randomization by controlling for imbalances in prognostic factors at baseline. To remove the risk of bias caused by problems in the randomization process, it would be necessary to know, and measure, all the prognostic factors that were imbalanced at baseline. It is unlikely that all important prognostic factors are known and measured, so such analyses will at best reduce the risk of bias. If review authors wish to assess the risk of bias in a trial that controlled for baseline imbalances in order to mitigate failures of randomization, the study should be assessed using the ROBINS-I tool.	Y/PY/<u>PN/N</u>/NI
**Risk-of-bias judgement**	See algorithm.	Low / High / Some concerns

Optional: What is the predicted direction of bias arising from the randomization process?	If the likely direction of bias can be predicted, it is helpful to state this. The direction might be characterized either as being towards (or away from) the null, or as being in favour of one of the interventions.	NA / Favours experimental / Favours comparator / Towards null /Away from null / Unpredictable

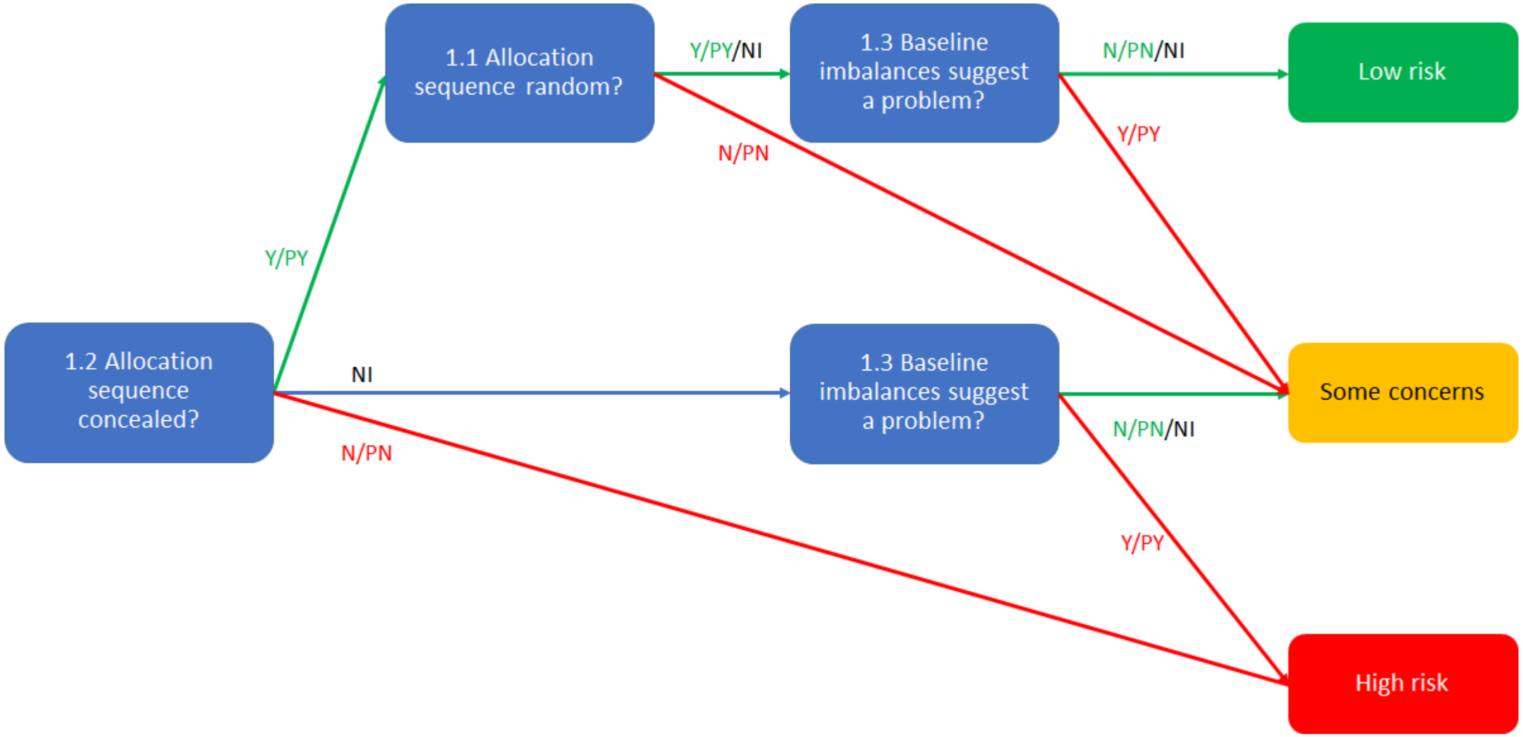

Algorithm for suggested judgement of risk of bias arising from the randomization process

Domain 2: Risk of bias due to deviations from the intended interventions (*effect of assignment to intervention*)

Signalling questions	Elaboration	Response options
**2.1. Were participants aware of their assigned intervention during the trial?**	If participants are aware of their assigned intervention it is more likely that health-related behaviours will differ between the intervention groups. Blinding participants, most commonly through use of a placebo or sham intervention, may prevent such differences. If participants experienced side effects or toxicities that they knew to be specific to one of the interventions, answer this question 'Yes' or 'Probably yes'.	Y/PY/PN/N/NI
**2.2. Were carers and people delivering the interventions aware of participants' assigned intervention during the trial?**	If carers or people delivering the interventions are aware of the assigned intervention then its implementation, or administration of non-protocol interventions, may differ between the intervention groups. Blinding may prevent such differences. If participants experienced side effects or toxicities that carers or people delivering the interventions knew to be specific to one of the interventions, answer question 'Yes' or 'Probably yes'. If randomized allocation was not concealed, then it is likely that carers and people delivering the interventions were aware of participants' assigned intervention during the trial.	Y/PY/PN/N/NI

**2.3. If Y/PY/NI to 2.1 or 2.2: Were there deviations from the intended intervention that arose because of the trial context?**	For the effect of assignment to intervention, this domain assesses problems that arise when changes from assigned intervention that are inconsistent with the trial protocol arose because of the trial context. We use the term **trial context** to refer to effects of recruitment and engagement activities on trial participants and when trial personnel (carers or people delivering the interventions) undermine the implementation of the trial protocol in ways that would not happen outside the trial. For example, the process of securing informed consent may lead participants subsequently assigned to the comparator group to feel unlucky and therefore seek the experimental intervention, or other interventions that improve their prognosis.<br><br>Answer 'Yes' or 'Probably yes' **only** if there is evidence, or strong reason to believe, that the trial context led to failure to implement the protocol interventions or to implementation of interventions not allowed by the protocol.<br><br>Answer 'No' or 'Probably no' if there were changes from assigned intervention that are inconsistent with the trial protocol, such as non-adherence to intervention, but these are consistent with what could occur outside the trial context.<br><br>Answer 'No' or 'Probably no' for changes to intervention that are consistent with the trial protocol, for example cessation of a drug intervention because of acute toxicity or use of additional interventions whose aim is to treat consequences of one of the intended interventions.<br><br>If blinding is compromised because participants report side effects or toxicities that are specific to one of the interventions, answer 'Yes' or 'Probably yes' only if there were changes from assigned intervention that are inconsistent with the trial protocol and arose because of the trial context.<br><br>The answer 'No information' may be appropriate, because trialists do not always report whether deviations arose because of the trial context.	NA/Y/PY/PN/N/NI
**2.4 If Y/PY to 2.3: Were these deviations likely to have affected the outcome?**	Changes from assigned intervention that are inconsistent with the trial protocol and arose because of the trial context will impact on the intervention effect estimate if they affect the outcome, but not otherwise.	NA/Y/PY/PN/N/NI

**2.5. If Y/PY/NI to 2.4: Were these deviations from intended intervention balanced between groups?**	Changes from assigned intervention that are inconsistent with the trial protocol and arose because of the trial context are more likely to impact on the intervention effect estimate if they are not balanced between the intervention groups.	NA/Y/PY/PN/N/NI
**2.6 Was an appropriate analysis used to estimate the effect of assignment to intervention?**	Both intention-to-treat (ITT) analyses and modified intention-to-treat (mITT) analyses excluding participants with missing outcome data should be considered appropriate. Both naïve 'per-protocol' analyses (excluding trial participants who did not receive their assigned intervention) and 'as treated' analyses (in which trial participants are grouped according to the intervention that they received, rather than according to their assigned intervention) should be considered inappropriate. Analyses excluding eligible trial participants post-randomization should also be considered inappropriate, but post-randomization exclusions of ineligible participants (when eligibility was not confirmed until after randomization, and could not have been influenced by intervention group assignment) can be considered appropriate.	Y/PY/PN/N/NI
**2.7 If N/PN/NI to 2.6: Was there potential for a substantial impact (on the result) of the failure to analyse participants in the group to which they were randomized?**	This question addresses whether the number of participants who were analysed in the wrong intervention group, or excluded from the analysis, was sufficient that there could have been a substantial impact on the result. It is not possible to specify a precise rule: there may be potential for substantial impact even if fewer than 5% of participants were analysed in the wrong group or excluded, if the outcome is rare or if exclusions are strongly related to prognostic factors.	NA/Y/PY/PN/N/NI
**Risk-of-bias judgement**	See algorithm.	Low / High / Some concerns
Optional: What is the predicted direction of bias due to deviations from intended interventions?	If the likely direction of bias can be predicted, it is helpful to state this. The direction might be characterized either as being towards (or away from) the null, or as being in favour of one of the interventions.	NA / Favours experimental / Favours comparator / Towards null /Away from null / Unpredictable

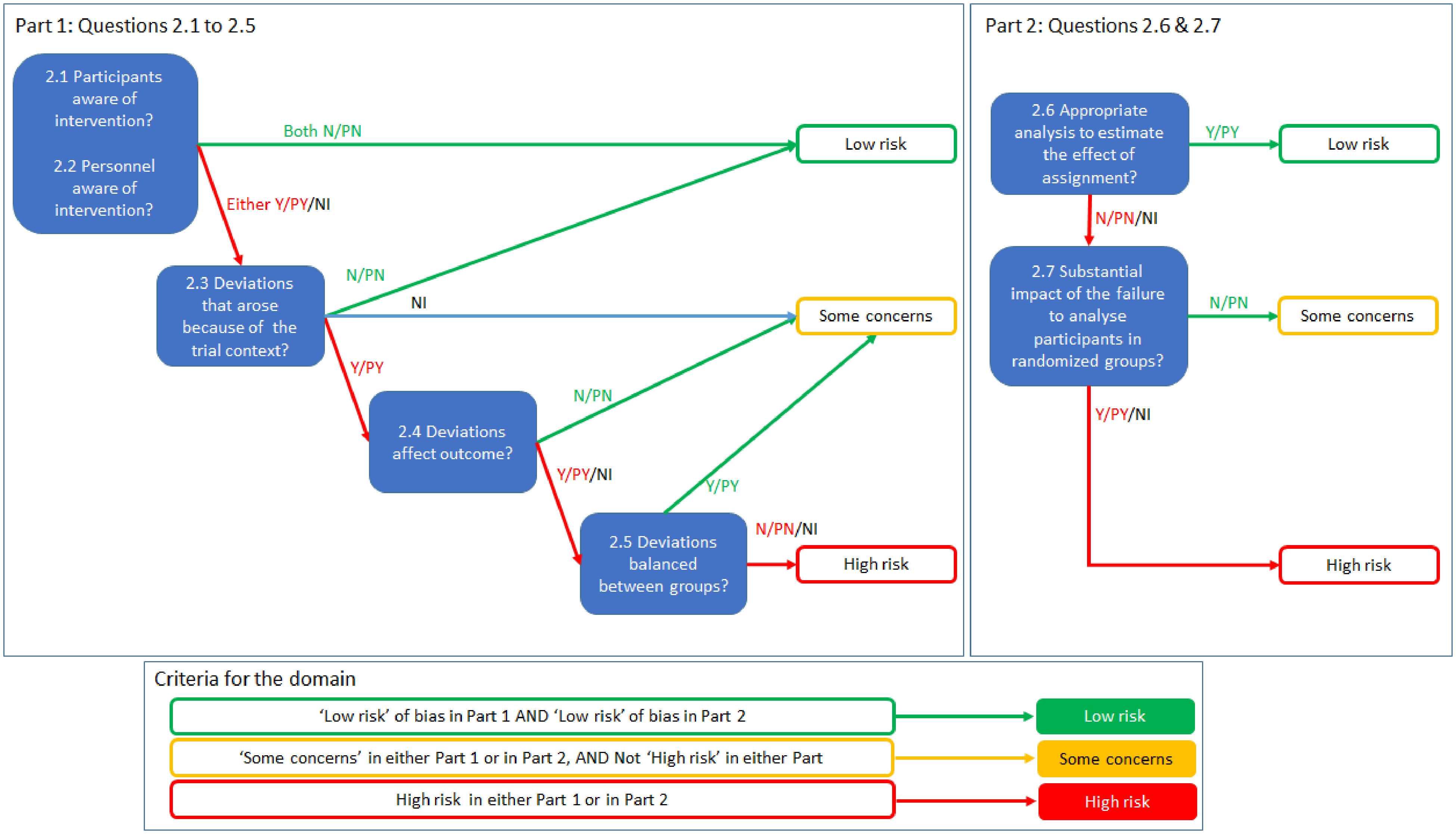

Algorithm for suggested judgement of risk of bias due to deviations from the intended interventions (effect of assignment to intervention)

Domain 2: Risk of bias due to deviations from the intended interventions (*effect of adhering to intervention*)

Signalling questions	Elaboration	Response options
**2.1. Were participants aware of their assigned intervention during the trial?**	If participants are aware of their assigned intervention it is more likely that health-related behaviours will differ between the intervention groups. Blinding participants, most commonly through use of a placebo or sham intervention, may prevent such differences. If participants experienced side effects or toxicities that they knew to be specific to one of the interventions, answer this question 'Yes' or 'Probably yes'.	Y/PY/PN/N/NI
**2.2. Were carers and people delivering the interventions aware of participants' assigned intervention during the trial?**	If carers or people delivering the interventions are aware of the assigned intervention then its implementation, or administration of non-protocol interventions, may differ between the intervention groups. Blinding may prevent such differences. If participants experienced side effects or toxicities that carers or people delivering the interventions knew to be specific to one of the interventions, answer 'Yes' or 'Probably yes'. If randomized allocation was not concealed, then it is likely that carers and people delivering the interventions were aware of participants' assigned intervention during the trial.	Y/PY/PN/N/NI
**2.3. [If applicable:] If Y/PY/NI to 2.1 or 2.2: Were important non-protocol interventions balanced across intervention groups?**	This question is asked only if the preliminary considerations specify that the assessment will address imbalance of important non-protocol interventions between intervention groups. Important non-protocol interventions are the additional interventions or exposures that: (1) are inconsistent with the trial protocol; (2) trial participants might receive with or after starting their assigned intervention; and (3) are prognostic for the outcome. Risk of bias will be higher if there is imbalance in such interventions between the intervention groups.	NA/Y/PY/PN/N/NI
**2.4. [If applicable:] Were there failures in implementing the intervention that could have affected the outcome?**	This question is asked only if the preliminary considerations specify that the assessment will address failures in implementing the intervention that could have affected the outcome. Risk of bias will be higher if the intervention was not implemented as intended by, for example, the health care professionals delivering care. Answer 'No' or 'Probably no' if implementation of the intervention was successful for most participants.	NA/Y/PY/PN/N/NI
**2.5. [If applicable:] Was there non-adherence to the assigned intervention regimen that could have affected participants' outcomes?**	This question is asked only if the preliminary considerations specify that the assessment will address non-adherence that could have affected participants' outcomes. Non-adherence includes imperfect compliance with a sustained intervention, cessation of intervention, crossovers to the comparator intervention and switches to another active intervention. Consider available information on the proportion of study participants who continued with their assigned intervention throughout follow up, and answer 'Yes' or 'Probably yes' if the proportion who did not adhere is high enough to raise concerns. Answer 'No' for studies of interventions that are administered once, so that imperfect adherence is not possible, and all or most participants received the assigned intervention.	NA/Y/PY/PN/N/NI

**2.6. If N/PN/NI to 2.3, or Y/PY/NI to 2.4 or 2.5: Was an appropriate analysis used to estimate the effect of adhering to the intervention?**	Both ' naïve 'per-protocol' analyses (excluding trial participants who did not receive their allocated intervention) and 'as treated' analyses (comparing trial participants according to the intervention they actually received) will usually be inappropriate for estimating the effect of adhering to intervention (the 'per-protocol' effect). However, it is possible to use data from a randomized trial to derive an unbiased estimate of the effect of adhering to intervention. Examples of appropriate methods include: (1) instrumental variable analyses to estimate the effect of receiving the assigned intervention in trials in which a single intervention, administered only at baseline and with all-or-nothing adherence, is compared with standard care; and (2) inverse probability weighting to adjust for censoring of participants who cease adherence to their assigned intervention, in trials of sustained treatment strategies. These methods depend on strong assumptions, which should be appropriate and justified if the answer to this question is 'Yes' or 'Probably yes'. It is possible that a paper reports an analysis based on such methods without reporting information on the deviations from intended intervention, but it would be hard to judge such an analysis to be appropriate in the absence of such information.<br><br>If an important non-protocol intervention was administered to all participants in one intervention group, adjustments cannot be made to overcome this.<br><br>Some examples of analysis strategies that would not be appropriate to estimate the effect of adhering to intervention are (i) 'Intention to treat (ITT) analysis', (ii) 'per protocol analysis', (iii) 'as-treated analysis', (iv) 'analysis by treatment received'.	NA/Y/PY/PN/N/NI
**Risk-of-bias judgement**	See algorithm.	Low / High / Some concerns
Optional: What is the predicted direction of bias due to deviations from intended interventions?	If the likely direction of bias can be predicted, it is helpful to state this. The direction might be characterized either as being towards (or away from) the null, or as being in favour of one of the interventions.	NA / Favours experimental / Favours comparator / Towards null /Away from null / Unpredictable

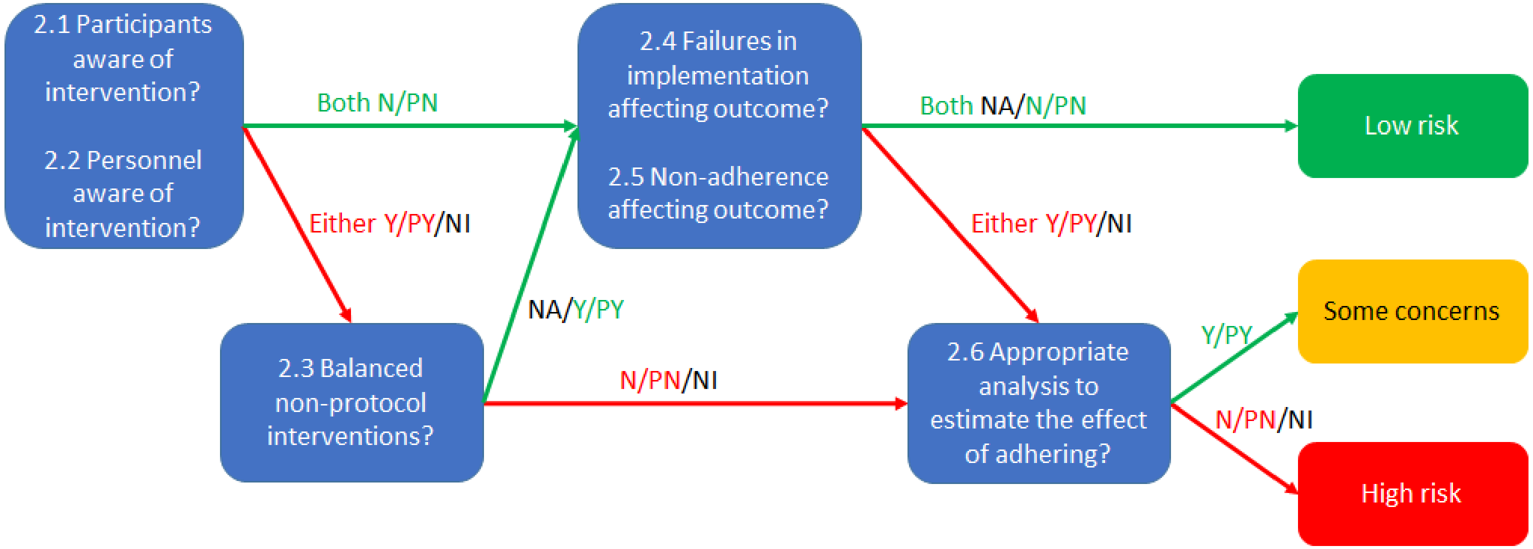

Algorithm for suggested judgement of risk of bias due to deviations from the intended interventions (effect of adhering to intervention)

Domain 3: Risk of bias due to missing outcome data

Signalling questions	Elaboration	Response options
**3.1 Were data for this outcome available for all, or nearly all, participants randomized?**	The appropriate study population for an analysis of the intention to treat effect is all randomized participants.<br>“Nearly all” should be interpreted as that the number of participants with missing outcome data is sufficiently small that their outcomes, whatever they were, could have made no important difference to the estimated effect of intervention.<br>For continuous outcomes, availability of data from 95% of the participants will often be sufficient. For dichotomous outcomes, the proportion required is directly linked to the risk of the event. If the observed number of events is much greater than the number of participants with missing outcome data, the bias would necessarily be small.<br>Only answer ‘No information’ if the trial report provides no information about the extent of missing outcome data. This situation will usually lead to a judgement that there is a high risk of bias due to missing outcome data.<br>Note that imputed data should be regarded as missing data, and not considered as ‘outcome data’ in the context of this question.	Y/PY/PN/N/NI
**3.2 If N/PN/NI to 3.1: Is there evidence that the result was not biased by missing outcome data?**	Evidence that the result was not biased by missing outcome data may come from: (1) analysis methods that correct for bias; or (2) sensitivity analyses showing that results are little changed under a range of plausible assumptions about the relationship between missingness in the outcome and its true value. However, imputing the outcome variable, either through methods such as ‘last-observation-carried-forward’ or via multiple imputation based only on intervention group, should not be assumed to correct for bias due to missing outcome data.	NA/Y/PY/PN/N
**3.3 If N/PN to 3.2: Could missingness in the outcome depend on its true value?**	If loss to follow up, or withdrawal from the study, could be related to participants’ health status, then it is possible that missingness in the outcome was influenced by its true value. However, if all missing outcome data occurred for documented reasons that are unrelated to the outcome then the risk of bias due to missing outcome data will be low (for example, failure of a measuring device or interruptions to routine data collection).<br>In time-to-event analyses, participants censored during trial follow-up, for example because they withdrew from the study, should be regarded as having missing outcome data, even though some of their follow up is included in the analysis. Note that such participants may be shown as included in analyses in CONSORT flow diagrams.	NA/Y/PY/PN/N/NI

**3.4 If Y/PY/NI to 3.3: Is it likely that missingness in the outcome depended on its true value?**	This question distinguishes between situations in which (i) missingness in the outcome could depend on its true value (assessed as 'Some concerns') from those in which (ii) it is likely that missingness in the outcome depended on its true value (assessed as 'High risk of bias'). Five reasons for answering 'Yes' are:<br>1. Differences between intervention groups in the proportions of missing outcome data. If there is a difference between the effects of the experimental and comparator interventions on the outcome, and the missingness in the outcome is influenced by its true value, then the proportions of missing outcome data are likely to differ between intervention groups. Such a difference suggests a risk of bias due to missing outcome data, because the trial result will be sensitive to missingness in the outcome being related to its true value. For time-to-event-data, the analogue is that rates of censoring (loss to follow-up) differ between the intervention groups.<br>2. Reported reasons for missing outcome data provide evidence that missingness in the outcome depends on its true value;<br>3. Reported reasons for missing outcome data differ between the intervention groups;<br>4. The circumstances of the trial make it likely that missingness in the outcome depends on its true value. For example, in trials of interventions to treat schizophrenia it is widely understood that continuing symptoms make drop out more likely.<br>5. In time-to-event analyses, participants' follow up is censored when they stop or change their assigned intervention, for example because of drug toxicity or, in cancer trials, when participants switch to second-line chemotherapy.<br>Answer 'No' if the analysis accounted for participant characteristics that are likely to explain the relationship between missingness in the outcome and its true value.	NA/Y/PY/PN/N/NI
**Risk-of-bias judgement**	See algorithm.	Low / High / Some concerns
Optional: What is the predicted direction of bias due to missing outcome data?	If the likely direction of bias can be predicted, it is helpful to state this. The direction might be characterized either as being towards (or away from) the null, or as being in favour of one of the interventions.	NA / Favours experimental / Favours comparator / Towards null /Away from null / Unpredictable

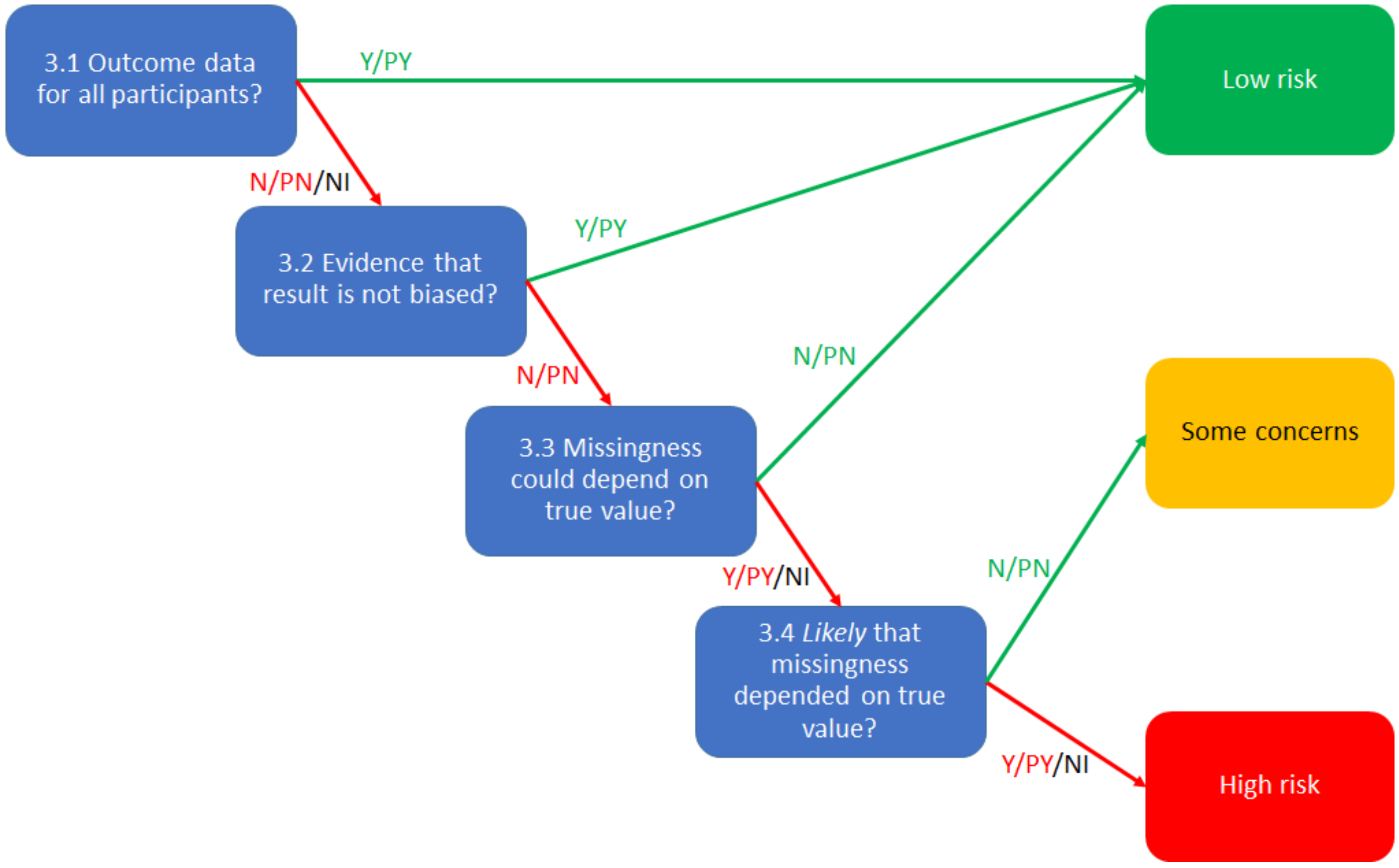

Algorithm for suggested judgement of risk of bias due to missing outcome data

Domain 4: Risk of bias in measurement of the outcome

Signalling questions	Elaboration	Response options
**4.1 Was the method of measuring the outcome inappropriate?**	This question aims to identify methods of outcome measurement (data collection) that are unsuitable for the outcome they are intended to evaluate. The question *does not* aim to assess whether the choice of outcome being evaluated was sensible (e.g. because it is a surrogate or proxy for the main outcome of interest). In most circumstances, for pre-specified outcomes, the answer to this question will be 'No' or 'Probably no'.<br><br>Answer 'Yes' or 'Probably yes' if the method of measuring the outcome is inappropriate, for example because:<br><br>(1) it is unlikely to be sensitive to plausible intervention effects (e.g. important ranges of outcome values fall outside levels that are detectable using the measurement method); or<br>(2) the measurement instrument has been demonstrated to have poor validity.	Y/PY/PN/N/NI
**4.2 Could measurement or ascertainment of the outcome have differed between intervention groups?**	Comparable methods of outcome measurement (data collection) involve the same measurement methods and thresholds, used at comparable time points. Differences between intervention groups may arise because of 'diagnostic detection bias' in the context of passive collection of outcome data, or if an intervention involves additional visits to a healthcare provider, leading to additional opportunities for outcome events to be identified.	Y/PY/PN/N/NI
**4.3 If N/PN/NI to 4.1 and 4.2: Were outcome assessors aware of the intervention received by study participants?**	Answer 'No' if outcome assessors were blinded to intervention status. For participant-reported outcomes, the outcome assessor is the study participant.	NA/Y/PY/PN/N/NI
**4.4 If Y/PY/NI to 4.3: Could assessment of the outcome have been influenced by knowledge of intervention received?**	Knowledge of the assigned intervention could influence participant-reported outcomes (such as level of pain), observer-reported outcomes involving some judgement, and intervention provider decision outcomes. They are unlikely to influence observer-reported outcomes that do not involve judgement, for example all-cause mortality.	NA/Y/PY/PN/N/NI

**4.5 If Y/PY/NI to 4.4: Is it likely that assessment of the outcome was influenced by knowledge of intervention received?**	This question distinguishes between situations in which (i) knowledge of intervention status could have influenced outcome assessment but there is no reason to believe that it did (assessed as 'Some concerns') from those in which (ii) knowledge of intervention status was likely to influence outcome assessment (assessed as 'High'). When there are strong levels of belief in either beneficial or harmful effects of the intervention, it is more likely that the outcome was influenced by knowledge of the intervention received. Examples may include patient-reported symptoms in trials of homeopathy, or assessments of recovery of function by a physiotherapist who delivered the intervention.	NA/Y/PY/PN/N/NI
**Risk-of-bias judgement**	See algorithm.	Low / High / Some concerns
Optional: What is the predicted direction of bias in measurement of the outcome?	If the likely direction of bias can be predicted, it is helpful to state this. The direction might be characterized either as being towards (or away from) the null, or as being in favour of one of the interventions.	NA / Favours experimental / Favours comparator / Towards null /Away from null / Unpredictable

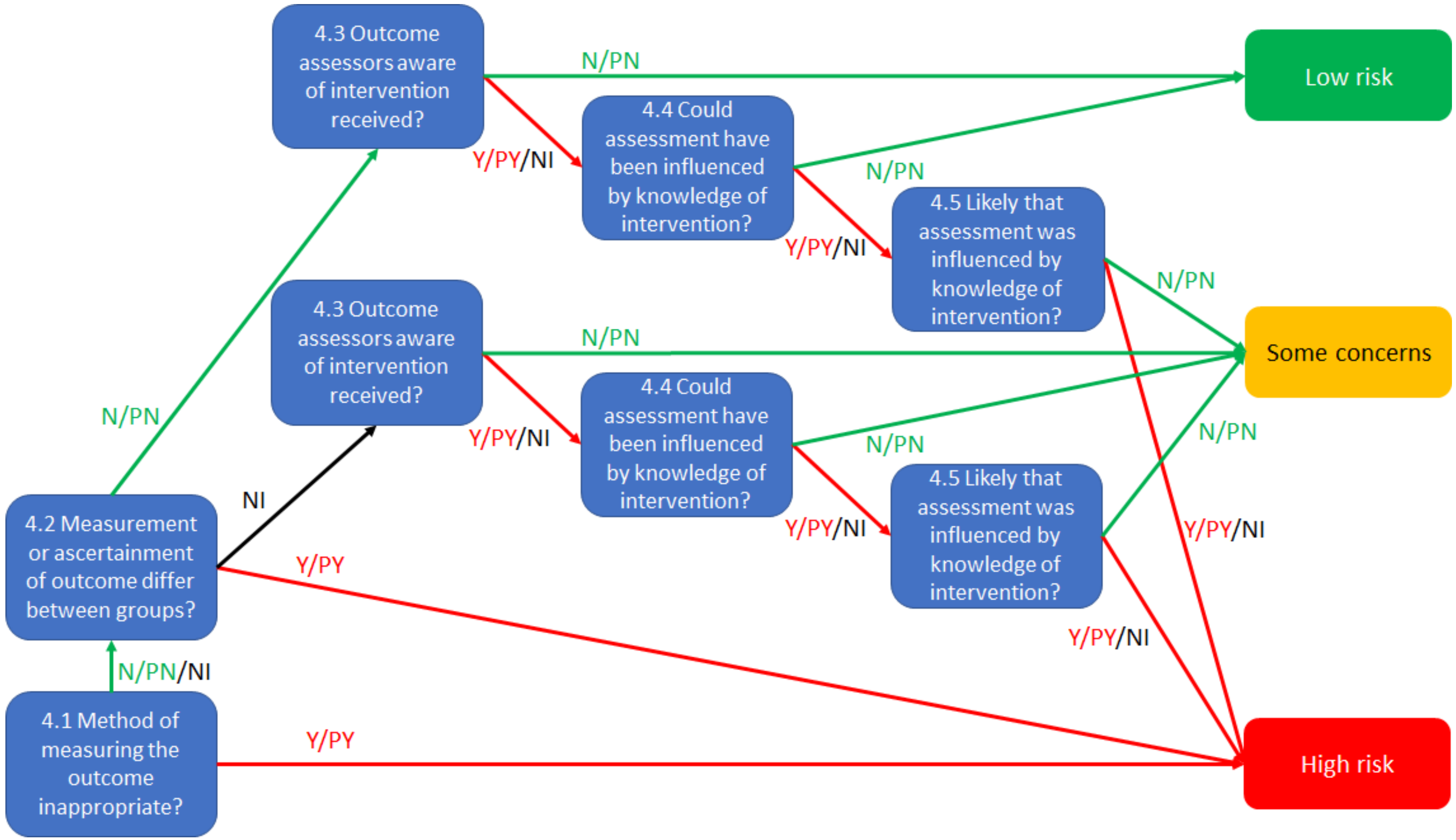

Algorithm for suggested judgement of risk of bias in measurement of the outcome

Domain 5: Risk of bias in selection of the reported result

Signalling questions	Elaboration	Response options
**5.1 Were the data that produced this result analysed in accordance with a pre-specified analysis plan that was finalized before unblinded outcome data were available for analysis?**	If the researchers' pre-specified intentions are available in sufficient detail, then planned outcome measurements and analyses can be compared with those presented in the published report(s). To avoid the possibility of selection of the reported result, finalization of the analysis intentions must precede availability of unblinded outcome data to the trial investigators.<br>Changes to analysis plans that were made before unblinded outcome data were available, or that were clearly unrelated to the results (e.g. due to a broken machine making data collection impossible) do not raise concerns about bias in selection of the reported result.	Y/PY/PN/N/NI
**Is the numerical result being assessed likely to have been selected, on the basis of the results, from...**		
**5.2. ... multiple eligible outcome measurements (e.g. scales, definitions, time points) within the outcome domain?**	A particular outcome domain (i.e. a true state or endpoint of interest) may be **measured** in multiple ways. For example, the domain pain may be measured using multiple scales (e.g. a visual analogue scale and the McGill Pain Questionnaire), each at multiple time points (e.g. 3, 6 and 12 weeks post-treatment). If multiple measurements were made, but only one or a subset is reported on the basis of the results (e.g. statistical significance), there is a high risk of bias in the fully reported result. Attention should be restricted to outcome measurements that are eligible for consideration by the RoB 2 tool user. For example, if only a result using a specific measurement scale is eligible for inclusion in a meta-analysis (e.g. Hamilton Depression Rating Scale), and this is reported by the trial, then there would not be an issue of selection even if this result was reported (on the basis of the results) in preference to the result from a different measurement scale (e.g. Beck Depression Inventory).<br>Answer 'Yes' or 'Probably yes' if:<br>There is clear evidence (usually through examination of a trial protocol or statistical analysis plan) that a domain was measured in multiple eligible ways, but data for only one or a subset of measures is fully reported (without justification), and the fully reported result is likely to have been selected on the basis of the results. Selection on the basis of the results can arise from a desire for findings to be newsworthy, sufficiently noteworthy to merit publication, or to confirm a prior hypothesis. For example, trialists who have a preconception, or vested interest in showing, that an	Y/PY/PN/N/NI

	experimental intervention is beneficial may be inclined to report outcome measurements selectively that are favourable to the experimental intervention.<br>Answer ‘No’ or ‘Probably no’ if:<br>There is clear evidence (usually through examination of a trial protocol or statistical analysis plan) that all eligible reported results for the outcome domain correspond to all intended outcome measurements.<br>or<br>There is only one possible way in which the outcome domain can be measured (hence there is no opportunity to select from multiple measures).<br>or<br>Outcome measurements are inconsistent across different reports on the same trial, but the trialists have provided the reason for the inconsistency and it is not related to the nature of the results.<br>Answer ‘No information’ if:<br>Analysis intentions are not available, or the analysis intentions are not reported in sufficient detail to enable an assessment, and there is more than one way in which the outcome domain could have been measured.	
**5.3 ... multiple eligible analyses of the data?**	A particular outcome measurement may be analysed in multiple ways. Examples include: unadjusted and adjusted models; final value vs change from baseline vs analysis of covariance; transformations of variables; different definitions of composite outcomes (e.g. ‘major adverse event’); conversion of continuously scaled outcome to categorical data with different cut-points; different sets of covariates for adjustment; and different strategies for dealing with missing data. Application of multiple methods generates multiple effect estimates for a specific outcome measurement. If multiple estimates are generated but only one or a subset is reported on the basis of the results (e.g. statistical significance), there is a high risk of bias in the fully reported result. Attention should be restricted to analyses that are eligible for consideration by the RoB 2 tool user. For example, if only the result from an analysis of post-intervention values is eligible for inclusion in a meta-analysis (e.g. at 12 weeks after randomization), and this is reported by the trial, then there would not be an issue of selection even if this result was reported (on the basis of the results) in preference to the result from an analysis of changes from baseline.<br>Answer ‘Yes’ or ‘Probably yes’ if:	Y/PY/PN/N/NI

	There is clear evidence (usually through examination of a trial protocol or statistical analysis plan) that a measurement was analysed in multiple eligible ways, but data for only one or a subset of analyses is fully reported (without justification), and the fully reported result is likely to have been selected on the basis of the results. Selection on the basis of the results arises from a desire for findings to be newsworthy, sufficiently noteworthy to merit publication, or to confirm a prior hypothesis. For example, trialists who have a preconception or vested interest in showing that an experimental intervention is beneficial may be inclined to selectively report analyses that are favourable to the experimental intervention.<br>Answer ‘No’ or ‘Probably no’ if:<br>There is clear evidence (usually through examination of a trial protocol or statistical analysis plan) that all eligible reported results for the outcome measurement correspond to all intended analyses.<br>or<br>There is only one possible way in which the outcome measurement can be analysed (hence there is no opportunity to select from multiple analyses).<br>or<br>Analyses are inconsistent across different reports on the same trial, but the trialists have provided the reason for the inconsistency and it is not related to the nature of the results.<br>Answer ‘No information’ if:<br>Analysis intentions are not available, or the analysis intentions are not reported in sufficient detail to enable an assessment, and there is more than one way in which the outcome measurement could have been analysed.	
**Risk-of-bias judgement**	See algorithm.	Low / High / Some concerns
Optional: What is the predicted direction of bias due to selection of the reported result?	If the likely direction of bias can be predicted, it is helpful to state this. The direction might be characterized either as being towards (or away from) the null, or as being in favour of one of the interventions.	NA / Favours experimental / Favours comparator / Towards null /Away from null / Unpredictable

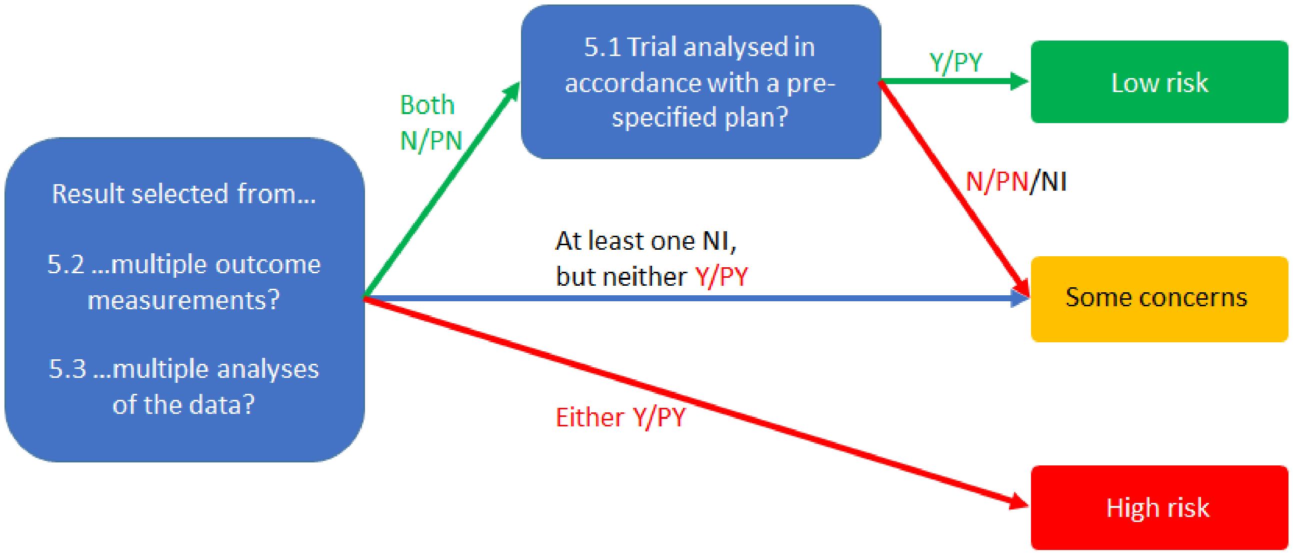

Algorithm for suggested judgement of risk of bias in selection of the reported result

Overall risk of bias

Risk-of-bias judgement		Low / High / Some concerns
Optional: What is the overall predicted direction of bias for this outcome?		Favours experimental / Favours comparator / Towards null /Away from null / Unpredictable / NA

Overall risk-of-bias judgement	Criteria
Low risk of bias	The study is judged to be at **low risk of bias for all domains** for this result.
Some concerns	The study is judged to raise **some concerns** in at least one domain for this result, but not to be at high risk of bias for any domain.
High risk of bias	The study is judged to be at **high risk of bias** in at least one domain for this result.<br>Or<br>The study is judged to have **some concerns** for **multiple domains** in a way that substantially lowers confidence in the result.

The development of the RoB 2 tool was supported by the MRC Network of Hubs for Trials Methodology Research (MR/L004933/2- N61), with the support of the host MRC ConDuCT-II Hub (Collaboration and innovation for Difficult and Complex randomised controlled Trials In Invasive procedures - MR/K025643/1), by MRC research grant MR/M025209/1, and by a grant from The Cochrane Collaboration.

This work is licensed under a Creative Commons Attribution-NonCommercial-NoDerivatives 4.0 International License.
