# Supplementary material for: Lipid-lowering effect of combined therapy with high-intensity statins and CETP inhibitors: a Systematic Review and meta-analysis
Source: Front Endocrinol (Lausanne). 2025 May 1;16:1512670. doi: 10.3389/fendo.2025.1512670 (PMC12078159; doi:10.3389/fendo.2025.1512670)
Supplement: Supplementary file 1 [file DataSheet1.zip › Raw Data/Raw Data/flow chart.pptx]

## Slide 1
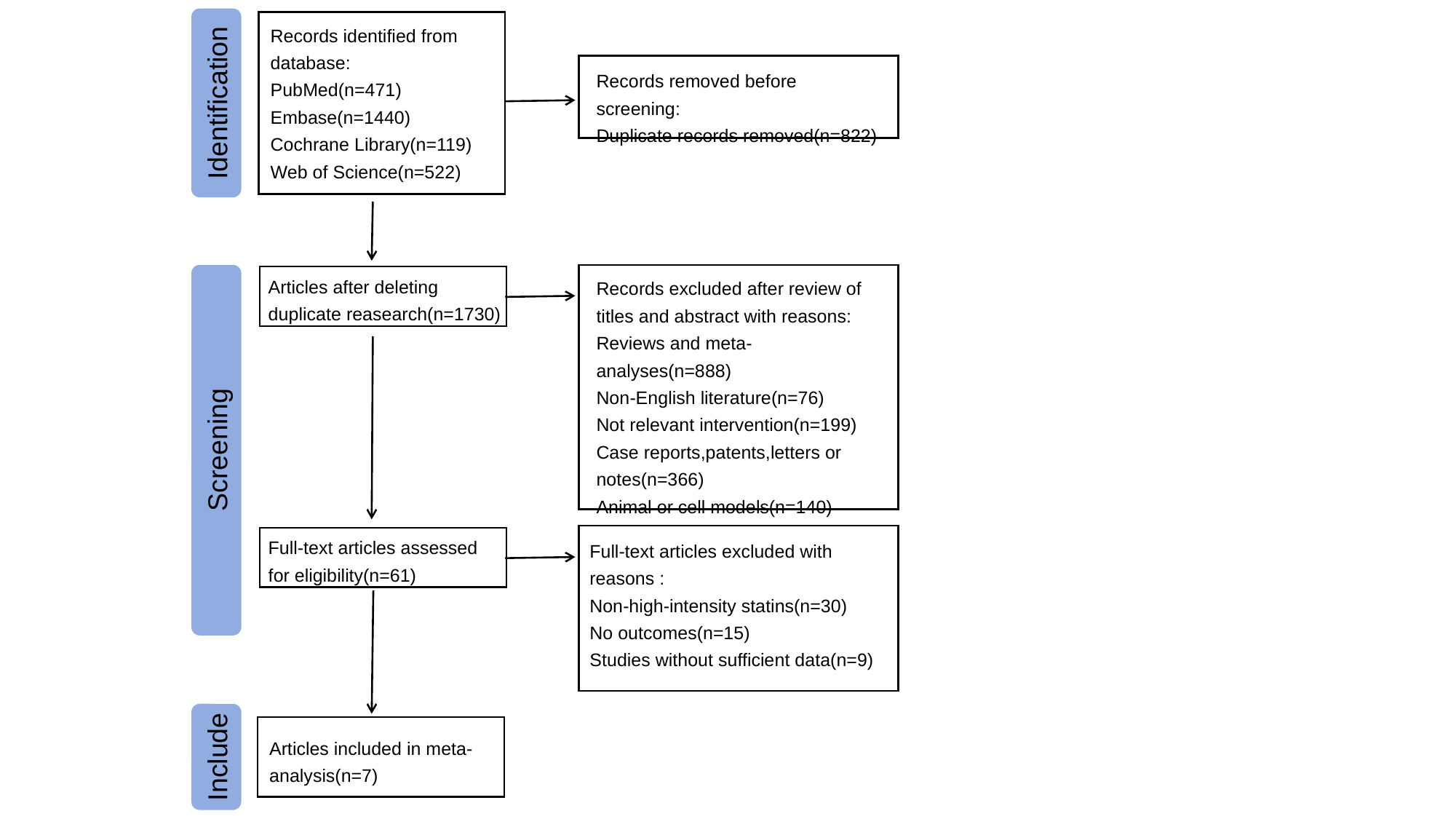

Identification
Records identified from database:
PubMed(n=471)
Embase(n=1440)
Cochrane Library(n=119)
Web of Science(n=522)
Records removed before screening:
Duplicate records removed(n=822)
Articles after deleting
duplicate reasearch(n=1730)
Screening
Records excluded after review of titles and abstract with reasons:
Reviews and meta-analyses(n=888)
Non-English literature(n=76)
Not relevant intervention(n=199)
Case reports,patents,letters or notes(n=366)
Animal or cell models(n=140)
Full-text articles assessed for eligibility(n=61)
Full-text articles excluded with reasons :
Non-high-intensity statins(n=30)
No outcomes(n=15)
Studies without sufficient data(n=9)
Include
Articles included in meta-analysis(n=7)
