# Supplementary material for: Lipid-lowering effect of combined therapy with high-intensity statins and CETP inhibitors: a Systematic Review and meta-analysis
Source: Front Endocrinol (Lausanne). 2025 May 1;16:1512670. doi: 10.3389/fendo.2025.1512670 (PMC12078159; doi:10.3389/fendo.2025.1512670)
Supplement: Supplementary file 1 [file DataSheet1.zip › Raw Data/Raw Data/Original Documentation Fruit/Nicholls2017✔.pdf]

Atherosclerosis 261 (2017) 12–18

Contents lists available at ScienceDirect

Atherosclerosis

journal homepage: www.elsevier.com/locate/atherosclerosis

Comparative effects of cholesteryl ester transfer protein inhibition, statin or ezetimibe on lipid factors: The ACCENTUATE trial

Stephen J. Nicholls [a,*], Kausik K. Ray [b], Christie M. Ballantyne [c], Lauren A. Beacham [d], Debra L. Miller [d], Giacomo Ruotolo [d], Steven E. Nissen [e], Jeffrey S. Riesmeyer [d], for the ACCENTUATE Investigators

[a] *South Australian Health and Medical Research Institute, University of Adelaide, Adelaide, Australia*
[b] *Imperial College of London, London, UK*
[c] *Baylor College of Medicine and Methodist DeBakey Heart and Vascular Center, Houston, TX, USA*
[d] *Eli Lilly and Company, United States*
[e] *Cleveland Clinic, United States*

A R T I C L E I N F O

Article history:
Received 12 February 2017
Received in revised form
3 April 2017
Accepted 7 April 2017
Available online 8 April 2017

Keywords:
CETP
Clinical trials
Lipids
Cardiovascular disease

A B S T R A C T

Background and aims: The optimal approaches to management of patients treated with moderate statin doses on lipid parameters are unknown. The ACCENTUATE study aimed to compare the effects of adding the cholesteryl ester transfer protein inhibitor (CETP) evacetrapib, ezetimibe or increasing statin dose in atorvastatin-treated high-vascular risk patients on lipid parameters.
Methods: 366 patients with atherosclerotic cardiovascular disease (ASCVD) and/or diabetes were treated with atorvastatin 40 mg/day for 28 days prior to randomization to atorvastatin 40 mg plus evacetrapib 130 mg, atorvastatin 80 mg, atorvastatin 40 mg plus ezetimibe 10 mg or atorvastatin 40 mg plus placebo, daily for 90 days at 64 centers in the United States. Lipid parameters, safety and tolerability were measured.
Results: Addition of evacetrapib significantly reduced LDL-C (−33%) compared with ezetimibe (−27%, p=0.045), increasing statin dose (−6%) and statin alone (0%, p<0.001). Evacetrapib also decreased apoB by 23% compared to 19% with ezetimibe (p=0.06) and 7% with increased statin dose (p<0.001), and reduced Lp(a) by 29% (p<0.001 *vs.* other groups). Evacetrapib increased HDL-C (+125%), apoA-I (+46%), apoC-III (+50%) and apoE (+28%) (p<0.001 *vs.* other groups). Non-ABCA1-mediated efflux increased by 53% (p<0.001 *vs.* other groups) with evacetrapib. ABCA1-mediated efflux also increased by 13% with evacetrapib (p<0.001 *vs.* ezetimibe, p=0.002 *vs.* increasing statin dose, and p=0.004 *vs.* statin alone). Addition of evacetrapib to atorvastatin produced an increase in hsCRP compared with ezetimibe (p=0.02).
Conclusions: While evacetrapib improved traditional atherogenic and putative protective lipid measures compared with ezetimibe and increasing statin dose in patients with ASCVD and/or diabetes, it also adversely affected novel atherogenic risk factors. These findings may contribute to the lack of clinical benefit observed in the ACCELERATE trial.

© 2017 Elsevier B.V. All rights reserved.

1. Introduction

While lowering levels of low-density lipoprotein cholesterol (LDL-C) with statins has consistently been demonstrated to reduce cardiovascular morbidity and mortality in large outcome trials, there remains a substantial residual risk of clinical events [1–10]. This has prompted the search to identify additional therapies that will complement the role of statins to more effectively reduce cardiovascular risk [11]. By virtue of their ability to raise levels of high-density lipoprotein cholesterol (HDL-C), pharmacological inhibitors of cholesteryl ester transfer protein (CETP) have received considerable attention as a potential cardioprotective strategy [12–16].

Potent CETP inhibitors have also been demonstrated to reduce

* Corresponding author. South Australian Health and Medical Research Institute, PO Box 11060, Adelaide, SA, 5001, Australia.
E-mail address: stephen.nicholls@sahmri.com (S.J. Nicholls).

http://dx.doi.org/10.1016/j.atherosclerosis.2017.04.008
0021-9150/© 2017 Elsevier B.V. All rights reserved.

levels of atherogenic lipid parameters including both LDL-C and lipoprotein (a) [Lp(a)]. As a result, these agents may also be of utility in terms of their ability to increase the proportion of statin-treated patients who are able to achieve LDL-C treatment goals. While the LDL-C lowering effects of potent CETP inhibitors have been evaluated when administered as monotherapy or in combination with commonly used statin doses, they have not been directly compared with use of alternative lipid modifying strategies in statin-treated patients who have not currently achieved their treatment goals.

Evacetrapib is a potent CETP inhibitor, which has been demonstrated to raise HDL-C and cholesterol efflux, in addition to lowering LDL-C and Lp(a) in phase 2 studies.[15] The ACCENTUATE study was performed with the objective of comparing the effect of evacetrapib on lipid parameters with other lipid modifying strategies when used in combination with statin therapy.[17] Given the recent report that evacetrapib failed to reduce cardiovascular events in a large clinical outcome trial, despite favorable effects on both LDL-C and HDL-C, there is an urgent need to further understand the impact of this pharmacological strategy on atherogenic and putative protective lipid parameters [18].

2. Materials and methods

2.1. Study population

The study was a multicenter, randomized, double-blind, parallel, placebo-controlled clinical trial designed by the academic steering committee in collaboration with the sponsor. The institutional review boards of all participating centers approved the protocol and all patients provided informed written consent. Eligible patients were at least 18 years of age, with either atherosclerotic cardiovascular disease (defined as either [1] coronary stenosis ≥50%, [2] myocardial infarction or unstable angina ≥30 days prior to screening, [3] stable angina pectoris, [4] myocardial ischemia on stress testing, [5] coronary revascularization or [6] non-coronary atherosclerotic disease [peripheral arterial disease, atherosclerotic aortic disease or carotid artery disease]) or type 1 or 2 diabetes mellitus, treated with atorvastatin 40 mg daily for at least 30 days prior to screening and compliant with study drug for the 28 day lead-in phase, had a LDL cholesterol >70 mg/dL or non-HDL cholesterol >100 mg/dL at screening and prior to randomization and a triglyceride ≤400 mg/dL. Exclusion criteria included recent stroke or acute coronary syndrome, uncontrolled hypertension (systolic blood pressure ≥180 mmHg or diastolic blood pressure ≥110 mmHg), documented hyperaldosteronism, uncontrolled diabetes (hemoglobin A1c >9.5%), malignancy or significant liver, kidney or cardiac disease.

Potentially eligible patients entered a 28 day lead-in period in which they were treated with atorvastatin 40 mg daily. Patients with persistent LDL cholesterol >70 mg/dL or non-HDL cholesterol >100 mg/dL after this period and met all inclusion and none of the exclusion criteria were subsequently randomized in a 2:1:2:1 ratio to treatment with (i) atorvastatin 40 mg plus evacetrapib 130 mg daily, (ii) atorvastatin 80 mg daily, (iii) atorvastatin 40 mg plus ezetimibe 10 mg daily or (iv) atorvastatin 40 mg daily for 90 days. Randomization was performed at the site level by an Interactive Web Response System (IWRS).

2.2. Clinical visits and laboratory tests

Patients were examined at scheduled visits at days 30 and 90 during the treatment phase. Those patients who demonstrated study drug compliance during this period (defined as taking 80–120% study drug dosage) subsequently entered an open label extension phase, receiving treatment with atorvastatin 40 mg plus evacetrapib 130 mg daily for a planned 9 months with visits at the end of months 3, 6 and 9. A planned final study visit was to be performed 30 days following cessation of study drug. Lipoprotein levels and safety laboratory measurements were obtained at all visits. Blood pressure was measured at each visit by three replicate measurements. A central laboratory performed all biochemical determinations (Covance). Standard lipid profiles (LDL-C, HDL-C, triglycerides) were determined by enzymatic assay. LDL-C was also determined by beta quantification (ultracentrifugation followed by enzymatic determination). Serum apolipoprotein (A-I, A-II, B, C-III and E) levels were measured with standardized commercial immunoturbidimetric assays. High-sensitivity C-reactive protein (CRP) was determined by immunonephelometry. Cellular cholesterol efflux capacity of apoB depleted serum samples was determined by incubation with J774 macrophages. (Vascular Strategies). All cardiovascular events were reported by the investigators.

2.3. Statistical analyses

Efficacy analyses were performed using patients who completed the double-blind phase prior to study termination. An analysis of covariance (ANCOVA) model, with terms for treatment and baseline values, was used to perform between treatment comparisons for change from baseline to 90 day lipid values. Mixed-Effect Model Repeated Measure (MMRM) with terms for treatment, visit, baseline values as fixed effects and patient as a random effect were used to conduct analysis of repeated lipid measures across visits. Pearson coefficients were to asses correlations between lipid parameters. Statistical significance was established at 2-tailed $p<0.05$ level. Safety events between treatment groups were compared using Pearson Chi-square test, analysis of variance for blood pressure and Wilcoxon Signed-Rank Test for C-reactive protein with all treated patients in the analyses.

3. Results

The study was terminated on 12 October, 2015 following premature cessation of the large cardiovascular outcomes trial (ACCELERATE) evaluating the impact of evacetrapib due to clinical futility. At this point in time, 366 patients were randomized, 71% of patients had completed the double-blind treatment period and mean time of treatment was 75 days (Fig. 1).

3.1. Patient characteristics

The clinical characteristics of patients randomized to study drug are presented in Table 1. Patients were predominantly male and Caucasian, with a mean age of 63.4 years and high prevalence of obesity, diabetes and prior coronary revascularization. Baseline lipid and inflammatory parameters are summarized in Table 2. In the setting of treatment with atorvastatin 40 mg daily, patients demonstrated a median LDL-C 83.0 mg/dL and HDL-C 46.0 mg/dL. Levels of triglycerides (130 mg/dL), Lp(a) (43.1 nmol/L) and CRP (1.5 mg/L) were within normal limits.

3.2. Change in lipid and inflammatory parameters

Fig. 2 illustrates the percentage change in LDL-C (the primary endpoint) and HDL-C in the treatment groups. Addition of evacetrapib produced a significant reduction in LDL-C (−33.4%), compared to ezetimibe (−27.3%, $p=0.045$ for difference between treatments), atorvastatin 80 mg (−6.2%, $p<0.001$) and atorvastatin 40 mg (0.0%, $p < 0.001$). Addition of evacetrapib also produced a greater increase in HDL-C (+125.4%) compared to that observed in

ezetimibe (−2.2%, p<0.001), atorvastatin 80 mg (−6.1%, p<0.001) and atorvastatin 40 mg (+0.1%, p<0.001) groups.

The combination of evacetrapib and atorvastatin 40 mg daily also produced greater increases in apoA-I, apoA-II, apoC-III and apoE (p<0.001 compared with all other groups for each lipid parameter), and greater decreases in apoB (p<0.001 compared with both atorvastatin monotherapy groups) and Lp(a) (p<0.001 compared with all other groups). Full lipid results are shown in Table 3.

The correlation between changes in lipids and apolipoproteins is summarized in Table 4. In the overall cohort, changes in LDL-C correlated directly with changes in apoB (r = 0.83, p<0.001), and inversely with apoA-I (r = −0.24, p<0.001). In contrast, changes in HDL-C correlated directly with changes in apoA-I (r = 0.92, p<0.001), apoA-II (r = 0.70, p<0.001), apoC-III (r = 0.67, p<0.001) and apoE (r = 0.47, p<0.001), and inversely with changes in apoB (r = −0.27, p<0.001).

Ex vivo cellular cholesterol efflux capacity of serum samples was determined in 227 patients. The combination of evacetrapib and atorvastatin 40 mg daily produced increases in global cholesterol efflux of 35.1%, superior to −3% observed with atorvastatin 40 mg, −7.0% with atorvastatin 80 mg and −4.6% with ezetimibe (p<0.0001 for all comparisons) (Fig. 3). This benefit was derived from superior increases in ABCA1 but predominantly in non-ABCA1 dependent forms of cholesterol efflux in patients treated with the combination of evacetrapib and atorvastatin 40 mg daily.

Table 1
Clinical demographics.

Parameter	Cohort (n = 366)
Age (years)	63.4 ± 9.2
Males (%)	66.1
Caucasian (%)	81.1
BMI (kg/m^2)	31.7 ± 7.0
Atherosclerotic CVD (%)	74.3
Non-coronary clinical manifestation (%)	8.2
Diabetes (%)	50.5
Prior MI (%)	27.3
Prior coronary revascularization (%)	53.0

Clinical characteristics of all patients randomized to study drug.
BMI, body mass index; CVD, cardiovascular disease; MI, myocardial infarction.

3.3. *Safety and tolerability*

In general, evacetrapib was well tolerated with no greater incidence of discontinuation due to adverse events or biochemical evidence of either hepatic or muscle toxicity. While there was a 0.7 mmHg systolic blood pressure increase seen with evacetrapib, this difference was not statistically significant when compared with the other groups (Table 5).

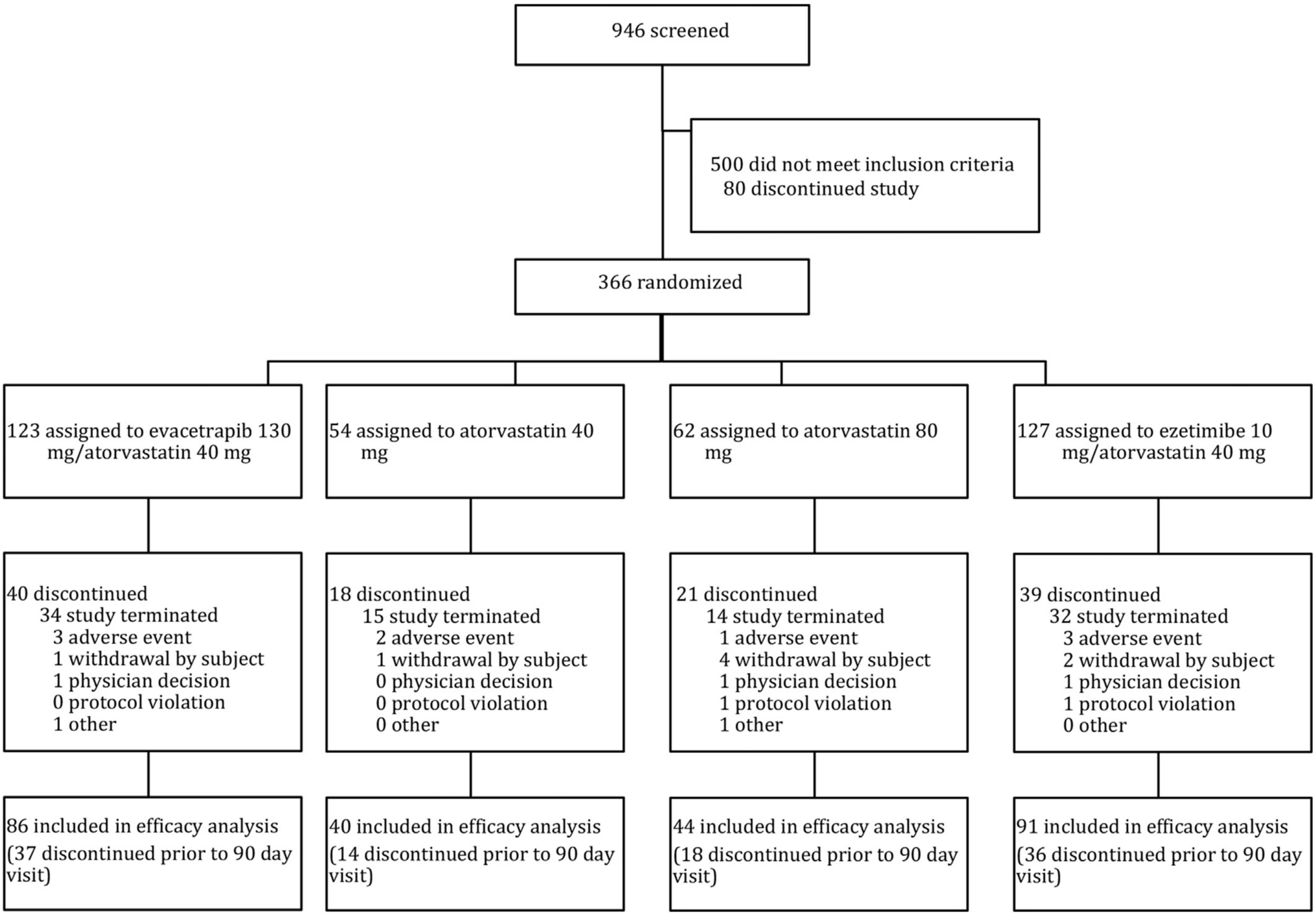

Fig. 1. Patient disposition.

Table 2
Baseline lipid and CRP values.

Parameter	Cohort (n = 261) median (IQR)
LDL cholesterol (mg/dL)	83.0 (22.0)
HDL cholesterol (mg/dL)	46.0 (17.0)
Triglycerides (mg/dL)	130.0 (88.0)
Non HDL cholesterol (mg/dL)	119.0 (30.0)
Apolipoprotein A-I (mg/dL)	140.5 (36.0)
Apolipoprotein A-II (mg/dL)	35.0 (8.0)
Apolipoprotein B (mg/dL)	81.0 (21.0)
Lipoprotein (a) (nmol/L)	43.1(138.2)
Apolipoprotein C-III (mg/dL)	11.3 (5.8)
Apolipoprotein E (mg/dL)	4.0 (2.0)
C-reactive protein (mg/L)[a]	1.5 (2.8)

Baseline lipid values for efficacy cohort completing 90 day visit and C-reactive protein levels for all randomized patients.
HDL, high-density lipoprotein; LDL, low-density lipoprotein.
[a] N = 366.

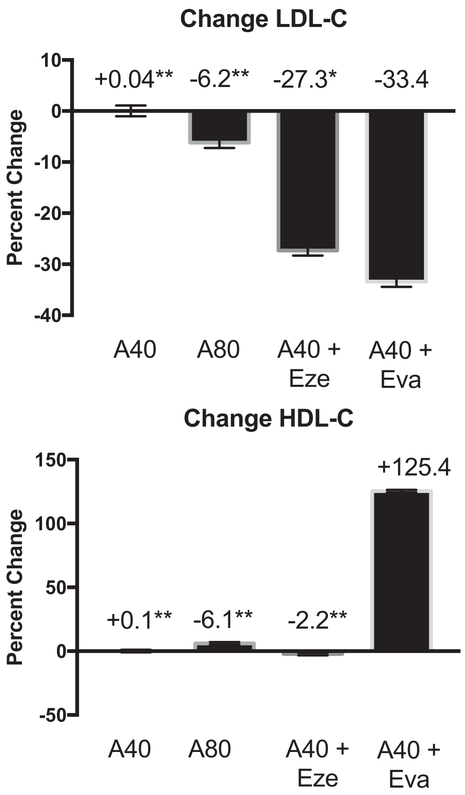

Fig. 2. Percentage change in LDL and HDL cholesterol.
Mean (SEM) percentage change from baseline in low-density lipoprotein cholesterol (upper panel) and high-density lipoprotein cholesterol (lower panel) in patients treated with atorvastatin 40 mg (A40), atorvastatin 80 mg (A80), atorvastatin plus ezetimibe (A40 + Eze) and atorvastatin 40 mg plus evacetrapib (A40 + Eva). $^{*}p<0.05$ and $^{**}p<0.001$ compared with atorvastatin 40 mg plus evacetrapib group.

4. Discussion

Treatment guidelines have increasingly focused on reduction of LDL cholesterol levels in patients deemed to be at high cardiovascular risk. However, despite increasing use of statins, many patients continue to have unacceptably high LDL cholesterol levels. Accordingly, new strategies are required to achieve more effective lipid lowering in high-risk patients. In the ACCENTUATE study, we aimed to determine the impact of addition of the CETP inhibitor, evacetrapib, on lipid levels in statin-treated patients in comparison with commonly employed strategies including statin dose escalation and combination therapy with ezetimibe. Given that the majority of patients are not typically treated with the highest statin doses, the study had the potential to inform on new strategies for lipid modification in clinical practice.

We observed that addition of evacetrapib to atorvastatin 40 mg daily resulted in increases in HDL cholesterol and incremental reductions in LDL cholesterol, comparable with previous reports. These changes were superior to those observed in patients who either underwent statin dose escalation or in those who were treated with the combination of atorvastatin and ezetimibe. Addition of evacetrapib also produced substantial increases in cellular cholesterol efflux capacity, suggesting potentially favorable effects on lipid mobilization. Given that evacetrapib administration was safe and well tolerated, these findings should have provided enthusiasm that CETP inhibition represents a viable strategy for use in high-risk patients who are not adequately treated with statin therapy.

However, these seemingly favorable findings with evacetrapib did not produce a clinical benefit. A larger cardiovascular outcomes trial of evacetrapib (ACCELERATE) was stopped prematurely due to clinical futility. This study reported no evacetrapib induced reduction in cardiovascular events, with no separation of event curves extending more than 2 years of treatment. This lack of benefit was observed despite favorable effects on both LDL and HDL cholesterol, widely accepted surrogate biomarkers of cardiovascular risk, and in cholesterol efflux capacity, a measure of HDL functionality. Accordingly, there is considerable debate as to why evacetrapib did not produce clinical benefit. The findings of the ACCENTUATE trial provide some opportunity to further characterize the impact of evacetrapib on atherogenic and protective lipid parameters and to potentially provide some rationale why this agent is not cardioprotective in statin-treated patients.

Evacetrapib predictably increased HDL cholesterol by 125%. Given that population and clinical studies consistently demonstrate an inverse relationship between HDL cholesterol and prospective cardiovascular risk, even in the setting of high intensity statin therapy, there has been considerable interest that raising HDL cholesterol should be beneficial. However, no HDL cholesterol raising strategy has been demonstrated to reduce cardiovascular events in large outcome trials of statin-treated patients. Furthermore, the lack of association between genetic variants, which are associated with levels of HDL cholesterol but not with cardiovascular risk in Mendelian randomization studies, suggests that HDL cholesterol is not a causal factor in atherosclerosis. It is therefore possible that raising HDL cholesterol does not represent a viable approach to reducing cardiovascular risk.

In addition to raising HDL cholesterol, evacetrapib increased cholesterol efflux capacity by 35%. This increase in cholesterol efflux was evidenced in both ABCA1 and non-ABCA1 mediated pathways, consistent with prior observations with potent CETP inhibitors of an increase not only in large, cholesterol rich HDL particles, but also in circulating concentrations of lipid-depleted pre-beta HDL. The lack of clinical benefit, despite these changes, is surprising given recent reports that cholesterol efflux capacity is an independent predictor of cardiovascular outcomes in a range of clinical settings. While these reports suggest a potential association between functional measures of lipid mobilization, they reflected cross-sectional analyses and observational prospective data and did not directly evaluate the relationship between changes in efflux

Table 3
Percent change in lipids and C-reactive protein.

Parameter	Atorvastatin 40 mg + evacetrapib (n = 86)	Atorvastatin 40 mg (n = 40)	*p* value compared with evacetrapib	Atorvastatin 80 mg (n = 44)	*p* value compared with evacetrapib	Atorvastatin 40 mg + ezetimibe (n = 91)	*p* value compared with evacetrapib
LDL-C	−33.4	0.04	<0.001	−6.2	<0.001	−27.3	0.045
HDL-C	125.4	0.11	<0.001	−6.1	<0.001	−2.2	<0.001
Triglycerides	−11.6	−13.0	0.76	−10.7	0.84	−13.8	0.52
Non HDL-C	−31.4	−5.0	<0.001	−9.4	<0.001	−24.4	<0.001
ApoA-I	+46.1	−0.3	<0.001	−6.1	<0.001	−2.4	<0.001
ApoA-II	+24.3	+0.9	<0.001	−3.9	<0.001	−4.9	<0.001
ApoB	−23.0	0.2	<0.001	−6.5	<0.001	−18.8	0.06
Lp(a)	−28.7	4.5	<0.001	3.9	<0.001	13.4	<0.001
ApoC-II	+28.6	+1.0	<0.001	−12.7	<0.001	−21.4	<0.001
ApoC-III	+50.1	1.5	<0.001	−12.8	<0.001	−9.9	<0.001
ApoE	+28.0	−3.5	<0.001	−8.7	<0.001	−11.2	<0.001
CRP	0.15	−0.02	0.11	−0.10	0.08	−0.11	0.02

Percentage change from baseline in lipid values and median change from baseline in C-reactive protein (CRP) levels across treatment groups.
Apo, apolipoprotein; HDL-C, high-density lipoprotein cholesterol; LDL, low-density lipoprotein; Lp(a), lipoprotein (a).

Table 4
Correlation between changes in lipids and apolipoproteins.

Parameter	Total cohort (n = 261)		Pooled atorvastatin (n = 84)		Atorvastatin + ezetimibe (n = 91)		Atorvastatin + evacetrapib (n = 86)	
	LDL-C	HDL-C	LDL-C	HDL-C	LDL-C	HDL-C	LDL-C	HDL-C
ApoB	r = 0.83	r = −0.27	r = 0.82	r = 0.11	r = 0.71	r = 0.05	r = 0 0.80	r = −0.04
	p < 0.001	*p* < 0.001	*p* < 0.001	*p*=0.36	*p* < 0.001	*p*=0.67	*p* < 0.001	*p*=0.72
ApoA-I	r = −0.24	r = 0.92	r = 0.18	r = 0 0.46	r = 0.01	r = 0 0.60	r = 0.03	r = 0 0.79
	p<0.001	*p* < 0.001	*p*=0.13	*p*<0.001	*p* = 0.91	*p*<0.001	*p* = 0.78	*p*<0.001
ApoA-II	r = −0.09	r = 0.70	r = 0 0.13	r = 0.35	r = 0.17	r = 0.37	r = 0 0.19	r = 0.27
	p = 0.19	*p*<0.001	*p* = 0.28	*p* = 0.002	*p* = 0.12	*p*<0.001	*p* = 0.09	*p* = 0.02
ApoC-III	r = −0.04	r = 0.67	r = 0.14	r = 0.12	r = 0.12	r = −0.08	r = 0.31	r = 0.48
	p = 0.54	*p* < 0.001	*p* = 0.25	*p* = 0.29	*p* = 0.28	*p* = 0.47	*p*=0.005	*p*<0.001
ApoE	r = 0.09	r = 0.47	r = 0.189	r = 0.130	r = 0.028	r = −0.132	r = 0.383	r = 0.280
	p = 0.17	*p* < 0.001	*p* = 0.12	*p* = 0.27	*p* = 0.80	*p* = 0.23	*p* < 0.001	*p* = 0.01

Correlation between percentage change in both low-density lipoprotein cholesterol (LDL-C) and high-density lipoprotein cholesterol (HDL-C) and apolipoproteins (apo).

and subsequent outcomes. Unfortunately, both quantitative and functional changes attributed to HDL with evacetrapib failed to result in clinical benefit, further bringing into question the HDL hypothesis.

Even if the HDL associated effects of evacetrapib are not taken into consideration, its effects on LDL cholesterol had also provided enthusiasm that it should lower cardiovascular risk. The association between LDL cholesterol and cardiovascular risk is supported by the statin outcome trials, meta-analyses demonstrating a direct relationship between LDL cholesterol and cardiovascular events and genomic studies reporting that polymorphisms regulating LDL cholesterol levels also associate with cardiovascular risk. The recent findings that incremental LDL cholesterol lowering with the addition of ezetimibe to statin therapy improves cardiovascular outcomes provides further support for the concept of the beneficial effects of lipid lowering [19]. We observed that use of evacetrapib produced 33% lowering of LDL cholesterol, which was superior to that reported with either statin dose escalation or addition of ezetimibe. This alone should have produced a reduction in cardiovascular events.

However, while evacetrapib also lowered apoB, a surrogate measure of circulating LDL particle concentration, the 23% reduction was disproportionally lower than the degree of LDL cholesterol lowering. This less robust reduction in atherogenic lipoproteins may have contributed to a less favorable impact on cardiovascular events than expected. LDL cholesterol measurements are less precise in the setting of CETP inhibition and it is uncertain whether the association between LDL cholesterol lowering observed with statins and ezetimibe can be extrapolated to this therapeutic class. Furthermore, little is known regarding the atherogenicity of LDL particles in the setting of CETP inhibition. While LDL cholesterol has proven to be an effective biomarker for both risk prediction and therapeutic targeting, it is possible that the mechanism of LDL cholesterol lowering may be an important factor influencing its impact on cardiovascular outcomes.

The ACCENTUATE study provided assessment of the impact of evacetrapib on additional lipoproteins, which may provide some rationale for the lack of cardiovascular benefit. While evacetrapib lowered Lp(a), baseline levels were within the normal range and there is currently no evidence that Lp(a) lowering translates to clinical benefit. CETP inhibition generates large, cholesterol rich HDL particles with a larger surface area for occupation by apolipoproteins. While levels of apoA-I, the major protein carried on HDL, predictably increased, we also observed greater levels of apoE and apoC-III, which directly correlated with increases in HDL cholesterol levels. The implications of these findings remain uncertain. However, population and genomic studies demonstrate that elevated apoC-III levels associate with cardiovascular risk. ApoC-III enriched HDL particles have been reported to possess less functional activity, although we observed an increase in cholesterol efflux capacity with evacetrapib. Whether this increase in efflux activity is insufficient to modulate outcomes highlights the

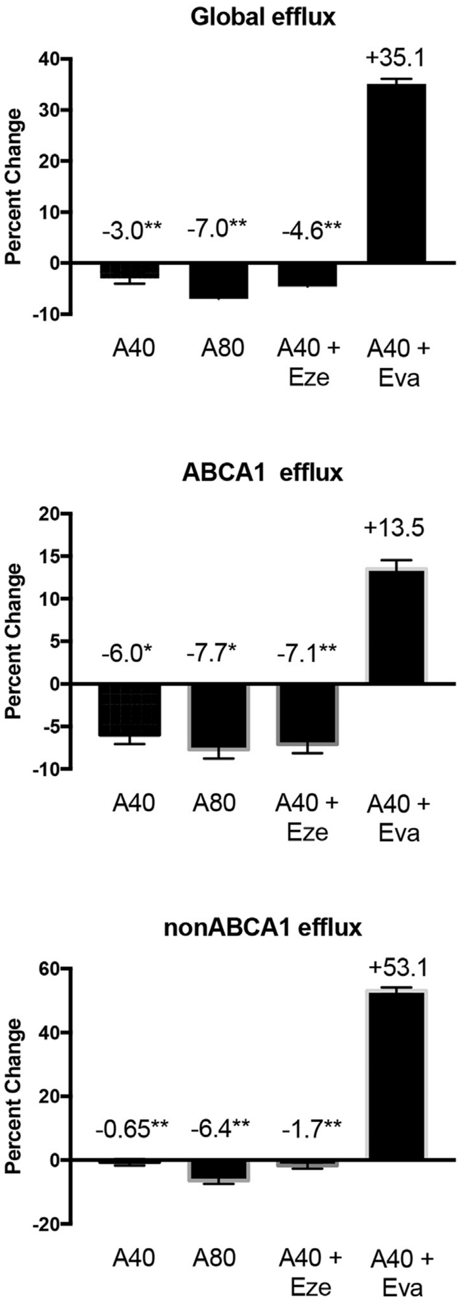

Fig. 3. Percentage change in cholesterol efflux.
Mean (SEM) percentage change from baseline in global (upper panel), ABCA1 dependent (middle panel) and nonABCA1 dependent (lower panel) cholesterol efflux in patients treated with atorvastatin 40 mg (A40), atorvastatin 80 mg (A80), atorvastatin plus ezetimibe (A40 + Eze) and atorvastatin 40 mg plus evacetrapib (A40 + Eva). **p*<0.01 and ***p*<0.001 compared with atorvastatin 40 mg plus evacetrapib group.

knowledge gap linking therapeutic changes in efflux and cardiovascular risk. The potential impact of apoC-III on additional HDL activities remains uncertain. Reports that apoC-III may exert a direct proinflammatory effect at the level of the artery wall may provide a rationale, by which large HDL particles generated by CETP inhibition serve as a vehicle to deliver apoC-III to the artery wall. Additional research will be required to elucidate the potential impact of apoC-III elevations with CETP inhibition.

It is possible that other effects of evacetrapib may have counteracted any potential benefit of atherogenic lipoprotein lowering. Whether this reflects yet to be characterized adverse effects of HDL or apoC-III raising or reports from larger studies of small increases in systolic blood pressure and CRP remains to be elucidated. They do provide important factors that warrant further investigation. Whether these findings reflect a class effect or are limited to evacetrapib also requires further study. A large outcome trial of the CETP inhibitor, anacetrapib, is ongoing and will involve longer patient treatment. This will provide further important insights in determining whether CETP inhibition may ultimately prove to be a useful clinical approach to reducing cardiovascular risk in the statin-treated patient. Whether it is a useful strategy in the absence of statin therapy has not been well studied. For now, CETP continues to be an interesting strategy with no evidence to support its use in patients at a high risk of cardiovascular events.

Conflict of interest

SJN reports receiving research support from Amgen, AstraZeneca, Cerenis, Eli Esperion, InfraReDx, Lilly, LipoScience, Novartis, Resverlogix and Sanofi-Regeneron and is a consultant for Amgen, AstraZeneca, Boehringer Ingelheim, CSL Behring, Eli Lilly, Kowa, Merck, Novartis, Roche, Sanofi-Regeneron and Takeda. .KKR reports grants and/or personal fees from Pfizer, MSD, Astra Zeneca, Sanofi, Aegerion, Regeneron, Abbvie, Kowa, Cerenis, Medicines Company, Lilly, Esperion, Amgen, Cipla, Algorithm, Takeda, Boehringer Ingelheim and Novo Nordisk within the last 12 months outside of the submitted work. CMB has received research support paid to his institution from Abbott Diagnostic, Amarin, Amgen, Eli Lilly, Esperion, Ionis, Novartis, Pfizer, Regeneron, Roche Diagnostic, Sanofi-Synthelabo, NIH, AHA, ADA and is a consultant for Abbott Diagnostics, Amarin, Amgen, Astra Zeneca, Boehringer Ingelheim, Eli Lilly, Esperion, Ionis, Matinas BioPharma Inc, Merck, Novartis, Pfizer, Regeneron, Roche Diagnostic, Sanofi-Synthelabo. LAB, DLM, GR and JSR are employees of Eli Lilly. SEN has received research support from Amgen, Abbvie, AstraZeneca, Cerenis, Eli Lilly, Esperion Therapeutics, Novo-Nordisk, The Medicines Company, Orexigen, Pfizer, and Takeda. He has consulted for a number of pharmaceutical companies without financial compensation. All honoraria, consulting fees or any other payments from any for-profit entity are paid directly to charity, so that neither income nor any tax deduction is received. The ACCENTUATE study was sponsored by Eli Lilly.

Table 5
Clinical and biochemical adverse events.

Parameter	Atorvastatin 40 mg (n = 54)	Atorvastatin 80 mg (n = 62)	Atorvastatin 40 mg + ezetimibe (n = 127)	Atorvastatin 40 mg + evacetrapib (n = 123)
Discontinuation due to adverse events	3.7%	1.6%	2.4%	2.4%
ALT > 3x ULN	0%	0%	0%	0.8%
Bilirubin > 2x ULN	0%	0%	0%	0%
CK > 5x ULN	0%	1.6%	0%	0%
Systolic blood pressure (mmHg)	−3.6 ± 13.4	−1.8 ± 11.7	−3.1 ± 15.1	+0.7 ± 13.8

Incidence of clinical and biochemical adverse events and mean change from baseline and standard deviation systolic blood pressure across treatment groups.

Clinical trial number

Clinicaltrials.gov registration number: NCT02227784.

Appendix A. Supplementary data

Supplementary data related to this article can be found at http://dx.doi.org/10.1016/j.atherosclerosis.2017.04.008.

References

[1] Randomised trial of cholesterol lowering in 4444 patients with coronary heart disease: the Scandinavian Simvastatin Survival Study (4S), Lancet 344 (1994) 1383–1389.
[2] Long-Term Intervention With Pravastatin in Ischaemic Disease (LIPID) Study Group, Prevention of cardiovascular events and death with pravastatin in patients with coronary heart disease and a broad range of initial cholesterol levels, N. Engl. J. Med. 339 (1998) 1349–1357.
[3] Heart Protection Study Collaborative Group, MRC/BHF Heart Protection Study of cholesterol lowering with simvastatin in 20,536 high-risk individuals: a randomized placebo-controlled trial, Lancet 360 (2002) 7–22.
[4] J.R. Downs, M. Clearfield, S. Weis, et al., Primary prevention of acute coronary events with lovastatin in men and women with average cholesterol levels: results of AFCAPS/TexCAPS, JAMA 279 (1998) 1615–1622.
[5] F.M. Sacks, M.A. Pfeffer, L.A. Moye, et al., Cholesterol and Recurrent Events Trial Investigators. The effect of pravastatin on coronary events after myocardial infarction in patients with average cholesterol levels, N. Engl. J. Med. 335 (1996) 1001–1009.
[6] J. Shepherd, S.M. Cobbe, I. Ford, et al., West of Scotland Coronary Prevention Study Group. Prevention of coronary heart disease with pravastatin in men with hypercholesterolemia, N. Engl. J. Med. 333 (1995) 1301–1307.
[7] C. Baigent, A. Keech, P.M. Kearney, et al., Cholesterol Treatment Trialists' (CTT) Collaborators. Efficacy and safety of cholesterol-lowering treatment: prospective meta-analysis of data from 90,056 participants in 14 randomised trials of statins, Lancet 366 (2005) 1267–1278.
[8] P.M. Ridker, E. Danielson, F.A. Fonseca, et al., JUPITER Study Group. Rosuvastatin to prevent vascular events in men and women with elevated C-reactive protein, N. Engl. J. Med. 359 (2008) 2195–2207.
[9] C.P. Cannon, E. Braunwald, C.H. McCabe, et al., Pravastatin or atorvastatin evaluation infection therapy-thrombolysis in myocardial infarction 22 investigators. Intensive versus moderate lipid lowering with statins after acute coronary syndromes, N. Engl. J. Med. 350 (2004) 1495–1504.
[10] J.C. LaRosa, S.M. Grundy, D.D. Waters, et al., Treating to New Targets (TNT) Investigators. Intensive lipid lowering with atorvastatin in patients with stable coronary disease, N. Engl. J. Med. 352 (2005) 1425–1435.
[11] S.J. Nicholls, HDL: still a target for new therapies? Curr. Opin. Investig. Drugs 9 (2008) 950–956.
[12] P.J. Barter, M. Caulfield, M. Eriksson, et al., ILLUMINATE Investigators. Effects of torcetrapib in patients at high risk for coronary events, N. Engl. J. Med. 357 (2007) 2109–2122.
[13] G.G. Schwartz, A.G. Olsson, M. Abt, et al., dal-OUTCOMES Investigators. Effects of dalcetrapib in patients with a recent acute coronary syndrome, N. Engl. J. Med. 367 (2012) 2089–2099.
[14] S.J. Nicholls, H.B. Brewer, J.J. Kastelein, et al., Effects of the CETP inhibitor evacetrapib administered as monotherapy or in combination with statins on HDL and LDL cholesterol: a randomized controlled trial, JAMA 306 (2011) 2099–2109.
[15] S.J. Nicholls, A.M. Lincoff, P.J. Barter, et al., Assessment of the clinical effects of cholesteryl ester transfer protein inhibition with evacetrapib in patients at high-risk for vascular outcomes: rationale and design of the ACCELERATE trial, Am. Heart J. 170 (2015) 1061–1069.
[16] C.P. Cannon, S. Shah, H.M. Dansky, et al., DEFINE Investigators. Safety of anacetrapib in patients with or at high risk for coronary heart disease, N. Engl. J. Med. 363 (2010) 2406–2415.
[17] https://www.eas-society.org/news/291661/EAS-2016-Innsbruck-Highlights-from-the-congress-Monday-May-30.htm Accessed December 19, 2016.
[18] http://www.thecardiologyadvisor.com/acc-meeting-highlights/evacetrapib-did-not-reduce-cv-events-in-high-risk-patients/article/487228/ Accessed December 19, 2016.
[19] C.P. Cannon, M.A. Blazing, R.P. Giugliano, et al., IMPROVE-IT Investigators. Ezetimibe added to statin therapy after acute coronary syndromes, N. Engl. J. Med. 372 (2015) 2387–2397.
