# Supplementary material for: Lipid-lowering effect of combined therapy with high-intensity statins and CETP inhibitors: a Systematic Review and meta-analysis
Source: Front Endocrinol (Lausanne). 2025 May 1;16:1512670. doi: 10.3389/fendo.2025.1512670 (PMC12078159; doi:10.3389/fendo.2025.1512670)
Supplement: Supplementary file 1 [file DataSheet1.zip › Raw Data/Raw Data/Original Documentation Fruit/Derks2010(1)✔.pdf]

Expert Opinion

1. Introduction
2. Methods
3. Results
4. Discussion

No clinically relevant drug–drug interactions when dalcetrapib is co-administered with atorvastatin

Michael Derks†, Markus Abt, Graeme Parr, Georgina Meneses-Lorente, Anne-Marie Young & Mary Phelan
†*F. Hoffmann-La Roche Ltd, Department of Clinical Pharmacology, Basel, Switzerland*

Objectives: Dalcetrapib, which targets cholesteryl ester transfer protein, is in clinical development for prevention of cardiovascular events and is likely to be used concomitantly with statins. Two studies investigated co-administration of dalcetrapib with atorvastatin and any effects of the timing of atorvastatin on the pharmacokinetics of dalcetrapib.
Research design and methods: Two crossover studies were performed in healthy subjects: a two-period study of dalcetrapib 900 mg concurrently with atorvastatin (concurrent dosing study) and a three-period study of dalcetrapib 600 mg (dose chosen for Phase III) with atorvastatin concurrently or serially 4 h after dalcetrapib (interval dosing study).
Main outcome measures: The primary pharmacokinetic end points were AUC_{0-24} and C_{max}; lipid effects and tolerability were secondary end points.
Results: In the concurrent study (n = 26), co-administration reduced dalcetrapib AUC_{0-24} and C_{max} and caused small changes in AUC_{0-24} and C_{max} of atorvastatin and its active metabolites. In the interval study (n = 52), serial and concurrent co-administration of atorvastatin resulted in similar reductions in dalcetrapib exposure that were comparable to those observed in the concurrent dosing study. Co-administration did not decrease the efficacy of dalcetrapib or atorvastatin and was generally well tolerated.
Conclusions: These results indicate no clinically relevant interactions for co-administration of dalcetrapib with atorvastatin.

Keywords: atorvastatin, CETP, dalcetrapib, drug–drug interactions

Expert Opin. Investig. Drugs (2010) ***19****(10):1135-1145*

Copyright © 2010 Informa Healthcare
Not for sale or commercial distribution
Unauthorized use prohibited. Authorised users can download, display, view and print a single copy for personal use

1. Introduction

Despite the efficacy of 3-hydroxy-3-methylglutaryl co-enzyme A (HMG-CoA) reductase inhibitors (statins) in reducing the risk of cardiovascular disease (CVD) through the lowering of low-density lipoprotein cholesterol (LDL-C), there remains a substantial residual risk of CVD that is not adequately addressed by current treatments [1]. Raising levels of high-density lipoprotein cholesterol (HDL-C) is a strategy to address the residual risk of CVD that persists despite current standard of care [2-4]. Increase in HDL-C may potentially be achieved through inhibition of cholesteryl ester transfer protein (CETP) activity, as decreased plasma levels of CETP have been associated with increased HDL-C [5] and a decreased risk of coronary artery disease [6]. Dalcetrapib, a compound targeting CETP, is currently in clinical development and has been shown to increase levels of HDL-C in Phase II trials [7,8].

Dalcetrapib will probably be taken concomitantly with statins. Consequently, two studies were performed to investigate any pharmacokinetic interactions between dalcetrapib and the highest commonly used dose of atorvastatin (40 mg). One study investigated the effect of concurrent co-administration on the pharmacokinetic

informa
healthcare

Expert Opin. Investig. Drugs Downloaded from informahealthcare.com by University of Southern California on 04/05/14
For personal use only.

All rights reserved: reproduction in whole or in part not permitted
RIGHTSLINK

parameters for dalcetrapib, atorvastatin and major metabolites of atorvastatin (the concurrent dosing study), while another study was used to establish the effect of an interval between doses of dalcetrapib and atorvastatin on the relative bioavailability of dalcetrapib (the interval dosing study). The safety and tolerability of the combination along with effects on lipid profiles and CETP were also investigated. While the interval dosing study used the Phase III dose of dalcetrapib (600 mg), the concurrent dosing study used a higher dose (900 mg) that was used in earlier studies [7-9].

2. Methods

2.1 Study population

Two drug–drug interaction studies were performed in healthy participants: a study of dalcetrapib 900 mg administered concurrently with atorvastatin 40 mg (concurrent dosing study), and a study of dalcetrapib 600 mg administered either concurrently with atorvastatin 40 mg or serially with atorvastatin 40 mg 4 h later (interval dosing study). The 40 mg dose of atorvastatin was used as this is the highest commonly used dose of this statin. The concurrent dosing study was limited to males, while the interval dosing study included both males and females who were surgically sterile or postmenopausal for > 1 year, and who were not receiving hormone replacement therapy. Both studies included subjects with body mass index of 18 – 32 kg/m^2 inclusive, who were aged between 18 – 65 years inclusive. Exclusion criteria included clinically significant symptoms of infectious disease, known history of porphyria, myopathy, or active liver disease, use of concomitant medication except paracetamol, recent use of a CYP3A4 inhibitor or inducer, clinically relevant history of drug or alcohol misuse or abuse, alcohol intake greater than approximately 21 units per week, and positive drugs of abuse test at screening.

The studies were in compliance with the principles of the Declaration of Helsinki and were performed according to Good Clinical Practice Guidelines. Written informed consent was provided by each participant and the study protocols were reviewed by an independent ethics committee (Comité Consultatif de Protection des Personnes dans la Recherche Biomédicale d'Alsace, Strasbourg, France).

2.2 Study medication

Dalcetrapib 300 mg tablets were provided by Clinical Trial Supplies, F. Hoffmann-La Roche Ltd (Basel, Switzerland) in accordance with Roche standards and local regulations. Atorvastatin 40 mg tablets were purchased locally by the Roche Clinical Pharmacology Unit (Strasbourg, France).

2.3 Study design

The concurrent and interval studies were randomized, open-label, crossover studies with two and three treatment periods respectively, separated by a washout period (**Figure 1**). Screening was performed on days -28 to -2 and included a full medical history and physical examination with electrocardiogram (ECG), vital signs, and laboratory tests. On day -1, blood and urine samples were collected for laboratory safety and drugs of abuse tests and a medical re-evaluation was performed.

In the concurrent dosing study, the pharmacokinetic profile of dalcetrapib was obtained from venous blood samples on day 13 of Treatment A (dalcetrapib plus atorvastatin) and day 8 of Treatment B (dalcetrapib alone); pharmacokinetic profiles for atorvastatin were obtained on days 5 and 13 of Treatment A. In the interval dosing study, profiles for dalcetrapib were determined on day 7 of Treatment A (dalcetrapib alone), B (dalcetrapib plus atorvastatin) and C (dalcetrapib plus atorvastatin separated by 4 h).

2.4 Pharmacokinetic assessments

The primary pharmacokinetic parameters were AUC_{0-24} and C_{max} for dalcetrapib and atorvastatin in the concurrent dosing study, and for dalcetrapib in the interval dosing study.

2.4.1 *Measurement of plasma levels of dalcetrapib, atorvastatin and metabolites*

Plasma samples were treated with dithiothreitol for thiolysis and dalcetrapib active form concentration was determined after formation of the *N*-ethylmaleimide derivative using validated liquid chromatography-tandem mass spectrometry by F. Hoffmann-La Roche Ltd (Basel, Switzerland) in the concurrent dosing study and by Swiss BioAnalytics AG (Birsfelden, Switzerland) in the interval dosing study. In the concurrent dosing study, plasma concentrations of the methyl and glucuronide metabolites of dalcetrapib (dalcetrapib-S-Me and dalcetrapib-S-Glu) were determined by Roche, and plasma concentrations of atorvastatin and its five metabolites (2-OH-atorvastatin, 4-OH-atorvastatin, atorvastatin lactone, 2-OH-atorvastatin lactone and 4-OH-atorvastatin lactone) were determined by Pharma Bio-Research Group BV (Zuidlaren, The Netherlands).

The precision and accuracy of the assays for dalcetrapib, atorvastatin and their metabolites were within satisfactory limits for the studies (see Supplementary Table 1) as determined from the analysis of quality control samples. The lower limits of quantification were 5 ng/ml for dalcetrapib and dalcetrapib-S-Me, 50 ng/ml for dalcetrapib-S-Glu, and 0.25 ng/ml for atorvastatin and its metabolites.

2.5 Pharmacodynamic assessments

CETP mass and activity were measured in the concurrent dosing study using a scintillation proximity assay and a sandwich enzyme immunoassay respectively by Pacific Biometrics, Inc. (Seattle, WA, USA). Also in the concurrent dosing study, fasting lipid profiles were assessed and included measurement of HDL-C, LDL-C, triglycerides (TG), very low-density lipoprotein cholesterol (VLDL-C), apolipoprotein (apo) A-I, apoA-II, apoB, and total cholesterol. The composition of lipoprotein subfractions was measured by fast lipoprotein

Expert Opin. Investig. Drugs Downloaded from informahealthcare.com by University of Southern California on 04/05/14
For personal use only.

RIGHTSLINK

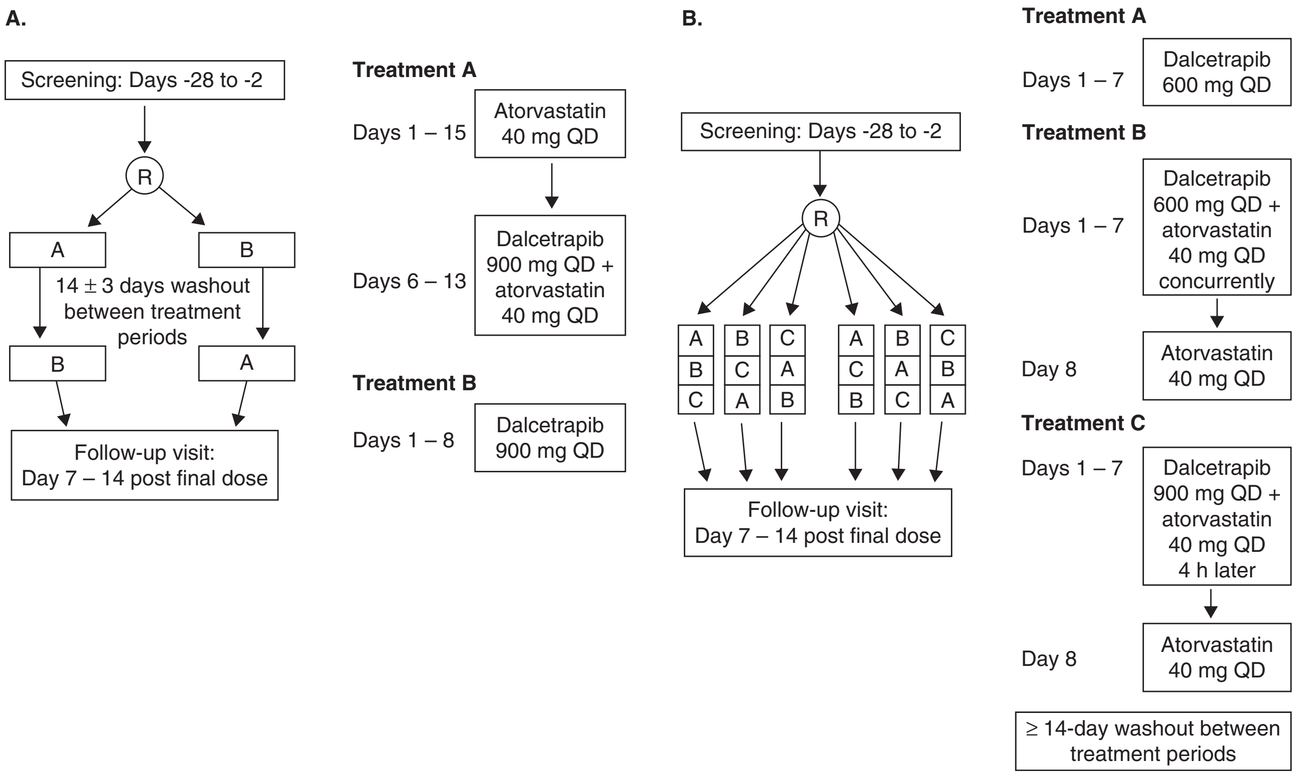

Figure 1. Study design. A. Concurrent dosing study. **B.** Interval dosing study.

QD: Once daily; R: Randomization.

chromatography (FLPC) and ^{1}H nuclear magnetic resonance (NMR). Lipid profile analysis and FLPC was performed by CRL Medinet BV (Breda, The Netherlands). Analysis of lipoprotein subclasses by NMR was performed by LipoScience, Inc. (Raleigh, NC, USA).

Assessment of HMG-CoA reductase activity in the interval dosing study was measured by PPD Global Central Labs (Highland Heights, Kentucky, USA) and involved incubation of atorvastatin extracted from plasma with [^{14}C] HMG-CoA and recombinant human HMG-CoA reductase, before and after hydrolysis with 0.5 N KOH, to determine the level of active and total HMG-CoA reductase inhibitor, respectively. The amount of the reaction product, [^{14}C]-mevalonate, as determined radiometrically, is inversely proportional to the concentration of active and total HMG-CoA reductase inhibitors in ng Eq/ml. Assessment of paraoxonase-1 (PON1) activity was included as an exploratory end point in the interval dosing study and was assessed by Eurofins Medinet BV (Breda, The Netherlands) using a spectrophotometric method.

2.6 Safety assessments

Safety was assessed by monitoring adverse events (AEs), laboratory testing, vital signs and ECG. The investigator assessed and recorded the intensity of AEs and relationship to study treatment. Laboratory tests included hematology, biochemistry including electrolytes, liver enzymes, thyroid function, reproductive function, coagulation, urinalysis, serology, alcohol breath test, and drugs of abuse.

2.7 Statistical analysis

The primary pharmacokinetic parameters AUC_{0-24} and C_{max} were natural log transformed and evaluated with a linear mixed-effects model with treatment as a fixed effect and subject as a random effect. Analysis of variance was performed to assess differences between treatments for the primary pharmacokinetic parameters. The 90% *CIs* on the estimated geometric mean ratio (GMR) of combination therapy to single therapy for AUC_{0-24} and C_{max} were used to judge the significance of any observed interactions. No statistical testing was performed for pharmacodynamic or safety parameters.

In the concurrent dosing study, a planned sample size of 24 was chosen to ensure that with at least 80% probability, the 90% *CIs* for the relative effects of combined treatment of atorvastatin and dalcetrapib on atorvastatin and on dalcetrapib do not extend by a factor of > 1.5 above or by a factor of < 0.667 below the true relative difference. This sample size assumed a within-subject coefficient of variation of 43%. In the interval dosing study, a planned sample size of 42 was chosen to ensure at least 36 evaluable participants. For the largest within-subject coefficient of variation (47%), this sample size would ensure with at least 75% probability

Expert Opin. Investig. Drugs Downloaded from informahealthcare.com by University of Southern California on 04/05/14
For personal use only.

Table 1. Baseline characteristics.

	Concurrent dosing study	Interval dosing study
n	26	52
Gender, male (%)	26 (100)	47 (90)
Race		
White (%)	26 (100)	49 (94)
Black (%)	0	1 (2)
Asian (%)	0	2 (4)
Age, years	33.9 ± 12.2	32.0 ± 11.7
Weight, kg	76.3 ± 10.2	77.6 ± 10.4
Height, cm	177.3 ± 6.3	176.9 ± 6.8
Body mass index, kg/m^2	24.3 ± 2.9	24.8 ± 3.0

Values are mean ± standard deviation unless otherwise noted.

that the 90% *CIs* for the relative effects of combined treatment of atorvastatin and dalcetrapib on dalcetrapib did not extend by a factor of > 1.33 above or by a factor of < 0.72 below the true relative difference.

3. Results

3.1 Baseline demographic characteristics

In the concurrent dosing study, 26 participants were randomized and 22 completed the study; three participants were withdrawn due to AEs, and one refused treatment. In the interval dosing study, 52 participants were randomized and 39 participants completed. Of the 13 participants who failed to complete the interval dosing study, five were withdrawn due to AEs, three due to protocol deviations, and five refused treatment. Baseline demographic characteristics are shown in **Table 1**. In the interval study, 90% of participants were male. The concurrent dosing study was conducted from 12 April 2005 to 14 June 2005, and the interval dosing study was conducted from 7 February 2007 to 16 August 2007.

3.2 Pharmacokinetic results

In the concurrent dosing study, dalcetrapib plasma concentrations were reduced by concurrent co-administration with atorvastatin (**Figure 2A**). This was reflected by a reduction of approximately 30% for AUC_{0-24}; GMR (90% CI); 0.699 (0.655, 0.747) and a reduction of approximately 15% for C_{max}, 0.852 (0.779, 0.930) relative to dalcetrapib monotherapy. The terminal half-life of dalcetrapib was also reduced during co-administration with atorvastatin (18.53 ± 5.03 vs 22.98 ± 7.54 h for dalcetrapib alone). Co-administration had no effect on plasma levels of the pharmacologically inactive dalcetrapib metabolites dalcetrapib-S-Glu and dalcetrapib-S-Me.

Atorvastatin exposure based on AUC_{0-24} remained essentially unchanged during co-administration with dalcetrapib (**Figure 2B**; **Table 2**); however, there was a small increase (11%) in atorvastatin C_{max} and a small decrease (14%) in the C_{max} of its active metabolite, 2-OH-atorvastatin (**Table 2**). There was no significant change in 4-OH atorvastatin exposure as assessed by the GMRs of AUC_{0-24} and C_{max}. Among the inactive metabolites, atorvastatin lactone exposure was reduced by approximately 30% during co-administration with dalcetrapib, whereas 4-OH-atorvastatin lactone C_{max} was increased by approximately 16%. No significant change was observed in 2-OH-atorvastatin lactone exposure during co-administration of dalcetrapib.

In the interval dosing study, dalcetrapib exposure was reduced by concurrent and serial (+ 4 h) co-administration of atorvastatin compared with dalcetrapib 600 mg alone. The AUC_{0-24} and C_{max} for dalcetrapib decreased by approximately 27 and 13%, respectively, with concurrent co-administration of atorvastatin and by 35 and 20%, respectively, for serial co-administration (**Table 3**; **Figure 2C**). Exposure to atorvastatin and its active metabolites was not measured in the interval dosing study with dalcetrapib 600 mg. Because 90% of participants in the interval study were male, no test for gender effects was performed.

3.3 Pharmacodynamic results

3.3.1 *Effect of concurrent co-administration of dalcetrapib with atorvastatin on CETP activity and mass*

In the concurrent dosing study, dalcetrapib 900 mg administered alone and concurrently with atorvastatin resulted in decreases in CETP activity of 32.7 and 35.5% respectively. CETP mass was increased by 92.5 and 53.0% when dalcetrapib was administered alone and concurrently with atorvastatin, respectively. Administration of atorvastatin alone for 5 days had little effect on either CETP activity or mass (data not shown).

3.3.2 *Effect of concurrent co-administration of dalcetrapib with atorvastatin on plasma lipids*

Assessment of plasma lipids was only performed in the concurrent dosing study. Similar increases in HDL-C were observed for dalcetrapib 900 mg administered alone and concurrently with atorvastatin (**Table 4**) and were accompanied by small increases in mean HDL particle size (data not shown). A reduction in LDL-C was observed in all groups in the concurrent study, with dalcetrapib co-administered with atorvastatin achieving the largest decrease, this was also accompanied by the largest observed decrease in atherogenic small LDL particles (data not shown). Co-administration of dalcetrapib with atorvastatin was associated with the largest reductions in VLDL-C and total cholesterol. The reduction in triglyceride levels was comparable between atorvastatin administered alone and concurrently with dalcetrapib (**Table 4**).

3.3.3 *Effect of concurrent or serial co-administration of dalcetrapib with atorvastatin on HMG-CoA reductase activity and PON1 activity*

In the interval dosing study, on day 7, the maximum total HMG-CoA reductase inhibitory activity was

Expert Opin. Investig. Drugs Downloaded from informahealthcare.com by University of Southern California on 04/05/14
For personal use only.

RIGHTSLINK

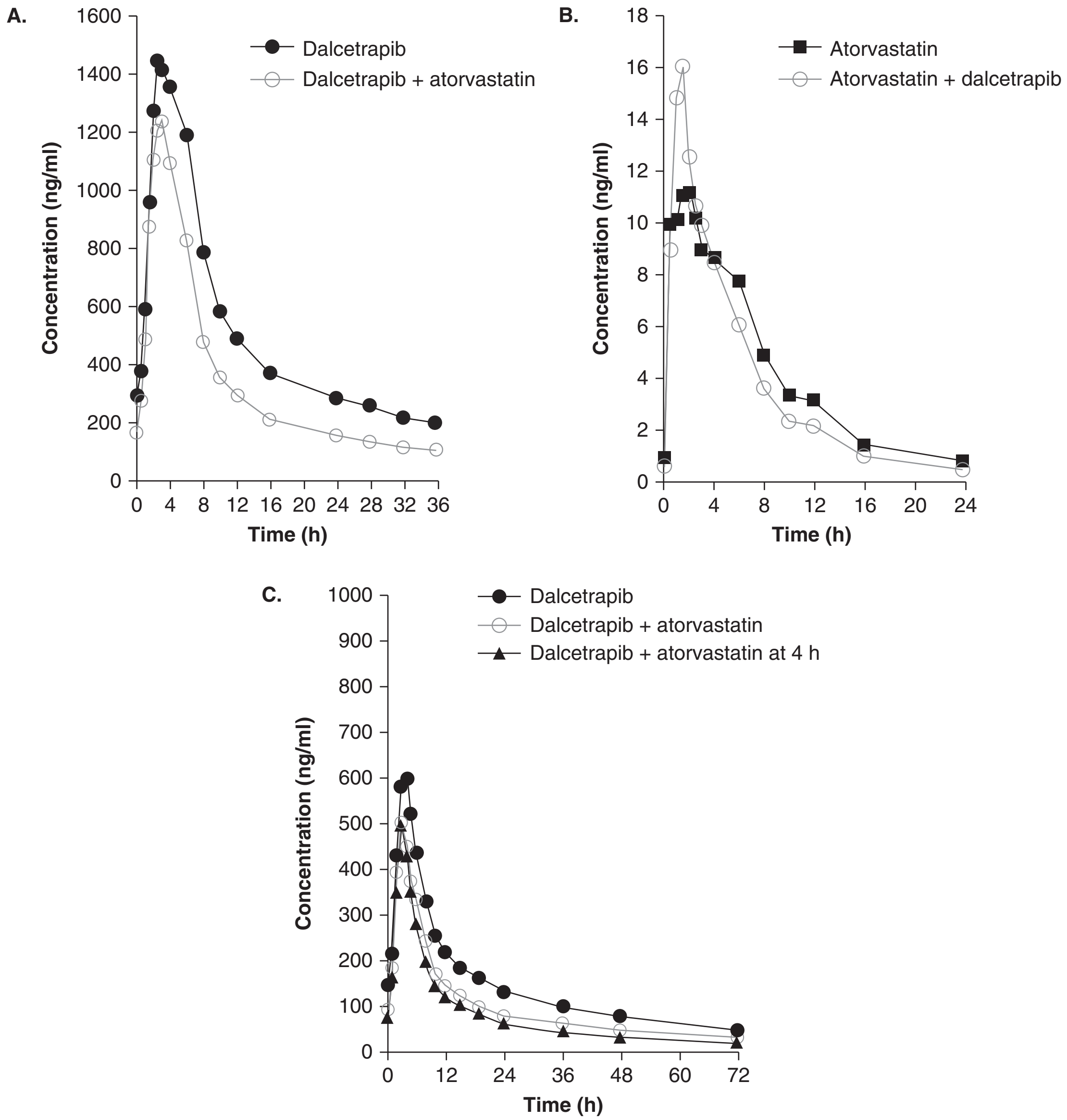

Figure 2. Plasma concentration-time profiles for dalcetrapib and atorvastatin. A. Mean dalcetrapib plasma concentrations following administration of dalcetrapib 900 mg alone or in combination with atorvastatin administered concurrently. **B.** Mean atorvastatin plasma concentrations following administration of atorvastatin alone or in combination with dalcetrapib 900 mg. **C.** Mean dalcetrapib plasma concentrations following administration of dalcetrapib 600 mg alone, in combination with atorvastatin administered concurrently, and in combination with atorvastatin administered serially (4 h later).

observed 6 h after administration of dalcetrapib for both concurrent and serial administration of atorvastatin; 33.5 ± 12.6 and 33.1 ± 14.3 ng Eq/ml, respectively, compared with day 7 pre-dose levels of 11.7 ± 5.3 and 16.4 ± 7.9 ng Eq/ml, respectively. Maximum active HMG-CoA reductase inhibitory activity was also similar between the concurrent and serial groups at 6 h after dalcetrapib; 27.4 ± 12.6 and 28.9 ± 16.2 ng Eq/ml, respectively, with day 7 pre-dose levels of 8.5 ± 3.9 and 13.0 ± 7.7 ng Eq/ml, respectively. Treatment with dalcetrapib alone appeared to have no effect on total and active HMG-CoA reductase inhibitory activity measured at 6 h post dose; 0.31 ± 0.25 and 0.29 ± 0.18 ng Eq/ml, respectively, compared with pre-dose day 7 levels of 0.32 ± 0.26 and 0.37 ± 0.49 ng Eq/ml, respectively.

There was little change in the activity of PON1 in any of the treatment groups from baseline (pre-dose day 1) to day 8 (24 h following the day 7 dose), with increases of approximately 5% in the groups receiving dalcetrapib or dalcetrapib co-administered with atorvastatin concurrently, and a decrease of approximately 5% in the group receiving atorvastatin 4 h after dalcetrapib.

Expert Opin. Investig. Drugs Downloaded from informahealthcare.com by University of Southern California on 04/05/14
For personal use only.

Table 2. Effect of concurrent co-administration of dalcetrapib 900 mg with atorvastatin on the pharmacokinetic parameters of dalcetrapib and atorvastatin and their metabolites.

Analyte/parameter	Administered concurrently	Administered alone	Geometric mean ratio	90% CI
Dalcetrapib				
AUC_{0-24} (h*ng/ml)	10,600 ± 3220	15,400 ± 4580	0.699	0.655, 0.747
C_{max} (ng/ml)	1450 ± 396	1720 ± 436	0.852	0.779, 0.930
Dalcetrapib-S-Glu				
AUC_{0-24} (h*ng/ml)	1380 ± 917	1350 ± 981	1.088	0.967, 1.225
C_{max} (ng/ml)	540 ± 462	463 ± 294	1.027	0.904, 1.167
Dalcetrapib-S-Me				
AUC_{0-24} (h*ng/ml)	3070 ± 712	3070 ± 691	0.991	0.937, 1.048
C_{max} (ng/ml)	525 ± 143	540 ± 146	0.975	0.885, 1.074
Atorvastatin				
AUC_{0-24} (h*ng/ml)	89.6 ± 51.6	98.6 ± 57.1	0.898	0.827, 0.975
C_{max} (ng/ml)	20.3 ± 13.5	18.2 ± 17.5	1.108	0.894, 1.372
2-OH-atorvastatin				
AUC_{0-24} (h*ng/ml)	90.2 ± 37.5	97.0 ± 34.2	0.889	0.826, 0.957
C_{max} (ng/ml)	10.3 ± 4.6	11.2 ± 4.5	0.856	0.751, 0.975
4-OH-atorvastatin				
AUC_{0-24} (h*ng/ml)	16.2 ± 9.8	16.7 ± 10.3	0.948	0.867, 1.037
C_{max} (ng/ml)	1.2 ± 0.8	1.2 ± 0.8	1.020	0.889, 1.171
Atorvastatin lactone				
AUC_{0-24} (h*ng/ml)	58.0 ± 21.5	82.7 ± 33.5	0.706	0.657, 0.758
C_{max} (ng/ml)	6.4 ± 2.7	9.2 ± 4.4	0.699	0.622, 0.785
2-OH-atorvastatin-lactone				
AUC_{0-24} (h*ng/ml)	150 ± 38.7	167 ± 54.5	0.908	0.849, 0.971
C_{max} (ng/ml)	15.5 ± 4.5	17.7 ± 7.0	0.904	0.818, 0.998
4-OH-atorvastatin-lactone				
AUC_{0-24} (h*ng/ml)	43.3 ± 14.3	41.4 ± 16.2	1.081	1.010, 1.157
C_{max} (ng/ml)	4.8 ± 1.9	4.3 ± 1.8	1.159	1.003, 1.340

Values are mean ± standard deviation unless otherwise noted.
AUC_{0-24}: Area under the concentration-time curve from 0 to 24 h; CI: Confidence interval; C_{max}: Maximum observed plasma concentration; Dalcetrapib-S-Glu: Dalcetrapib-S-glucuronide; Dalcetrapib-S-Me: Dalcetrapib-S-methyl.

Expert Opin. Investig. Drugs Downloaded from informahealthcare.com by University of Southern California on 04/05/14
For personal use only.

Table 3. Effect of co-administration of dalcetrapib 600 mg with atorvastatin with concurrent or serial dosing on the pharmacokinetic parameters of dalcetrapib.

Dosing schedule	Dalcetrapib AUC_{0-24} (h*ng/ml)	Dalcetrapib C_{max} (ng/ml)
A) Dalcetrapib alone	6450 ± 2080	727 ± 318
B) Concurrent co-administration	4660 ± 1540	631 ± 293
C) Serial co-administration	4180 ± 1280	568 ± 262
Geometric mean ratio (90% CI)		
Concurrent co-administration vs dalcetrapib alone	0.726 (0.667, 0.790)	0.876 (0.761, 1.009)
Serial co-administration vs dalcetrapib alone	0.650 (0.596, 0.709)	0.801 (0.693, 0.925)
Serial vs concurrent co-administration	0.896 (0.823, 0.975)	0.914 (0.792, 1.054)

Values are mean ± standard deviation unless otherwise noted.
AUC_{0-24}: Area under the concentration-time curve from 0 to 24 h; C_{max}: Maximum observed plasma concentration.

3.4 Safety

Dalcetrapib co-administered with atorvastatin was generally well tolerated (Table 5). The most common AE reported in each study was headache. In the concurrent dosing study, headache was reported in 19% of participants taking dalcetrapib, 20% of participants taking atorvastatin, and 12% of participants taking combination treatment. In the interval dosing study, the incidence of headache was similar for the three treatment groups (13% for either dalcetrapib alone or concurrent co-administration; 12% for serial

RIGHTS LINK

Table 4. Effect of concurrent co-administration of dalcetrapib 900 mg with atorvastatin on lipids.

	Dalcetrapib 900 mg alone	Atorvastatin alone	Dalcetrapib 900 mg + atorvastatin
HDL-C, mmol/l			
Day 1	1.18 ± 0.29	1.17 ± 0.28	1.17 ± 0.28
Final day of dosing*	1.51 ± 0.32	1.09 ± 0.26	1.42 ± 0.31
LDL-C, mmol/l			
Day 1	2.75 ± 0.59	2.81 ± 0.68	2.81 ± 0.68
Final day of dosing*	2.34 ± 0.62	1.93 ± 0.66	1.07 ± 0.45
VLDL-C, mmol/l			
Day 1	0.62 ± 0.25	0.60 ± 0.21	0.60 ± 0.21
Final day of dosing*	0.60 ± 0.15	0.52 ± 0.10	0.51 ± 0.09
Total cholesterol, mmol/l			
Day 1	4.55 ± 0.68	4.58 ± 0.72	4.58 ± 0.72
Final day of dosing*	4.44 ± 0.65	3.54 ± 0.73	3.01 ± 0.56
TG, mmol/l [median (range)]			
Day 1	0.86 (0.71 – 1.09)	0.83 (0.65 – 1.12)	0.83 (0.65 – 1.12)
Final day of dosing*	0.84 (0.66 – 1.02)	0.67 (0.62 – 0.95)	0.67 (0.55 – 0.85)

*Final day of dosing = day 8 (Treatment B) for dalcetrapib 900 mg alone, day 5 (Treatment A) for atorvastatin alone, and day 13 (Treatment A) for dalcetrapib 900 mg + atorvastatin. Values are mean ± standard deviation unless otherwise stated.
HDL-C: High-density lipoprotein cholesterol; LDL-C: Low-density lipoprotein cholesterol; VLDL-C: Very low-density lipoprotein cholesterol.

Table 5. Overview of adverse events (AEs).

	Concurrent dosing study*		
	Dalcetrapib 900 mg alone	Dalcetrapib 900 mg + atorvastatin	Atorvastatin alone
n	26	25	25
Number (%) participants with ≥ 1 AE	15 (58)	16 (64)	7 (28)
Number of AEs	24	22	8
Number (%) of participants with ≥ 1 severe AE	1 (4)	0	0
Number (%) of participants with ≥ 1 treatment-related AE‡	3 (12)	6 (24)	4 (16)
Number (%) of participants withdrawn due to AEs	0	3 (12)	0
Number (%) of participants withdrawn due to treatment-related AEs‡	0	1 (4)	0
	**Interval dosing study**		
	**Dalcetrapib 600 mg alone**	**Dalcetrapib 600 mg + atorvastatin: concurrent co-administration**	**Dalcetrapib 600 mg + atorvastatin: serial co-administration**
n	47	47	43
Number (%) participants with ≥ 1 AE	18 (38)	26 (55)	20 (47)
Number of AEs	25	38	30
Number (%) of participants with ≥ 1 severe AE	0	1 (2)	1 (2)
Number (%) of participants with ≥ 1 treatment-related AE‡	11 (23)	10 (21)	9 (21)
Number (%) of participants withdrawn due to AEs	1 (2)	3 (6)	1 (2)
Number (%) of participants withdrawn due to treatment-related AEs‡	1 (2)	0	0

*A direct comparison of the three study treatments in the concurrent dosing study is not appropriate because the treatment periods were not balanced: atorvastatin alone was administered for a shorter duration than dalcetrapib alone or combination treatment.
‡Assessed as possibly or probably related to treatment.

Expert Opin. Investig. Drugs Downloaded from informahealthcare.com by University of Southern California on 04/05/14
For personal use only.

RIGHTSLINK

Expert Opin. Investig. Drugs Downloaded from informahealthcare.com by University of Southern California on 04/05/14
For personal use only.

co-administration). The majority of AEs were mild in intensity and were considered either unrelated or remotely related to treatment. There were no cases of myalgia and no serious AEs in either study.

In the concurrent dosing study, one participant who received atorvastatin alone was withdrawn due to abnormal laboratory values (elevated alanine aminotransferase, isolated elevated aspartate aminotransferase, elevated bicarbonate, and elevated potassium); these were considered possibly related to treatment by the investigator. Isolated abnormal laboratory values were also reported for two other participants in this study (one with elevated red blood cell count during combination treatment and one with elevated bicarbonate on day -1 prior to Treatment A and during combination treatment).

In the interval dosing study, although the majority of participants (79%) had at least one marked laboratory abnormality, none were considered clinically significant by the investigator. One participant had elevated levels of C-reactive protein (CRP), which was considered to be due to ongoing prostatitis and resulted in withdrawal from treatment. The geometric mean (min – max) levels of CRP on day 6 for dalcetrapib alone, dalcetrapib administered concurrently with atorvastatin, and atorvastatin administered 4 h later were 0.69 (0.1 – 9.0), 0.64 (0.1 – 3.9) and 0.55 (0.1 – 4.9) respectively, corresponding to small reductions from baseline levels of 0.75 (0.1 – 9.8), 0.72 (0.1 – 41.7) and 0.63 (0.1 – 4.9), respectively.

No clinically relevant changes in blood pressure were observed in either study. In the concurrent dosing study for dalcetrapib administered alone, the mean (SD) diastolic and systolic blood pressures (DBP/SBP) at 24 h post dose on day 8 were 68.5 (7.6) and 119.8 (8.8) mmHg, respectively, compared with 70.7 (7.2) and 122.3 (9.4) mmHg at baseline. For co-administration of dalcetrapib and atorvastatin in the concurrent dosing study, the mean (SD) DBP and SBP at 24 h post dose on day 13 were 67.1 (6.6) and 120.5 (6.9) mmHg, respectively, compared with 69.5 (10.4) and 119.2 (9.1) mmHg at baseline. In the interval dosing study, the mean (SD) DBP decreased from baseline to day 8 by -3.5 (9.3), -4.1 (8.3) and -4.6 (8.3) mmHg for dalcetrapib administered alone, concurrently with atorvastatin, and serially with atorvastatin, respectively, while the changes in mean (SD) SBP were -0.4 (11.3), +0.59 (0.2) and -2.4 (8.3) mmHg, respectively.

In the concurrent dosing study, there were no QTcB intervals > 450 ms reported for any participant, but abnormal ECG readings led to withdrawals of two participants during co-administration of atorvastatin with dalcetrapib 900 mg. One of these readings, first-degree atrioventricular block, was considered by the investigator to be remotely related to treatment, and the relationship to treatment was not assessed by the investigator for the other abnormal reading (fluctuating ECG abnormalities attributed to normal variant or Brugada syndrome). In addition, one participant was withdrawn from the interval dosing study due to mild first-degree atrioventricular block during concurrent co-administration of atorvastatin with dalcetrapib; this event was also considered remotely related to treatment by the study investigator.

4. Discussion

Co-administration of statins with other drugs may result in side effects such as myalgia and rhabdomyolysis if the exposure of statins is increased [10]. Dalcetrapib is in clinical development for the prevention of CVD events, and is likely to be prescribed concomitantly with statins. It was therefore important to determine whether co-administration of dalcetrapib with atorvastatin results in any clinically relevant pharmacokinetic interactions or effects on efficacy or safety, compared with administration of dalcetrapib or atorvastatin alone. The studies presented here did not show any safety issues associated with co-administration of atorvastatin with dalcetrapib. Most AEs were mild.

Compared with monotherapy, co-administration of atorvastatin with dalcetrapib 600 or 900 mg was associated with a decrease in exposure of dalcetrapib. Serial co-administration of atorvastatin following dalcetrapib 600 mg resulted in decreases in dalcetrapib exposure comparable with concurrent co-administration. Nonetheless, the decreases in dalcetrapib exposure observed in the concurrent dosing study did not result in apparent effects on the inhibition of CETP activity or the increase in HDL-C, suggesting that the pharmacokinetic interaction between the drugs is not clinically relevant even at the higher dalcetrapib dose. In previously reported data from Phase II trials in patients with dyslipidemia or with coronary heart disease or coronary heart disease risk equivalents, HDL-C was increased by a similar magnitude when dalcetrapib 600 or 900 mg was administered as monotherapy or in addition to background therapy with atorvastatin, pravastatin, or simvastatin [7]. Likewise, in a recent report of drug–drug interaction studies of dalcetrapib co-administered with pravastatin, rosuvastatin, and simvastatin, slight decreases in dalcetrapib exposure during co-administration of rosuvastatin and simvastatin neither abrogated the increase in HDL-C nor the decrease in CETP activity that was seen with dalcetrapib alone [11].

In the concurrent dosing study, a small (10%) decrease in exposure to atorvastatin and a small (14%) increase in exposure to its active metabolite 2-OH atorvastatin was observed during co-administration of atorvastatin and dalcetrapib 900 mg. Among the inactive metabolites, co-administration of dalcetrapib 900 mg had the largest effect on atorvastatin lactone exposure (~ 30% decrease), whereas the exposure of 4-OH-atorvastatin lactone as assessed by C_{max} increased by 16%. Exposure of 2-OH-atorvastatin lactone was not significantly altered.

The possible mechanisms underlying the observed changes in plasma exposure during co-administration of dalcetrapib

RIGHTSLINK

Expert Opin. Investig. Drugs Downloaded from informahealthcare.com by University of Southern California on 04/05/14
For personal use only.

and atorvastatin remain to be elucidated, but could possibly be related to changes in the distribution and clearance of dalcetrapib as a result of the altered lipoprotein distribution or composition caused by atorvastatin. The small changes in the exposure of atorvastatin and most of its metabolites are unlikely to be the result of a change in CYP3A4 activity, since studies have shown that dalcetrapib does not have an effect on any of the major cytochrome P450 (CYP) isoforms [12]. Another study showed no clinically significant interactions between dalcetrapib and ketoconazole, a strong CYP3A4 inhibitor [13]. Hepatic uptake mediated by organic anion transporting polypeptides (OATP) may play a role in drug–drug interactions with atorvastatin [14,15]. Inhibition of OATP-mediated uptake of atorvastatin has been implicated, for example, in the increase in atorvastatin exposure observed during co-administration with rifampicin [14]. Statins also interact with P-glycoprotein, a member of the superfamily of ATP-binding cassette transporters involved in the absorption, distribution and excretion of certain drugs [16,17]. At present the role of these transporters in the alterations in atorvastatin exposure during dalcetrapib co-administration remain speculative. Moreover, the lack of increase in atorvastatin exposure provides additional support for the expectation that co-administration of atorvastatin with dalcetrapib should be well tolerated and not associated with myalgia or rhabdomyolysis.

Although the effects of dalcetrapib on lipid parameters have been described in considerably larger and longer studies, results presented here show that even after short-term dosing, the observed improvements in the overall lipid profile (beyond the increase in HDL-C) in healthy volunteers are not dissimilar to those observed in patients with dyslipidemia or with coronary heart disease or coronary heart disease risk equivalents. In the concurrent dosing study, atorvastatin co-administered with dalcetrapib 900 mg was associated with LDL-C-lowering beyond that observed with atorvastatin alone. In larger Phase II studies of dalcetrapib in combination with atorvastatin, pravastatin, or simvastatin, reductions in LDL-C were comparable for combination treatment and statins alone [7,8,18].

Activity of PON1, an HDL-associated esterase/lactonase, was included as an exploratory end point in the interval study, as it has been suggested that changes in HDL subfraction ratios could be linked to changes in the stability and antioxidant capability of PON1 [19]. Moderate increases in PON1 activity have also been observed in studies of 3 months of treatment with atorvastatin alone [20,21]. As no difference in PON1 activity was observed in any group in the interval dosing study, it is possible that the time course of 7 days treatment may have been too short to observe an effect. Similarly, treatment with atorvastatin alone has been shown to decrease CETP activity in studies of up to 3 months [22,23]; however, the duration of atorvastatin monotherapy (5 days) in the concurrent dosing study may have been too short to observe an effect on CETP activity or mass.

In conclusion, effects on LDL-C and HDL-C, and safety parameters were comparable with co-administration of atorvastatin and dalcetrapib compared with atorvastatin or dalcetrapib alone, even though changes in exposures of dalcetrapib and atorvastatin and most of its metabolites were observed. Co-administration of atorvastatin with dalcetrapib does not appear to be associated with any clinically relevant drug–drug interactions. These results support the use of dalcetrapib alongside statin therapy as part of current evidence-based care, in the large, long-term Phase III dal-OUTCOMES study (ClinicalTrials.gov identifier: NCT00658515) [24].

Acknowledgements

Content has previously been presented as an abstract and poster at the National Lipid Association Annual Scientific Sessions in Miami, Florida, 30 April to 3 May 2009. Editorial assistance was provided by K Whitfield, Prime Healthcare Ltd.

Declaration of interest

M Derks and M Abt are employees of F. Hoffmann-La Roche Ltd, Basel, Switzerland and the remaining four authors are employees of Roche Products Ltd UK. Editorial assistance was funded by F. Hoffmann-La Roche Ltd.

RIGHTSLINK

Expert Opin. Investig. Drugs Downloaded from informahealthcare.com by University of Southern California on 04/05/14
For personal use only.

Bibliography

Papers of special note have been highlighted as either of interest (•) or of considerable interest (••) to readers.

1. Fruchart JC, Sacks F, Hermans MP, et al. The residual risk reduction initiative: a call to action to reduce residual vascular risk in patients with dyslipidemia. Am J Cardiol 2008;102(10 Suppl):1K-34K
   •• **Position paper on residual risk.**
2. Robins SJ, Collins D, Wittes JT, et al.; VA-HIT Study Group. Veterans affairs high-density lipoprotein intervention trial. Relation of gemfibrozil treatment and lipid levels with major coronary events: VA-HIT: a randomized controlled trial. JAMA 2001;285:1585-91
3. Canner PL, Furberg CD, McGovern ME. Benefits of niacin in patients with versus without the metabolic syndrome and healed myocardial infarction (from the Coronary Drug Project). Am J Cardiol 2006;97:477-9
4. Goldenberg I, Goldbourt U, Boyko V, et al.; BIP Study Group. Relation between on-treatment increments in serum high-density lipoprotein cholesterol levels and cardiac mortality in patients with coronary heart disease (from the Bezafibrate Infarction Prevention trial). Am J Cardiol 2006;97:466-71
5. Inazu A, Brown ML, Hesler CB, et al. Increased high-density lipoprotein levels caused by a common cholesteryl-ester transfer protein gene mutation. N Engl J Med 1990;323:1234-8
6. Boekholdt SM, Kuivenhoven JA, Wareham NJ, et al. Plasma levels of cholesteryl ester transfer protein and the risk of future coronary artery disease in apparently healthy men and women: the prospective EPIC (European Prospective Investigation into Cancer and nutrition)-Norfolk population study. Circulation 2004;110:1418-23
7. Stein EA, Stroes ES, Steiner G, et al. Safety and tolerability of dalcetrapib. Am J Cardiol 2009;104:82-91
   • **Dalcetrapib short-term efficacy and safety trials.**
8. Stein EA Roth EM, Rhyne JM, et al. Safety and tolerability of dalcetrapib (RO4607381/JTT-705): results from a 48-week trial. Eur Heart J 2010;31:480-8
   • **Dalcetrapib long-term efficacy and safety trial.**
9. de Grooth GJ, Kuivenhoven JA, Stalenhoef AF, et al. Efficacy and safety of a novel cholesteryl ester transfer protein inhibitor, JTT-705, in humans: a randomized phase II dose-response study. Circulation 2002;105:2159-65
   • **Dalcetrapib short-term efficacy and safety trial.**
10. Neuvonen PJ, Niemi M, Backman JT. Drug interactions with lipid-lowering drugs: mechanisms and clinical relevance. Clin Pharmacol Ther 2006;80:565-81
11. Derks M, Abt M, Phelan M, et al. Coadministration of dalcetrapib with pravastatin, rosuvastatin, or simvastatin: no clinically relevant drug–drug interactions. J Clin Pharmacol In press, DOI: 10.1177/0091270009358709
   •• **Dalcetrapib–statin interactions.**
12. Derks M, Fowler S, Kuhlmann O. In vitro and in vivo assessment of the effect of dalcetrapib on a panel of CYP substrates. Curr Med Res Opin 2009;25:891-902
   •• **Dalcetrapib–CYP interactions.**
13. Derks M, Fowler S, Kuhlmann O. A single-center, open-label, one-sequence study of dalcetrapib coadministered with ketoconazole, and an in vitro study of the S-methyl metabolite of dalcetrapib. Clin Ther 2009;31:586-99
   •• **Dalcetrapib interaction with a CYP3A4 inhibitor.**
14. Lau YY, Okochi H, Huang Y, Benet LZ. Multiple transporters affect the disposition of atorvastatin ans its two active hydroxy metabolites: application of in vitro and ex situ systems. J Pharmacol Exp Ther 2006;316:762-71
15. Shitara Y, Sugiyama Y. Pharmacokinetic and pharmacodynamic alterations of 3-hydroxy-3-methylglutaryl coenzyme A (HMG-CoA) reductase inhibitors: drug-drug interactions and interindividual differences in transporter and metabolic enzyme functions. Pharmacol Ther 2006;122:71-105
16. Bogman K, Peyer AK, Torok M, et al. HMG-CoA reductase inhibitors and P-glycoprotein modulation. Br J Pharmacol 2001;132:1183-92
17. Chen C, Lin J, Smolarek T, Tremaine L. P-glycoprotein has differential effects on the disposition of statin acid and lactone forms in mdr1a/b knockout and wild-type mice. Drug Metab Dispos 2007;35:1725-29
18. Kuivenhoven JA, de Grooth GJ, Kawamura H, et al. Effectiveness of inhibition of cholesteryl ester transfer protein by JTT-705 in combination with pravastatin in type II dyslipidemia. Am J Cardiol 2005;95:1085-8
   • **Dalcetrapib short-term efficacy and safety trial.**
19. Moren X, Deakin S, Liu M-L, et al. HDL subfraction distribution of paraoxonase-1 and its relevance to enzyme activity and resistance to oxidative stress. J Lipid Res 2008;49:1246-53
20. Mirdamadi HZ, Sztanek F, Derdak Z, et al. The human paraoxonase-1 phenotype modifies the effect of statins on paraoxonase activity and lipid parameters. Br J Clin Pharmacol 2008;66:366-74
21. Nagila A, Permpongpaiboon T, Tantrarongroj S, et al. Effect of atorvastatin on paraoxonase1 (PON1) and oxidative status. Pharmacol Rep 2009;61:892-8
22. Guerin M, Lassel TS, Le Goff W, et al. Action of atorvastatin in combined hyperlipidemia: preferential reduction of cholesteryl ester transfer from HDL to VLDL1 particles.

RIGHTSLINK

Arterioscler Thromb Vasc Biol 2000;20:189-97

23. Kassai A, Illyes L, Mirdamadi HZ, et al. The effect of atorvastatin therapy on lecithin: cholesterol acyltransferase, cholesteryl ester transfer protein and the antioxidant paraoxonase. Clin Biochem 2007;40:1-5

24. Schwartz GG, Olsson AG, Ballantyne CM, et al. Rationale and design of the dal-OUTCOMES trial: efficacy and safety of dalcetrapib in patients with recent acute coronary syndrome. Am Heart J 2009;158:896-901
• **Dalcetrapib end point trial design.**

Affiliation

Michael Derks[†1], Markus Abt[2], Graeme Parr[3], Georgina Meneses-Lorente[4], Anne-Marie Young[4] & Mary Phelan[4]
[†]Author for correspondence
[1]F. Hoffmann-La Roche Ltd, Department of Clinical Pharmacology, Bldg. 663, Hochstrasse 16, CH-4070 Basel, Switzerland
Tel: +41 61 68 79584; Fax: +41 61 68 86007; E-mail: michael.derks@roche.com
[2]F. Hoffmann-La Roche Ltd, Statistics, Early Clinical Development, Basel, Switzerland
[3]Roche Products Ltd, Clinical Research and Exploratory Development-Study Management, Welwyn Garden City, UK
[4]Roche Products Ltd, Department of Clinical Pharmacology, Welwyn Garden City, UK

Supplementary material available online

Supplementary Table 1.

Expert Opin. Investig. Drugs Downloaded from informahealthcare.com by University of Southern California on 04/05/14
For personal use only.
