# Supplementary material for: Lipid-lowering effect of combined therapy with high-intensity statins and CETP inhibitors: a Systematic Review and meta-analysis
Source: Front Endocrinol (Lausanne). 2025 May 1;16:1512670. doi: 10.3389/fendo.2025.1512670 (PMC12078159; doi:10.3389/fendo.2025.1512670)
Supplement: Supplementary file 1 [file DataSheet1.zip › Raw Data/Raw Data/Original Documentation Fruit/Barter2012✔.pdf]

Relationship between atorvastatin dose and the harm caused by torcetrapib

Philip J. Barter,[1,]* Kerry-Anne Rye,* Mohan S. Beltangady,[†] William C. Ports,[†] William T. Duggan,[†] S. Matthijs Boekholdt,[§] David A. DeMicco,[†] John J. P. Kastelein,[§] and Charles L. Shear[2,†]

The Heart Research Institute,* Sydney, Australia; Pfizer, Inc.[†]; and Academic Medical Center,[§] University of Amsterdam, The Netherlands

Abstract Development of the cholesteryl ester transfer protein (CETP) inhibitor, torcetrapib, was halted after the ILLUMINATE trial revealed an increase in both all-cause mortality (ACM) and major cardiovascular events (MCVEs) associated with its use. We now report that the harm caused by torcetrapib was confined to those in the 10 mg atorvastatin subgroup for both ACM [hazard ratio (HR) = 2.68, 95% CI (1.58, 4.54), *P* < 0.0001] and MCVEs [HR = 1.41, 95% CI (1.14, 1.74), *P* = 0.002], with no evidence of harm when torcetrapib was coadministered with higher doses of atorvastatin. In the atorvastatin 10 mg subgroup, age, prior heart failure and stroke were significantly associated with ACM, independent of torcetrapib treatment, whereas low apoA-I, smoking, hypertension, heart failure, myocardial infarction, and stroke were independently associated with MCVEs. After adjusting for these factors, the HR associated with torcetrapib treatment in the 10 mg atorvastatin subgroup remained elevated for both ACM [HR = 2.67, 95% CI (1.57, 4.54), *P* < 0.001] and MCVE [HR = 1.36, 95% CI (1.10, 1.69), *P* = 0.005]. Thus, the harm caused by torcetrapib was confined to individuals taking atorvastatin 10 mg. The harm could not be explained by torcetrapib-induced changes in lipid levels, blood pressure, or electrolytes. It is conceivable that higher doses of atorvastatin protected against the harm caused by torcetrapib.—Barter, P. J., K-A. Rye, M. S. Beltangady, W. C. Ports, W. T. Duggan, S. M. Boekholdt, D. A. DeMicco, J. J. P. Kastelein, and C. L. Shear. **Relationship between atorvastatin dose and the harm caused by torcetrapib. *J. Lipid Res.* 2012.** 53: **2436–2442.**

Supplementary key words atherosclerosis • cardiovascular diseases • cholesteryl ester transfer protein • cholesterol • drugs • lipoproteins

The Investigation of Lipid Level management to Understand its iMpact IN ATherosclerotic Events (ILLUMINATE) trial was designed to test the hypothesis that torcetrapib, the first cholesteryl ester transfer protein (CETP) inhibitor to be tested in a clinical outcome trial, would decrease the risk of cardiovascular events in subjects with coronary heart disease or risk equivalents (1). Quite unexpectedly, the trial was terminated prematurely for safety reasons. The primary results have been described previously (1). Briefly, torcetrapib 60 mg/day on a background of atorvastatin 10, 20, 40, or 80 mg/day, raised HDL-cholesterol (HDL-C) and lowered LDL-cholesterol (LDL-C), but also resulted in a significantly increased risk of both all-cause mortality (ACM) [hazard ratio (HR) = 1.58, 95% CI (1.14, 2.19), *P* = 0.006] and major cardiovascular events (MCVEs) [HR = 1.25, 95% CI (1.09, 1.44), *P* = 0.001].

Observed adverse effects of torcetrapib included an increase in both systolic blood pressure (SBP) and diastolic blood pressure (DBP), an increase in the levels of sodium, bicarbonate, and aldosterone, and a decrease in the level of potassium (1). Subsequent preclinical studies have shown that torcetrapib increases the synthesis and secretion of aldosterone and cortisol from cultured adrenal cortical cells (2, 3), has an adverse effect on endothelial function, and increases expression of endothelin-1 in the artery wall (4). These adverse effects of torcetrapib have been shown to be unrelated to inhibition of CETP activity (2, 3).

We now report a post hoc exploratory analysis of the ILLUMINATE trial in an attempt to determine potential predictors of ACM and the occurrence of MCVEs.

METHODS

ILLUMINATE was a prospective, randomized, multi-center, double-blind clinical trial as previously described (1). Briefly,

Abbreviations: ACM, all-cause mortality; BMI, body mass index; CETP, cholesteryl ester transfer protein; CHF, congestive heart failure; CRP, C-reactive protein; DBP, diastolic blood pressure; HDL-C, HDL-cholesterol; HR, hazard ratio; LDL-C, LDL-cholesterol; LLQ, lower limit of quantification; MCVE, major cardiovascular event; SBP, systolic blood pressure.

[1] To whom correspondence should be addressed.
e-mail: barterp@hri.org.au
[2] Currently employed by Commonwealth Serum Laboratory, Ltd.

Manuscript received 13 March 2012 and in revised form 2 September 2012.

Published, JLR Papers in Press, September 2, 2012
DOI 10.1194/jlr.P026328

Copyright © 2012 by the American Society for Biochemistry and Molecular Biology, Inc.

This article is available online at http://www.jlr.org

This is an Open Access article under the CC BY license.

men and women between the ages of 45 and 75 years were eligible to participate in the trial if they had a history of cardiovascular disease (including myocardial infarction, stroke, acute coronary syndrome, unstable angina, peripheral vascular disease, or cardiac revascularization) 30 days to 5 years before screening. Subjects with type 2 diabetes were also eligible. Exclusion criteria included evidence of an unstable medical condition, life expectancy of less than 5 years, or an LDL-C level of less than 100 mg/dl (2.6 mmol/l) if the subject was not receiving a lipid-lowering drug. Written informed consent was obtained from the subjects who agreed to participate.

During a run-in period of 4 to 10 weeks, patients underwent lifestyle counseling and also received atorvastatin at a dose titrated at 2-week intervals to achieve an LDL-C concentration of less than 100 mg/dl. Subjects were excluded at the end of the run-in if they had had a cardiovascular event during the run-in period or if they had uncontrolled hypertension (defined as SBP >140 mm Hg or DBP >90 mm Hg), or if the LDL-C target level had not been reached at the end of the run-in period. Eligible subjects were then randomly assigned to one of two treatment arms: torcetrapib 60 mg or its matching placebo added to their atorvastatin dose established during the run-in period (10, 20, 40, or 80 mg/day). All baseline values presented in this paper are those collected at the time of randomization. It should be noted, however, that most of the participants were already taking statins at the time of recruitment into the trial and, by design, all were on atorvastatin at the time of randomization. Thus, baseline values do not represent values prior to taking statins.

Study outcomes

The primary outcome was time to the first occurrence of an MCVE, a composite that included four components: death from coronary heart disease (defined as fatal myocardial infarction excluding procedure-related events, fatal heart failure, sudden cardiac death, or other cardiac death), nonfatal myocardial infarction (excluding procedure-related events), stroke, and hospitalization for unstable angina. The various methodologies employed in the measurement of blood pressure, aldosterone, lipids, and lipoproteins have been previously described (1).

Statistical analysis

All treatment comparisons were performed with the use of an intention-to-treat analysis. All data were censored for the primary analyses on December 2, 2006, when the trial was prematurely terminated. Multiple regression analysis using the Cox proportional hazards model was employed to screen those baseline covariates related to ACM and MCVEs. A forward stepwise selection procedure (entry at $P < 0.10$ and exit at $P > 0.15$) was used to select the covariates among the baseline factors considered to be of prognostic importance while retaining treatment group in the model. The baseline factors considered included age; body mass index (BMI); gender; smoking status; weight; levels of apoA-I, apoB, HDL-C, LDL-C, triglyceride, serum bicarbonate, C-reactive protein (CRP), potassium, and sodium; SBP; and a history of congestive heart failure (CHF), hypertension, myocardial infarction, stroke, and type 2 diabetes. The final models were derived using those factors that were deemed significant based on the stepwise selection process.

The treatment effect on observed month 3 biomarker values (e.g., lipids, apolipoproteins, and electrolytes) was examined using an ANCOVA model that included terms for treatment group (placebo or torcetrapib 60 mg), baseline biomarker value, background atorvastatin dose, age category (⩽60, 61 to 70, >70) and an interaction term for treatment group and atorvastatin dose. The least-squares means estimates were obtained and examined for the final model for each biomarker.

To determine the potential for loss of information bias in these later analyses, we examined characteristics of subjects at baseline and those with available information at month 3.

As previously described for the post hoc measurements of aldosterone (1), most analyzed samples had aldosterone levels below the lower limit of quantification (LLQ; <4 ng/dl for samples with sufficient volume and <8 ng/dl for samples with insufficient volume requiring dilution). Baseline levels of aldosterone were not included in the forward stepwise regression procedure for ACM or MCVEs due to missing aldosterone values. Month 3 values were used in the ANCOVA model described above; values reported at the LLQ were imputed to a value half of the LLQ for statistical analyses. All analyses were performed with SAS 8.2 (SAS Institute, Inc.; Cary, NC).

RESULTS

ACM

Of the 34 excess deaths in the torcetrapib group (93 vs. 59 for torcetrapib vs. placebo, respectively), 31 (91%) occurred in the 10 mg atorvastatin dose subgroup (50 vs. 19 for torcetrapib vs. placebo, respectively) for an HR of 2.68 (95% CI, 1.58 to 4.54; $P < 0.001$). No other atorvastatin dose subgroup had an elevated risk (**Fig. 1**). The torcetrapib plus 10 mg atorvastatin subgroup, as compared with the atorvastatin 10 mg-alone subgroup, had an increased risk of death from both cardiovascular causes (30 vs. 13, $P < 0.008$) and noncardiovascular causes (19 vs. 6, $P < 0.008$) (**Table 1**).

MCVEs

Of the 91 excess MCVEs in the torcetrapib treatment group (464 vs. 373 for torcetrapib vs. placebo, respectively), 55 (60%) were in the atorvastatin 10 mg subgroup (202 vs. 147 for torcetrapib vs. placebo, respectively) for an HR of 1.41 (95% CI, 1.14 to 1.74; $P = 0.002$) (**Fig. 2**).

Because the torcetrapib plus atorvastatin 10 mg subgroup was the only atorvastatin dose subgroup with either a trend toward or a statistically significant increase in ACM and MCVEs, this subgroup provided an opportunity for further exploration of possible factors responsible for the harm. Whereas the randomization was not stratified by atorvastatin dose, baseline demographic and clinical characteristics of the two treatment groups in the atorvastatin

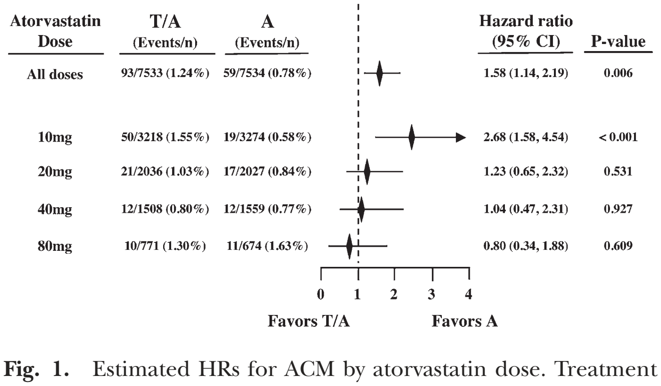

Fig. 1. Estimated HRs for ACM by atorvastatin dose. Treatment groups: T/A indicates torcetrapib treatment on background atorvastatin; A indicates atorvastatin background treatment alone.

TABLE 1. Cause of death by treatment group and atorvastatin dose

	Atorvastatin dose							
	10 mg		20 mg		40 mg		80 mg	
Number of subjects	T/A 3,218	A 3,274	T/A 2,036	A 2,027	T/A 1,508	A 1,559	T/A 771	A 674
Event	Number of events							
All Deaths	50	19	21	17	12	12	10	11
Any CV cause	30	13	11	9	4	6	4	7
Sudden death	15	8	5	7	3	4	3	6
Fatal MI (not procedure related)	6	2	2	2	0	1	0	1
Fatal stroke	3	0	2	0	1	0	0	0
Hemorrhagic	1	0	2	0	1	0	0	0
Ischemic	2	0	0	0	0	0	0	0
Embolic	0	0	0	0	0	0	0	0
Fatal heart failure	2	0	0	0	0	1	0	0
Other vascular cause	1	2	0	0	0	0	0	0
Fatal MI (procedure related)	2	0	0	0	0	0	0	0
Other cardiac cause	1	1	2	0	0	0	1	0
Any non-CV cause	19	6	9	6	6	4	6	4
Cancer	12	5	4	3	4	4	4	2
Infection	3	0	3	0	2	0	1	0
Trauma	1	1	1	2	0	0	1	0
Suicide or homicide	0	0	0	1	0	0	0	0
Other cause	3	0	1	0	0	0	0	2
Reason unknown	1	0	1	2	2	2	0	0

T/A: torcetrapib plus atorvastatin; A atorvastatin alone; CV: cardiovascular; MI: myocardial infarction.

10 mg subgroup were balanced (**Table 2**), indicating that the hazards presented above were not the result of identifiable selection bias. Although this poses some limitations, the comparisons done were still between randomized treatment groups.

Baseline predictors of ACM in the atorvastatin 10 mg subgroup

Results from the forward stepwise regression procedure for ACM are shown in **Table 3**. Independent of treatment group, the presence at baseline of increased age, a history of CHF or stroke, or an increased CRP level were associated with an increased risk of death due to any cause in the atorvastatin 10 mg subgroup. The strongest baseline predictor of death was age >70 years. After accounting for these baseline predictors, the adjusted HR for subjects treated with torcetrapib versus placebo in the atorvastatin 10 mg subgroup was 2.67 ($P < 0.001$). Baseline factors that were not predictive of ACM ($P > 0.10$) were gender; smoking status; history of diabetes or myocardial infarction; BMI; weight; and serum levels of sodium, potassium, bicarbonate, triglycerides, HDL-C, LDL-C, apoA-I, and apoB.

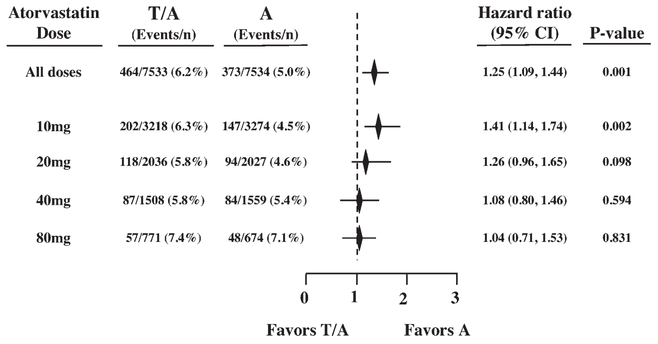

Fig. 2. Estimated HRs for major cardiovascular events by atorvastatin dose subgroup. Treatment groups: T/A indicates torcetrapib treatment on background atorvastatin; A indicates atorvastatin background treatment alone.

Baseline predictors of MCVEs in the atorvastatin 10 mg subgroup

Results from the forward stepwise regression procedure for MCVEs are shown in **Table 4**. Independent of treatment group, baseline factors associated with the occurrence of MCVEs in the atorvastatin 10 mg subgroup included an inverse association with apoA-I level and positive association with smoking status; and history of CHF, hypertension, myocardial infarction, and stroke. After accounting for these baseline predictors, the adjusted HR for subjects treated with torcetrapib versus placebo in the atorvastatin 10 mg subgroup was 1.36 ($P = 0.005$). Baseline factors that were not associated with the occurrence of an MCVE ($P > 0.10$) included gender; age; history of diabetes; BMI; weight; and blood levels of CRP, sodium, potassium, bicarbonate, triglycerides, HDL-C, LDL-C, and apoB.

Measurements at 3 months postrandomization

The torcetrapib-induced changes between baseline and month 3 in levels of LDL-C, HDL-C, SBP , sodium, potassium, bicarbonate and CRP were analyzed for heterogeneity across the four atorvastatin dose subgroups (**Table 5**). This was done to determine whether differences between the changes in the atorvastatin 10 mg subgroup and those in the other atorvastatin subgroups could account for the harm being confined to subjects taking the 10 mg dose of atorvastatin. There was no heterogeneity in the torcetrapib-induced change across the different atorvastatin subgroups in any of the measured parameters, with the exception of HDL-C and apoA-I. However, the magnitude of the differences in the torcetrapib-induced increases in HDL-C and apoA-I between the subgroups was very small

TABLE 2. Baseline characteristics of patients by atorvastatin subgroup

Variable	10 mg		20 mg		40 mg		80 mg	
	T/A N = 3,218	AN = 3,274	T/A N = 2,036	AN = 2,027	T/A N = 1,508	AN = 1,559	T/A N = 771	AN = 674
Male sex, no. (%)	2,489/3,218 (77.3)	2,564/3,274 (78.3)	1,579/2036 (77.6)	1,551/2,027 (76.5)	1,174/1,508 (77.9)	1,212/1, 559 (77.7)	612/771 (79.4)	534/674 (79.2)
White race, no. (%)	2,975/3,217 (92.5)	3,036/3,274 (92.7)	1,888/2,036 (92.7)	1,901/2,027 (93.8)	1,433/1,508 (95.0)	1,469/1, 559 (94.2)	723/771 (93.8)	622/674 (92.3)
Age (yrs)	62.0 ± 7.6	62.0 ± 7.5	61.3 ± 7.5	61.1 ± 7.6	60.6 ± 7.6	60.4 ± 7.6	59.8 ± 7.6	60.4 ± 7.5
Weight (kg)	89.3 ± 17.9	89.1 ± 17.7	90.3 ± 18.7	90.8 ± 18.2	89.3 ± 17.7	90.1 ± 17.2	89.8 ± 17.9	89.6 ± 17.7
BMI (kg/m^2)	30.1 ± 5.7	30.0 ± 5.6	30.4 ± 5.9	30.6 ± 5.9	30.0 ± 5.6	30.2 ± 5.4	30.2 ± 5.5	30.1 ± 5.2
Smoking history, no. (%)								
Current smoker	406/3,218 (12.6)	409/3,274 (12.5)	275/2,036 (13.5)	308/2,027 (15.2)	214/1,508 (14.2)	230/1,559 (14.8)	116/771 (15.0)	100/674 (14.8)
Former smoker	1,753/3,218 (54.5)	1,779/3,274 (54.3)	1,142/2,036 (56.1)	1,088/2,027 (53.7)	855/1,508 (56.7)	859/1,559 (55.1)	417/771 (54.1)	397/674 (58.9)
Nonsmoker	1,059/3,218 (32.9)	1,086/3,274 (33.2)	619/2,036 (30.4)	631/2,027 (31.1)	439/1,508 (29.1)	470/1,559 (30.1)	238/771 (30.9)	177/674 (26.3)
Medical History, no./total no. (%)								
Hypertension	2,254/3,204 (70.3)	2,403/3,267 (73.6)	1,499/2,029 (73.9)	1,511/2,022 (74.7)	1,087/1,503 (72.3)	1,138/1,553 (73.3)	583/768 (75.9)	502/673 (74.6)
Coronary revascularization	2,051/3,218 (63.7)	2,076/3,274 (63.4)	1,355/2,036 (66.6)	1,320/2,027 (65.1)	1,151/1,508 (76.3)	1,186/1,559 (76.1)	631/771 (81.8)	551/674 (81.8)
MyocardialiInfarction	1,432/3,194 (44.8)	1,432/3,252 (44.0)	870/2,020 (43.1)	853/2,007 (42.5)	724/1,491 (48.6)	741/1,545 (48.0)	424/758 (55.9)	362/668 (54.2)
Angina	1,852/3,200 (57.9)	1,814/3,261 (55.6)	1,191/2,026 (58.8)	1,194/2,016 (59.2)	1,009/1,507 (67.0)	1,025/1,554 (66.0)	529/766 (69.1)	464/668 (69.5)
Diabetes	1,496/3,212 (46.6)	1,583/3,259 (48.6)	913/2,032 (44.9)	971/2,020 (48.1)	588/1,504 (39.1)	598/1,554 (38.5)	274/769 (35.6)	238/671 (35.5)
Peripheral vascular disease	351/3,176 (11.1)	350/3,222 (10.9)	263/2,008 (13.1)	251/1,998 (12.6)	202/1,484 (13.6)	224/1,530 (14.6)	114/758 (15.0)	119/667 (17.8)
Congestive heart failure (class I or II)	203/3,207 (6.3)	220/3,257 (6.8)	137/2,024 (6.8)	145/2,016 (7.2)	100/1,498 (6.7)	101/1,547 (6.5)	64/765 (8.4)	57/669 (8.5)
Stroke	175/3,212 (5.4)	191/3,269 (5.8)	109/2,032 (5.4)	104/2,019 (5.2)	77/1,501 (5.1)	79/1,553 (5.1)	33/767 (4.3)	37/672 (5.5)
Transient ischemic attack	126/3,200 (3.9)	161/3,250 (5.0)	80/2,027 (3.9)	129/2,009 (6.4)	66/1,499 (4.4)	78/1,547 (5.0)	39/764 (5.1)	37/671 (5.5)
Lipids								
Cholesterol, mg/dl								
Total	155.4 ± 25.6	156.0 ± 26.0	157.7 ± 26.4	158.9 ± 26.7	158.3 ± 26.9	157.4 ± 27.8	157.3 ± 30.1	158.2 ± 29.6
HDL	48.8 ± 12.5	48.7 ± 12.7	48.5 ± 11.9	48.4 ± 11.8	48.9 ± 11.8	48.4 ± 12.1	47.2 ± 10.6	47.9 ± 11.5
LDL	78.5 ± 20.3	78.7 ± 20.2	80.0 ± 20.2	80.5 ± 21.1	80.1 ± 21.0	79.7 ± 21.4	80.9 ± 22.7	81.5 ± 22.3
Triglyceride, mg/dl								
Median	123.0	125.0	132.0	130.0	131.0	129.0	129.5	
Interquartile range	90.0, 174.0	91.0, 178.0	94.0, 181.0	95.0, 185.0	94.0, 179.0	94.0, 175.0	94.0, 182.0	97.0, 178.0
ApoA-I	129.3 ± 23.7	129.2 ± 23.6	128.6 ± 23.3	128.4 ± 22.9	127.7 ± 22.8	127.3 ± 22.9	123.1 ± 21.4	125.7 ± 22.8
ApoB	71.7 ± 15.2	72.0 ± 15.2	74.3 ± 15.6	74.7 ± 15.7	74.2 ± 16.2	74.2 ± 16.5	74.2 ± 17.3	75.0 ± 17.1
Vital signs								
BP, mm Hg								
Systolic	123.2 ± 11.0	123.6 ± 10.8	123.0 ± 10.9	123.0 ± 10.9	122.8 ± 10.6	122.4 ± 11.0	121.7 ± 11.0	121.5 ± 11.2
Diastolic	73.8 ± 7.6	74.0 ± 7.4	73.6 ± 7.6	74.0 ± 7.6	74.1 ± 7.6	73.9 ± 7.4	73.1 ± 7.7	72.6 ± 7.9
Heart rate, beats/min	65.5 ± 9.9	65.4 ± 9.7	65.5 ± 9.4	65.5 ± 9.5	65.2 ± 9.4	65.4 ± 9.3	64.8 ± 8.9	65.2 ± 9.1
Electrolytes, mmol/l								
Potassium	4.4 ± 0.4	4.4 ± 0.4	4.4 ± 0.4	4.4 ± 0.4	4.4 ± 0.4	4.4 ± 0.4	4.4 ± 0.4	4.4 ± 0.4
Sodium	140.4 ± 2.8	140.3 ± 2.7	140.3 ± 2.7	140.4 ± 2.8	140.4 ± 2.6	140.4 ± 2.8	140.5 ± 2.8	140.6 ± 2.7
Chloride	102.7 ± 2.9	102.6 ± 2.9	102.8 ± 2.9	102.7 ± 2.9	102.7 ± 2.9	102.9 ± 2.9	103.1 ± 2.9	103.1 ± 3.0
Bicarbonate	24.3 ± 3.1	24.4 ± 3.2	24.2 ± 3.0	24.3 ± 3.0	24.5 ± 2.9	24.3 ± 3.0	24.3 ± 2.8	24.3 ± 2.9
Renal function								
Creatinine, mg/dl	1.00 ± 0.22	1.00 ± 0.22	1.01 ± 0.22	1.00 ± 0.21	1.00 ± 0.21	1.01 ± 0.22	1.01 ± 0.22	1.01 ± 0.21
Estimated glomerular filtration rate, ml/min/1.73m[32]	85.5 ± 22.2	85.3 ± 21.3	84.8 ± 20.8	85.4 ± 20.7	86.0 ± 21.7	85.1 ± 20.7	85.3 ± 20.6	84.7 ± 20.4
Other selected measures								
CRP, mg/l								
Median	1.4	1.5	1.3	1.3	1.2	1.2	1.2	1.2
Interquartile range	0.7, 3.1	0.8, 3.3	0.6, 2.8	0.7, 2.9	0.6, 2.5	0.6, 2.8	0.6, 2.5	0.6, 2.5

Baseline clinical characteristics were determined following a run-in period of 4 to 10 weeks during which patients underwent lifestyle counseling and also received atorvastatin, titrated if needed, at 2 week intervals to achieve an LDL-C of less than 100 mg per deciliter. Plus-minus values indicate means ± SD. Percentages are based on nonmissing values in that randomized treatment group. Median triglycerides and CRP presented instead of mean due to skew of the data.

TABLE 3. Baseline predictors of ACM from the Cox Proportional Hazards Model in the atorvastatin 10 mg group

Baseline factors	H (*P* value)	95% Confidence interval
Treatment group (T/A versus A)	2.67 (<0.001)	(1.57, 4.54)
Age >70 years[a]	3.51 (<0.001)	(1.76, 6.98)
Age 61–70[a]	2.20 (0.014)	(1.17, 4.12)
History of CHF	3.20 (< 0.001)	(1.80, 5.69)
History of stroke	2.76 (0.002)	(1.46, 5.20)
CRP > 7.5 mg/l[b]	2.67 (0.004)	(1.36, 5.23)
CRP >3–7.5 mg/l[b]	1.81 (0.038)	(1.03, 3.17)

T/A, torcetrapib plus atorvastatin; A, atorvastatin alone; CHF, congestive heart failure; CRP, C reactive protein. A treatment hazard ratio >1 indicates increased risk in the T/A treatment group compared with the A treatment group.

[a] Relative to age ≤60.

[b] Relative to CRP ≤3 mg/l.

(30 mg/dl in the 10 mg group vs. 28 mg/dl in the other groups for HDL-C and 32 mg/dl in the 10 mg group vs. 28 mg/dl in the other groups for apoA-I) (4). There was no evidence that the slightly higher HDL-C and apoA-I levels after 3 months of treatment conferred additional risk of death in the atorvastatin 10 mg dose subgroup, as the levels at month 3 and changes from baseline to month 3 were identical for those who were alive or deceased at trial termination.

Impact of potential loss of information in the analyses from month 3

Of the 69 deaths that occurred in the atorvastatin 10 mg dose subgroup (torcetrapib, 50; placebo, 19), 64 deaths (torcetrapib, 47; placebo, 17) occurred after the 3 month time point. A comparison of the two treatment groups' baseline demographic and clinical characteristics in the atorvastatin 10 mg dose subgroup showed similar relationships when comparing all deaths and deaths occurring after month 3 (data not shown).

Similarly, of the 349 MCVEs occurring in the atorvastatin 10 mg dose subgroup (torcetrapib, 202; placebo, 147), 280 MCVEs (torcetrapib, 161; placebo, 119) occurred after month 3. Baseline demographic and clinical characteristics in the atorvastatin 10 mg dose subgroup showed similar relationships when comparing all MCVEs and MCVEs occurring after month 3 (data not shown).

Baseline levels of aldosterone were not included in the forward stepwise regression procedure due to missing aldosterone values, which would have excluded 13 of 50 (26%) torcetrapib and 9 of 19 (47%) placebo ACM events, and 34 of 202 (17%) torcetrapib and 32 of 147 (22%) placebo MCVEs.

TABLE 4. Baseline predictors of major cardiovascular events from the Cox Proportional Hazards Model in the atorvastatin 10 mg group

Baseline factors	HR (*P* value)	95% confidence Interval
Treatment group (T/A versus A)	1.36 (0.005)	(1.10, 1.69)
ApoA-I > 142 mg/dl	0.76 (0.043)	(0.58, 0.99)
History of CHF	1.55 (0.012)	(1.10, 2.18)
History of hypertension	1.46 (0.005)	(1.12, 1.89)
History of MI	1.41 (0.002)	(1.13, 1.75)
Smoker at baseline	1.35 (0.015)	(1.06, 1.73)
History of prior stroke	1.57 (0.017)	(1.08, 2.28)

T/A, torcetrapib plus atorvastatin; A, atorvastatin alone; apoA-I, apolipoprotein A-1; CHF, congestive heart failure; MI, myocardial infarction. A treatment hazard ratio >1 indicates increased risk in the T/A treatment group compared with the A treatment group.

DISCUSSION

The main finding from this post hoc analysis was that the torcetrapib-induced increase in ACM and MCVEs observed in the ILLUMINATE trial was confined to the participants taking 10 mg atorvastatin, with no evidence of harm in those taking higher doses of atorvastatin.

It is possible that the 10 mg atorvastatin group was different from other groups. This is not supported by the values in Table 2 showing no differences between atorvastatin dose subgroups for any parameter measured. However, it should be noted that these baseline values were collected at the time of randomization, when all participants were, by design, taking atorvastatin. Indeed, most of the participants had already been taking statins for some time before recruitment into the trial, making it impossible to obtain true baseline values collected prior to commencing statin therapy. However, patients who achieved LDL-C targets with a daily dose of only 10 mg atorvastatin most likely had lower prestatin levels of LDL-C than those needing higher doses of atorvastatin to achieve the target. Thus, we cannot exclude the possibility that those with lower prestatin levels of LDL-C were more susceptible to the harm caused by torcetrapib.

It is recognized that any post hoc analysis must be viewed with caution and could be spurious. Nevertheless, the apparent trend of decreasing risk with increasing atorvastatin dose for both endpoints, combined with a lack of imbalance in other predictors that would explain the result, is consistent with the proposition that higher doses of atorvastatin may have protected against the adverse effects of torcetrapib via properties of the drug unrelated to the lowering of LDL-C (5). These results do not support the proposition, suggested by others (6), that addition of higher doses of atorvastatin may have interacted adversely with torcetrapib, because in this case, the opposite relationship with atorvastatin dose would have been observed. Nor does the analysis support the proposition that the adverse effects of torcetrapib were the consequence of the observed changes in blood pressure, concentrations of plasma lipoproteins, electrolytes, or CRP. Rather, the analysis is consistent with the proposition that the harm cause by torcetrapib was independent of any of the factors measured. One unexpected result was the observation that event rates in both groups taking 80 mg atorvastatin (with or without torcetrapib) were higher than in those taking lower doses of atorvastatin. The explanation for this is not known.

It should be noted that the concentration of HDL-C does not necessarily equate with HDL function (7) and that HDL function was not assessed in the ILLUMINATE trial. We cannot, therefore, exclude the possibility that HDL function may have been impaired by torcetrapib and that the impairment was greater in the 10 mg atorvastatin group than in those taking higher doses of atorvastatin.

TABLE 5. Change in values from baseline to month 3 by atorvastatin dose

		Atorvastatin dose							
		10 mg		20 mg		40 mg		80 mg	
Variable	*P*	T/A	A	T/A	A	T/A	A	T/A	A
HDL-C (mg/dl)	<0.001	30 ± 14.4<br>n = 3,100	0.5 ± 6.1<br>n = 3,180	28.2 ± 14.3<br>n = 1,964	0.2 ± 6.0<br>n = 1,961	28.4 ± 14.4<br>n = 1,447	0.5 ± 6.3<br>n = 1510	28.0 ± 14.1<br>n = 745	1.1 ± 6.9<br>n = 663
LDL-C (mg/dl)	0.195	−21.7 ± 20.0<br>n = 3,090	0.8 ± 15.4<br>n = 3,170	−20.8 ± 22.1<br>n = 1,954	0.5 ± 16.6<br>n = 1,955	−21.6 ± 21.2<br>n = 1,443	0.6 ± 16.8<br>n = 1,504	−20.4 ± 23.6<br>n = 740	0.1 ± 18.6<br>n = 659
ApoA-I (mg/dl)	<0.001	26.4 ± 24.3<br>n = 3,059	1.5 ± 21.7<br>n = 3,135	24.8 ± 29.2<br>n = 1,929	1.1 ± 17.7<br>n = 1,934	24.2 ± 20.1<br>n = 1,426	1.4 ± 14.1<br>n = 1,497	24.4 ± 17.5<br>n = 736	1.4 ± 14.1<br>n = 652
ApoB (mg/dl)	0.225	−12,9 ± 18.4<br>n = 3,060	2.1 ± 15.9<br>n = 3,060	−11.8 ± 20.8<br>n = 1,929	1.9 ± 17.5<br>n = 1,934	−13.1 ± 17.9<br>n = 1,425	2.0 ± 16.7<br>n = 736	−11.6 ± 20.2<br>n = 736	1.1 ± 16.5<br>n = 652
Systolic BP (mm Hg)	0.121	4.8 ± 12.2<br>n = 3,135	0.3 ± 10.7<br>n = 3,207	4.1 ± 11.4<br>n = 1,973	0.4 ± 10.5<br>n = 1,972	4.0 ± 11.5<br>n = 1,460	0.7 ± 10.2<br>n = 1,517	4.9 ± 12.3<br>n = 749	0.4 ± 11.0<br>n = 667
Potassium (mmol/l)	0.325	−0.1 ± 0.4<br>n = 3,081	0.0 ± 0.4<br>n = 3,155	−0.1 ± 0.4<br>n = 1,955	0.0 ± 0.4<br>n = 1,950	−0.1 ± 0.4<br>n = 1,440	0.0 ± 0.4<br>n = 1,502	−0.1 ± 0.4<br>n = 742	0.0 ± 0.4<br>n = 659
Sodium (mmol/l)	0.109	0.6 ± 3.3<br>n = 3,099	−0.1 ± 3.1<br>n = 3,168	0.6 ± 3.3<br>n = 1,963	0.0 ± 3.2<br>n = 1,960	0.6 ± 3.1<br>n = 1,447	0.1 ± 3.1<br>n = 1,509	0.4 ± 3.1<br>n = 744	0.0 ± 3.1<br>n = 662
Bicarbonate (mmol/l)	0.571	0.9 ± 3.7<br>n = 3,089	0.6 ± 3.8<br>n = 3,163	0.8 ± 3.3<br>n = 1,954	0.5 ± 3.4<br>n = 1,956	0.7 ± 3.4<br>n = 1,444	0.6 ± 3.3<br>n = 1,504	0.9 ± 3.2<br>n = 740	0.5 ± 3.2<br>n = 661
Aldosterone (ng/dl))	0.214	0.4 ± 6.5<br>n = 863	0.3 ± 5.2<br>n = 800	0.7 ± 5.3<br>n = 607	−0.4 ± 8.0<br>n = 527	0.8 ± 5.5<br>n = 399	0.2 ± 5.7<br>n = 404	0.4 ± 7.3<br>n = 188	0.0 ± 6.6<br>n = 170
CRP (mg/l)	0.461	0.0 (−0.5, 0.6)<br>n = 3,074	0.0 (−0.6, 0.6)<br>n = 3,149	0.1 (−0.3, 0.7)<br>n = 1,944	0.1 (−0.4, 0.7)<br>n = 1,943	0.0 (−0.4, 0.5)<br>n = 1,439	0.0 (−0.5, 0.5)<br>n = 1497	0.0 (−0.4, 0.5)<br>n = 737	0.0 (−0.6, 0.5)<br>n = 656

Mean ± SD, except for C-reactive protein (CRP) values that are medians (interquartile range). *P* values indicate the test of heterogeneity across the atorvastatin doses. T/A, torcetrapib plus atorvastatin; A, atorvastatin alone.

The observation that postrandomization changes in LDL-C, SBP, aldosterone, and electrolytes were not associated with adverse clinical outcome should again be interpreted with caution, inasmuch as post hoc exploratory analyses are known to have methodological limitations and thus may have the potential to yield false-negative results (8).

If the harm caused by torcetrapib cannot be easily explained by changes in lipid levels, SBP, electrolytes, aldosterone levels, or CRP, the question arises: what did cause the harm? Clearly, this question cannot be answered by a post hoc analysis of the ILLUMINATE trial, but there are some interesting possibilities. One is that increasing doses of atorvastatin had beneficial effects on lipoprotein metabolism and function (unrelated to lipoprotein concentration) that counteracted the adverse effects of torcetrapib. However, there are other possibilities that have some support from preclinical studies. For example, clues from recent studies have shown a reduced expression of endothelial nitric oxide synthase mRNA and protein, reduced nitric oxide release, and an increased expression of endothelin-1 in the arteries of animals treated with torcetrapib (4). These changes coincided with a reduced endothelium-dependent vasorelaxation. Because endothelin-1 levels were not measured in the ILLUMINATE trial, it was not possible to determine whether a torcetrapib-induced increase in endothelin-1 in arteries may have contributed to the cardiovascular harm observed in those taking torcetrapib in this trial. Nevertheless, given the evidence that endothelin-1 levels are also associated with a worse prognosis in people with cancer (9), it is possible that a torcetrapib-induced increase in endothelin-1 may also have contributed to the apparent (nonsignificant) increase in cancer deaths in those taking torcetrapib in the ILLUMINATE trial. Given the evidence that atorvastatin reduces endothelin-1 levels (10–12), it may be speculated that treatment with higher doses of atorvastatin partially protected against this adverse effect of torcetrapib. An analogous argument can be made with regard to cortisol levels. Preclinical experiments have shown that torcetrapib stimulates cortisol release via induction of 11 β-hydroxylase (2, 3). It has also been reported that elevated cortisol levels are associated with poorer cancer survival in a mechanism involving inflammatory cytokines such as IL-6 (13). The fact that atorvastatin reduces IL-6 levels (14) may again explain the apparent protection against cancer death in those taking higher doses of atorvastatin. It should be noted, however, that although the excess of ACM in those taking atorvastatin plus torcetrapib (compared with those taking atorvastatin alone) was significant in the 10 mg atorvastatin group (but not in the higher-atorvastatin groups), there were very few deaths attributed to cancer and infection across all atorvastatin subgroups, limiting conclusions to be drawn about these specific causes of death.

Other than the dose of atorvastatin, increasing age was the most important factor predicting the torcetrapib-induced increase in ACM, an effect that could not be explained by differing lipid responses in this group. These age-related findings are consistent with the observation of a reduced clearance of torcetrapib in older subjects (unpublished observation) that would result in a higher exposure to the drug and thus an increased risk of adverse effects. Baseline predictors of mortality other than age included a history of CHF or stroke and high baseline CRP (>3 mg/l). A lower baseline apoA-I and a history of hypertension, CHF, myocardial infarction, stroke, or being a smoker were all associated with an increased risk of having an MCVE. For both endpoints, the increased risk associated with these factors is not unexpected, based on prior

experience in clinical trials. However, after adjusting for these predictors, the hazard associated with taking torcetrapib in the 10 mg atorvastatin group remained virtually unaltered. Furthermore, with the exception of minor differences in the increase in HDL-C and apoA-I, the torcetrapib-induced changes in these parameters were not different between the atorvastatin 10 mg dose and the higher-atorvastatin dose subgroups.

It should be emphasized that the analyses reported in this paper were not prespecified and that conclusions drawn from post hoc examinations such as these should be viewed with a degree of caution. It is possible, for example, that conclusions may have been different had the trial not been terminated early (the median follow-up was only 550 days) and had data been available over a longer follow-up.

It must also be emphasized that even in the absence of the adverse off-target of torcetrapib, it cannot be assumed that CETP inhibition will be cardio-protective. The unexpected termination (for reasons of futility but not harm) of the dalOUTCOMES trial using dalcetrapib (15) has raised questions about the value of CETP inhibition as a cardio-protective strategy. However, the hypothesis will be tested in an ongoing clinical outcome trial using anacetrapib, a CETP inhibitor that is much more potent than dalcetrapib and which does not have the adverse off-target effects of torcetrapib (16).

In conclusion, we have shown that adverse effects of torcetrapib in the ILLUMINATE trial were largely confined to participants taking the lowest dose of atorvastatin. This effect of atorvastatin dose cannot be explained in terms of changes in lipid levels, blood pressure, electrolytes, or indeed in any of the variables that were measured. It is possible that the adverse effects were the consequence of a torcetrapib-induced increase in endothein-1 in the artery and that this adverse effect was partly offset in those taking higher doses of atorvastatin.

REFERENCES

1. Barter, P. J., M. Caulfield, M. Eriksson, S. M. Grundy, J. J. Kastelein, M. Komajda, J. Lopez-Sendon, L. Mosca, J. C. Tardif, D. D. Waters, et al. 2007. Effects of torcetrapib in patients at high risk for coronary events. *N. Engl. J. Med.* **357:** 2109–2122.
2. Forrest, M. J., D. Bloomfield, R. J. Briscoe, P. N. Brown, A. M. Cumiskey, J. Ehrhart, J. C. Hershey, W. J. Keller, X. Ma, H. E. McPherson, et al. 2008. Torcetrapib-induced blood pressure elevation is independent of CETP inhibition and is accompanied by increased circulating levels of aldosterone. *Br. J. Pharmacol.* **154:** 1465–1473.
3. Hu, X., J. D. Dietz, C. Xia, D. R. Knight, W. T. Loging, A. H. Smith, H. Yuan, D. A. Perry, and J. Keiser. 2009. Torcetrapib induces aldosterone and cortisol production by an intracellular calcium-mediated mechanism independently of cholesteryl ester transfer protein inhibition. *Endocrinology.* **150:** 2211–2219.
4. Simic, B., M. Hermann, S. G. Shaw, L. Bigler, U. Stalder, C. Dorries, C. Besler, T. F. Luscher, and F. Ruschitzka. 2012. Torcetrapib impairs endothelial function in hypertension. *Eur. Heart J. In press.*
5. Liao, J. K. 2005. Clinical implications for statin pleiotropy. *Curr. Opin. Lipidol.* **16:** 624–629.
6. de Haan, W., J. de Vries-van der Weij, J. W. van der Hoorn, T. Gautier, C. C. van der Hoogt, M. Westerterp, J. A. Romijn, J. W. Jukema, L. M. Havekes, H. M. Princen, et al. 2008. Torcetrapib does not reduce atherosclerosis beyond atorvastatin and induces more proinflammatory lesions than atorvastatin. *Circulation.* **117:** 2515–2522.
7. Rader, D. J. 2007. Illuminating HDL—is it still a viable therapeutic target? *N. Engl. J. Med.* **357:** 2180–2183.
8. Fleming, T. R. 2008. Identifying and addressing safety signals in clinical trials. *N. Engl. J. Med.* **359:** 1400–1402.
9. Bagnato, A., M. Loizidou, B. R. Pflug, J. Curwen, and J. Growcott. 2011. Role of the endothelin axis and its antagonists in the treatment of cancer. *Br. J. Pharmacol.* **163:** 220–233.
10. Chang, C. Z., S. C. Wu, C. L. Lin, S. L. Hwang, S. L. Howng, and A. L. Kwan. 2010. Atorvastatin preconditioning attenuates the production of endothelin-1 and prevents experimental vasospasm in rats. *Acta Neurochir. (Wien).* **152:** 1399–1406.
11. Maguire, J. J., K. E. Wiley, R. E. Kuc, V. E. Stoneman, M. R. Bennett, and A. P. Davenport. 2006. Endothelin-mediated vasoconstriction in early atherosclerosis is markedly increased in ApoE$-/-$ mouse but prevented by atorvastatin. *Exp. Biol. Med. (Maywood).* **231:** 806–812.
12. Strey, C. H., J. M. Young, J. H. Lainchbury, C. M. Frampton, M. G. Nicholls, A. M. Richards, and R. S. Scott. 2006. Short-term statin treatment improves endothelial function and neurohormonal imbalance in normocholesterolaemic patients with non-ischaemic heart failure. *Heart.* **92:** 1603–1609.
13. Lissoni, P., F. Brivio, L. Fumagalli, G. Messina, G. Secreto, B. Romelli, G. Fumagalli, F. Rovelli, M. Colciago, and G. Brera. 2007. Immune and endocrine mechanisms of advanced cancer-related hypercortisolemia. *In Vivo.* **21:** 647–650.
14. Kinlay, S., G. G. Schwartz, A. G. Olsson, N. Rifai, S. J. Leslie, W. J. Sasiela, M. Szarek, P. Libby, and P. Ganz. 2003. High-dose atorvastatin enhances the decline in inflammatory markers in patients with acute coronary syndromes in the MIRACL study. *Circulation.* **108:** 1560–1566.
15. Cannon, C. P., S. Shah, H. M. Dansky, M. Davidson, E. A. Brinton, A. M. Gotto, M. Stepanavage, S. X. Liu, P. Gibbons, T. B. Ashraf, et al. 2010. Safety of anacetrapib in patients with or at high risk for coronary heart disease. *N. Engl. J. Med.* **363:** 2406–2415.
16. Schwartz, G. G., A. G. Olsson, C. M. Ballantyne, P. J. Barter, I. M. Holme, D. Kallend, L. A. Leiter, E. Leitersdorf, J. J. McMurray, P. K. Shah, et al. 2009. Rationale and design of the dal-OUTCOMES trial: efficacy and safety of dalcetrapib in patients with recent acute coronary syndrome. *Am. Heart J.* **158:** 896–901.
