# Supplementary material for: Lipid-lowering effect of combined therapy with high-intensity statins and CETP inhibitors: a Systematic Review and meta-analysis
Source: Front Endocrinol (Lausanne). 2025 May 1;16:1512670. doi: 10.3389/fendo.2025.1512670 (PMC12078159; doi:10.3389/fendo.2025.1512670)
Supplement: Supplementary file 1 [file DataSheet1.zip › Raw Data/Raw Data/Original Documentation Fruit/Furtado2022✔.pdf]

Arteriosclerosis, Thrombosis, and Vascular Biology

CLINICAL AND POPULATION STUDIES

Pharmacological Inhibition of CETP (Cholesteryl Ester Transfer Protein) Increases HDL (High-Density Lipoprotein) That Contains ApoC3 and Other HDL Subspecies Associated With Higher Risk of Coronary Heart Disease

Jeremy D. Furtado, Giacomo Ruotolo, Stephen J. Nicholls, Robert Dullea, Santos Carvajal-Gonzalez, Frank M. Sacks

OBJECTIVE: Plasma total HDL (high-density lipoprotein) is a heterogeneous mix of many protein-based subspecies whose functions and associations with coronary heart disease vary. We hypothesize that increasing HDL by CETP (cholesteryl ester transfer protein) inhibition failed to reduce cardiovascular disease risk, in part, because it increased dysfunctional subspecies associated with higher risk such as HDL that contains apoC3.

APPROACH AND RESULTS: We studied participants in 2 randomized, double-blind, placebo-controlled trials of a CETP inhibitor on a background of atorvastatin treatment: ACCENTUATE (The Addition of Evacetrapib to Atorvastatin Compared to Placebo, High Intensity Atorvastatin, and Atorvastatin With Ezetimibe to Evaluate LDL-C Lowering in Patients With Primary Hyperlipidemia; 130 mg evacetrapib; n=126) and ILLUMINATE (Phase 3 Multi Center, Double Blind, Randomized, Parallel Group Evaluation of the Fixed Combination Torcetrapib/Atorvastatin, Administered Orally, Once Daily [Qd], Compared With Atorvastatin Alone, on the Occurrence of Major Cardiovascular Events in Subjects With Coronary Heart Disease or Risk Equivalents; 60 mg torcetrapib; n=80). We measured the concentration of apoA1 in total plasma and 17 protein-based HDL subspecies at baseline and 3 months. Both CETP inhibitors increased apoA1 in HDL that contains apoC3 the most of all HDL subspecies (median placebo-adjusted percent increase: evacetrapib 99% and torcetrapib 50%). They also increased apoA1 in other HDL subspecies associated with higher coronary heart disease risk such as those involved in inflammation (α-2-macroglobulin and complement C3) or hemostasis (plasminogen), and in HDL that contains both apoE and apoC3, a complex subspecies associated with higher coronary heart disease risk. ApoA1 in HDL that contains apoC1, associated with lower risk, increased 71% and 40%, respectively. Only HDL that contains apoL1 showed no response to either drug.

CONCLUSIONS: CETP inhibitors evacetrapib and torcetrapib increase apoA1 in HDL subspecies that contain apoC3 and other HDL subspecies associated with higher risk of coronary heart disease. Subspecies-specific effects shift HDL subspecies concentrations toward a profile associated with higher risk, which may contribute to lack of clinical benefit from raising HDL by pharmaceutical CETP inhibition.

GRAPHIC ABSTRACT: A graphic abstract is available for this article.

Key Words: apolipoproteins ■ cholesterol ester transfer proteins, antagonists & inhibitors ■ heart diseases ■ hydroxymethylglutaryl-CoA reductase inhibitors ■ lipoproteins, HDL

The concentration of HDL-C (cholesterol in high-density lipoproteins) in the blood, widely accepted as an inverse risk factor for cardiovascular disease (CVD), is routinely measured in primary care screening and was the target of therapies to reduce risk.[1] However, it is not the cholesterol itself that reduces risk of CVD. Rather, the

Correspondence to: Jeremy D. Furtado, ScD, Department of Nutrition, Harvard T.H. Chan School of Public Health, 665 Huntington Avenue, Boston, MA 20115. Email jfurtado@hsph.harvard.edu

Supplemental Material is available at https://www.ahajournals.org/doi/suppl/10.1161/ATVBAHA.121.317181.

For Sources of Funding and Disclosures, see page 235.

© 2021 The Authors. *Arteriosclerosis, Thrombosis, and Vascular Biology* is published on behalf of the American Heart Association, Inc., by Wolters Kluwer Health, Inc. This is an open access article under the terms of the Creative Commons Attribution Non-Commercial-NoDerivs License, which permits use, distribution, and reproduction in any medium, provided that the original work is properly cited, the use is noncommercial, and no modifications or adaptations are made.

Arterioscler Thromb Vasc Biol is available at www.ahajournals.org/journal/atvb

Nonstandard Abbreviations and Acronyms

**CETP**	cholesteryl ester transfer protein
**CHD**	coronary heart disease
**CVD**	cardiovascular disease
**HDL-C**	high density lipoprotein cholesterol
**LDL-C**	low-density lipoprotein cholesterol
**PON-1**	paraoxonase-1
**SR-BI**	scavenger receptor BI
**VLDL**	very-low-density lipoprotein

Highlights

- CETP (Cholesteryl ester transfer protein) inhibitors torcetrapib and evacetrapib increase the apoA1 concentration of protein-defined HDL (high-density lipoprotein) subspecies to markedly different degrees.
- HDL subspecies differ in their association with coronary heart disease: some are associated with lower risk, some have no association with coronary heart disease, and some are associated with higher risk. Pharmacological increases in HDL concentration would not be beneficial if they are achieved with dysfunctional types of HDL that are associated with null or higher risk of coronary heart disease.
- ApoA1 concentration in HDL that contains apoC3, a dysfunctional HDL subspecies, increased the most of all subspecies. ApoA1 in HDL that contains apoE was also increased, but this increase was in its non-protective subtype that also contains apoC3.
- Taken together, the changes in apoA1 concentrations in protein-defined HDL subspecies elicited by torcetrapib and evacetrapib result in an altered HDL profile that may be less protective, which could contribute to the lack of clinical benefit from pharmaceutical CETP inhibition.

HDLs carrying that cholesterol are the actors that facilitate efflux of cholesterol from tissues for excretion via the liver, mediate inflammation and oxidation, and participate in protective hemostatic and apoptotic processes.[2] Thus, while epidemiological evidence supports HDL-C as a biomarker for CVD risk, the measure is inherently imperfect, merely providing a snapshot of the amount of the cholesterol within a dynamic system of HDL subject to perturbation. For example, reduced ability of HDLs to deliver cholesterol to the liver via SR-BI (scavenger receptor BI) results in elevated levels of HDL-C, but rather than a decrease in risk of coronary heart disease, there is an increase.[3] With reduced clearance through the liver, plasma HDL-C rises as HDLs continue to efflux cholesterol from tissues. In this case, elevated HDL-C is a marker of dysfunction. Conversely, enhanced efficiency of the same mechanism results in low levels of plasma HDL-C due to increased flux of cholesterol out of the body. In this case, a low level of HDL-C is a marker of enhanced function. Furthermore, neither of these perturbations in HDL-C concentration may have any effect on the other protective functions of HDL beyond reverse cholesterol transport. Thus, HDL-C is not a reliable marker of protection against CVD imparted by HDLs. Recent epidemiological studies have included individuals with very high levels of HDL-C and have found that the inverse linear relationship between HDL-C and CVD risk is lost at elevated levels.[4,5]

CETP (Cholesteryl ester transfer protein) is a plasma protein that transfers cholesteryl ester from HDL particles to apoB-containing lipoproteins in exchange for triglyceride.[6,7] Epidemiological evidence that HDL-C concentration is associated with lower CVD risk while LDL-C (low-density lipoprotein cholesterol) is associated with higher risk sparked the hypothesis that this transfer of cholesterol by CETP increases risk of CVD. Indeed, studies in certain animal models found the actions of CETP to be proatherogenic. Mice are naturally deficient in CETP yet are relatively resistant to diet-induced atherosclerosis.[7] The introduction and expression of the simian *cetp* gene resulted in enhanced formation of fatty streak lesions compared with controls, primarily due to redistribution of cholesterol from HDL to VLDL (very-low-density lipoprotein) and LDL.[8] Induction of CETP expression in APOE*3-Leiden mice, which exhibit a human-like lipoprotein profile and develop atherosclerosis upon feeding with saturated fat and cholesterol, shifted the distribution of cholesterol from HDL to VLDL and LDL and reduced plasma-mediated SR-BI-dependent cholesterol efflux.[9] It was the increased VLDL-cholesterol rather than the decreased HDL-cholesterol that was found to increase atherosclerosis in these mice.[10] Unlike mice, rabbits normally express high levels of CETP and are highly susceptible to the development of diet-induced atherosclerosis.[7] In a study of cholesterol-fed rabbits, animals with their plasma CETP activity reduced through antibody inhibition exhibited a substantial increase in the concentration of HDL-C, a modest decrease in LDL-C concentration, and a significant reduction in aortic atherosclerotic lesions, indicating reduced susceptibility to atherosclerosis.[11] In another study, administration of a CETP inhibitor to rabbits increased HDL-C, decreased non-HDL-cholesterol, and inhibited the progression of atherosclerosis.[12] However, other animal models have found CETP to be protective. Atherosclerosis was reduced by CETP in mice engineered to overexpress human apoC3.[13] Expression of human CETP protects SR-BI deficient mice from atherosclerosis.[14] In addition, in transgenic mice expressing human LCAT (lecithin-cholesterol acyltransferase) that have an increased concentration of HDL-cholesterol but also an increased susceptibility to atherosclerosis, expression of the simian *cetp* gene reduces atherosclerosis.[15]

The relationship between CETP and CVD risk in humans is equally complex. Although genetic polymorphisms in

humans that reduce expression of CETP are associated with higher HDL-C and apoA1,[16–18] associations with CVD risk are inconsistent.[19–26] Genome-wide association studies and Mendelian randomization studies have identified several genes that are associated with higher plasma HDL-C but are not associated with lower risk of CVD.[27–30] Furthermore, a CETP mutation has been identified in humans that results in lower HDL-C concentration yet is associated with reduced risk of ischemic heart disease.[31] Pharmaceutical inhibition of CETP significantly increases HDL-C concentrations, but these increases have not led to reduced risk of CVD. Two of these drugs, evacetrapib and torcetrapib, increased HDL-C by 125% and 29% and apoA1 by 46% and 25%, and both were found to increase the cholesterol efflux capacity of total HDL, yet neither reduced cardiovascular events.[32–34] Anacetrapib reduced coronary heart disease (CHD) by 9%, but this mild effect is consistent with its 18% lowering of non-HDL-cholesterol, rather than its doubling of HDL-C level.[35] Taken together, the evidence suggests that the concentration of HDL-C or plasma apoA1 may not be a reliable marker of the cardioprotective quality of HDL.

HDL is a highly heterogeneous collection of lipid-protein complexes comprised of various lipids, apoA1, and an assortment of over 200 other lipophilic proteins (https://homepages.uc.edu/~davidswm/HDLproteome.html), some of which define stable HDL subspecies.[36–39] We recently identified and characterized the proteomes of 15 novel protein-defined HDL subspecies.[39] The subspecies each comprised 1% to 18% of total HDL, and their concentrations were stable within an individual over 1 to 2 years. Several of these subspecies, defined by proteins such as apoC3, complement C3, α-2-macroglobulin, haptoglobin, or plasminogen, were associated with higher risk of CHD than the HDL that lacked the defining protein, whereas HDL that contained apoC1 or apoE were associated with lower risk.[40] Among these subspecies, HDL that contains apoC3 has been studied the most extensively. This subspecies is associated with higher risk of subclinical atherosclerosis, CHD, insulin resistance, and type 2 diabetes.[38,40–45] Furthermore, the copresence of apoC3 on HDL that contains apoE eliminates its protective association against CHD by impairing metabolic pathways active in reverse cholesterol transport that moves cholesterol from macrophages to the liver.[37]

We hypothesized that effects of CETP on protein-based HDL subspecies differently associated with CHD risk may in part explain the lack of therapeutic benefit on CHD of raising total HDL by CETP inhibition. For example, inhibition of CETP by evacetrapib and torcetrapib may increase detrimental subspecies such as HDL that contains apoC3. To test this hypothesis, we measured total plasma apoA1 and apoA1 in 17 protein-based HDL subspecies at baseline and 3 months in blood samples from participants in 2 randomized, double-blind, placebo-controlled trials.[32,33]

MATERIALS AND METHODS

To minimize the possibility of unintentionally sharing information that can be used to reidentify participants, a subset of the data generated for this study is available from the corresponding author upon reasonable request.

Study Population

The details of the ACCENTUATE (The Addition of Evacetrapib to Atorvastatin Compared to Placebo, High Intensity Atorvastatin, and Atorvastatin With Ezetimibe to Evaluate LDL-C Lowering in Patients With Primary Hyperlipidemia) and ILLUMINATE (Phase 3 Multi Center, Double Blind, Randomized, Parallel Group Evaluation of the Fixed Combination Torcetrapib/Atorvastatin, Administered Orally, Once Daily [Qd], Compared With Atorvastatin Alone, on the Occurrence of Major Cardiovascular Events in Subjects With Coronary Heart Disease or Risk Equivalents) trials have been published.[32,33] Both were multicenter, prospective, randomized, double-blind, parallel, placebo-controlled clinical trials. Details of the trials are provided in the Supplemental Material, and the characteristics of the participants are shown in the Table. Briefly, the ACCENTUATE trial (supported by Eli Lilly and Company, URL: https://www.clinicaltrials.gov; Unique identifier: NCT02227784) was a Phase 3 randomized clinical trial designed to evaluate the effects of evacetrapib on HDL-C and other lipids in participants with high cholesterol and atherosclerotic CVD and diabetes. Atorvastatin was given as a background medication to both evacetrapib and placebo groups to control and equalize LDL-C. Our study compared all of the subjects included in the final efficacy analysis from 2 of the study treatment arms: atorvastatin 40 mg and evacetrapib 130 mg daily (n=86); and atorvastatin 40 mg and placebo evacetrapib (n=40) daily. The ILLUMINATE trial (supported by Pfizer, URL: https://www.clinicaltrials.gov; Unique identifier: NCT00134264) was a Phase 3 randomized controlled trial designed to demonstrate if torcetrapib plus atorvastatin could reduce the risk for major CVD events compared with atorvastatin alone in patients with coronary heart disease or risk equivalents. Our study randomly selected 20 men and 20 women from the atorvastatin 10 mg + torcetrapib 60 mg daily dose group and an additional 40 participants from the atorvastatin 10 mg group + placebo torcetrapib who were matched on sex, race, and age (within 1 year).

Laboratory Analysis

Stored plasma samples from the ACCENTUATE trial of evacetrapib and the ILLUMINATE trial of torcetrapib collected at baseline and after 3 months were shipped on dry ice by overnight courier to the Harvard T.H. Chan School of Public Health and stored at −80°C pending analysis. Modified sandwich ELISAs to quantify the concentrations of the 17 HDL subspecies defined by selected proteins were performed as described in detail previously[39,40] and in the Supplemental Material. All HDL subspecies are quantified as the concentration of apoA1 (mg/dL) in that subspecies. To minimize batch effects, baseline and 3-month samples were paired side-by side in the same ELISA plate in random order and CETP-treated and untreated samples were alternated with equal numbers on each ELISA plate. The lab was blinded to time point and treatment status.

For the analysis of apoA1 concentration in HDL subspecies defined by content of both apoE and apoC3, samples were

CLINICAL AND POPULATION STUDIES - AL

Table. Participant Characteristics

	ACCENTUATE			ILLUMINATE		
	Full study	Placebo	Evacetrapib (130 mg)	Full study	Placebo	Torcetrapib (60 mg)
N	366	36	81	15 067	40	40
White, %	81.1	80.6	80.2	93	100*	100
Diabetes, %	50.5	36.1	48.1	45	75	70
Males, %	66.1	72.2	67.9	78	50*	50
Age, y	63±9	63±8	63±8	61±8	65±6*	65±6
Body mass index, kg/m^2	31.7±7.0	30.4±6.4	31.0±6.0	30.2±5.6	31.7±7.2	30.6±6.6
Triglycerides,† mg/dL	129 (97–180)	123 (84–159)	128 (88–189)	128 (93–179)	184 (123–219)	120 (87–208)
LDL-C, mg/dL	85±19	83±17	88±17	80±20	85±20	75±23
HDL-C, mg/dL	48±13	52±16	48±13	49±12	48±12	50±12
ApoA-I, mg/dL	141±26	149±32	142±27	128±23	133±21	134±25
ApoB, mg/dL	83±16	80±17	84±15	74±16	79±18	72±16

Accentuate placebo, atorvastatin 40 mg. Illuminate placebo, atorvastatin 10 mg. ACCENTUATE indicates The Addition of Evacetrapib to Atorvastatin Compared to Placebo, High Intensity Atorvastatin, and Atorvastatin With Ezetimibe to Evaluate LDL-C Lowering in Patients With Primary Hyperlipidemia; HDL, high-density lipoprotein; ILLUMINATE, Phase 3 Multi Center, Double Blind, Randomized, Parallel Group Evaluation of the Fixed Combination Torcetrapib/Atorvastatin, Administered Orally, Once Daily [Qd], Compared With Atorvastatin Alone, on the Occurrence of Major Cardiovascular Events in Subjects With Coronary Heart Disease or Risk Equivalents; IQR, interquartile range; and LDL, low-density lipoprotein.

Data are means±SD or %;

*matching factor; and

†median (IQR).

fractionated by sequential immuno-affinity column chromatography and then measured for concentration of apoA1, apoE, and apoC3 by standard sandwich ELISA as described in detail by Talayero et al.[46] Due to the time-consuming protocol and expense of immuno-affinity column chromatography, a subset of 14 samples was randomly selected from the ILLUMINATE trial from those individuals receiving torcetrapib excluding the 5 individuals for whom torcetrapib did not increase HDL that contains apoE. Samples from the ACCENTUATE trial had insufficient volume available to be included in this secondary analysis. The efficiencies of the apoC3 and apoE immuno-affinity columns were 93% and 96%, respectively. ELISAs were performed as described in the Supplemental Material.

Statistics

All statistical analyses were performed using the SAS software package, version 9.4 (SAS Institute Inc, Cary, NC). Percent change for concentration of apoA1 in total plasma and in each of the 17 HDL subspecies was calculated by taking the difference between the concentrations in the 3-month sample and the baseline sample, then dividing by the baseline concentration. The median change in apoA1 concentration and the median percent change in apoA1 concentration of total plasma and the HDL subspecies in the placebo and treatment groups were compared by Wilcoxon rank-sum test. The proportion of HDL comprised of each individual HDL subspecies at baseline and at 3 months was calculated as the concentration of apoA1 in that HDL subspecies divided by the concentration of apoA1 in total plasma. The percent change in the proportion of HDL comprised of each individual HDL subspecies was calculated as the difference between the proportion of HDL comprised of the HDL subspecies at 3 months minus the proportion of HDL comprised the HDL subspecies at baseline divided by the proportion of HDL comprised of the HDL subspecies at baseline. This percent change was tested for difference from zero by Wilcoxon rank-sum test. For all tests, multiple comparison adjustment with control for false discovery rate was used because the HDL subspecies overlap and their concentrations and changes in concentrations are correlated, as in the genomic literature.[47,48] While this study was not powered for subgroups analyses, we looked for evidence that drug response may have been associated with sex, age, or comorbidity.

RESULTS

The baseline characteristics of the full and subset study populations are summarized in the Table. In the subset of samples used in this study, the patients were predominantly white, overweight or obese, and mean age 63 to 65 years. The baseline characteristics comparing the CETP inhibitors with placebo are generally similar except for triglyceride in the torcetrapib study (median baseline torcetrapib 120 mg/dL versus median baseline placebo 184 mg/dL).

CETP Inhibitor Treatment Increased the ApoA1 Concentration of Total HDL and of HDL Subspecies Defined by Proteins With Lipid Metabolism Functions, Raising HDL That Contains ApoC3 the Most

Torcetrapib and evacetrapib increased median placebo-adjusted concentration of total apoA1 of 30% and 60%, respectively (Figures 1 and 2, Tables S1 and S2). The largest treatment effects occurred in the HDL subspecies defined by proteins linked to lipid metabolism. Compared with placebo, both treatments increased apoA1 concentration in HDL that contains apoC3 by the largest percentage of all subspecies (median placebo-adjusted increase of 50% for torcetrapib and 99% for evacetrapib, both FDR (false detection rate)-adjusted $P<0.001$; Figures 1 and 2, Tables S1 and S2). This was nearly double the percent

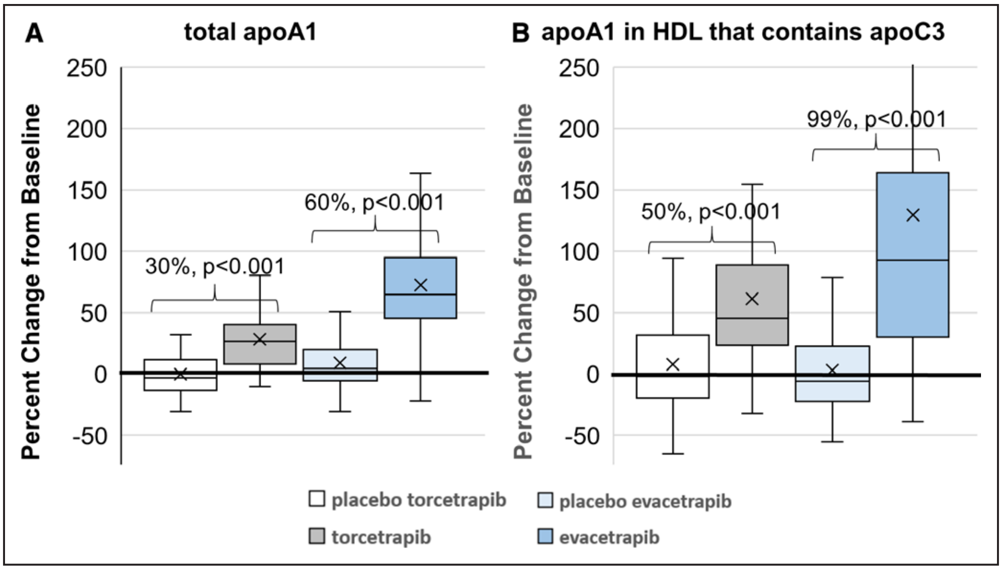

Figure 1. Torcetrapib and evacetrapib increase the concentrations of apoA1 in total plasma and in HDL (high-density lipoprotein) that contains apoC3 compared with placebo.

Atorvastatin was given as a background medication to all groups to control and equalize LDL (low-density lipoprotein)-cholesterol. Percentages indicated above the bars are median placebo-adjusted changes from baseline. Box plots show median, interquartile range, and mean (indicated by x). *P* values are for effect of drug compared with placebo and are FDR (false detection rate)-adjusted.

increase in total apoA1. ApoA1 concentration in HDL that contains apoE also increased by a larger percentage than total apoA1 (40% and 86% relative to placebo, respectively, $P<0.001$; Figure 2, Tables S1 and S2). Both drugs increased apoA1 concentration in HDL that contains apoC1 (40% and 71%, $P<0.001$) and in HDL that contains apoJ (32%, $P=0.02$; 49%, $P<0.001$). Evacetrapib increased apoA1 concentration in HDL that contains apoA4 (70%, $P<0.001$) and in HDL that contains apoC2 (54%, $P<0.001$). Both drugs increased apoA1 concentration in HDL that contains apoA2 (28% and 44%, $P<0.001$), a subspecies that comprises ≈80% of total HDL, while they increased apoA1 concentration in the complementary subspecies HDL that lacks apoA2 by a larger percentage (39% and 76%, $P<0.001$; Figure 2, Tables S1 and S2).

The CETP Inhibitor Mediated Increase in HDL That Contains ApoE Occurs Only in Its Dysfunctional Subtype That Also Contains ApoC3

HDL subspecies that contain apoE or apoC3 overlap substantially in their proteomes[39]; about 50% of HDL that contains apoC3 also contains apoE, and 50% of HDL that contains apoE also contains apoC3. HDL that contains apoE but not apoC3 is associated with lower risk, but risk is elevated[37] in HDL particles that also contain apoC3. Therefore, we studied the effect of CETP inhibition on HDL subspecies defined simultaneously by both apoE and apoC3 in a subset of 14 participants in the torcetrapib study. Torcetrapib increased the concentration of apoA1 in HDL that contains apoC3 but lacks apoE by a median of 96% and increased apoA1 concentration in HDL that contains both apoE and apoC3 by 43%. These subspecies are associated with higher CHD risk. In contrast, torcetrapib did not increase the concentration of apoA1 in HDL that contains apoE but lacks apoC3, the subspecies associated with lower risk (Figure 3). The results were not affected by 2 outliers, one from the subspecies that lacks apoE and apoC3 (458% increase) and the other from the subspecies that contains apoE and lacks apoC3 (988% increase).

Evacetrapib Treatment Also Increased the Concentration of HDL Subspecies Defined by Proteins Linked to Functions Other Than Lipid Metabolism

Compared with placebo, evacetrapib increased the concentration of apoA1 in HDL subspecies defined by proteins associated with hemostasis (HDL that contains plasminogen or fibrinogen, 43% and 44%, $P<0.001$) or inflammation (HDL that contains α-1-antitrypsin or α-2-macroglobulin, 69% and 32%, $P\leq0.01$; Figure 2, Tables S1 and S2). HDL subspecies defined by proteins associated with antioxidation include HDL that contains ceruloplasmin, haptoglobin, or PON-1 (paraoxonase-1). Compared with placebo, evacetrapib increased apoA1 concentration in these HDL subspecies by 27% to 41% ($P\leq0.01$; Figure 2, Tables S1 and S2). ApoA1 concentration in HDL that contains complement C3, a subspecies related to inflammation and immunity, is also increased by evacetrapib (30%, FDR-adjusted $P=0.02$; Figure 2, Tables S1 and S2). Like evacetrapib, torcetrapib increased apoA1 concentration in these HDL subspecies but to a lesser degree and the difference was

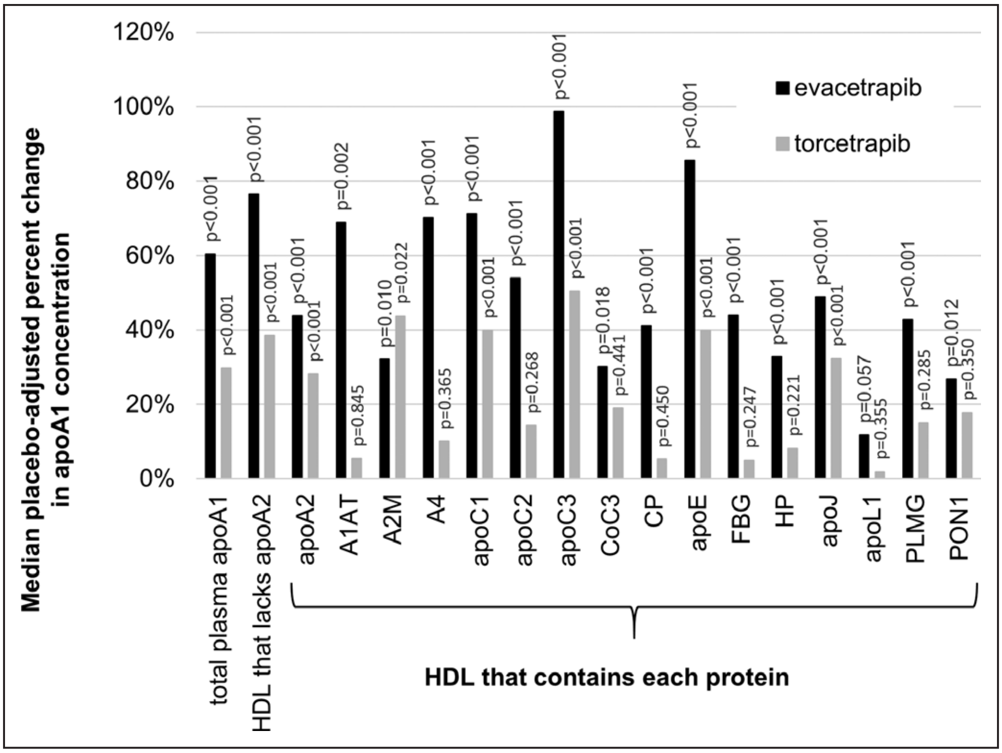

Figure 2. Both torcetrapib and evacetrapib nominally increased the apoA1 concentrations of all HDL (high-density lipoprotein) subspecies studied, except apoL1, but to different degrees.

Bars are median placebo-adjusted percent change. *P* values are for effect of drug compared with placebo and are FDR (false detection rate)-adjusted. A1AT indicates α-1-antitrypsin; A2M, α-2-macroglobulin; CoC3, complement C3; CP, ceruloplasmin; FBG, fibrinogen; HP, haptoglobin; PLMG, plasminogen; and PON-1, paraoxonase-1.

not statistically significant compared with placebo. HDL that contains apoL1 has a proteomic profile unlike any other subspecies.[39] Compared with placebo, neither drug significantly affected the concentration of apoA1 in HDL that contains apoL1 (Figure 2, Tables S1 and S2).

CETP Inhibitor Treatment Changes the Proportion of Total HDL Comprised by the HDL Subspecies

In addition to examining changes in absolute concentration of apoA1 in HDL subspecies, we also examined changes in the proportion of total HDL comprised by each of the HDL subspecies (Figure 4). Increases in apoA1 concentration in an HDL subspecies as a proportion of total apoA1 indicates a relative enrichment of this subspecies (ie, more of the total HDL is comprised of this subspecies) while decreases indicate a relative depletion. Both torcetrapib and evacetrapib increased the proportion of total apoA1 that is comprised by HDL that lacks apoA2 and HDL that contains apoC1, apoC3, or apoE (FDR-adjusted $P<0.05$). The proportion of total apoA1 comprised HDL that contains apoC3 was increased the most by both treatments (increased by 16.5% and 18.4%, respectively). Both drugs decreased the proportion of total apoA1 that is comprised by HDL that contains apoL1 (−10% and −30%, respectively) or haptoglobin (−8% and −13%, respectively). Evacetrapib decreased the proportion of total apoA1 that is comprised by HDL that contains apoC2, apoJ, α-1-antitrypsin, α-2-macroglobulin, ceruloplasmin, complement C3, fibrinogen, plasminogen, or PON-1 to varying degrees, ranging from −1% to −21% apoE (FDR-adjusted $P<0.05$).

Subgroup and Sensitivity Analyses

Although this study was not powered for subgroup analyses, we looked for evidence that drug response may have been associated with sex, age, or comorbidity but found no statistically significant interactions. In sensitivity analyses we compared the *P* values obtained by *t* test, by *t* test after log-transformation, and by Wilcoxon rank-sum test. We also ran the models after removing outliers beyond 3 SDs from the mean. We found no deviations from the patterns of statistical significance that we have reported.

DISCUSSION

Treatment with the CETP inhibitors, evacetrapib and torcetrapib, increased total apoA1 concentration, and the apoA1 concentration of 17 HDL subspecies to markedly different degrees. The 17 HDL subspecies studied differ in their association with CHD risk: some are associated with lower risk, some have no association with CHD, and some are

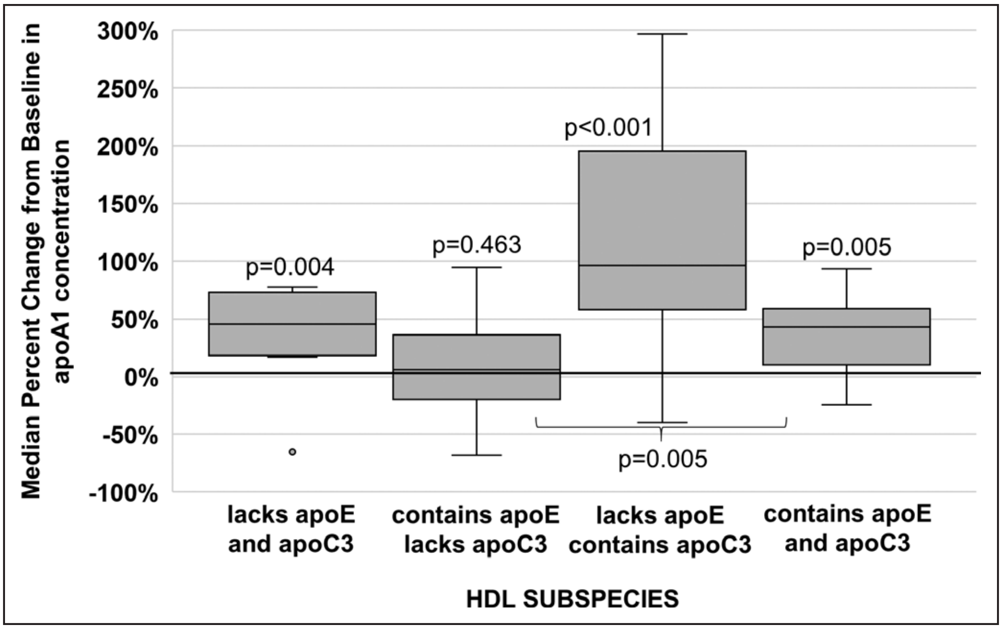

Figure 3. Torcetrapib increases the apoA1 concentrations of subspecies of HDL (high-density lipoprotein) that lacks both apoE and apoC3, the subspecies of HDL that contains both apoE and apoC3, and the subspecies of HDL that contains apoC3 but not apoE. Torcetrapib had no effect on the subspecies of HDL that contains apoE but not apoC3. Atorvastatin was given as a background medication to all groups to control and equalize LDL (low-density lipoprotein)-cholesterol. N=14. *P* values above plots are for change from baseline. *P* value below bracket compares HDL that contains both apoE and apoC3 to HDL that contains apoE and lacks apoC3.

associated with higher risk.[40] ApoA1 concentration in HDL that contains apoC3, a subspecies of HDL associated with higher CHD risk, was increased the most of all HDL subspecies. ApoA1 concentration in protective HDL that contains apoE was increased, but we found that it was its nonprotective subtype that also contains apoC3 that increased. Pharmacological increases in HDL concentration would not be beneficial if they are achieved with dysfunctional types of HDL that are associated with null or higher risk of CHD. The relationship between these changes in HDL subspecies concentration and disease risk cannot be determined directly from this study, but inference can be drawn from associations between the HDL subspecies and disease risk in previous observational studies.

Evacetrapib treatment increases plasma total apoC3 by 50%.[33] However, plasma total apoC3 does not correlate strongly with the concentration of apoA1 in HDL that contains apoC3.[40] Therefore, plasma total apoC3 is not a reliable marker of the concentration of apoA1 in HDL that contains apoC3. HDL that contains apoC3, whether quantified by apoA1 or cholesterol concentration, is a dysfunctional subspecies[37] associated with higher risk of CHD independent of plasma apoC3 concentration.[38,40–44] The present study showed that the type of HDL increased the most by evacetrapib and torcetrapib is HDL that contains apoC3. A recent study of 4 US cohorts found that an increase in apoA1 concentration in HDL that contains apoC3 of 1 SD was associated with a 9% increase in risk of incident coronary heart disease.[38] In the present study, the increase in concentration of apoA1 in HDL that contains apoC3 was ≈1 SD of the baseline concentration in each group. Therefore, considered in isolation, we may expect a 9% increase in risk to result solely from the increase in HDL that contains apoC3 elicited by the drugs. Furthermore, the copresence of apoC3 on HDL that contains apoE nullifies the beneficial effect of apoE on HDL metabolism in relation to reverse cholesterol transport and CVD.[37]

CETP inhibitor treatment significantly increased other HDL subspecies associated with higher risk of CHD: HDL that contains α-2-macroglobulin (HR [hazard ratio]=1.09), complement C3 (HR=1.11), or plasminogen (HR=1.06).[40] Increases in the concentrations of these 3 subspecies may increase CHD risk. Alpha-2-macroglobulin is a general protease inhibitor, may interfere with the action of plasmin,[49] and inactivates LCAT,[50] actions that inhibit clot dissolving and cholesterol transport. Plasminogen is the inactive precursor of plasmin, a protein that inhibits HDL-induced cholesterol efflux from macrophages.[51] Plasma complement C3 is associated with higher risk of CHD.[52,53] However, it is not yet known if these subspecies themselves are the causal factor that increases CHD risk, are simply a marker of risk, or instead are produced in response to chronic disease. For example, it is not established whether complement C3 acts to promote or reduce atherothrombosis.[54] It has been suggested both that HDL may be transporting biologically active molecules like complement C3 to active lesions and facilitating their action by acting as a platform; or sequestering CoC3 and other promoters of inflammation.[55] More research is needed to answer these important questions.

The CETP inhibitors also increased apoA1 concentration in HDL that contains apoE as well as apoA1 in HDL

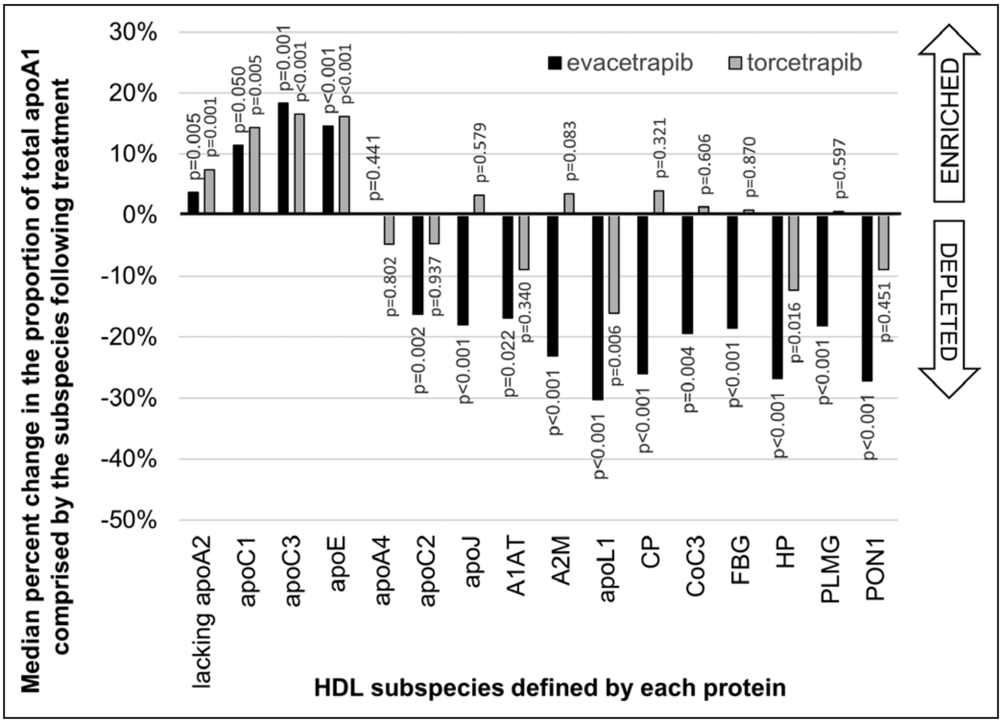

Figure 4. Both torcetrapib and evacetrapib increase the proportion of total apoA1 comprised by HDL (high-density lipoprotein) that lacks apoA2 and HDL that contains apoC1, apoC3, or apoE.

Both drugs decrease the proportion of total apoA1 that is comprised by HDL that contains apoL1 or haptoglobin (HP). Evacetrapib decreases the proportion of total apoA1 that is comprised by HDL that contains apoC2, apoJ, α-1-antitrypsin (A1AT), α-2-macroglobulin (A2M), ceruloplasmin (CP), complement C3 (CoC3), fibrinogen (FBG), plasminogen (PLMG), or paraoxonase-1 (PON-1). All *P* values are from Wilcoxon rank-sum test and are FDR (false detection rate)-adjusted.

that contains apoC1, subspecies associated with protection against CHD.[40] HDL that contains apoE has a unique metabolism that supports reverse cholesterol transport with a disproportionately high secretion into the circulation, active expansion while circulating, and quick clearance from the circulation. However, these metabolic steps are strongly attenuated by the copresence of apoC3.[37] The antagonistic relationship between apoC3 and apoE has been well-documented in apoB-containing lipoproteins in humans, in vitro, and in animal models.[56–63] Similar to the effects observed in the apoB-containing lipoproteins, apoC3 may block the apoE-enhanced clearance of HDL via liver receptors. We found that the torcetrapib-mediated increases of apoA1 in HDL that contains apoE were in the subtype that also contains apoC3, a subspecies that has an elevated risk similar to HDL that contains apoC3. There was no effect on HDL that contains apoE and lacks apoC3 (Figure 3), the apoE-containing subspecies associated with lower risk. Therefore, the increase in HDL that contains apoE may be entirely of the deleterious form that also contains apoC3. ApoC1 increases ABCA1 (ATP-binding cassette subfamily A member 1)-mediated cholesterol efflux from macrophages,[64,65] activates LCAT,[66] and inhibits CETP,[67,68] all processes that facilitate the maturation of HDL from small discoidal nascent HDL to larger spherical HDL. These functions support mechanistically the protective association between HDL that contains apoC1 and CHD. However, it is not yet known if the copresence of apoC3 on HDL that contains apoC1 could weaken the association with lower CHD risk as the copresence of apoC3 does for HDL that contains apoE.

Increases in the HDL subspecies defined by proteins linked to functions other than lipid metabolism may also have effects on CHD risk. Our findings show that most of these subspecies are associated with nullification of the protective effect of total HDL.[40] The hazard ratios per 1 SD increase in HDL that lacks any one of them are similar to total HDL (≈0.8) while they are null for HDL that contains each one (≈1.0). The lack of effect on HDL that contains apoL1 by either drug is unique among the HDL subspecies. HDL that contains apoL1 has a unique proteomic profile, which suggests that it comprised a specialized category of HDL overlapping minimally with and likely functioning independently from the other HDL subspecies we studied.[39] Indeed, HDL that contains apoL1 has a well-characterized specialized function in innate immunity as the trypanosome lytic factor[69,70] and may have unique origins and metabolism. The clinical significance of this lack of effect, however, is presently unknown.

We know that risk associated with the HDL subspecies changes with their absolute concentration.[40] It is also possible that it is the proportion of each subspecies relative to total HDL that drives the association with CVD risk. Thus, changes in proportionality may alter risk.

In the present study, while CETP inhibition increased the concentrations of all HDL subspecies to varying degrees, it proportionally enriched some fractions while it proportionally depleted others. Both drugs proportionally enriched total HDL with HDL subspecies that contain apoA2, apoC1, apoC3, or apoE while they proportionally depleted total HDL of HDL that contains apoL1 and haptoglobin. Evacetrapib also proportionally depleted total HDL of all the other subspecies that we studied except for apoA4. Interestingly, these results are in line with those reported by Asztalos and colleagues in a study of genetically CETP-deficient humans.[71] In individuals homozygous for mutations that depleted CETP, HDL-C, and apoA1 concentration was higher compared with the control group with no mutation (300% and 75%, respectively). Concentration of apoC1, apoC2, apoC3, and apoE in HDL was also higher in these CETP loss of function individuals by 11% to 43% while apoA4 did not differ between the groups. Other proteins were not quantified.

The mechanism(s) by which the CETP inhibitors elicit differential changes to the apoA1 concentrations of the HDL subspecies are not known. There are qualities to individual HDL particles that allow these subspecies to exist. For example, there is more than enough apoC3 in plasma to associate with all HDL particles, yet apoC3 resides on only a small minority of HDLs. We speculate that these unique qualities include core lipid content, phospholipid composition, and interaction with other proteins present on the HDL particle. It may be that the greater increases in the concentrations of apoA1 in HDL subspecies defined by proteins involved in lipid metabolism are driven by the increase in the lipid content and in the size of the HDL particles elicited by CETP inhibition providing a more favorable environment for these proteins. Perhaps other subspecies increase to a smaller degree because they are attracted to their HDLs through compositional aspects other than lipid content and size, such as protein content or phospholipid quality.

LIMITATIONS

The changes in HDL subspecies elicited by torcetrapib and evacetrapib in this study population may not be representative of the effects in other study populations. This study should be repeated in populations with diverse demographics and disease states. Similarly, this study would benefit from including other doses of the CETP inhibitor drugs. Yvan-Charvet et al[34] found that the dose of torcetrapib used in this study (60 mg/d) did not change per-particle cholesterol efflux or functional capacity of the HDL particles while 120 mg/d increased these potentially protective mechanisms.[34] In a study of individuals from the ACCENTUATE trial from which our samples were obtained, Nicholls et al[33] found that global, ABCA1-mediated, and non-ABCA1-mediated cholesterol efflux by total HDL were all higher after evacetrapib treatment. Study of the efflux capacity of each HDL subspecies would enhance the interpretation of our results and may help explain the paradox of increased efflux without clinical impact. Furthermore, our conclusions are limited by a lack of knowledge about how these changes in HDL subspecies will alter CVD risk and the effects of potential interplay among HDL subspecies. Additionally, with over 200 proteins identified in association with HDL by proteomics techniques (https://homepages.uc.edu/~davidswm/HDLproteome.html), there are potentially many other HDL subspecies beyond those we studied that may have significant associations with CHD risk that would be important to study for the effect of CETP inhibition. This study focused on the panel of 17 HDL subspecies for which we had validated assays and had previously studied for associations with CHD.

In conclusion, CETP inhibitors evacetrapib and torcetrapib increase the apoA1 concentration of all 17 HDL subspecies studied, and the increase varies greatly in degree and significance across subspecies. Both drugs increased HDL subspecies defined by apoC3 by the largest percentage of all, a dysfunctional HDL subspecies associated with higher risk of CHD. Both drugs increased HDL that contains apoE, but only its subtype that also contains apoC3, which is associated with higher risk. Three other detrimental HDL subspecies were also increased: HDL that contains α-2-macroglobulin, complement C3, and plasminogen. Taken together, these changes result in an altered HDL profile that may be less protective, which could contribute to the lack of clinical benefit from pharmaceutical CETP inhibition. Further study of the mechanism by which the subspecies respond to these treatments may help design treatments targeted to increase only the beneficial subspecies of HDL to reduce risk of CHD.

ARTICLE INFORMATION

Received September 1, 2021; accepted November 29, 2021.

Affiliations

Department of Nutrition, Harvard T.H. Chan School of Public Health, Boston MA (J.D.F., F.M.S.). Channing Laboratory, Department of Medicine, Brigham and Women's Hospital, Harvard Medical School, Boston, MA (F.M.S.). Eli Lilly & Company, Indianapolis, IN (G.R.). Victorian Heart Institute, Monash University, Victoria, Australia (S.J.N.). Pfizer, Inc, Cambridge, MA (R.D., S.C.-G.).

Sources of Funding

This work was funded by Eli Lilly & Company, Indianapolis, IN, and Pfizer, Inc, New York, NY.

Disclosures

J.D. Furtado and F.M. Sacks reports inventors on 2 patents held by Harvard University related to HDL (high-density lipoprotein) that contains apoC3 as a risk factor for coronary heart disease (CHD) and the measurement of HDL subspecies. F.M. Sacks was a consultant and expert witness for Pfizer and a consultant for DalCor, AstraZeneca, CSL Behring, and Inventiva. G. Ruotolo reports full-time employee of Eli Lilly & Company with significant ownership interest. R. Dullea and S. Carvajal-Gonzalez reports full-time employees of Pfizer Inc, with significant ownership interest. The other author reports no conflicts.

Supplemental Material

Supplemental Methods
Tables S1–S2
Major Resources Table

REFERENCES

1. National Cholesterol Education Program Expert Panel on Detection E, Treatment of High Blood Cholesterol in A. Third Report of the National Cholesterol Education Program (NCEP) expert panel on detection, evaluation, and treatment of high blood cholesterol in adults (Adult Treatment Panel III) final report. *Circulation*. 2002;106:3143-3421. doi: 10.1161/circ.106.25.3143
2. Rye KA, Barter PJ. Cardioprotective functions of HDLs. *J Lipid Res*. 2014;55:168–179. doi: 10.1194/jlr.R039297
3. Zanoni P, Khetarpal SA, Larach DB, Hancock-Cerutti WF, Millar JS, Cuchel M, DerOhannessian S, Kontush A, Surendran P, Saleheen D, et al; CHD Exome+ Consortium; CARDIoGRAM Exome Consortium; Global Lipids Genetics Consortium. Rare variant in scavenger receptor BI raises HDL cholesterol and increases risk of coronary heart disease. *Science*. 2016;351:1166–1171. doi: 10.1126/science.aad3517
4. Wilkins JT, Ning H, Stone NJ, Criqui MH, Zhao L, Greenland P, Lloyd-Jones DM. Coronary heart disease risks associated with high levels of HDL cholesterol. *J Am Heart Assoc*. 2014;3:e000519. doi: 10.1161/JAHA.113.000519
5. Madsen CM, Varbo A, Nordestgaard BG. Extreme high high-density lipoprotein cholesterol is paradoxically associated with high mortality in men and women: two prospective cohort studies. *Eur Heart J*. 2017;38:2478–2486. doi: 10.1093/eurheartj/ehx163
6. Brown ML, Inazu A, Hesler CB, Agellon LB, Mann C, Whitlock ME, Marcel YL, Milne RW, Koizumi J, Mabuchi H. Molecular basis of lipid transfer protein deficiency in a family with increased high-density lipoproteins. *Nature*. 1989;342:448–451. doi: 10.1038/342448a0
7. Barter PJ, Brewer HB Jr, Chapman MJ, Hennekens CH, Rader DJ, Tall AR. Cholesteryl ester transfer protein: a novel target for raising HDL and inhibiting atherosclerosis. *Arterioscler Thromb Vasc Biol*. 2003;23:160–167. doi: 10.1161/01.atv.0000054658.91146.64
8. Marotti KR, Castle CK, Boyle TP, Lin AH, Murray RW, Melchior GW. Severe atherosclerosis in transgenic mice expressing simian cholesteryl ester transfer protein. *Nature*. 1993;364:73–75. doi: 10.1038/364073a0
9. Westerterp M, van der Hoogt CC, de Haan W, Offerman EH, Dallinga-Thie GM, Jukema JW, Havekes LM, Rensen PC. Cholesteryl ester transfer protein decreases high-density lipoprotein and severely aggravates atherosclerosis in APOE*3-Leiden mice. *Arterioscler Thromb Vasc Biol*. 2006;26:2552–2559. doi: 10.1161/01.ATV.0000243925.65265.3c
10. de Vries-van der Weij J, Zadelaar S, Toet K, Havekes LM, Kooistra T, Rensen PC. Human CETP aggravates atherosclerosis by increasing VLDL-cholesterol rather than by decreasing HDL-cholesterol in APOE*3-Leiden mice. *Atherosclerosis*. 2009;206:153–158. doi: 10.1016/j.atherosclerosis.2009.02.038
11. Rittershaus CW, Miller DP, Thomas LJ, Picard MD, Honan CM, Emmett CD, Pettey CL, Adari H, Hammond RA, Beattie DT, et al. Vaccine-induced antibodies inhibit CETP activity in vivo and reduce aortic lesions in a rabbit model of atherosclerosis. *Arterioscler Thromb Vasc Biol*. 2000;20:2106–2112. doi: 10.1161/01.atv.20.9.2106
12. Okamoto H, Yonemori F, Wakitani K, Minowa T, Maeda K, Shinkai H. A cholesteryl ester transfer protein inhibitor attenuates atherosclerosis in rabbits. *Nature*. 2000;406:203–207. doi: 10.1038/35018119
13. Hayek T, Masucci-Magoulas L, Jiang X, Walsh A, Rubin E, Breslow JL, Tall AR. Decreased early atherosclerotic lesions in hypertriglyceridemic mice expressing cholesteryl ester transfer protein transgene. *J Clin Invest*. 1995;96:2071–2074. doi: 10.1172/JCI118255
14. Harder C, Lau P, Meng A, Whitman SC, McPherson R. Cholesteryl ester transfer protein (CETP) expression protects against diet induced atherosclerosis in SR-BI deficient mice. *Arterioscler Thromb Vasc Biol*. 2007;27:858–864. doi: 10.1161/01.ATV.0000259357.42089.dc
15. Föger B, Chase M, Amar MJ, Vaisman BL, Shamburek RD, Paigen B, Fruchart-Najib J, Paiz JA, Koch CA, Hoyt RF, et al. Cholesteryl ester transfer protein corrects dysfunctional high density lipoproteins and reduces aortic atherosclerosis in lecithin cholesterol acyltransferase transgenic mice. *J Biol Chem*. 1999;274:36912–36920. doi: 10.1074/jbc.274.52.36912
16. Groener JE, Van Rozen AJ, Erkelens DW. Cholesteryl ester transfer activity. Localization and role in distribution of cholesteryl ester among lipoproteins in man. *Atherosclerosis*. 1984;50:261–271. doi: 10.1016/0021-9150(84)90074-1
17. Yamashita S, Hui DY, Wetterau JR, Sprecher DL, Harmony JA, Sakai N, Matsuzawa Y, Tarui S. Characterization of plasma lipoproteins in patients heterozygous for human plasma cholesteryl ester transfer protein (CETP) deficiency: plasma CETP regulates high-density lipoprotein concentration and composition. *Metabolism*. 1991;40:756–763. doi: 10.1016/0026-0495(91)90097-g
18. Inazu A, Brown ML, Hesler CB, Agellon LB, Koizumi J, Takata K, Maruhama Y, Mabuchi H, Tall AR. Increased high-density lipoprotein levels caused by a common cholesteryl-ester transfer protein gene mutation. *N Engl J Med*. 1990;323:1234–1238. doi: 10.1056/NEJM199011013231803
19. Ordovas JM, Cupples LA, Corella D, Otvos JD, Osgood D, Martinez A, Lahoz C, Coltell O, Wilson PW, Schaefer EJ. Association of cholesteryl ester transfer protein-TaqIB polymorphism with variations in lipoprotein subclasses and coronary heart disease risk: the Framingham study. *Arterioscler Thromb Vasc Biol*. 2000;20:1323–1329. doi: 10.1161/01.atv.20.5.1323
20. Barzilai N, Atzmon G, Schechter C, Schaefer EJ, Cupples AL, Lipton R, Cheng S, Shuldiner AR. Unique lipoprotein phenotype and genotype associated with exceptional longevity. *JAMA*. 2003;290:2030–2040. doi: 10.1001/jama.290.15.2030
21. Koropatnick TA, Kimbell J, Chen R, Grove JS, Donlon TA, Masaki KH, Rodriguez BL, Willcox BJ, Yano K, Curb JD. A prospective study of high-density lipoprotein cholesterol, cholesteryl ester transfer protein gene variants, and healthy aging in very old Japanese-american men. *J Gerontol A Biol Sci Med Sci*. 2008;63:1235–1240. doi: 10.1093/gerona/63.11.1235
22. Millwood IY, Bennett DA, Holmes MV, Boxall R, Guo Y, Bian Z, Yang L, Sansome S, Chen Y, Du H, et al; China Kadoorie Biobank Collaborative Group. Association of CETP Gene Variants With Risk for Vascular and Nonvascular Diseases Among Chinese Adults. *JAMA Cardiol*. 2018;3:34–43. doi: 10.1001/jamacardio.2017.4177
23. Nomura A, Won HH, Khera AV, Takeuchi F, Ito K, McCarthy S, Emdin CA, Klarin D, Natarajan P, Zekavat SM, et al. Protein-truncating variants at the cholesteryl ester transfer protein gene and risk for coronary heart disease. *Circ Res*. 2017;121:81–88. doi: 10.1161/CIRCRESAHA.117.311145
24. Agerholm-Larsen B, Nordestgaard BG, Steffensen R, Jensen G, Tybjaerg-Hansen A. Elevated HDL cholesterol is a risk factor for ischemic heart disease in white women when caused by a common mutation in the cholesteryl ester transfer protein gene. *Circulation*. 2000;101:1907–1912. doi: 10.1161/01.cir.101.16.1907
25. Johannsen TH, Frikke-Schmidt R, Schou J, Nordestgaard BG, Tybjærg-Hansen A. Genetic inhibition of CETP, ischemic vascular disease and mortality, and possible adverse effects. *J Am Coll Cardiol*. 2012;60:2041–2048. doi: 10.1016/j.jacc.2012.07.045
26. Webb TR, Erdmann J, Stirrups KE, Stitziel NO, Masca NG, Jansen H, Kanoni S, Nelson CP, Ferrario PG, König IR, et al; Wellcome Trust Case Control Consortium; MORGAM Investigators; Myocardial Infarction Genetics and CARDIoGRAM Exome Consortia Investigators. Systematic evaluation of Pleiotropy identifies 6 further Loci associated with coronary artery disease. *J Am Coll Cardiol*. 2017;69:823–836. doi: 10.1016/j.jacc.2016.11.056
27. Voight BF, Peloso GM, Orho-Melander M, Frikke-Schmidt R, Barbalic M, Jensen MK, Hindy G, Hólm H, Ding EL, Johnson T, et al. Plasma HDL cholesterol and risk of myocardial infarction: a mendelian randomisation study. *Lancet*. 2012;380:572–580. doi: 10.1016/S0140-6736(12)60312-2
28. Willer CJ, Schmidt EM, Sengupta S, Peloso GM, Gustafsson S, Kanoni S, Ganna A, Chen J, Buchkovich ML, Mora S, et al; Global Lipids Genetics Consortium. Discovery and refinement of loci associated with lipid levels. *Nat Genet*. 2013;45:1274–1283. doi: 10.1038/ng.2797
29. Holmes MV, Asselbergs FW, Palmer TM, Drenos F, Lanktree MB, Nelson CP, Dale CE, Padmanabhan S, Finan C, Swerdlow DI, et al; UCLEB consortium. Mendelian randomization of blood lipids for coronary heart disease. *Eur Heart J*. 2015;36:539–550. doi: 10.1093/eurheartj/eht571
30. White J, Swerdlow DI, Preiss D, Fairhurst-Hunter Z, Keating BJ, Asselbergs FW, Sattar N, Humphries SE, Hingorani AD, Holmes MV. Association of lipid fractions with risks for coronary artery disease and diabetes. *JAMA Cardiol*. 2016;1:692–699. doi: 10.1001/jamacardio.2016.1884
31. Agerholm-Larsen B, Tybjaerg-Hansen A, Schnohr P, Steffensen R, Nordestgaard BG. Common cholesteryl ester transfer protein mutations, decreased HDL cholesterol, and possible decreased risk of ischemic heart disease: The Copenhagen City Heart Study. *Circulation*. 2000;102:2197–2203. doi: 10.1161/01.cir.102.18.2197
32. Barter PJ, Caulfield M, Eriksson M, Grundy SM, Kastelein JJ, Komajda M, Lopez-Sendon J, Mosca L, Tardif JC, Waters DD, et al; ILLUMINATE Investigators. Effects of torcetrapib in patients at high risk for coronary events. *N Engl J Med*. 2007;357:2109–2122. doi: 10.1056/NEJMoa0706628
33. Nicholls SJ, Ray KK, Ballantyne CM, Beacham LA, Miller DL, Ruotolo G, Nissen SE, Riesmeyer JS; ACCENTUATE Investigators. Comparative effects of cholesteryl ester transfer protein inhibition, statin or ezetimibe on lipid factors: The ACCENTUATE trial. *Atherosclerosis*. 2017;261:12–18. doi: 10.1016/j.atherosclerosis.2017.04.008
34. Yvan-Charvet L, Matsuura F, Wang N, Bamberger MJ, Nguyen T, Rinninger F, Jiang XC, Shear CL, Tall AR. Inhibition of cholesteryl ester transfer protein by torcetrapib modestly increases macrophage cholesterol efflux to HDL. *Arterioscler Thromb Vasc Biol*. 2007;27:1132-1138. doi: 10.1161/ATVBAHA.106.138347

35. Bowman L, Hopewell JC, Chen F, Wallendszus K, Stevens W, Collins R, Wiviott SD, Cannon CP, Braunwald E, Sammons E, et al; HPS3/TIMI55–REVEAL Collaborative Group. Effects of Anacetrapib in patients with atherosclerotic vascular disease. *N Engl J Med*. 2017;377:1217–1227. doi: 10.1056/NEJMoa1706444
36. Melchior JT, Street SE, Andraski AB, Furtado JD, Sacks FM, Shute RL, Greve EI, Swertfeger DK, Li H, Shah AS, et al. Apolipoprotein A-II alters the proteome of human lipoproteins and enhances cholesterol efflux from ABCA1. *J Lipid Res*. 2017;58:1374–1385. doi: 10.1194/jlr.M075382
37. Morton AM, Koch M, Mendivil CO, Furtado JD, Tjønneland A, Overvad K, Wang L, Jensen MK, Sacks FM. Apolipoproteins E and CIII interact to regulate HDL metabolism and coronary heart disease risk. *JCI Insight*. 2018;3:98045. doi: 10.1172/jci.insight.98045
38. Jensen MK, Aroner SA, Mukamal KJ, Furtado JD, Post WS, Tsai MY, Tjønneland A, Polak JF, Rimm EB, Overvad K, et al. High-density lipoprotein subspecies defined by presence of Apolipoprotein C-III and incident coronary heart disease in four cohorts. *Circulation*. 2018;137:1364–1373. doi: 10.1161/CIRCULATIONAHA.117.031276
39. Furtado JD, Yamamoto R, Melchior JT, Andraski AB, Gamez-Guerrero M, Mulcahy P, He Z, Cai T, Davidson WS, Sacks FM. Distinct proteomic signatures in 16 HDL (High-Density Lipoprotein) subspecies. *Arterioscler Thromb Vasc Biol*. 2018;38:2827–2842. doi: 10.1161/ATVBAHA.118.311607
40. Sacks FM, Liang L, Furtado JD, Cai T, Davidson WS, He Z, McClelland RL, Rimm EB, Jensen MK. Protein-defined subspecies of HDLs (High-Density Lipoproteins) and differential risk of coronary heart disease in 4 prospective studies. *Arterioscler Thromb Vasc Biol*. 2020;40:2714–2727. doi: 10.1161/ATVBAHA.120.314609
41. Aroner SA, Furtado JD, Sacks FM, Tsai MY, Mukamal KJ, McClelland RL, Jensen MK. Apolipoprotein C-III and its defined lipoprotein subspecies in relation to incident diabetes: the Multi-Ethnic Study of Atherosclerosis. *Diabetologia*. 2019;62:981–992. doi: 10.1007/s00125-019-4847-8
42. Aroner SA, Koch M, Mukamal KJ, Furtado JD, Stein JH, Tattersall MC, McClelland RL, Jensen MK. High-Density Lipoprotein subspecies defined by Apolipoprotein C-III and subclinical atherosclerosis measures: MESA (The Multi-Ethnic Study of Atherosclerosis). *J Am Heart Assoc*. 2018;7:e007824. doi: 10.1161/JAHA.117.007824
43. Aroner SA, Yang M, Li J, Furtado JD, Sacks FM, Tjønneland A, Overvad K, Cai T, Jensen MK. Apolipoprotein C-III and High-Density Lipoprotein subspecies defined by Apolipoprotein C-III in relation to diabetes risk. *Am J Epidemiol*. 2017;186:736–744. doi: 10.1093/aje/kwx143
44. Yamamoto R, Sacks FM, Hu FB, Rosner B, Furtado JD, Aroner SA, Ferrannini E, Baldi S, Kozakova M, Balkau B, et al; RISC Investigators. High density lipoprotein with apolipoprotein C-III is associated with carotid intima-media thickness among generally healthy individuals. *Atherosclerosis*. 2018;269:92–99. doi: 10.1016/j.atherosclerosis.2017.12.029
45. Yamamoto R, Jensen MK, Aroner S, Furtado JD, Rosner B, Hu FB, Balkau B, Natali A, Ferrannini E, Baldi S, et al. HDL containing Apolipoprotein C-III is associated with insulin sensitivity: A Multicenter Cohort Study. *J Clin Endocrinol Metab*. 2021;106:e2928–e2940. doi: 10.1210/clinem/dgab234
46. Talayero B, Wang L, Furtado J, Carey VJ, Bray GA, Sacks FM. Obesity favors apolipoprotein E- and C-III-containing high density lipoprotein subfractions associated with risk of heart disease. *J Lipid Res*. 2014;55:2167–2177. doi: 10.1194/jlr.M042333
47. Storey JD, Tibshirani R. Statistical significance for genomewide studies. *Proc Natl Acad Sci USA*. 2003;100:9440–9445. doi: 10.1073/pnas.1530509100
48. Goeman JJ, Solari A. Multiple hypothesis testing in genomics. *Stat Med*. 2014;33:1946–1978. doi: 10.1002/sim.6082
49. Schaller J, Gerber SS. The plasmin-antiplasmin system: structural and functional aspects. *Cell Mol Life Sci*. 2011;68:785–801. doi: 10.1007/s00018-010-0566-5
50. Krimbou L, Marcil M, Davignon J, Genest J Jr. Interaction of lecithin:cholesterol acyltransferase (LCAT).alpha 2-macroglobulin complex with low density lipoprotein receptor-related protein (LRP). Evidence for an alpha 2-macroglobulin/LRP receptor-mediated system participating in LCAT clearance. *J Biol Chem*. 2001;276:33241–33248. doi: 10.1074/jbc.M100326200
51. Lindstedt L, Kovanen PT. Plasmin and kallikrein reduce HDL-induced cholesterol efflux from foam cells. *Biochem Biophys Res Commun*. 2000;277:552–557. doi: 10.1006/bbrc.2000.3704
52. Muscari A, Bozzoli C, Puddu GM, Sangiorgi Z, Dormi A, Rovinetti C, Descovich GC, Puddu P. Association of serum C3 levels with the risk of myocardial infarction. *Am J Med*. 1995;98:357–364. doi: 10.1016/S0002-9343(99)80314-3
53. Széplaki G, Prohászka Z, Duba J, Rugonfalvi-Kiss S, Karádi I, Kókai M, Kramer J, Füst G, Kleiber M, Romics L, et al. Association of high serum concentration of the third component of complement (C3) with pre-existing severe coronary artery disease and new vascular events in women. *Atherosclerosis*. 2004;177:383–389. doi: 10.1016/j.atherosclerosis.2004.07.022
54. Speidl WS, Kastl SP, Huber K, Wojta J. Complement in atherosclerosis: friend or foe? *J Thromb Haemost*. 2011;9:428–440. doi: 10.1111/j.1538-7836.2010.04172.x
55. Gordon SM, Remaley AT. High density lipoproteins are modulators of protease activity: implications in inflammation, complement activation, and atherothrombosis. *Atherosclerosis*. 2017;259:104–113. doi: 10.1016/j.atherosclerosis.2016.11.015
56. Zheng C, Khoo C, Ikewaki K, Sacks FM. Rapid turnover of apolipoprotein C-III-containing triglyceride-rich lipoproteins contributing to the formation of LDL subfractions. *J Lipid Res*. 2007;48:1190–1203. doi: 10.1194/jlr.P600011-JLR200
57. Aalto-Setälä K, Weinstock PH, Bisgaier CL, Wu L, Smith JD, Breslow JL. Further characterization of the metabolic properties of triglyceride-rich lipoproteins from human and mouse apoC-III transgenic mice. *J Lipid Res*. 1996;37:1802–1811.
58. Mendivil CO, Zheng C, Furtado J, Lel J, Sacks FM. Metabolism of very-low-density lipoprotein and low-density lipoprotein containing apolipoprotein C-III and not other small apolipoproteins. *Arterioscler Thromb Vasc Biol*. 2010;30:239–245. doi: 10.1161/ATVBAHA.109.197830
59. Clavey V, Lestavel-Delattre S, Copin C, Bard JM, Fruchart JC. Modulation of lipoprotein B binding to the LDL receptor by exogenous lipids and apolipoproteins CI, CII, CIII, and E. *Arterioscler Thromb Vasc Biol*. 1995;15:963–971. doi: 10.1161/01.atv.15.7.963
60. Zheng C, Khoo C, Furtado J, Sacks FM. Apolipoprotein C-III and the metabolic basis for hypertriglyceridemia and the dense low-density lipoprotein phenotype. *Circulation*. 2010;121:1722–1734. doi: 10.1161/CIRCULATIONAHA.109.875807
61. Sehayek E, Eisenberg S. Mechanisms of inhibition by apolipoprotein C of apolipoprotein E-dependent cellular metabolism of human triglyceride-rich lipoproteins through the low density lipoprotein receptor pathway. *J Biol Chem*. 1991;266:18259–18267.
62. Shelburne F, Hanks J, Meyers W, Quarfordt S. Effect of apoproteins on hepatic uptake of triglyceride emulsions in the rat. *J Clin Invest*. 1980;65:652–658. doi: 10.1172/JCI109710
63. de Silva HV, Lauer SJ, Wang J, Simonet WS, Weisgraber KH, Mahley RW, Taylor JM. Overexpression of human apolipoprotein C-III in transgenic mice results in an accumulation of apolipoprotein B48 remnants that is corrected by excess apolipoprotein E. *J Biol Chem*. 1994;269:2324–2335.
64. Swertfeger DK, Li H, Rebholz S, Zhu X, Shah AS, Davidson WS, Lu LJ. Mapping Atheroprotective Functions and Related Proteins/Lipoproteins in Size Fractionated Human Plasma. *Mol Cell Proteomics*. 2017;16:680–693. doi: 10.1074/mcp.M116.066290
65. Smith LE, Segrest JP, Davidson WS. Helical domains that mediate lipid solubilization and ABCA1-specific cholesterol efflux in apolipoproteins C-I and A-II. *J Lipid Res*. 2013;54:1939–1948. doi: 10.1194/jlr.M037903
66. Soutar AK, Garner CW, Baker HN, Sparrow JT, Jackson RL, Gotto AM, Smith LC. Effect of the human plasma apolipoproteins and phosphatidylcholine acyl donor on the activity of lecithin: cholesterol acyltransferase. *Biochemistry*. 1975;14:3057–3064. doi: 10.1021/bi00685a003
67. de Barros JP, Boualam A, Gautier T, Dumont L, Vergès B, Masson D, Lagrost L. Apolipoprotein CI is a physiological regulator of cholesteryl ester transfer protein activity in human plasma but not in rabbit plasma. *J Lipid Res*. 2009;50:1842–1851. doi: 10.1194/jlr.M800588-JLR200
68. Gautier T, Masson D, de Barros JP, Athias A, Gambert P, Aunis D, Metz-Boutigue MH, Lagrost L. Human apolipoprotein C-I accounts for the ability of plasma high density lipoproteins to inhibit the cholesteryl ester transfer protein activity. *J Biol Chem*. 2000;275:37504–37509. doi: 10.1074/jbc.M007210200
69. Thomson R, Samanovic M, Raper J. Activity of trypanosome lytic factor: a novel component of innate immunity. *Future Microbiol*. 2009;4:789–796. doi: 10.2217/fmb.09.57
70. Catapano AL, Pirillo A, Bonacina F, Norata GD. HDL in innate and adaptive immunity. *Cardiovasc Res*. 2014;103:372–383. doi: 10.1093/cvr/cvu150
71. Asztalos BF, Horvath KV, Kajinami K, Nartsupha C, Cox CE, Batista M, Schaefer EJ, Inazu A, Mabuchi H. Apolipoprotein composition of HDL in cholesteryl ester transfer protein deficiency. *J Lipid Res*. 2004;45:448–455. doi: 10.1194/jlr.M300198-JLR200
