# Supplementary material for: Lipid-lowering effect of combined therapy with high-intensity statins and CETP inhibitors: a Systematic Review and meta-analysis
Source: Front Endocrinol (Lausanne). 2025 May 1;16:1512670. doi: 10.3389/fendo.2025.1512670 (PMC12078159; doi:10.3389/fendo.2025.1512670)
Supplement: Supplementary file 1 [file DataSheet1.zip › Raw Data/Raw Data/Original Documentation Fruit/Kastelein2007✔.pdf]

ORIGINAL ARTICLE

Effect of Torcetrapib on Carotid Atherosclerosis in Familial Hypercholesterolemia

John J.P. Kastelein, M.D., Ph.D., Sander I. van Leuven, M.D., Leslie Burgess, M.D., Greg W. Evans, M.A., Jan A. Kuivenhoven, Ph.D., Philip J. Barter, M.D., Ph.D., James H. Revkin, M.D., Diederick E. Grobbee, M.D., Ph.D., Ward A. Riley, Ph.D., Charles L. Shear, Dr.P.H., William T. Duggan, Ph.D., and Michiel L. Bots, M.D., Ph.D., for the RADIANCE 1 Investigators*

ABSTRACT

BACKGROUND

Torcetrapib, an inhibitor of cholesteryl ester transfer protein, may reduce atherosclerotic vascular disease by increasing levels of high-density lipoprotein (HDL) cholesterol.

METHODS

A total of 850 patients with heterozygous familial hypercholesterolemia underwent B-mode ultrasonography at baseline and at follow-up to measure changes in carotid intima–media thickness. The patients completed an atorvastatin run-in period and were subsequently randomly assigned to receive either atorvastatin monotherapy or atorvastatin combined with 60 mg of torcetrapib for 2 years.

RESULTS

After 24 months, in the atorvastatin-only group, the mean (±SD) HDL cholesterol level was 52.4±13.5 mg per deciliter and the mean low-density lipoprotein (LDL) cholesterol level was 143.2±42.2 mg per deciliter, as compared with 81.5±22.6 mg per deciliter and 115.1±48.5 mg per deciliter, respectively, in the torcetrapib–atorvastatin group. During the study, average systolic blood pressure increased by 2.8 mm Hg in the torcetrapib–atorvastatin group, as compared with the atorvastatin-only group. The increase in maximum carotid intima–media thickness, the primary measure of efficacy, was 0.0053±0.0028 mm per year in the atorvastatin-only group and 0.0047±0.0028 mm per year in the torcetrapib–atorvastatin group (P=0.87). The secondary efficacy measure, annualized change in mean carotid intima–media thickness for the common carotid artery, indicated a decrease of 0.0014 mm per year in the atorvastatin-only group, as compared with an increase of 0.0038 mm per year in the torcetrapib–atorvastatin group (P=0.005).

CONCLUSIONS

In patients with familial hypercholesterolemia, the use of torcetrapib with atorvastatin, as compared with atorvastatin alone, did not result in further reduction of progression of atherosclerosis, as assessed by a combined measure of carotid arterial-wall thickness, and was associated with progression of disease in the common carotid segment. These effects occurred despite a large increase in HDL cholesterol levels and a substantial decrease in levels of LDL cholesterol and triglycerides. (ClinicalTrials.gov number, NCT00136981.)

From the Academic Medical Center, University of Amsterdam, Amsterdam (J.J.P.K., S.I.L., J.A.K.); Tygerberg Hospital and Stellenbosch University, Tygerberg, South Africa (L.B.); Wake Forest University, Winston-Salem, NC (G.W.E., W.A.R.); the Heart Research Institute, Sydney (P.J.B.); Pfizer, New London, CT (J.H.R., C.L.S., W.T.D.); and the Julius Center for Health Sciences and Primary Care, University Medical Center, Utrecht, the Netherlands (D.E.G., M.L.B.). Address reprint requests to Dr. Kastelein at the Department of Vascular Medicine, Academic Medical Center, Meibergdreef 9, Room F4-159.2, 1105 AZ, Amsterdam, the Netherlands, or at j.j.kastelein@amc.uva.nl.

Drs. Kastelein and Bots contributed equally to this article.

*Investigators and committees of the Rating Atherosclerotic Disease Change by Imaging with a New CETP Inhibitor (RADIANCE 1) trial are listed in the Appendix.

This article (10.1056/NEJMoa071359) was published at www.nejm.org on March 26, 2007.

N Engl J Med 2007;356:1620-30.
Copyright © 2007 Massachusetts Medical Society.

The New England Journal of Medicine
Downloaded from nejm.org on August 5, 2024. For personal use only.
No other uses without permission. Copyright © 2007 Massachusetts Medical Society. All rights reserved.

Guidelines for the prevention and management of cardiovascular disease focus on reducing levels of low-density lipoprotein (LDL) cholesterol by means of hydroxymethylglutaryl coenzyme A (HMG-CoA) reductase inhibitors (collectively referred to as statins).[1,2] However, recent meta-analyses have shown that even with the most aggressive treatment,[3,4] these drugs reduce the risk of a major coronary event by only 30%.[5] This finding, combined with an estimation that mortality from cardiovascular causes will increase worldwide by 90% by the year 2020, as compared with that in 1990,[6] illustrates the need for new efficacious treatments. A review of four large, prospective epidemiologic studies has shown that an increase of 1 mg per deciliter (0.03 mmol per liter) in high-density lipoprotein (HDL) cholesterol was associated with a 2 to 3% reduction in the risk of cardiovascular disease.[7] Moreover, HDL cholesterol levels remain predictive of the risk of recurrent cardiovascular disease in patients who have reached LDL cholesterol levels below 70 mg per deciliter (1.8 mmol per liter) with intensive statin treatment.[8]

During the past few years, attempts to raise HDL cholesterol levels have been particularly successful with small-molecule inhibitors of cholesteryl ester transfer protein (CETP).[9,10] By blocking the CETP-mediated transfer of cholesteryl ester from HDL cholesterol to apolipoprotein-B–containing lipoproteins and the simultaneous transfer of triglycerides in the opposite direction, torcetrapib is very effective at raising HDL cholesterol levels.[10] Indeed, elevated CETP levels were shown to be associated with an increased risk of future coronary artery disease in apparently healthy subjects.[11] Furthermore, the inhibition of CETP in rabbit models of atherosclerosis dramatically reduced the extent of disease.[12] It is not known, however, whether CETP inhibition attenuates atherosclerosis in humans. Since new lipid-modulating drugs will be primarily used in addition to evidence-based lowering of LDL cholesterol, torcetrapib has been developed for use in combination with atorvastatin. In this setting, torcetrapib not only increased levels of HDL cholesterol and apolipoprotein A-I but also decreased levels of LDL cholesterol and apolipoprotein B-100 (the latter especially at higher doses) and also showed favorable effects on increasing the size of both HDL and LDL particles.[10]

In our study, we used a combination of torcetrapib and atorvastatin in patients with heterozygous familial hypercholesterolemia. The rationale for studying this target population was that mutations in the LDL-receptor gene are associated with decreased levels of HDL cholesterol,[13] smaller HDL particle size,[14] and increased levels of CETP.[15] Also, the progression of atherosclerosis in familial hypercholesterolemia is related to levels of both HDL cholesterol[16] and CETP.[17] Therefore, it was hypothesized that the use of torcetrapib would have distinct favorable effects in this group of patients. The aim of our study was to evaluate the effects of torcetrapib on carotid intima–media thickness, a surrogate marker for end points of cardiovascular disease in patients with familial hypercholesterolemia.

The results of this study need to be considered in light of the recent discontinuation of the large Investigation of Lipid Level Management to Understand Its Impact in Atherosclerotic Events (ILLUMINATE) trial (ClinicalTrials.gov number, NCT00134264), which showed an increase in all-cause mortality associated with torcetrapib.

METHODS

STUDY DESIGN

The Rating Atherosclerotic Disease Change by Imaging with a New CETP Inhibitor (RADIANCE 1) trial was a prospective, double-blind, randomized, multicenter, parallel-group study. The trial was designed by the academic investigators in collaboration with the study sponsor. The institutional review board at each participating center approved the protocol, and patients provided written informed consent. Patients were eligible for entry into the study if they had received a diagnosis of heterozygous familial hypercholesterolemia either by genotyping or by having met the diagnostic criteria outlined by the World Health Organization.[18]

During a run-in phase of 6 to 14 weeks, patients were counseled on therapeutic lifestyle changes[1] and were administered atorvastatin at a dose of 20, 40, or 80 mg, titrated at 4-week intervals, for up to three visits to reach target LDL cholesterol levels, as recommended by guidelines from the National Cholesterol Education Program,[1] or to reach the patient's maximum tolerated dose. At study entry, patients who were taking cholesterol absorption inhibitors or bile-acid binders were permitted to continue taking these medications, provided that the dose was not changed during

The New England Journal of Medicine
Downloaded from nejm.org on August 5, 2024. For personal use only.
No other uses without permission. Copyright © 2007 Massachusetts Medical Society. All rights reserved.

Table 1. Baseline Characteristics, Values at 24-Month Follow-up, and Changes from Baseline.*

Variable	Atorvastatin Monotherapy	Atorvastatin plus Torcetrapib	P Value
**All patients who underwent randomization**			
No. of patients	454	450	
Age — yr	45.2±12.9	46.8±12.0	0.06
Male sex — no. (%)	232 (51.1)	214 (47.6)	0.29
Body-mass index	26.7±4.4	26.7±4.3	1.00
Risk factors — no. (%)			
History of diabetes	19 (4.2)	12 (2.7)	0.21
History of hypertension	114 (25.1)	110 (24.4)	0.82
Current smoker	95 (20.9)	86 (19.1)	0.50
Medication use — no. (%)			
Aspirin	133 (29.3)	138 (30.7)	0.65
Beta-blocker	92 (20.3)	83 (18.4)	0.49
ACE inhibitor or ARB	87 (19.2)	72 (16.0)	0.21
Ezetimibe	50 (11.0)	47 (10.4)	0.78
**Baseline values for patients who completed the study**			
No. of patients	427	423	
Cholesterol — mg/dl			
Total	213.5±42.1	213.0±39.3	0.86
LDL	138.9±37.6	138.4±35.5	0.84
HDL	51.8±12.8	52.9±12.7	0.24
Ratio of LDL to HDL			0.29†
Median	2.7	2.5	
Interquartile range	2.1 to 3.4	2.1 to 3.3	
Triglycerides — mg/dl			0.22†
Median	97.4	97.4	
Interquartile range	75.2 to 141.6	70.8 to 132.8	
C-reactive protein — mg/liter			0.70†
Median	0.8	0.8	
Interquartile range	0.4 to 1.9	0.4 to 1.9	
Blood pressure — mm Hg			
Systolic	116.6±10.9	115.9±11.7	0.42
Diastolic	73.5±7.0	72.9±7.5	0.17

the course of the study. At the conclusion of the run-in period, patients were randomly assigned to receive either atorvastatin (at a dose established during the run-in period) with 60 mg of torcetrapib daily or atorvastatin monotherapy with corresponding placebo tablets. Patients and study personnel were unaware of study-group assignments, laboratory measurements, and carotid-imaging findings.

This article was written by the lead academic author, who vouches for the accuracy and completeness of the data and analyses. The study contract specified that a copy of the study database be provided to the coordinating center for independent analysis and granted the academic authors the unrestricted right to publish the results. The analyses have been confirmed by an independent center.

The New England Journal of Medicine
Downloaded from nejm.org on August 5, 2024. For personal use only.
No other uses without permission. Copyright © 2007 Massachusetts Medical Society. All rights reserved.

Table 1. (Continued.)

Variable	Atorvastatin Monotherapy	Atorvastatin plus Torcetrapib	P Value
**24-Mo follow-up**			
Cholesterol — mg/dl			
Total	218.8±45.7	216.9±51.1	0.58
LDL	143.2±42.2	115.1±48.5	<0.001
HDL	52.4±13.5	81.5±22.6	<0.001
Ratio of LDL to HDL			<0.001†
Median	2.7	1.3	
Interquartile range	2.1 to 3.4	1.0 to 1.8	
Triglycerides — mg/dl			0.001†
Median	97.4	88.5	
Interquartile range	70.8–141.6	70.8–119.0	
C-reactive protein — mg/liter			0.85†
Median	0.8	0.9	
Interquartile range	0.4 to 2.0	0.4 to 2.2	
Blood pressure — mm Hg‡			
Systolic	117.9±9.9	120.1±12.2	<0.001
Diastolic	74.2±6.3	74.7±7.0	<0.001
**Change from baseline**			
Cholesterol — %§			
Total	5.1±0.9	3.8±0.9	0.28
LDL	6.3±1.3	−14.4±1.3	<0.001
HDL	2.5±1.1	54.4±1.1	<0.001
Triglycerides — %			<0.001¶
Median	2.1	−7.7	
Interquartile range	−17.6 to 25.0	−25.0 to 20.0	
C-reactive protein — mg/liter			0.95¶
Median	0	0	
Interquartile range	−0.3 to 0.4	−0.3 to 0.4	
Blood pressure — mm Hg‡			
Systolic	1.3±6.9	4.1±8.0	<0.001
Diastolic	0.6±4.4	1.8±4.8	<0.001

* Plus–minus values are means ±SD. Body-mass index is the weight in kilograms divided by the square of the height in meters. To convert cholesterol values to millimoles per liter, multiply by 0.02586. To convert triglyceride values to millimoles per liter, multiply by 0.01129. ACE denotes angiotensin-converting enzyme, and ARB angiotensin-receptor blocker.
† The P value was calculated with the use of the Wilcoxon rank-sum test.
‡ Values are averages of all post-randomization measurements.
§ Values are the least-square mean percentage change (SE) from analysis of covariance with terms for treatment, region, baseline atorvastatin dose, and baseline lipid measure, with the last observation carried forward.
¶ The P value was calculated from analysis of covariance on rank-transformed data, with the last observation carried forward.

CAROTID ULTRASONOGRAPHY

All patients underwent carotid ultrasonography to assess carotid intima–media thickness.[19] Replicate scans were performed within a week of each other at baseline and at 24 months, with interim follow-up scans at 6, 12, and 18 months. At each visit, a circumferential scan was performed with image acquisition at four predefined angles of the near and far walls of the right and left common carotid artery, carotid bifurcation, and internal carotid

The New England Journal of Medicine
Downloaded from nejm.org on August 5, 2024. For personal use only.
No other uses without permission. Copyright © 2007 Massachusetts Medical Society. All rights reserved.

artery.[20] All imaging centers used the same imaging hardware, Sequoia 512 scanners equipped with 8L5 transducers (Siemens), and protocols for imaging acquisition. Five-second image sequences were saved in Digital Imaging in Communications in Medicine (DICOM) format (National Electrical Manufacturers Association) and were written to magnetic optical disks for transfer to reading centers. Two reading centers (at the University Medical Center in Utrecht, the Netherlands, and at Wake Forest University Medical Center in Winston-Salem, NC) used standardized equipment and protocols to process stored images.

Semiautomated readings were analyzed with the use of automated measurement software (Image and Data Analysis).[21] From each image sequence, the reader selected one frame in end diastole for measurement of carotid intima–media thickness. The leading edge (far wall) and trailing edge (near wall) of boundaries between the media and adventitia and between the lumen and intima were traced within the region of interest specified by the reader. Maximum carotid intima–media thickness was determined from a set of measurements perpendicular to the media–adventitia boundary. The readers were unaware of study-group assignments and of previous measurements of carotid intima–media thickness when reading an image.

Quality-assurance processes included central training and certification of all sonographers and readers on each continent, annual international meetings of sonographers and readers to reinforce protocol and standardized implementation, and regular site visits and performance reviews. Intraclass correlation coefficients for the mean maximum carotid intima–media thickness between replicate scans at baseline for 875 patients and at the end of the study for 814 patients were 0.90 and 0.88, respectively. The intraclass correlation coefficient for the monthly quality-assurance scans for 128 patients was 0.96. These estimates include differences within and between visits, within and between sonographers, and within and between reader-variability components.

The primary end point was annualized change in the maximum carotid intima–media thickness for the 12 carotid-artery segments (near and far walls of the right and left common carotid artery, the carotid bifurcation, and the internal carotid artery) based on all scans performed during the 2-year study period.

STATISTICAL ANALYSIS

Three hundred four patients were needed in each treatment group for the study to have a power of 90% to detect a difference of 0.020 mm in the annualized rate of change of carotid intima–media thickness with a two-sided alpha level of 0.05, assuming a common standard deviation of 0.076 mm per year.

A linear mixed-effects model was used to analyze the annualized rate of change in maximum carotid intima–media thickness, including 84 maximum measurements (12 segments multiplied by 7 visits) for each patient as the dependent variables, with random intercepts and slopes as a function of time and fixed effects for geographic region, atorvastatin dose at run-in, carotid segment, treatment, time, and interaction between time and treatment. Testing was two-sided and conducted with a 5% type I error rate.[22] Laboratory measurements were analyzed by analysis of covariance, including terms for baseline value, treatment, geographic region, and atorvastatin dose at run-in. Safety data were analyzed with the use of a linear model with terms for baseline value, hypertensive status, age, sex, race, smoking status, history of diabetes mellitus, body-mass index, creatinine clearance, and treatment.

With multiplicative interaction terms, we studied whether treatment effects differed across subgroups. These prespecified analyses were performed for age (<65 and ≥65 years), sex, race (white or nonwhite), HDL cholesterol levels (<40 mg and ≥40 mg per deciliter [1.0 mmol per liter]), LDL cholesterol levels (above and below the median), triglycerides (<150 mg and ≥150 mg per deciliter [1.7 mmol per liter]), smoking status, history of diabetes mellitus, history of hypertension, C-reactive protein levels (<3.0 mg and ≥3.0 mg per deciliter), and baseline maximum carotid intima–media thickness (above and below the median). Investigator-reported clinical adverse events were not tested for statistical differences because such events were not adjudicated and were expected to be low in frequency of occurrence. Therefore, the study did not have the power to detect differences.

RESULTS

PATIENTS

From December 19, 2003, to November 22, 2004, a total of 904 patients underwent randomization

The New England Journal of Medicine
Downloaded from nejm.org on August 5, 2024. For personal use only.
No other uses without permission. Copyright © 2007 Massachusetts Medical Society. All rights reserved.

at 37 centers in North America, Europe, and South Africa. Of these patients, 454 were assigned to the atorvastatin-only group and 450 to the torcetrapib–atorvastatin group. A total of 850 patients (427 in the atorvastatin-only group and 423 in the torcetrapib–atorvastatin group) remained in the study and underwent ultrasonography of the carotid artery at least once both at baseline and at follow-up (the full-analysis set). (Details of study-group assignments appear in the Supplementary Appendix, which is available with the full text of this article at www.nejm.org.) Demographic characteristics and baseline medications were similar in the two study groups (Table 1). The titrated daily dose of atorvastatin averaged 56.5 mg in both groups.

LABORATORY RESULTS AND BLOOD PRESSURE

Table 1 summarizes laboratory values and blood pressure at baseline and during the study period for the 850 patients in the full-analysis set who had post-baseline ultrasonographic results that could be evaluated. After 24 months, mean HDL cholesterol levels increased from 51.8 to 52.4 mg per deciliter (1.3 to 1.4 mmol per liter) in the atorvastatin-only group and from 52.9 to 81.5 mg per deciliter (1.4 to 2.1 mmol per liter) in the torcetrapib–atorvastatin group (Fig. 1). In the atorvastatin-only group, mean LDL cholesterol levels measured 165.5 mg per deciliter (4.3 mmol per liter) at screening and fell during the run-in period to 138.9 mg per deciliter (3.6 mmol per liter) at baseline. After 24

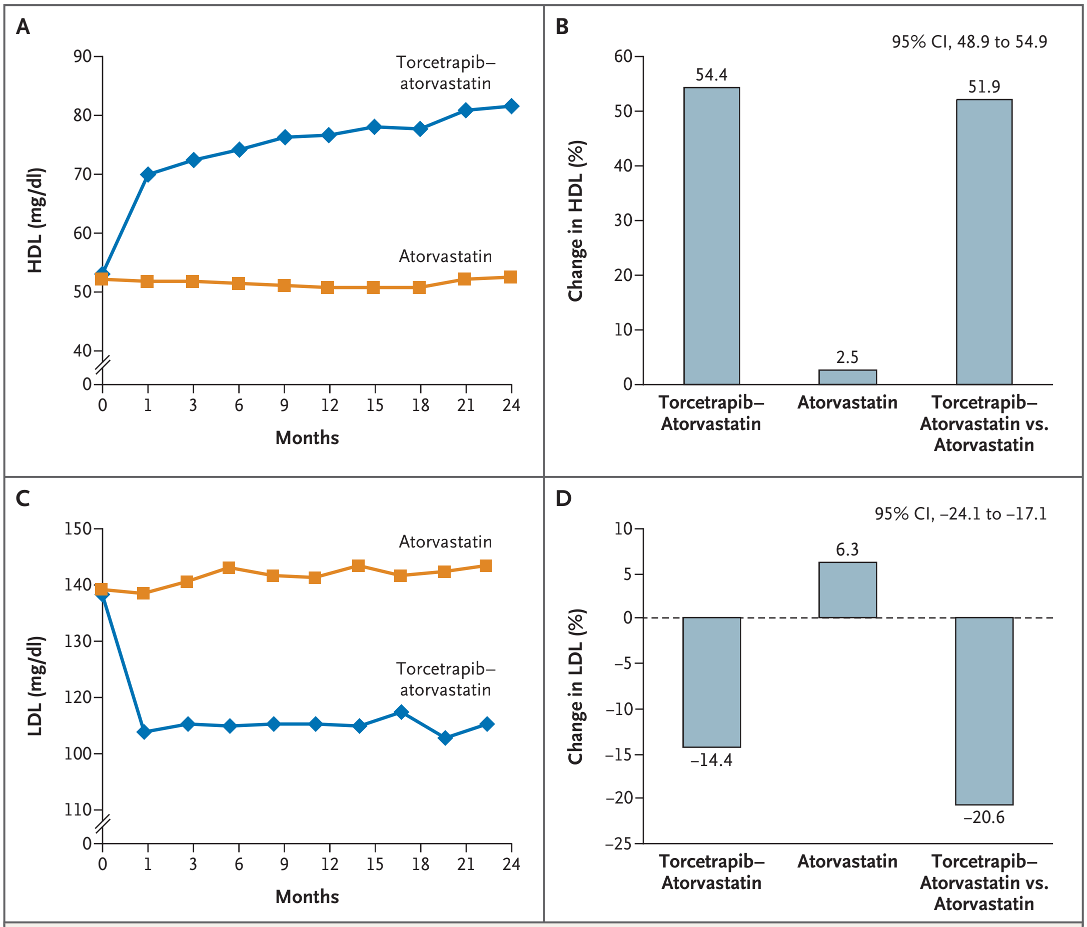

Figure 1. Changes in Levels of High-Density Lipoprotein (HDL) and Low-Density Lipoprotein (LDL) Cholesterol in Patients Receiving Atorvastatin Alone or Atorvastatin plus Torcetrapib.

Panels A and C show the levels of HDL and LDL cholesterol, respectively, in the study patients, and Panels B and D show the percent changes in HDL and LDL cholesterol, respectively, from baseline to 24 months, including a comparison of the percent change between the torcetrapib–atorvastatin group and the atorvastatin-only group for both HDL and LDL cholesterol (right-hand columns). To convert values for cholesterol to millimoles per liter, multiply by 0.02586.

The New England Journal of Medicine
Downloaded from nejm.org on August 5, 2024. For personal use only.
No other uses without permission. Copyright © 2007 Massachusetts Medical Society. All rights reserved.

months of treatment, mean LDL cholesterol levels in the atorvastatin-only group were 143.2 mg per deciliter (3.7 mmol per liter). In the torcetrapib-atorvastatin group, mean LDL cholesterol levels were 168.2 mg per deciliter (4.3 mmol per liter) at screening, 138.4 mg per deciliter (3.6 mmol per liter) at baseline, and 115.1 mg per deciliter (3.0 mmol per liter) at 24 months. As compared with atorvastatin alone, the net effect of torcetrapib was a 51.9% relative increase in HDL cholesterol and a 20.6% relative decrease in LDL cholesterol. Table 2 shows changes in lipoprotein subclasses in the two study groups. Baseline blood pressure was 116/73 mm Hg in the torcetrapib–atorvastatin group and 117/74 mm Hg in the atorvastatin-only group. During the study, mean systolic blood pressure increased by 1.3 mm Hg in the atorvastatin-only group and by 4.1 mm Hg in the torcetrapib–atorvastatin group, a least-square mean difference of 2.8 mm Hg (95% confidence interval [CI], 1.9 to 3.7; P<0.001).

CAROTID ULTRASONOGRAPHY

Table 3 summarizes the change in the primary and secondary efficacy measures as seen on carotid ultrasonography. The primary efficacy measure, annualized rate of change in maximum carotid intima–media thickness, was an increase of 0.0053 mm per year in the atorvastatin-only group and an increase of 0.0047 mm per year in the torcetrapib–atorvastatin group (P=0.87) (Fig. 2). However, the secondary efficacy measure, annualized change in the maximum and mean measures of carotid intima–media thickness for the common carotid artery, indicated regression in the atorvastatin-only group (a maximum decrease of 0.0042 mm per year and a mean decrease of 0.0014 mm per year) and progression in the torcetrapib–atorvastatin group (a maximum increase of 0.0040 mm per year [P=0.02] and a mean increase of 0.0038 mm per year [P=0.005]) (Table 3).

For nearly all prespecified subgroups, no heterogeneity in the difference between study groups was observed. Annualized change in maximum carotid intima–media thickness in patients with a history of diabetes was lower in the torcetrapib–atorvastatin group than in the atorvastatin-only group (P=0.05), although the number of patients with diabetes was limited (9 in the torcetrapib–atorvastatin group and 17 in the atorvastatin-only group). For patients with baseline HDL cholesterol levels of less than 40 mg per deciliter (1.0 mmol per liter), the results showed a trend in favor of atorvastatin monotherapy (P=0.09). However both of these results are probably due to chance.

CLINICAL ADVERSE EVENTS

Among patients who had at least 1 unadjudicated, investigator-reported serious adverse cardiovascular event (some had more than 1 event), 11 events were in the atorvastatin-only group (1 death from a cardiovascular cause, 1 stroke, and 9 ischemic or other cardiovascular events), and 24 were in the torcetrapib–atorvastatin group (3 nonfatal myocardial infarctions, 1 stroke, 18 ischemic or other cardiovascular events, 1 carotid stenosis, and 1 blood-pressure elevation considered to be a serious adverse event). Investigator-reported hypertensive adverse events were more common in the torcetrapib–atorvastatin group (8.9% vs. 3.7%), and blood-pressure values of more than 140/90 mm Hg were recorded more frequently in the torcetrapib–atorvastatin group (7.8% vs. 3.1%). A sustained increase of more than 15 mm Hg in systolic blood pressure occurred in 2.2% of patients in the torcetrapib–atorvastatin group, as compared with 0.9% of those treated with atorvastatin alone. (A complete list of adverse events appears in the Supplementary Appendix.)

Table 2. Changes in Lipoprotein Subclasses.*

Measure	Least-Square Mean	P Value
Apolipoprotein A		
Apolipoprotein A-I (%)	24.80	<0.001
HDL2 cholesterol (%)	157.12	<0.001
HDL3 cholesterol (%)	45.93	<0.001
HDL particle size (nm)	0.88	<0.001
Apolipoprotein B		
Apolipoprotein B-100 (%)	−16.71	<0.001
Non-HDL cholesterol (%)	−19.38	<0.001
Small LDL particles (nmol/liter)	−376.31	<0.001
LDL particle size (nm)	0.45	<0.001

* All comparisons are between the torcetrapib–atorvastatin group and the atorvastatin-only group.

DISCUSSION

The RADIANCE 1 trial showed that the addition of torcetrapib to atorvastatin did not provide incremental halting of the progression of athero-

The New England Journal of Medicine
Downloaded from nejm.org on August 5, 2024. For personal use only.
No other uses without permission. Copyright © 2007 Massachusetts Medical Society. All rights reserved.

Table 3. Baseline, Follow-up, and Change from Baseline in End Points for Maximum Carotid Intima–Media Thickness.*

Variable	Atorvastatin Monotherapy (N=427)		Atorvastatin plus Torcetrapib (N=423)		P Value
	Mean	Median (IQR)	Mean	Median (IQR)	
	millimeters				
**Baseline**					
Maximum carotid intima–media thickness for each of the 12 carotid-artery sites	1.15±0.31	1.09 (0.93–1.33)	1.13±0.28	1.09 (0.94–1.27)	0.38
Maximum carotid intima–media thickness for each of the 4 common carotid-artery sites	1.01±0.23	0.98 (0.83–1.17)	0.99±0.22	0.97 (0.82–1.14)	0.33
Mean carotid intima–media thickness for each of the 4 common carotid-artery sites	0.72±0.15	0.70 (0.60–0.82)	0.71±0.15	0.70 (0.59–0.81)	0.44
**24-Mo follow-up†**					
Maximum carotid intima–media thickness for each of the 12 carotid-artery sites	1.16±0.33	1.09 (0.94–1.32)	1.14±0.29	1.10 (0.95–1.27)	0.37
Maximum carotid intima–media thickness for each of the 4 common carotid-artery sites	1.00±0.22	0.97 (0.83–1.13)	1.00±0.21	0.99 (0.84–1.13)	0.87
Mean carotid intima–media thickness for each of the 4 common carotid-artery sites	0.71±0.14	0.70 (0.61–0.80)	0.72±0.14	0.71 (0.60–0.81)	0.71
**Annualized change from longitudinal model**	Slope	SE	Slope	SE	
	millimeters per year				
Maximum carotid intima–media thickness for each of the 12 carotid-artery sites	0.0053	0.0028	0.0047	0.0028	0.87
Maximum carotid intima–media thickness for each of the 4 common carotid-artery sites	−0.0042	0.0025	0.0040	0.0025	0.02
Mean carotid intima–media thickness for each of the 4 common carotid-artery sites	−0.0014	0.0013	0.0038	0.0013	0.005

* Plus–minus values are means ±SD. IQR denotes interquartile range, and SE standard error.
† Number was calculated by the last-observation-carried-forward method.

sclerosis in the carotid arteries of patients with familial hypercholesterolemia, as has been previously shown with the use of atorvastatin alone.[23] If anything, our data suggest a worsening of pathology conferred by this CETP inhibitor, despite a 52% increase in HDL cholesterol levels and a robust 21% decrease in LDL cholesterol levels in comparison with the results in the atorvastatin-only group. On the basis of extensive epidemiologic and various clinical-intervention studies, such lipoprotein changes were anticipated to render significant benefit.

To study atherosclerosis, we used ultrasonography to assess carotid intima–media thickness, a surrogate marker for cardiovascular disease.[24] The annualized change in maximum carotid intima–media thickness, the primary end point of the study, did not differ significantly between patients with familial hypercholesterolemia who were treated with atorvastatin alone and those treated with a combination of atorvastatin and torcetrapib. In fact, carotid intima–media thickness of the common carotid artery, a secondary end point of our study, provided evidence of accelerated atherogenesis in the patients who were receiving torcetrapib. It is highly unlikely that

The New England Journal of Medicine
Downloaded from nejm.org on August 5, 2024. For personal use only.
No other uses without permission. Copyright © 2007 Massachusetts Medical Society. All rights reserved.

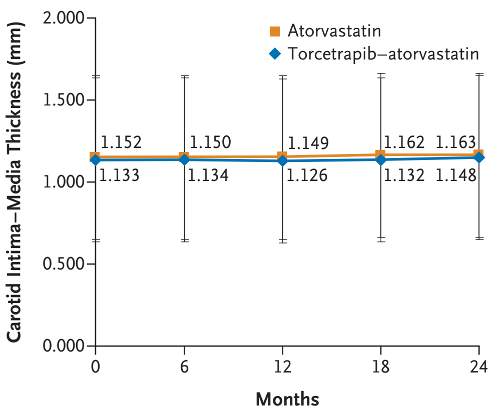

Figure 2. Average Maximum Carotid Intima–Media Thickness during 24 Months of Treatment.

The graph shows the average maximum carotid intima–media thickness in 12 carotid segments evaluated in the study patients treated with either atorvastatin monotherapy or a combination of torcetrapib and atorvastatin. I bars represent the standard deviation.

the unanticipated outcome of this trial can be attributed to the measurement of carotid intima–media thickness itself. This marker has previously been shown to constitute a strong and accurate predictor of the risk of future vascular events in population studies.[25] Furthermore, in evaluations of the efficacy of lipid-modifying medication,[26,27] antioxidants,[28] estrogens,[29] and antihypertensive drugs,[30] measurements of carotid intima–media thickness were successfully applied and were in line with the outcome of subsequent morbidity and mortality trials.

To account for the observed results, the potential benefit of the observed decrease in LDL cholesterol levels needs to be weighed against the detrimental effect of the rise in systolic blood pressure. The divergent effects of torcetrapib, as compared with atorvastatin alone, on LDL cholesterol (a decrease of 21%) and on systolic blood pressure (an increase of 2.8 mm Hg) are two prominent factors that may have affected carotid intima–media thickness. In a 2-year study of pravastatin, the Regression Growth Evaluation Statin Study (REGRESS),[26] a similar 28% decrease in LDL cholesterol levels was associated with a decrease of 0.05 mm in carotid intima–media thickness. In the Ezetimibe and Simvastatin in Hypercholesterolemia Enhances Atherosclerosis Regression (ENHANCE) trial, the addition of ezetimibe to high-dose simvastatin therapy was designed to reduce LDL cholesterol levels by 18 to 23% and was powered to detect a mean 2-year absolute difference of 0.05 mm in carotid intima–media thickness in 650 patients who were heterozygous for familial hypercholesterolemia.[31] The extrapolation of these findings to our study would suggest that the effect on LDL cholesterol levels would translate into a difference of 0.03 to 0.05 mm in carotid intima–media thickness in favor of the torcetrapib-atorvastatin group.

In contrast, the observed increase in systolic blood pressure can be expected to have an adverse effect on carotid intima–media thickness. In an attempt to account for this effect, we used data from a recent meta-analysis on the relationship between systolic blood pressure and carotid intima–media thickness.[30] That analysis suggests that the effect of the 2.8 mm Hg increase in systolic blood pressure would favor the atorvastatin-only group by 0.014 mm at 2 years. The net opposing effect of the LDL cholesterol level and systolic blood pressure should have left a residual benefit for patients in the torcetrapib–atorvastatin group. The fact that none was observed leaves no possibility of any beneficial effect of the large increase in HDL cholesterol.

In line with the concept that elevation in levels of HDL cholesterol protects against atherosclerosis, small and moderate increases in HDL cholesterol levels, as achieved by the use of nicotinic acid (21%)[27] and gemfibrozil (6%),[32] have previously been reported to yield a significant reduction in the rate of progression of carotid intima–media thickness and in the risk of major cardiovascular events. The absence of an effect of a much greater increase in HDL cholesterol (52%) in our study indicates that torcetrapib either has an adverse vascular effect that negated the changes in lipoprotein levels or that CETP inhibition is not an effective therapeutic strategy. Although our study cannot determine which hypothesis is accurate, there are several possibilities that merit consideration.

With respect to the discrepancy between the remarkable effects of torcetrapib on lipid metabolism and its effects on carotid intima–media thickness, a direct vasculotoxic effect — as shown by a rise in blood pressure — appears to be a possible explanation. The natural ability of HDL to induce vasorelaxation, an effect that is thought to be mediated through scavenger receptor B1,[33] may be adversely affected by torcetrapib.

Another possibility relates to the fact that in-

The New England Journal of Medicine
Downloaded from nejm.org on August 5, 2024. For personal use only.
No other uses without permission. Copyright © 2007 Massachusetts Medical Society. All rights reserved.

hibition of CETP by torcetrapib actually increases plasma levels of CETP. At a daily dose of 60 mg, torcetrapib continuously increases levels of CETP, a finding that is ascribed to an enhanced affinity of CETP for HDL.[34] This complex formation (CETP–torcetrapib–HDL) is in turn associated with extreme elevations of large HDL particles, as exemplified by the substantial increase in levels of HDL2 cholesterol (157%). In this context, it is worrisome that HDL cholesterol levels were found to increase steadily over the duration of the trial (Fig. 1). It can be hypothesized that these effects may interfere with one or more of the activities of HDL, which include serving as an acceptor of cellular cholesterol, inhibiting oxidation, thrombosis, and vascular inflammation, promoting endothelial repair, and protecting against apoptosis of endothelial cells.[35] The possibility that HDL may have lost its antiinflammatory potential is illustrated by the observation that torcetrapib did not affect levels of C-reactive protein. In contrast, monotherapy with a similar dose of atorvastatin in patients with familial hypercholesterolemia resulted in a 45% decrease in levels of high-sensitivity C-reactive protein in a trial similar to ours in duration and size.[36]

In conclusion, the use of torcetrapib in patients with familial hypercholesterolemia did not result in regression of atherosclerosis, as assessed by a combined measure of thickness of the carotid-artery wall, and even caused progression of disease in the common carotid segment. These effects occurred despite an unparalleled increase in the HDL cholesterol level (52%) and a substantial decrease in the LDL cholesterol level (21%).

Supported by Pfizer.

Dr. Kastelein reports receiving consulting fees and lecture fees from Pfizer, AstraZeneca, Merck, and Schering-Plough and grant support from Pfizer and AstraZeneca; Dr. Evans, receiving consulting fees from AstraZeneca and grant support from the National Heart, Lung, and Blood Institute and Pfizer; Dr. Barter, receiving consulting fees from Pfizer, AstraZeneca, Merck, and Abbott; lecture fees from Pfizer, AstraZeneca, Abbott, and Merck; and grant support from Pfizer; Drs. Revkin, Shear, and Duggan, being employees of Pfizer; Dr. Grobbee, receiving consulting fees and lecture fees from Pfizer, AstraZeneca, Servier, and Organon and grant support from Pfizer, AstraZeneca, Servier, and Organon; and Dr. Bots, receiving consulting and lecture fees and grant support from Pfizer, AstraZeneca, and Servier. No other potential conflict of interest relevant to this article was reported.

We thank Rudy Meijer in Schoorl, the Netherlands, for the design of the image-acquisition protocol, training, and quality control; and T. Roberts, D. Ambrose, A. Chin, W. Davidson, R. Burnside, A Caffrey, M. Li, and A. O'Reilly at Pfizer.

APPENDIX

The following investigators are listed according to ultrasonography center: **Europe:** *University Medical Center Utrecht, Utrecht, the Netherlands* — M. Bots (cochair), C. van Everdingen, M. Geurtsen, M. Djuanda, A. Kuin, F. Leus, R. Meijer, D. Mooiweer-Bogaerdt, K. Nijssen (cochair), H. Noordzij, L. Romkes, B. Sies, E. Stooker, F. Verhey, B. van der Vlist, L. van der Vlist, E. Wineke, H. Wisse. **United States:** *Wake Forest University School of Medicine, Winston-Salem, NC* — G. Evans and W. Riley (cochairs), B. Holley, J. Fleshman, M. Lauffer, L. Passmore, M. Wilder, P. Mille, T. Vitek.

In addition to the authors, the following investigators participated in this study: *Tygerberg Hospital, Parow, Cape Town, South Africa* — L. Burgess; *Cape Heart Centre University of Cape Town Health Science Faculty, Cape Town, South Africa* — A. Marais; *Johannesburg Hospital, Parktown, Johannesburg, South Africa* — F. Raal; *General University Hospital, Prague, Czech Republic* — R. Ceska; *Academisch Medisch Centrum, Amsterdam* — M. Trip; *Andro Medical Research, Rotterdam, the Netherlands* — E. Sijbrands; *Clinique des Maladies Lipidiques de Québec, Place de la Cité, Quebec, QC, Canada* — C. Gagne; *Vasculair Onderzoek Centrum Hoorn, the Netherlands* — D. Basart; *Academisch Ziekenhuis Leiden/Afdeling Inwendige Geneeskunde, Leiden, the Netherlands* — M. Huisman; *Universitair Medisch Centrum Utrecht, Utrecht, the Netherlands* — F. Visseren; *University of Utah, Salt Lake City* — P. Hopkins; *TweeSteden Ziekenhuis, Waalwijk, the Netherlands* — B. Imholz; *Universitair Medisch Centrum Sint Radboud, Nijmegen, the Netherlands* — A. Stalenhoef; *Oulu University Hospital, Oys, Finland* — M. Savolainen; *Hartford Hospital, Hartford, CT* — P. Thompson; *Centre de Médecine Génique Communautaire, Chicoutimi, QC, Canada* — D. Gaudet; *Clinical Research Institute of Montreal, Montreal* — J. Davignon; *University of Kuopio, Research Institute of Public Health, Kuopio, Finland* — J. Salonen; *Universita degli Studi di Brescia, Brescia, Italy* — E. Agabiti Rosei; *Institute for Clinical and Experimental Medicine, Prague, Czech Republic* — R. Cifkova; *University of Washington, Seattle* — R. Knopp; *Hôpital de la Pitié–Salpetrière, Paris* — E. Bruckert; *Medisch Centrum Alkmaar, Alkmaar, the Netherlands* — J. Sepers; *Oosterschelde Ziekenhuis, Goes, the Netherlands* — A. Liem; *Andromed Noord, Groningen, the Netherlands* — J. Jonker; *Albert Schweitzer Ziekenhuis, Sliedrecht, the Netherlands* — A. Cleophas; *Health Sciences Centre, Winnipeg, MB, Canada* — D. Mymin; *Massachusetts General Hospital, Boston* — L. Hemphill, R. Lees; *Baylor College of Medicine, Houston* — C. Ballantyne; *IRCCS Fondazione C. Mondino, Pavia, Italy* — A. Cavallini; *Reinier de Graaf Gasthuis/Cardio Research, Delft, the Netherlands* — A. Withagen; *Diakonessenhuis, Zeist Afdeling Inwendige Geneeskunde, Utrecht, the Netherlands* —M. Van De Ree; *St. Paul's Hospital, Vancouver, BC, Canada* — J. Frohlich; *Columbia–Presbyterian Medical Center, New York* — H. Ginsberg; *University of Minnesota, Minneapolis* — D. Duprez; and *Hopital Jeanne d'Arc Centre d'Investigation Clinique, Toul, France* — B. Guerci.

REFERENCES

1. Third Report of the National Cholesterol Education Program (NCEP) Expert Panel on Detection, Evaluation, and Treatment of High Blood Cholesterol in Adults (Adult Treatment Panel III) final report. Circulation 2002;106:3143-421.

2. Grundy SM, Cleeman JI, Merz CN, et al. Implications of recent clinical trials for the National Cholesterol Education Program Adult Treatment Panel III guidelines. Circulation 2004;110:227-39. [Erratum, Circulation 2004;110:763.]

The New England Journal of Medicine
Downloaded from nejm.org on August 5, 2024. For personal use only.
No other uses without permission. Copyright © 2007 Massachusetts Medical Society. All rights reserved.

3. LaRosa JC, Grundy SM, Waters DD, et al. Intensive lipid lowering with atorvastatin in patients with stable coronary disease. N Engl J Med 2005;352:1425-35.
4. Cannon CP, Braunwald E, McCabe CH, et al. Intensive versus moderate lipid lowering with statins after acute coronary syndromes. N Engl J Med 2004;350:1495-504. [Erratum, N Engl J Med 2006;354:778.]
5. LaRosa JC, He J, Vupputuri S. Effect of statins on risk of coronary disease: a meta-analysis of randomized controlled trials. JAMA 1999;282:2340-6.
6. Yusuf S, Reddy S, Ounpuu S, Anand S. Global burden of cardiovascular diseases: part I: general considerations, the epidemiologic transition, risk factors, and impact of urbanization. Circulation 2001;104:2746-53.
7. Gordon DJ, Probstfield JL, Garrison RJ, et al. High-density lipoprotein cholesterol and cardiovascular disease: four prospective American studies. Circulation 1989;79:8-15.
8. Barter P, Gotto A, LaRosa J, et al. On-treatment levels of HDL-C and the ratio of LDL-C/HDL-C as predictors of cardiovascular events in the Treating to New Targets (TNT) Study. Presented at the American College of Cardiology 55th Annual Scientific Session, Atlanta, March 11–14, 2006. J Am Coll Cardiol 2006;47:Suppl 4:298A. abstract.
9. de Grooth GJ, Kuivenhoven JA, Stalenhoef AF, et al. Efficacy and safety of a novel cholesteryl ester transfer protein inhibitor, JTT-705, in humans: a randomized phase II dose-response study. Circulation 2002;105:2159-65.
10. Brousseau ME, Schaefer EJ, Wolfe ML, et al. Effects of an inhibitor of cholesteryl ester transfer protein on HDL cholesterol. N Engl J Med 2004;350:1505-15.
11. Boekholdt SM, Kuivenhoven JA, Wareham NJ, et al. Plasma levels of cholesteryl ester transfer protein and the risk of future coronary artery disease in apparently healthy men and women: the prospective EPIC (European Prospective Investigation into Cancer and nutrition)-Norfolk population study. Circulation 2004;110:1418-23.
12. Okamoto H, Yonemori F, Wakitani K, Minowa T, Maeda K, Shinkai H. A cholesteryl ester transfer protein inhibitor attenuates atherosclerosis in rabbits. Nature 2000;406:203-7.
13. Kajinami K, Mabuchi H, Koizumi J, Takeda R. Serum apolipoprotein in heterozygous familial hypercholesterolemia. Clin Chim Acta 1992;211:93-9.
14. Hogue JC, Lamarche B, Gaudet D, et al. Association of heterozygous familial hypercholesterolemia with smaller HDL particle size. Atherosclerosis 2007;190:429-35.
15. Bagdade JD, Ritter MC, Subbaiah PV. Accelerated cholesteryl ester transfer in plasma of patients with hypercholesterolemia. J Clin Invest 1991;87:1259-65.
16. Junyent M, Cofan M, Nunez I, Gilabert R, Zambon D, Ros E. Influence of HDL cholesterol on preclinical carotid atherosclerosis in familial hypercholesterolemia. Arterioscler Thromb Vasc Biol 2006;26:1107-13.
17. de Grooth GJ, Smilde TJ, Van Wissen S, et al. The relationship between cholesteryl ester transfer protein levels and risk factor profile in patients with familial hypercholesterolemia. Atherosclerosis 2004;173:261-7.
18. Familial hypercholesterolaemia (FH): report of a WHO Consultation. Geneva: World Health Organization, 1998. (Accessed March 26, 2007, at http://whqlibdoc.who.int/hq/1998/WHO_HGN_FH_CONS_98.7.pdf.)
19. Kastelein JJP, van Leuven SI, Evans GW, et al. Designs of RADIANCE 1 and 2: carotid ultrasound studies comparing the effects of torcetrapib/atorvastatin with atorvastatin alone on atherosclerosis. Curr Med Res Opin 2007;23:885-94.
20. Bots ML, Evans GW, Riley WA, Grobbee DE. Carotid intima-media thickness measurements in intervention studies: design options, progression rates, and sample size considerations: a point of view. Stroke 2003;34:2985-94.
21. Liang Q, Wendelhag I, Wikstrand J, Gustavsson T. A multiscale dynamic programming procedure for boundary detection in ultrasonic artery images. IEEE Trans Med Imaging 2000;19:127-42.
22. Espeland MA, Craven TE, Miller ME, D'Agostino R Jr. 1996 Remington lecture: modeling multivariate longitudinal data that are incomplete. Ann Epidemiol 1999;9:196-205.
23. Smilde TJ, van Wissen S, Wollersheim H, Trip MD, Kastelein JJ, Stalenhoef AF. Effect of aggressive versus conventional lipid lowering on atherosclerosis progression in familial hypercholesterolaemia (ASAP): a prospective, randomised, double-blind trial. Lancet 2001;357:577-81.
24. de Groot E, van Leuven SI, de Graaf A, Bots ML, Kastelein JJP. Ultrasound measurements of carotid arterial walls and the progression and regression of atherosclerosis. Nat Clin Pract Cardiovasc Med (in press).
25. Bots ML, Baldassarre D, Simon A, et al. Carotid intima-media thickness and coronary atherosclerosis: weak or strong relations? Eur Heart J 2007;28:398-406.
26. de Groot E, Jukema JW, Montauban van Swijndregt AD, et al. B-mode ultrasound assessment of pravastatin treatment effect on carotid and femoral artery walls and its correlations with coronary arteriographic findings: a report of the Regression Growth Evaluation Statin Study (REGRESS). J Am Coll Cardiol 1998;31:1561-7.
27. Taylor AJ, Sullenberger LE, Lee HJ, Lee JK, Grace KA. Arterial Biology for the Investigation of the Treatment Effects of Reducing Cholesterol (ARBITER) 2: a double-blind, placebo-controlled study of extended-release niacin on atherosclerosis progression in secondary prevention patients treated with statins. Circulation 2004;110:3512-7. [Errata, Circulation 2004;110:3615, 2005;111:e446.]
28. Hodis HN, Mack WJ, LaBree L, et al. Alpha-tocopherol supplementation in healthy individuals reduces low-density lipoprotein oxidation but not atherosclerosis: the Vitamin E Atherosclerosis Prevention Study (VEAPS). Circulation 2002;106:1453-9.
29. Hodis HN, Mack WJ. Atherosclerosis imaging methods: assessing cardiovascular disease and evaluating the role of estrogen in the prevention of atherosclerosis. Am J Cardiol 2002;89:19E-27E.
30. Wang JG, Staessen JA, Li Y, et al. Carotid intima-media thickness and antihypertensive treatment: a meta-analysis of randomized controlled trials. Stroke 2006;37:1933-40.
31. Kastelein JJ, Sager PT, de Groot E, Veltri E. Comparison of ezetimibe plus simvastatin versus simvastatin monotherapy on atherosclerosis progression in familial hypercholesterolemia: design and rationale of the Ezetimibe and Simvastatin in Hypercholesterolemia Enhances Atherosclerosis Regression (ENHANCE) trial. Am Heart J 2005;149:234-9.
32. Rubins HB, Robins SJ, Collins D, et al. Gemfibrozil for the secondary prevention of coronary heart disease in men with low levels of high-density lipoprotein cholesterol. N Engl J Med 1999;341:410-8.
33. Yuhanna IS, Zhu Y, Cox BE, et al. High-density lipoprotein binding to scavenger receptor-BI activates endothelial nitric oxide synthase. Nat Med 2001;7:853-7.
34. Clark RW, Sutfin TA, Ruggeri RB, et al. Raising high-density lipoprotein in humans through inhibition of cholesteryl ester transfer protein: an initial multidose study of torcetrapib. Arterioscler Thromb Vasc Biol 2004;24:490-7.
35. Barter PJ, Nicholls S, Rye KA, Anantharamaiah GM, Navab M, Fogelman AM. Antiinflammatory properties of HDL. Circ Res 2004;95:764-72.
36. van Wissen S, Trip MD, Smilde TJ, de Graaf J, Stalenhoef AF, Kastelein JJ. Differential hs-CRP reduction in patients with familial hypercholesterolemia treated with aggressive or conventional statin therapy. Atherosclerosis 2002;165:361-6.

Copyright © 2007 Massachusetts Medical Society.

The New England Journal of Medicine
Downloaded from nejm.org on August 5, 2024. For personal use only.
No other uses without permission. Copyright © 2007 Massachusetts Medical Society. All rights reserved.
