# Supplementary material for: Lipid-lowering effect of combined therapy with high-intensity statins and CETP inhibitors: a Systematic Review and meta-analysis
Source: Front Endocrinol (Lausanne). 2025 May 1;16:1512670. doi: 10.3389/fendo.2025.1512670 (PMC12078159; doi:10.3389/fendo.2025.1512670)
Supplement: Supplementary file 1 [file DataSheet1.zip › Raw Data/Raw Data/Original Documentation Fruit/Derks2010(2)✔.pdf]

Coadministration of Dalcetrapib With Pravastatin, Rosuvastatin, or Simvastatin: No Clinically Relevant Drug–Drug Interactions

Michael Derks, MD, PhD, Markus Abt, PhD, Mary Phelan, BSc (Hons), Lynn Turnbull, BSc (Hons), Georgina Meneses-Lorente, PhD, Nuria Bech, MSc, Anne-Marie White, PhD, and Graeme Parr, BPharm (Hons)

Dalcetrapib targets cholesteryl ester transfer protein and increases high-density lipoprotein cholesterol (HDL-C) levels. It is in clinical development for the prevention of cardiovascular events and will likely be used in combination with standard of care, including statins. Three crossover studies in healthy males investigated the pharmacokinetic drug-drug interaction potential of 900 mg dalcetrapib and statins: two 3-period studies (dalcetrapib plus pravastatin or rosuvastatin) and a 2-period study (dalcetrapib plus simvastatin). Effect on lipids and safety were secondary end points. The 900 mg dose investigated is higher than the 600 mg dose currently being investigated in Phase III. Coadministration of dalcetrapib with pravastatin, rosuvastatin, or simvastatin was not associated with significant increases in statin exposure except for a 26% increase in rosuvastatin C_{max} (90% CI 1.088 to 1.468) but not AUC_{0-24} (90% CI 0.931 to 1.085). Dalcetrapib AUC_{0-24} and C_{max} were not significantly altered by coadministration with pravastatin, and were significantly lower when dalcetrapib was coadministered with rosuvastatin or simvastatin compared with dalcetrapib alone. The HDL-C increase with dalcetrapib was not compromised by coadministration with statins, and reduction in low-density lipoprotein cholesterol with dalcetrapib coadministered with statins was greater than with statins alone. Dalcetrapib alone and coadministered with statins was generally well tolerated.

Keywords: *dalcetrapib; CETP; statin; drug interactions*
Journal of Clinical Pharmacology, 2010;50:1188-1201
© 2010 The Author(s)

The use of 3-hydroxy-3-methylglutaryl coenzyme A (HMG-CoA) reductase inhibitors (statins), leads to reduced levels of low-density lipoprotein cholesterol (LDL-C) and results in improvements in cardiovascular disease (CVD) morbidity and mortality.[1] Despite the efficacy of current standard of care, there remains a substantial risk of CVD, as current therapies reduce risk by 30% to 40%.[2]

One potential approach to address the risk of CVD that is not adequately addressed by the standard of care is raising levels of high-density lipoprotein cholesterol (HDL-C). Increased HDL-C has been associated with a decreased risk of CVD in epidemiologic studies[3-5] and an improvement in CVD outcomes in clinical trials with lipid-modifying drugs.[6-8] Increase in HDL-C can be achieved through inhibition of cholesteryl ester transfer protein (CETP) activity, and decreased plasma levels of CETP have been associated with increased HDL-C[9] and a decreased risk of coronary artery disease.[10] Dalcetrapib, an agent that targets CETP, is currently in clinical development and has been reported to increase levels of HDL-C by >30%.[11]

Because dalcetrapib will in most cases be prescribed in combination with statins, a series of studies was performed to investigate any potential drug-drug interactions when dalcetrapib is coadministered with pravastatin, rosuvastatin, or simvastatin. Area under the concentration-time curve (AUC_{0-24}) and maximum plasma concentration observed (C_{max}) for dalcetrapib and for the statins and their metabolites were the primary pharmacokinetic end points. Secondary end points included effects on plasma lipids and safety. Dalcetrapib was given at a dose of 900 mg in all of the studies. Although the 900 mg dalcetrapib dose was used in earlier studies,[11,12] 600 mg is the dose chosen for the Phase III dal-OUTCOMES study (ClinicalTrials.gov identifier NCT00658515; accessed May 1, 2009).

From F. Hoffmann-La Roche Ltd, Basel, Switzerland. Submitted for publication July 20, 2009; version accepted November 11, 2009. Address for correspondence: Michael Derks, MD, PhD, Bldg. 663, office 2139, F. Hoffmann-La Roche Ltd, 4070 Basel, Switzerland; Phone: +41 61 68 79584; Fax: +41 61 68 86007; e-mail: michael.derks@roche.com.
DOI:10.1177/0091270009358709

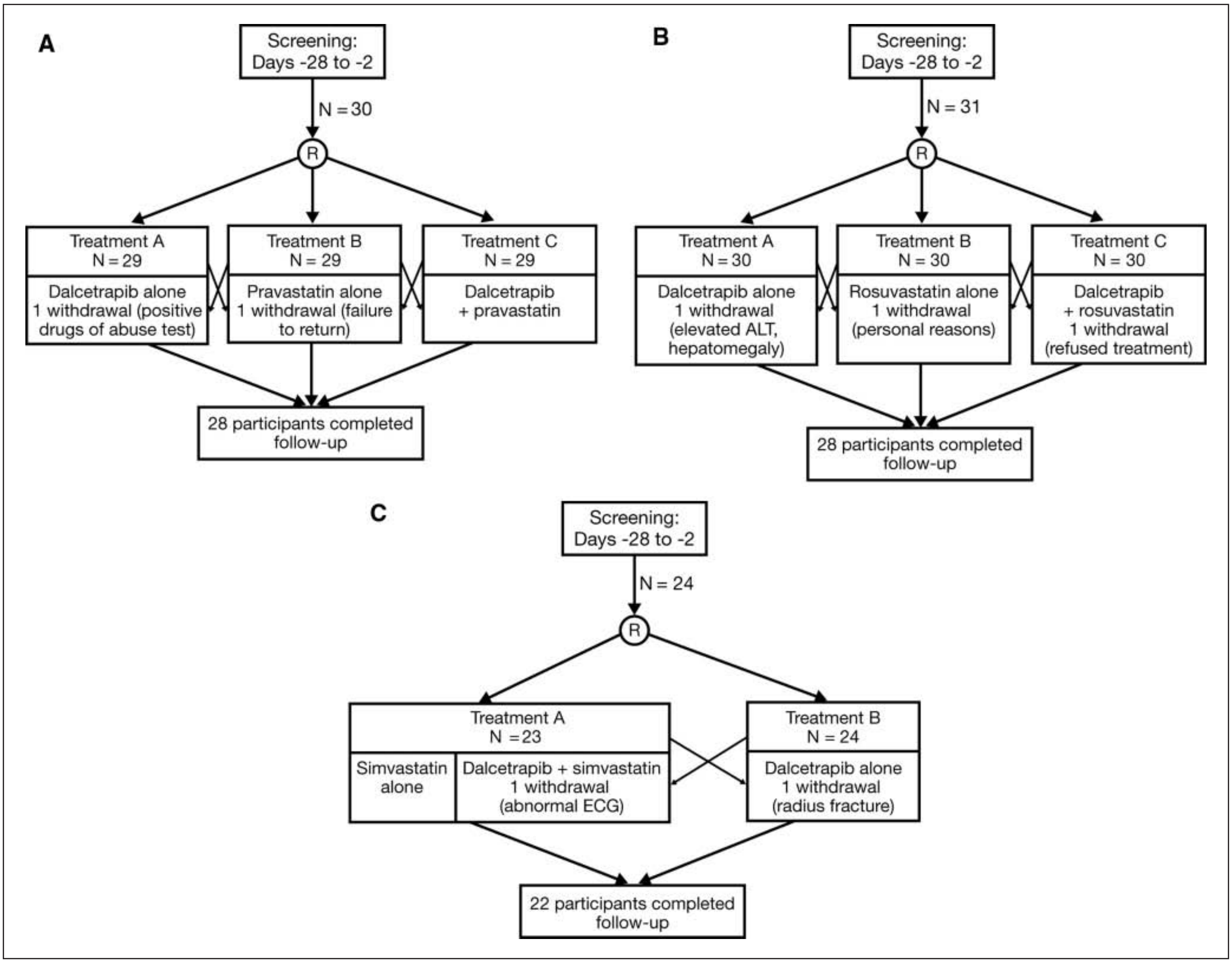

Figure 1. Study design and participant flow diagrams. (A) Pravastatin study: Each treatment was administered for 8 days. Washout between each treatment was 10 to 14 days and follow-up was 10 to 14 days after the final dose. Dalcetrapib dose: 900 mg QD; pravastatin dose=40 mg QD. (B) Rosuvastatin study: Each treatment was administered for 8 days. Washout between each treatment was 10 to 14 days and follow-up was 10 to14 days after the final dose. Dalcetrapib dose=900 mg QD; rosuvastatin dose=40 mg QD. (C) Simvastatin study: Simvastatin was administered on days 1 to 5 and dalcetrapib + simvastatin was administered on days 6 to 13 of treatment A. Treatment B was dalcetrapib alone administered for 8 days. Washout between each treatment was 11 to 17 days and follow-up was 7 to 14 days after the final dose. Dalcetrapib dose=900 mg QD; simvastatin dose=40 mg QD. R, randomization; N, number of participants who proceeded to the next step; QD, once daily.

METHODS

Study Population

Three drug-drug interaction studies investigating coadministration of dalcetrapib plus pravastatin, rosuvastatin, or simvastatin were performed in healthy male participants, aged 18 to 65 years inclusive (Figure 1). Body mass index was 18 to 32 kg/m^2 inclusive for the simvastatin study and 18 to 30 kg/m^2 inclusive for the pravastatin and rosuvastatin studies. Exclusion criteria included clinically significant symptoms of infectious disease; known history of porphyria, myopathy, or an active liver disease; use of concomitant medication except acetaminophen; clinically relevant history of drug or alcohol misuse or abuse; and positive drugs of abuse test at screening. History of clinically significant gastrointestinal, cardiovascular, musculoskeletal, endocrine, hematological, psychiatric, renal, hepatic,

bronchopulmonary, or neurological conditions; allergic disease; or lipid metabolism disorders also excluded enrollment. The pravastatin study excluded participants who smoked more than 5 cigarettes per day; the rosuvastatin and simvastatin studies were limited to nonsmokers.

The studies were conducted in compliance with the principles of the Declaration of Helsinki and were performed according to Good Clinical Practice guidelines. All participants provided written informed consent. For each study, the protocol was reviewed by an independent ethics committee: Guy's Research Ethics Committee, London, UK, for the pravastatin study; the Comité Consultatif de Protection des Personnes dans la Recherche Biomédicale d'Alsace, Strasbourg, France, for the rosuvastatin study; and the Welwyn Clinical Pharmacology Ethics Committee, Hatfield, Hertfordshire, UK, for the simvastatin study.

Study Medication

Dalcetrapib was provided in the form of 300-mg tablets from Clinical Trial Supplies, F. Hoffmann-La Roche (Basel, Switzerland), in accordance with Roche standards and local regulations. Pravastatin (40-mg tablets) was purchased by Roche and packaged and labeled per participant by Guy's Drug Research Unit (London, UK). Rosuvastatin (40-mg tablets) was purchased and packaged per participant by Clinical Trial Supplies, F. Hoffmann-La Roche (Basel, Switzerland). Simvastatin (40-mg tablets) was purchased locally by the Roche Clinical Pharmacology Unit (Welwyn, UK). To ensure compliance, in all 3 studies, medication was administered at the study center by an investigator or other designated personnel. Dalcetrapib, pravastatin, rosuvastatin, and simvastatin were administered orally once a day with approximately 200 mL of still water either 15 minutes after a standardized light breakfast (pravastatin and rosuvastatin studies) or 30 minutes after a standardized breakfast (simvastatin study).

Study Design

The studies were randomized, open-label studies with washout periods between treatments of approximately 14 days. The studies of 900 mg dalcetrapib with 40 mg pravastatin and 40 mg rosuvastatin were 3-period, 3-treatment crossover studies (Treatment A: 8 days dalcetrapib alone; Treatment B: 8 days pravastatin or rosuvastatin alone; Treatment C: 8 days dalcetrapib plus pravastatin or rosuvastatin), while the study of 900 mg dalcetrapib with 40 mg simvastatin was a 2-period, 2-treatment crossover study (Treatment A: Days 1 to 5 simvastatin alone, Days 6 to 13 dalcetrapib plus simvastatin; Treatment B: 8 days dalcetrapib alone) (Figure 1). For the pravastatin and rosuvastatin studies, participants were randomized to one of 6 sequences according to a 3-period Williams Latin square design. For the simvastatin study, participants were randomized to one of 2 treatment sequences.

Screening was performed between 28 and 2 days prior to dosing and involved a full medical history and complete physical examination including electrocardiogram (ECG), vital signs, clinical laboratory tests, height, and weight. On the morning of Day -1, blood and urine samples were collected for laboratory safety tests and a medical re-evaluation was performed. On Day -1, tests were performed for drugs of abuse; these included an alcohol breath analyzer test in all 3 studies and urinary cotinine test in the rosuvastatin and simvastatin studies. Clinical assessments during the study included measurement of vital signs, electrocardiogram (ECG), and blood sampling for CETP mass, CETP activity, plasma lipid profile, and pharmacokinetic measurements.

In the pravastatin and rosuvastatin studies, full pharmacokinetic profiles were determined for dalcetrapib and its main metabolites and for the statin and its main metabolites on Day 8 of the appropriate treatment periods. In the simvastatin study, full pharmacokinetic profiles were obtained for dalcetrapib and its metabolites on Day 13 of treatment A and on Day 8 of treatment B, and for simvastatin and its metabolites on Days 5 and 13 of treatment A.

Pharmacokinetic Assessments

The primary pharmacokinetic parameters—AUC_{0-24} and C_{max} for dalcetrapib and for pravastatin, rosuvastatin, and simvastatin and their metabolites—were assessed from venous blood samples. After treatment of plasma samples with dithiothreitol for thiolysis and the formation of the N-ethylmaleimide derivative, the concentration of dalcetrapib active form was determined using a validated liquid chromatography coupled with a tandem mass spectrometry (LC-MS/MS) method, by Roche for the pravastatin and simvastatin studies and by Swiss BioAnalytics AG, 4127 Birsfelden, Switzerland for the rosuvastatin study. Plasma concentrations of the S-methyl (S-Me) and S-glucuronide (S-Glu) metabolites of dalcetrapib were determined by Roche for the pravastatin and simvastatin studies and

by Swiss BioAnalytics for the rosuvastatin study. Plasma concentrations were determined for pravastatin and 3-alpha-hydroxy-iso-pravastatin using a validated LC-MS/MS method by AAI Deutschland GmbH & Co KG, Neu-Ulm, Germany, and for rosuvastatin, rosuvastatin-lactone, N-desmethyl rosuvastatin lactone, simvastatin, and simvastatin acid using a validated LC-MS/MS method by Advion BioServices, Ithaca, NY, USA.

The lower limit of quantification was 5 ng/mL for dalcetrapib and dalcetrapib-S-Me and was 50 ng/mL for the dalcetrapib-S-Glu. In the pravastatin and simvastatin studies, the calibration range was 5 to 4000 ng/mL for dalcetrapib and dalcetrapib-S-Me and 50 to 10 000 ng/mL for dalcetrapib-S-Glu. In the rosuvastatin study, the calibration range was 5 to 4000 ng/mL for dalcetrapib, 5 to 2000 ng/mL for S-Me, and 50 to 4000 ng/mL for S-Glu.

In the pravastatin study, the interassay precision obtained from quality control samples ranged from 10.5% to 11.2%, 6.2% to 9.2%, and 9.1% to 12.4% for dalcetrapib, dalcetrapib-S-Me, and dalcetrapib-S-Glu, respectively. The interassay accuracy ranged from 96.8% to 105.2%, 101.3% to 104.0%, and 91.1% to 95.9% for dalcetrapib, dalcetrapib-S-Me, and dalcetrapib-S-Glu, respectively. No influence of interference was observed with pravastatin on the assay. The lower limit of quantitation was 0.5 ng/mL for pravastatin and its metabolite 3-alpha-hydroxyiso-pravastatin, with a calibration range up to 250 ng/mL. The interassay precision obtained from quality control samples ranged from 8.9% to 9.4% for pravastatin and from 8.5% to 9.3% for 3-alpha-hydroxy-iso-pravastatin. The interassay accuracy ranged from 96.2% to 102.3% for pravastatin and 95.8% to 108.8% for 3-alpha-hydroxy-iso-pravastatin.

In the rosuvastatin study, the precision and accuracy of the assay, as determined from the analysis of quality control samples prepared in human plasma, ranged from 4.3% to 7.5% and from 94.3% to 104%, for dalcetrapib, dalcetrapib-S-Me, and dalcetrapib-S -Glu. For rosuvastatin, the lower limit of quantitation was 0.5 ng/mL, with a calibration range up to 100 ng/mL. For rosuvastatin lactone and *N*-desmethyl rosuvastatin, the lower limit of quantitation was 0.5 ng/mL with a calibration range up to 50 ng/mL. The interassay precision obtained from quality control samples ranged from 3.4% to 5.1% for rosuvastatin, 4.2% to 5.9% for rosuvastatin lactone, and 3.0% to 5.2% for *N*-desmethyl rosuvastatin. The mean accuracy of the assay, as determined from the analysis of quality control samples, ranged from 99.5% to 101.9% for rosuvastatin, 93.9% to 99.7% for rosuvastatin lactone, and 100.7% to 102.6% for *N*-desmethyl rosuvastatin.

In the simvastatin study, the precision and accuracy of the assay, as determined from the analysis of quality control samples prepared in human plasma, ranged from 3.7% to 11.9% and from 101.8% to 111.7%, for dalcetrapib, dalcetrapib-S-Me, and dalcetrapib-S-Glu. The precision of the assay for simvastatin, as determined from the analysis of quality control samples, was ≤10.8% for simvastatin and ≤10.3% for simvastatin acid. The accuracy of the assay, as determined from the analysis of quality control samples, ranged from 94.6% to 97.8% for simvastatin and from 98.7% to 104.3% for simvastatin acid. The lower limit of quantification was 0.5 ng/mL for simvastatin and simvastatin acid.

Pharmacodynamic Assessments

Pharmacodynamic parameters included fasting lipid profiles (triglycerides [TG], LDL-C, HDL-C, very-low-density lipoprotein cholesterol [VLDL-C], apolipoprotein [Apo] A-I, ApoA-II, ApoB, and total cholesterol); CETP mass and activity; HMG-CoA reductase activity; and the composition of lipoprotein subfractions obtained by fast lipoprotein chromatography (FLPC) and ^{1}H-NMR. Lipid profile analysis and FLPC was performed by Eurofins Medinet B.V., Breda, The Netherlands. Analysis of lipoprotein subclasses by ^{1}H-NMR in the simvastatin study was performed by LipoScience Inc., Raleigh, NC, USA. Cholesteryl ester transfer protein activity in all 3 studies and CETP mass in the rosuvastatin and simvastatin studies were measured by Pacific Biometrics Inc., Seattle, WA, USA. HMG-CoA reductase activity was assayed in the pravastatin and rosuvastatin studies and was analyzed by PPD Global Central Labs, Highland Heights, KY, USA.

Safety Assessments

Adverse events (AEs) were monitored throughout the study, and the intensity (mild, moderate, or severe) and relationship to study treatment (probable, possible, remote, or unrelated) was assessed and recorded by the investigator. Clinical laboratory tests, including hematology, biochemistry including electrolytes, liver enzymes, coagulation, urinalysis, serology; alcohol breath test; and drugs of abuse tests were performed. Vital signs and 12-lead ECG measurements were recorded.

Statistical Analyses

Sample size. Minimum sample sizes for the 3 studies were determined on the basis of their primary objective, which was to evaluate the effect of combined

Table I Baseline Characteristics

	Pravastatin Study	Rosuvastatin Study	Simvastatin Study
N	30	31[a]	24
Sex, male, n (%)	30 (100)	31 (100)	24 (100)
Race			
White, n (%)	26 (87)	28 (90)	24 (100)
Black, n (%)	3 (10)	3 (10)	0
Hispanic, n (%)	1 (3)	0	0
Age, y, mean ± SEM	29.8 ± 1.59	30.0 ± 1.93	41.5 ± 2.76
Weight, kg, mean ± SEM	75.23 ± 1.74	75.65 ± 1.69	83.35 ± 1.72
Body mass index, kg/m^2, mean ± SEM	24.37 ± 0.46	23.84 ± 0.46	26.73 ± 0.52
Tobacco use, n (%)[b]	5 (17)	0	0

Abbreviation: SEM, standard error of mean.
a. In the rosuvastatin study, an early dropout was replaced, giving a sample size of 31 subjects overall.
b. In the pravastatin study, men who smoked more than 5 cigarettes per day (or equivalent in cigars or pipe tobacco) were excluded. In the rosuvastatin and simvastatin studies, all smokers were excluded.

multiple dosing of dalcetrapib and the statin on the plasma pharmacokinetics of dalcetrapib and the statin. For each study, 24 evaluable patients were required, with replacement allowed if subjects dropped out. In the pravastatin and rosuvastatin 3-period studies, a sample size of 30 was planned to provide the minimum evaluable participants, ensuring with at least 80% probability in the pravastatin study and 90% probability in the rosuvastatin study that the 90% confidence limits for the relative effects of combination treatment on pravastatin, rosuvastatin, or dalcetrapib do not extend by a factor of >1.5 above or by a factor of <0.67 below the true relative difference. In the simvastatin 2-period study, a sample size of 24 was chosen (with replacement allowed if >2 subjects dropped out) to ensure with at least 80% probability that the 90% confidence limits for the relative effects of combination treatment on simvastatin and dalcetrapib did not extend by a factor of >1.5 above or by a factor of <0.67 below the true relative difference. A minimum sample size of 24 subjects was sufficiently powered to detect the occurrence of adverse events with an incidence of ≥10% in the population, therefore these sample sizes were considered sufficient to detect major tolerability and safety issues.

Pharmacokinetic data analysis: Respective contrasts from an analysis of variance performed on logarithmically transformed variables were used to assess the effect of multiple-dose statin on multiple-dose dalcetrapib and of multiple-dose dalcetrapib on multiple-dose statin. Geometric means ratio and its 90% confidence interval were estimated for $AUC_{0\text{-}24}$ and C_{max} for combination treatments relative to the statin or dalcetrapib alone.

RESULTS

Baseline Demographic Characteristics

The pravastatin study randomized 30 participants, the rosuvastatin study randomized 31 participants (an early dropout was replaced), and the simvastatin study randomized 24 participants. Baseline demographic characteristics for the participants in each of the studies are shown in Table I. The pravastatin study was conducted from September 27, 2006 to December 18, 2006; the rosuvastatin study was conducted from October 24, 2006 to January 15, 2007; and the simvastatin study was conducted from April 25, 2005 to September 2, 2005.

Study Compliance

Actual exposure to study drug was in excess of 90% of the per protocol exposures. Full courses of statin and dalcetrapib treatments were completed by 28 of 30 subjects in the pravastatin study, 28 of 31 subjects in the rosuvastatin study, and 22 of 24 subjects in the simvastatin study. In the pravastatin study, 1 subject received dalcetrapib for 7 days, and 1 completed the dalcetrapib plus pravastatin treatment phase but withdrew after 2 days of pravastatin alone. In the rosuvastatin study, 1 subject completed only the rosuvastatin alone and dalcetrapib alone phases and withdrew before the dalcetrapib plus rosuvastatin phase. Another completed the dalcetrapib alone and dalcetrapib plus rosuvastatin phases and withdrew on Day 1 of the rosuvastatin alone phase. A third withdrew after 7 days in the rosuvastatin phase. In the

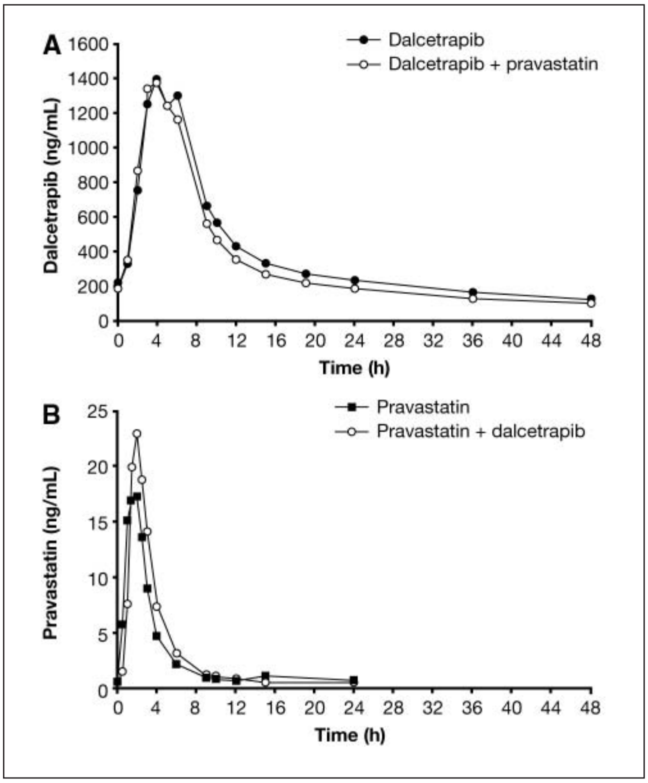

Figure 2. (A) Mean dalcetrapib plasma concentrations following administration alone or in combination with pravastatin. (B) Mean pravastatin plasma concentrations following administration alone or in combination with dalcetrapib.

simvastatin study, 1 subject was withdrawn after completing the simvastatin alone phase (because of bone fracture), while another completed the dalcetrapib alone phase but missed the final day of dosing in the dalcetrapib and simvastatin coadministration phase.

Pharmacokinetic Results

Effect of coadministration with statins on the pharmacokinetic profile of dalcetrapib: Coadministration of 900 mg dalcetrapib with pravastatin resulted in no significant changes in dalcetrapib exposure compared with dalcetrapib alone (Figure 2A; Table II). Mean ± standard deviation values for dalcetrapib AUC_{0-24} and C_{max} for dalcetrapib coadministered with pravastatin compared with dalcetrapib given alone were 12 600 ± 5000 h*ng/mL vs 13 800 ± 6640 h*ng/mL for AUC_{0-24} and 1700 ± 755 ng/mL vs 1770 ± 920 ng/mL for C_{max}.

There were statistically significant decreases in dalcetrapib exposure with coadministration of 900 mg dalcetrapib with rosuvastatin compared with administration of dalcetrapib alone (Figure 3A; Table III). Mean ± standard deviation values for AUC_{0-24} and C_{max} for dalcetrapib when coadministered with rosuvastatin compared with administration of dalcetrapib alone were 6270 ± 2040 h*ng/mL vs 9380 ± 2520 h*ng/mL for AUC_{0-24} and 937 ± 351 ng/mL vs 1170 ± 349 ng/mL for C_{max}.

Table II Pharmacokinetics of Coadministered Pravastatin and Dalcetrapib

Analyte/Parameter	Geometric Mean Ratio	90% CI	*P* Value[a]
Dalcetrapib			
AUC_{0-24} (h*ng/mL)	0.921	0.825, 1.027	.2086
C_{max} (ng/mL)	0.967	0.845, 1.106	.6707
Pravastatin			
AUC_{0-24} (h*ng/mL)	0.753	0.650, 0.872	.0037
C_{max} (ng/mL)	0.772	0.634, 0.940	.0335

Abbreviations: AUC_{0-24}, area under the concentration-time curve; CI, confidence interval; C_{max}, maximum plasma concentration.
a. For dalcetrapib, pravastatin coadministered with dalcetrapib vs dalcetrapib alone; for pravastatin, dalcetrapib coadministered with pravastatin vs pravastatin alone.

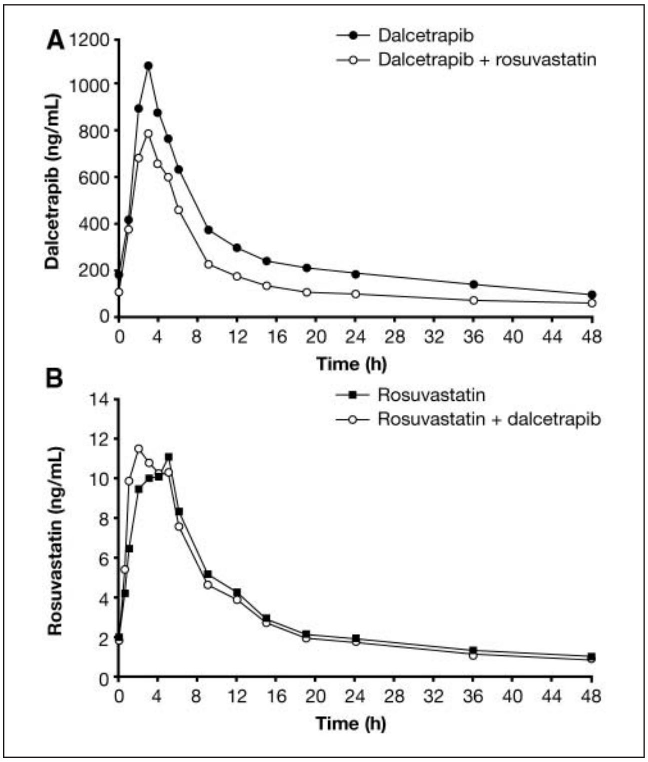

Figure 3. (A) Median dalcetrapib plasma concentrations following administration alone or in combination with rosuvastatin. (B) Median rosuvastatin plasma concentrations following administration alone or in combination with dalcetrapib.

Table III Pharmacokinetics of Coadministered Rosuvastatin and Dalcetrapib and Their Metabolites

Analyte/Parameter	Geometric Mean Ratio	90% CI	*P* Value[a]
Dalcetrapib			
AUC_{0-24} (h*ng/mL)	0.649	0.606, 0.695	<.0001
C_{max} (ng/mL)	0.768	0.704, 0.838	<.0001
Dalcetrapib-S-Glu			
AUC_{0-24} (h*ng/mL)	1.149	0.998, 1.322	.1056
C_{max} (ng/mL)	1.104	0.977, 1.248	.1783
Dalcetrapib-S-Me			
AUC_{0-24} (h*ng/mL)	0.873	0.822, 0.927	.0007
C_{max} (ng/mL)	0.908	0.822, 1.003	.1105
Rosuvastatin			
AUC_{0-24} (h*ng/mL)	1.005	0.931, 1.085	.9085
C_{max} (ng/mL)	1.264	1.088, 1.468	.0132
Rosuvastatin lactone			
AUC_{0-24} (h*ng/mL)	0.689	0.570, 0.832	.0072
C_{max} (ng/mL)	0.626	0.512, 0.765	.0031
N-desmethyl rosuvastatin			
AUC_{0-24} (h*ng/mL)	NC	NC	NC
C_{max} (ng/mL)	0.770	0.688, 0.861	0.0006

Abbreviations: AUC_{0-24}, area under the concentration-time curve; CI, confidence interval; C_{max}, maximum plasma concentration; dalcetrapib-S-Glu, dalcetrapib-S-glucuronide; dalcetrapib-S-Me, dalcetrapib-S-methyl; NC, not calculated.

a. For dalcetrapib and metabolites, rosuvastatin coadministered with dalcetrapib vs dalcetrapib alone; for rosuvastatin and metabolites, dalcetrapib coadministered with rosuvastatin vs rosuvastatin alone.

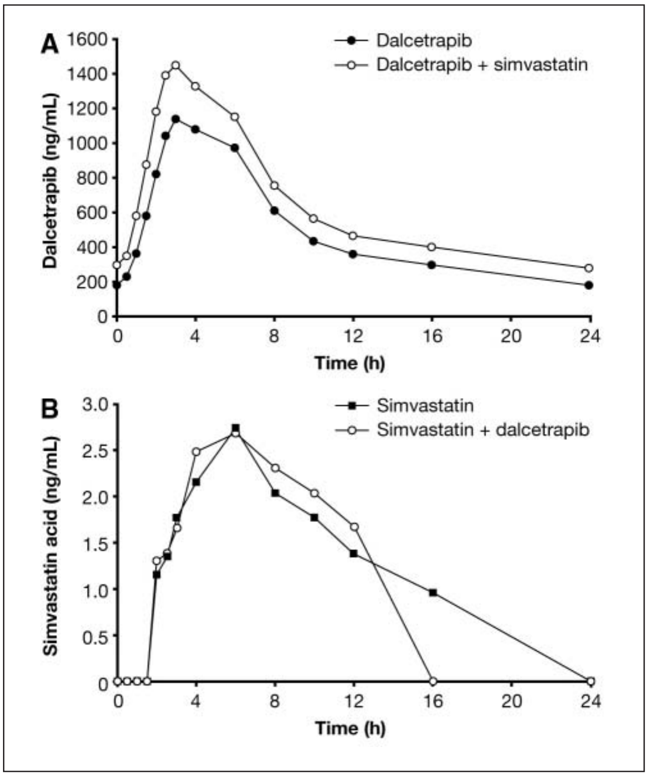

Figure 4. (A) Mean dalcetrapib plasma concentrations following administration alone or in combination with simvastatin. (B) Mean simvastatin acid plasma concentrations following administration alone or in combination with dalcetrapib.

When 900 mg dalcetrapib and simvastatin were coadministered, there was a statistically significant reduction in AUC_{0-24} and C_{max} for dalcetrapib (Figure 4A; Table IV). Compared with administration of dalcetrapib alone, the mean ± standard deviation values for coadministration of dalcetrapib with simvastatin were 11 500 ± 3400 h*ng/mL vs 15 000 ± 4780 h*ng/mL for AUC_{0-24} and 1370 ± 428 ng/mL vs 1610 ± 477 ng/mL for C_{max}.

Exposure to the dalcetrapib metabolites dalcetrapib-S-Me and dalcetrapib-S-Glu was comparable with dalcetrapib coadministered with pravastatin and with dalcetrapib administered alone (data not shown). When dalcetrapib was coadministered with rosuvastatin, there was a small but significant decrease in the AUC_{0-24} of dalcetrapib-S-Me but no significant difference in the C_{max} of dalcetrapib-S-Me or in exposure to dalcetrapib-S-Glu compared with dalcetrapib alone (Table III). Coadministration with simvastatin did not significantly alter the exposure to these dalcetrapib metabolites compared with dalcetrapib alone (Table IV).

Effect of coadministration with dalcetrapib on the pharmacokinetic profile of statins: Exposure to pravastatin was significantly reduced when pravastatin was coadministered with 900 mg dalcetrapib (Figure 2B; Table II). The mean ± standard deviation values for AUC_{0-24} and C_{max} for pravastatin coadministered with dalcetrapib compared with pravastatin administered alone were 61.4 ± 62.2 h*ng/mL vs 67.9 ± 55.9 h*ng/mL for AUC_{0-24} and 25.8 ± 27.9 ng/mL vs 27.5 ± 23.9 ng/mL for C_{max}. Coadministration of pravastatin with dalcetrapib was also associated with reductions in mean ± standard deviation AUC_{0-24} and C_{max} for the pravastatin metabolite 3-alpha-hydroxy-iso-pravastatin compared with administration of pravastatin alone (59.7 ± 36.6 h*ng/mL vs 76.0 ± 43.5 h*ng/mL for AUC_{0-24}; 20.6 ± 13.1 ng/mL vs 27.0 ± 17.8 ng/mL for C_{max}).

When rosuvastatin was coadministered with 900 mg dalcetrapib, there was a significant increase in C_{max} for rosuvastatin relative to administration of rosuvastatin alone, but rosuvastatin AUC_{0-24} showed little change (Figure 3B; Table III). The mean ± standard deviation values for coadministration compared to

Table IV Pharmacokinetics of Coadministered Simvastatin and Dalcetrapib and Their Metabolites

Analyte/Parameter	Geometric Mean Ratio	90% CI	*P* Value[a]
Dalcetrapib			
AUC_{0-24} (h*ng/mL)	0.792	0.746, 0.842	< .0001
C_{max} (ng/mL)	0.870	0.782, 0.968	.0353
Dalcetrapib-S-Glu			
AUC_{0-24} (h*ng/mL)	0.950	0.788, 1.145	.6328
C_{max} (ng/mL)	0.830	0.698, 0.987	.0785
Dalcetrapib-S-Me			
AUC_{0-24} (h*ng/mL)	0.937	0.886, 0.991	.0589
C_{max} (ng/mL)	0.877	0.748, 1.027	.1674
Simvastatin			
AUC_{0-24} (h*ng/mL)	0.614	0.553, 0.681	< .0001
C_{max} (ng/mL)	0.567	0.479, 0.671	< .0001
Simvastatin acid			
AUC_{0-24} (h*ng/mL)	0.985	0.784, 1.238	.9113
C_{max} (ng/mL)	1.050	0.889, 1.241	.6195

Abbreviations: AUC_{0-24}, area under the concentration-time curve; CI, confidence interval; C_{max}, maximum plasma concentration; dalcetrapib-S-Glu, dalcetrapib-S-glucuronide; dalcetrapib-S-Me, dalcetrapib-S-methyl.
a. For dalcetrapib and metabolites, simvastatin coadministered with dalcetrapib vs dalcetrapib alone; for simvastatin and metabolite, dalcetrapib coadministered with simvastatin vs simvastatin alone.

rosuvastatin alone were 116 ± 49.2 h*ng/mL vs 117 ± 64.7 h*ng/mL for AUC_{0-24} and 15.6 ± 8.45 ng/mL vs 12.3 ± 7.27 ng/mL for C_{max}. For the rosuvastatin metabolites rosuvastatin lactone and N-desmethyl rosuvastatin, there were reductions in AUC_{0-24} (37.8 ± 14 h*ng/mL vs 47.9 ± 19 h*ng/mL for rosuvastatin lactone; 17.7 ± 3.66 h*ng/mL vs 20.2 ± 6.46 h*ng/mL for N-desmethyl rosuvastatin) and in C_{max} (2.37 ± 1.01 ng/mL vs 3.21 ± 1.25 ng/mL for rosuvastatin lactone; 1.11 ± 0.582 ng/mL vs 1.38 ± 0.561 ng/mL for N-desmethyl rosuvastatin) when dalcetrapib was coadministered with rosuvastatin compared with administration of rosuvastatin alone. The changes in AUC_{0-24} and C_{max} for the rosuvastatin metabolites were significant (Table III), except for AUC_{0-24} of N-desmethyl rosuvastatin, which did not undergo statistical analysis owing to the small sample size (n = 5).

Although coadministration of simvastatin with 900 mg dalcetrapib resulted in a statistically significant reduction in the exposure to simvastatin compared with administration of simvastatin alone (26.3 ± 30.8 h*ng/mL vs 45.5 ± 65.4 h*ng/mL for AUC_{0-24}; 7.57 ± 6.11 ng/mL vs 13.1 ± 11.3 ng/mL for C_{max}; Table IV), there was no statistically significant change in exposure to the active metabolite of simvastatin, simvastatin acid (Figure 4B; Table IV).

Pharmacodynamic Results

Effect of coadministration of statins with dalcetrapib on CETP activity and mass: The effect of 900 mg dalcetrapib on inhibiting CETP activity was comparable when administered alone or when coadministered with pravastatin, rosuvastatin, or simvastatin (Table V). In the pravastatin and rosuvastatin studies, administration of 900 mg dalcetrapib alone or coadministered with statins was associated with maximum decreases in CETP activity at 6 hours post-dose on Day 8 compared with pre-dose Day 8. Following administration of pravastatin alone on Day 8, CETP activity was increased (on average ~8%) compared with pre-dose on Day 8 but remained largely unchanged over the 24-hour dosing interval. In the simvastatin study, dalcetrapib alone or coadministered with simvastatin was associated with a decrease in CETP activity at 6 hours post-dose on Day 8 compared with pre-dose on Day 1 (-33% with dalcetrapib alone; -36% with dalcetrapib coadministered with simvastatin; Table V). The decreases in CETP activity with dalcetrapib were accompanied by increases in CETP mass in the rosuvastatin and simvastatin studies; CETP mass was not measured in the pravastatin study. After 7 days of treatment in the rosuvastatin study, pre-dose CETP mass on Day 8 was higher with dalcetrapib than with rosuvastatin (3.27 μg/mL vs 1.72 μg/mL; Table V). Dalcetrapib alone was associated with an increase in CETP mass from Day 1 pre-dose to Day 8 at 6 hours post-dose in the simvastatin study (Table V). There was little effect on CETP activity or mass (<5% change) in the 24 hours following the Day 8 dose of rosuvastatin (data not shown). Similarly, administration of simvastatin alone from Day 1 pre-dose to Day 5 at 6 hours post-dose had little effect on CETP activity or mass (data not shown).

Effect of coadministration of statins with dalcetrapib on plasma lipids: The observed increase in HDL-C typically associated with dalcetrapib was comparable for dalcetrapib administered alone and when coadministered with pravastatin, rosuvastatin, or simvastatin (Table VI). Coadministration of 900 mg dalcetrapib with a statin was associated with a greater reduction in LDL-C compared with a statin alone. For example, the reductions in LDL-C were 25.7% with pravastatin alone compared with 35.3% when pravastatin was coadministered with dalcetrapib. Dalcetrapib alone or in combination with a statin had little effect on VLDL-C, ApoA-I, or ApoA-II

Table V Effect of Coadministration of Statins With Dalcetrapib on Cholesteryl Ester Transfer Protein (CETP) Activity and Mass

	Mean ± SD CETP Activity (%)		Mean ± SD CETP Mass (μg/mL)	
	Dalcetrapib 900 mg Alone	Dalcetrapib 900 mg + Statin	Dalcetrapib 900 mg Alone	Dalcetrapib 900 mg + Statin
Pravastatin study				
Pre-dose Day 8	38.48 ± 4.31[b]	38.09 ± 5.50[b]	NC	NC
6 h post-dose Day 8	30.36 ± 4.97[b]	29.09 ± 5.67[b]	NC	NC
Rosuvastatin study				
Pre-dose Day 8	40.6 ± 3.23[b]	39.9 ± 3.06[b]	3.27 ± 0.67	2.79 ± 0.50
6 h post-dose Day 8	32.2 ± 3.33[b]	31.4 ± 3.33[b]	3.23 ± 0.70	2.77 ± 0.54
Simvastatin study				
Pre-dose Day 1	87.70 ± 13.28	87.12 ± 11.89	1.53 ± 0.28	1.56 ± 0.40
6 h post-dose final day of dosing[a]	58.25 ± 14.28	57.89 ± 15.37	2.7 ± 0.37	2.31 ± 0.43

Abbreviations: NC, not calculated, SD, standard deviation.
a. Final day of dosing = Day 8 for 900 mg dalcetrapib alone; Day 13 for dalcetrapib with simvastatin.
b. Units are pmol/mL/min.

(Table VI). Dalcetrapib alone resulted in small reductions in ApoB (Table VI), and coadministration of dalcetrapib with a statin reduced ApoB levels beyond the reductions observed with the statin alone. For example, the ApoB reductions were 18.4% with pravastatin alone and 25% when pravastatin and dalcetrapib were administered together. The reductions in total cholesterol when dalcetrapib was coadministered with pravastatin, rosuvastatin, or simvastatin were similar to the reductions observed with each of these statins alone (Table VI; data not shown). Triglyceride levels were not notably altered by administration of dalcetrapib alone, by administration of pravastatin alone, or by coadministration of pravastatin and dalcetrapib (Table VI; data not shown). Coadministration of dalcetrapib with rosuvastatin or simvastatin did not have an additional effect beyond the reductions in TG seen with those statins alone (Table VI; data not shown).

Effect of coadministration of simvastatin and dalcetrapib on HDL, LDL, and VLDL particle parameters: The simvastatin study included an analysis of HDL, LDL, and VLDL particle concentration and size. Administration of 900 mg dalcetrapib alone or coadministration of dalcetrapib with simvastatin was associated with only slight decreases in the total concentration of HDL particles, but there were some changes observed in HDL particle subsets (Table VII). Dalcetrapib alone, and when coadministered with simvastatin, was associated with an increase in the concentration of large HDL particles and a decrease in the concentration of medium and small HDL particles, and a resultant small increase in mean HDL size (Table VII).

Simvastatin alone lowered the concentration of LDL particles, and the effect was greater when simvastatin was coadministered with dalcetrapib (Table VIII). Dalcetrapib alone slightly decreased the concentration of LDL particles (Table VIII). Although dalcetrapib alone had little effect on large LDL particles, simvastatin alone decreased the concentration of this subclass, and the combination of dalcetrapib and simvastatin was associated with an even greater reduction (Table VIII). For the rest of the LDL subclasses, there was a similar effect with dalcetrapib alone or when coadministered with simvastatin (Table VIII). There was no apparent change in the mean size of LDL particles on any of the treatments (Table VIII).

Dalcetrapib alone resulted in a reduction in the concentration of VLDL particles compared to baseline, as did simvastatin alone. The coadministration of dalcetrapib with simvastatin resulted in a further decrease in the concentration of VLDL particles. The reduced concentrations of VLDL were attributable to a decrease in the concentration of small VLDL particles (data not shown).

Effect of coadministration of pravastatin or rosuvastatin with dalcetrapib on HMG-CoA reductase activity: HMG-CoA reductase activity was measured in the pravastatin and rosuvastatin studies. In the pravastatin study, maximum inhibitory activity was observed at 2 hours post-dose on Day 8. Maximum inhibitory concentrations for total HMG-CoA reductase activity were 66.84 ± 21.87 ng Eq/mL with pravastatin alone and 48.30 ± 27.79 ng Eq/mL with pravastatin coadministered with dalcetrapib. For active HMG-CoA reductase activity, the maximum inhibitory concentrations were 118.48 ± 97.07 ng Eq/mL with pravastatin alone and

Table VI Effect of Coadministration of 900 mg Dalcetrapib With Statins on Concentrations of Plasma Lipids (Mean ± Standard Deviation)

	Pravastatin Study		Rosuvastatin Study			Simvastatin Study		
	**Dalcetrapib 900 mg Alone**	**Pravastatin Alone**	**Dalcetrapib 900 mg + Pravastatin**	**Dalcetrapib 900 mg Alone**	**Rosuvastatin Alone**	**Dalcetrapib 900 mg + Rosuvastatin**	**Dalcetrapib 900 mg Alone**	**Dalcetrapib 900 mg + Simvastatin**
HDL-C (mg/dL)								
Day 1	46.9 ± 10.75	45.8 ± 11.62	46.07 ± 10.47	48.7 ± 6.88	50.3 ± 7.76	49.8 ± 8.15	44.13 ± 8.25	45.13 ± 10.55
Final day of dosing[a]	51.19 ± 13.14	40.4 ± 9.25	53.79 ± 13.41	58.6 ± 7.66	47.4 ± 6.29	58.7 ± 8.69	53.29 ± 9.18	53.30 ± 10.26
LDL-C (mg/dL)								
Day 1	99.96 ± 23.52	96.39 ± 26.28	95.99 ± 25.58	101 ± 22.8	107 ± 25.3	106 ± 25.9	122.39 ± 31.66	121.62 ± 39.77
Final day of dosing[a]	82.08 ± 24.53	71.84 ± 18.54	62.84 ± 19.58	91.3 ± 26.2	53.8 ± 19.2	44.1 ± 19.1	103.86 ± 30.50	59.85 ± 18.15
LDL-C/ HDL-C ratio								
Day 1	2.2 ± 0.61	2.17 ± 0.65	2.14 ± 0.66	2.12 ± 0.55	2.19 ± 0.64	2.19 ± 0.64	2.72 ± 0.73	2.63 ± 0.90
Final day of dosing[a]	1.67 ± 0.56	1.84 ±0.57	1.22 ± 0.47	1.58 ± 0.49	1.15 ± 0.43	0.76 ± 0.32	1.90 ± 0.60	1.11 ± 0.33
NonHDL-C/ HDL-C ratio								
Day 1	2.63 ± 0.74	2.62 ± 0.76	2.61 ± 0.82	2.35 ± 0.65	2.42 ± 0.72	2.38 ± 0.71	3.11 ± 0.81	3.00 ± 0.95
Final day of dosing[a]	2.13 ± 0.71	2.36 ± 0.72	1.62 ± 0.57	1.78 ± 0.54	1.36 ± 0.50	0.94 ± 0.33	2.25 ± 0.69	1.49 ± 0.44
VLDL-C (mg/dL)								
Day 1	18.73 ± 7.48	18.77 ± 8.04	19.3 ± 9.29	11.0 ± 5.53	11.0 ± 6.08	8.65 ± 5.90	20.08 ± 9.27	18.92 ± 5.41
Final day of dosing[a]	22.02 ± 9.32	19.72 ± 8.23	20.5 ± 9.29	11.6 ± 5.06	9.24 ± 4.12	10.5 ± 2.95	20.46 ± 10.42	20.46 ± 9.65
ApoA-I (g/L)								
Day 1	1.40 ± 0.3	1.31 ± 0.23	1.33 ± 0.26	1.37 ± 0.17	1.37 ± 0.14	1.35 ± 0.13	1.36 ± 0.18	1.40 ± 0.25
Final day of dosing[a]	1.34 ± 0.23	1.21 ± 0.19	1.41 ± 0.26	1.50 ± 0.50	1.34 ± 0.14	1.47 ± 0.16	1.46 ± 0.19	1.46 ± 0.20
ApoA-II (g/L)								
Day 1	0.32 ± 0.04	0.31 ± 0.04	0.31 ± 0.04	0.30 ± 0.03	0.31 ± 0.03	0.31 ± 0.03	NC	NC
Final day of dosing[a]	0.32 ± 0.04	0.3 ± 0.04	0.33 ± 0.05	0.32 ± 0.03	0.31 ± 0.03	0.32 ± 0.03	NC	NC
ApoB (g/L)								
Day 1	0.73 ± 0.16	0.71 ± 0.17	0.71 ± 0.17	0.70 ± 0.15	0.74 ± 0.17	0.73 ± 0.17	0.80 ± 0.22	0.79 ± 0.23
Final day of dosing[a]	0.66 ± 0.14	0.58 ± 0.13	0.53 ± 0.12	0.66 ± 0.16	0.45 ± 0.10	0.32 ± 0.03	0.71 ± 0.20	0.48 ± 0.11
Total cholesterol (mg/dL)								
Day 1	165.59 ± 31.24	160.96 ± 32.45	161.36 ± 30.65	161 ± 26.7	168 ± 27.1	164 ± 27.6	185.33 ± 37.07	185.71 ± 44.02
Final day of dosing[a]	155.3 ± 34	131.96 ± 24.17	137.13 ± 28.9	161 ± 28.3	110 ± 25.3	113 ± 21.9	179.54 ± 39.38	135.52 ± 29.34
TG (mg/dL)								
Day 1	109.15 ± 45.59	109.25 ± 45.57	110.47 ± 43.39	83.3 ± 29.7	81.8 ± 35.5	79.9 ± 30.8	87.54 ± 33.51	82.30 ± 34.32
Final day of dosing[a]	118.98 ± 56.58	109.36 ± 38.93	110.77 ± 48.79	80.6 ± 36.9	64.8 ± 25.1	61.6 ± 19.1	82.75 ± 38.55	63.74 ± 36.71

Abbreviations: Apo, apolipoprotein; HDL-C, high-density lipoprotein cholesterol; LDL-C, low-density lipoprotein cholesterol; NC, not calculated, TG, triglycerides; VLDL-C, very low-density lipoprotein cholesterol.

Note: Day 1 samples taken pre-dose.

a. For the pravastatin study and rosuvastatin study, the final day of dosing was Day 8 (Figure 1A, Treatments A and C) and the sample was taken 24 hours post-dose on Day 9; For the simvastatin study, the final day of dosing was Day 8 for 900 mg dalcetrapib alone (Figure 1B, Treatment B) and Day 13 for 900 mg dalcetrapib + simvastatin (Figure 1B, Treatment A) and the sample was taken 1 hour prior to dosing on that day.

Table VII High-density Lipoprotein (HDL) Particle Parameters (Mean ± Standard Deviation) With Dalcetrapib Alone, Simvastatin Alone, and Coadministration of Dalcetrapib With Simvastatin

	900 mg Dalcetrapib Alone	40 mg Simvastatin Alone	900 mg Dalcetrapib + 40 mg Simvastatin
Total HDL particles (μmol/L)			
Day 1	28.55 ± 2.86	29.10 ± 4.45	29.10 ± 4.45
Final day of dosing[a]	25.86 ± 2.41	28.24 ± 3.09	26.65 ± 4.22
Large HDL (μmol/L)			
Day 1	6.03 ± 2.59	6.47 ± 2.63	6.47 ± 2.63
Final day of dosing[a]	9.91 ± 2.37	5.88 ± 2.76	9.5 ± 2.38
Medium HDL (μmol/L)			
Day 1	5.23 ± 3.13	4.37 ± 2.14	4.37 ± 2.14
Final day of dosing[a]	3.05 ± 2.21	5.47 ± 2.73	3.08 ± 3.08
Small HDL (μmol/L)			
Day 1	17.31 ± 2.89	18.27 ± 3.89	18.27 ± 3.89
Final day of dosing[a]	12.9 ± 2.31	16.91 ± 3.17	14.08 ± 3.49
HDL size (nm)			
Day 1	9 ± 0.35	9.02 ± 0.37	9.02 ± 0.37
Final day of dosing[a]	9.64 ± 0.36	8.97 ± 0.38	9.61 ± 0.39

a. Final day of dosing = Day 8 for 900 mg dalcetrapib alone; Day 5 for 40 mg simvastatin alone; Day 13 for dalcetrapib + simvastatin.

Table VIII Low-density Lipoprotein (LDL) Particle Parameters (Mean ± Standard Deviation) With Dalcetrapib Alone, Simvastatin Alone, and Coadministration of Dalcetrapib with Simvastatin

	900 mg Dalcetrapib Alone	40 mg Simvastatin Alone	900 mg Dalcetrapib + 40 mg Simvastatin
Total LDL particles (μmol/L)			
Day 1	0.99 ± 0.32	0.97 ± 0.33	0.97 ± 0.33
Final day of dosing[a]	0.82 ± 0.26	0.77 ± 0.28	0.62 ± 0.15
Large LDL (μmol/L)			
Day 1	0.40 ± 0.15	0.40 ± 0.17	0.40 ± 0.17
Final day of dosing[a]	0.41 ± 0.15	0.32 ± 0.14	0.22 ± 0.10
Medium-small LDL (μmol/L)			
Day 1	0.12 ± 0.06	0.12 ± 0.06	0.12 ± 0.06
Final day of dosing[a]	0.08 ± 0.05	0.09 ± 0.05	0.08 ± 0.03
Small LDL (μmol/L)			
Day 1	0.55 ± 0.30	0.54 ± 0.30	0.54 ± 0.30
Final day of dosing[a]	0.40 ± 0.22	0.43 ± 0.27	0.39 ± 0.14
Very small LDL (μmol/L)			
Day 1	0.43 ± 0.24	0.42 ± 0.25	0.42 ± 0.25
Final day of dosing[a]	0.32 ± 0.18	0.33 ± 0.22	0.31 ± 0.11
LDL size (nm)			
Day 1	21.31 ± 0.66	21.28 ± 0.63	21.28 ± 0.63
Final day of dosing[a]	21.54 ± 0.53	21.33 ± 0.64	21.03 ± 0.53

a. Final day of dosing = Day 8 for 900 mg dalcetrapib alone; Day 5 for 40 mg simvastatin alone; Day 13 for dalcetrapib + simvastatin.

83.24 ± 127.19 ng Eq/mL with pravastatin coadministered with dalcetrapib. In the rosuvastatin study, maximum total HMG-CoA reductase activity was observed 3 hours following the dose of rosuvastatin and was similar when rosuvastatin was coadministered with dalcetrapib and when rosuvastatin was administered alone (12.78 ± 7.89 ng Eq/mL vs 12.54 ± 9.33 ng Eq/mL), with increases from the pre-dose baseline of 385% and 296%, respectively. Active HMG-CoA reductase activity at 3 hours was also similar for rosuvastatin coadministered with dalcetrapib compared with rosuvastatin alone (10.71 ± 6.22 ng Eq/mL vs 10.63 ± 5.72 ng Eq/mL), with increases from baseline of 296% and 239%, respectively.

Table IX Overview of Adverse Events (AEs) for Studies of 900 mg Dalcetrapib Coadministered With Statins

	n	Number (%) Participants With at Least 1 AE, n (%)	Number of AEs	Number (%) of Participants With ≥1 Severe AE	Number (%) of Participants With ≥1 Treatment-Related AE,[a] n (%)	Withdrawals Owing to AEs	Withdrawals Owing to Treatment-Related AEs,[a] n (%)
Pravastatin study							
Dalcetrapib alone	29	9 (31)	16	0	8 (28)	0	0
Dalcetrapib + pravastatin	29	12 (41)	18	0	9 (31)	0	0
Pravastatin alone	29	13 (45)	23	0	9 (31)	0	0
Rosuvastatin study							
Dalcetrapib alone	30[b]	16 (53)	24	0	5 (17)	0	0
Dalcetrapib + rosuvastatin	30[b]	10 (33)	22	0	5 (17)	0	0
Rosuvastatin alone	30[b]	10 (33)	15	0	6 (20)	0	0
Simvastatin study							
Dalcetrapib alone	24	14 (58)	22	0	0	1 (4)	0
Dalcetrapib + simvastatin	23	14 (61)	29	0	5 (22)	0	0
Simvastatin alone	23	11 (48)	15	0	1 (4)	0	0

a. Assessed as possibly or probably related to study treatment.
b. In the rosuvastatin study, 1 subject dropped out, yielding 30 evaluable patients in the treatment phase of the study.

Table X Elevations in Blood Pressure (BP) for Coadministration of 900 mg Dalcetrapib With Statins

		Number (%) of Participants With BP Elevations	
	n	DBP > 90 mmHg	SBP > 140 mmHg
Pravastatin study			
Dalcetrapib alone	29	1 (3)	4 (14)
Dalcetrapib + pravastatin	29	0	3 (10)
Pravastatin alone	29	0	3 (10)
Rosuvastatin study			
Dalcetrapib alone	30[a]	1 (3)	2 (7)
Dalcetrapib + rosuvastatin	30[a]	1 (3)	2 (7)
Rosuvastatin alone	30[a]	2 (7)	2 (7)
Simvastatin study			
Dalcetrapib alone	24	2 (8)	2 (8)
Dalcetrapib + simvastatin	23	0	2 (9)
Simvastatin alone	23	1 (4)	3 (13)

Abbreviations: DBP, diastolic blood pressure; SBP, systolic blood pressure.
a. In the rosuvastatin study, 1 subject dropped out, yielding 30 evaluable patients in the treatment phase of the study.

Safety

Adverse events: Dalcetrapib alone and in combination with statins was generally well tolerated (Table IX). The most common AE reported in each of the 3 open-label studies was headache. In the pravastatin study, headache was reported by a similar percentage of participants in each treatment group (13.8% for dalcetrapib alone and pravastatin alone, 10.3% for dalcetrapib coadministered with pravastatin). In the rosuvastatin study, headache was more common with dalcetrapib alone (23.3%) and with dalcetrapib coadministered with rosuvastatin (20.0%) than with rosuvastatin alone (10.0%). In the simvastatin study, headache was less frequent with dalcetrapib alone (8.3%) than with simvastatin alone (13.0%) or with dalcetrapib coadministered with simvastatin (17.4%). The majority of the AEs were mild in intensity and were considered either unrelated or remotely related to treatment except in the pravastatin study, where the percentage of AEs considered possibly related to treatment by the investigator were similarly high among the different treatment groups (81.3% of AEs with dalcetrapib alone; 72.2% of AEs with dalcetrapib coadministered with pravastatin; 69.6% with pravastatin alone). There were 2 cases of myalgia during administration of pravastatin alone, 2 during coadministration of pravastatin with dalcetrapib, 2 during administration of rosuvastatin alone, and 1 during administration of dalcetrapib alone in the rosuvastatin study; all cases of myalgia were considered by the investigators to be mild in intensity and possibly related to trial treatment. No cases of myalgia were accompanied by creatine phosphokinase (CPK) elevations. There were no serious AEs in any of the studies.

Laboratory tests: In the pravastatin study, 3 participants had CPK elevations (1 during dalcetrapib alone, 1 during pravastatin alone, and 1 during each of the 3 treatments), and 1 participant taking dalcetrapib alone had elevated bicarbonate levels. No CPK elevations were >3× upper limit of normal (ULN). In the rosuvastatin study, 1 participant was withdrawn from treatment owing to elevated alanine aminotransferase (ALT) and moderate hepatomegaly, but these events were not considered related to study treatment. Three other participants also experienced laboratory abnormalities (elevated CPK >2× ULN on Day -1 of rosuvastatin alone, elevated ALT during rosuvastatin alone, and elevated ALT during coadministration of dalcetrapib with rosuvastatin). The increase in CPK was transient, with levels returning to the standard reference range on Day 4 of rosuvastatin treatment; this abnormality was not considered likely to be related to study treatment. None of the laboratory abnormalities in the rosuvastatin study were considered clinically significant by the investigator. In the simvastatin study, marked CPK elevations were reported in 2 participants following administration of dalcetrapib plus simvastatin; both patients also had abnormal CPK values reported during screening. Creatine phosphokinase elevations were also reported in 2 participants in the simvastatin study at the follow-up visit and in 1 participant at Day -1 of treatment with dalcetrapib alone, with a slight elevation at Day 8 of treatment and an elevation again at follow-up. There were no clinically relevant changes in laboratory parameters observed in the simvastatin study. No participants withdrew from the pravastatin or simvastatin studies as a result of abnormalities in laboratory parameters.

Blood pressure and ECG parameters: The frequency of blood pressure elevations was low in each of the 3 studies (Table X). Mean blood pressure increases over the duration of each of the studies were not apparently different between the different treatment arms. In the studies of pravastatin and rosuvastatin coadministered with 900 mg dalcetrapib, there were no QTcB intervals greater than 450 ms. QTcB intervals from 450 to 480 ms occurred in 2 participants in the simvastatin study (1 participant receiving dalcetrapib alone; 1 participant receiving simvastatin alone). An abnormal ECG reading (decreased T-wave amplitude) led to withdrawal of 1 participant during coadministration of simvastatin with dalcetrapib.

DISCUSSION

Current guidelines for the prevention of CVD events include lowering LDL-C with statins. Drug-drug interactions between cytochrome (CYP) 3A inhibitors and some statins may result in increased statin exposure, which is associated with serious side effects such as myopathy and rhabdomyolysis.[13] Because dalcetrapib, a new agent being investigated for the prevention of CVD events, will likely be coadministered with statins, it was important to determine whether coadministration of statins with dalcetrapib is associated with any pharmacokinetic interactions or effects on efficacy or safety.

Except for a 26% increase in rosuvastatin C_{max} ($P = .0132$), there was no significant increase in the pharmacokinetic parameters of statins or their active metabolites. To the contrary, exposure to pravastatin and simvastatin was significantly reduced when coadministered with dalcetrapib, although exposure to the active metabolite of simvastatin, simvastatin acid, was not significantly altered by coadministration. Except for pravastatin and rosuvastatin, most statins, including simvastatin, are metabolized by CYP450 enzymes.[13] Two previous Phase I studies, 1 using a panel of CYP substrates and 1 with rosiglitazone, showed that dalcetrapib had no effect on the major CYP isoforms.[14] Another Phase I study using the strong CYP3A4 inhibitor ketoconazole showed that dalcetrapib, unlike most statins, is not a substrate for CYP3A4.[15]

In addition, in the studies presented here, there was no increase in dalcetrapib exposure when statins were coadministered with dalcetrapib. Dalcetrapib exposure was not significantly changed by coadministration with pravastatin and was significantly lower with coadministration of rosuvastatin or simvastatin, versus monotherapy with dalcetrapib. However, the effect of dalcetrapib on raising HDL-C was not compromised by coadministration with the statins: there was a comparable increase in HDL-C with dalcetrapib administered alone or coadministered with pravastatin, rosuvastatin, or simvastatin. In the simvastatin study, which used nuclear magnetic resonance spectroscopy to measure HDL particle subsets, there was an increase in the concentration of large HDL particles and a decrease in the concentration of medium and small HDL particles observed with dalcetrapib alone and dalcetrapib coadministered with simvastatin.

Coadministration of dalcetrapib with the statins was associated with a greater reduction in LDL-C compared with administration of the statins alone.

Phase II studies of dalcetrapib in combination with pravastatin or simvastatin have also indicated effective LDL-C lowering in patients with dyslipidemia or with coronary heart disease or coronary heart disease risk equivalents.[11,16,17] In addition, the current study showed decreases in the concentrations of total LDL particles and large LDL particles when dalcetrapib was coadministered with simvastatin. Dalcetrapib alone and coadministered with simvastatin was also associated with reductions in the concentrations of small and very small LDL particles.

The studies presented here raised no substantial safety concerns regarding coadministration of pravastatin, rosuvastatin, or simvastatin with dalcetrapib. Incidences of AEs were similar for dalcetrapib alone, statins administered with dalcetrapib, and statins alone. The safety and tolerability profile observed with dalcetrapib in this study of healthy subjects is consistent with that reported in previous placebo-controlled trials of 4, 12, or 48 weeks' duration of dalcetrapib alone or in combination with statins in patients with dyslipidemia, CHD, or CHD risk equivalents.[11,16]

Three previous drug-drug interaction studies have shown no clinically relevant interactions between dalcetrapib and CYP substrates or the CYP3A4 inhibitor ketoconazole.[14,15] The 3 studies presented here further characterize the drug-drug interaction profile of dalcetrapib. The lack of substantial, clinically relevant differences in exposure or safety with coadministration compared with dalcetrapib or statins alone indicates no clinically significant drug-drug interaction when dalcetrapib is coadministered with pravastatin, rosuvastatin, or simvastatin, or their metabolites. The effect on lipid levels in these studies reflected that of the individual compounds with some additive benefit.

Editorial assistance was provided by Lisa Cimakasky (Prime Medica Inc., New York, NY). The studies were funded by F. Hoffmann-La Roche Ltd.

Financial disclosures: Michael Derks, Markus Abt, Mary Phelan, Lynn Turnbull, Georgina Meneses-Lorente, Nuria Bech, Anne-Marie White, and Graeme Parr are employees of F. Hoffmann-La Roche Ltd, Basel, Switzerland.

REFERENCES

1. Baigent C, Keech A, Kearney PM, et al. Efficacy and safety of cholesterol-lowering treatment: prospective meta-analysis of data from 90,056 participants in 14 randomised trials of statins. *Lancet*. 2005;366:1267-1278.

2. Fruchart JC, Sacks F, Hermans MP, et al. The Residual Risk Reduction Initiative: a call to action to reduce residual vascular risk in patients with dyslipidemia. *Am J Cardiol*. 2008;102(10 Suppl):1K-34K.

3. Goldbourt U, Yaari S, Medalie JH. Isolated low HDL cholesterol as a risk factor for coronary heart disease mortality. A 21-year follow-up of 8000 men. *Arterioscler Thromb Vasc Biol*. 1997;17:107-113.

4. Schaefer EJ, Lamon-Fava S, Ordovas JM, et al. Factors associated with low and elevated plasma high density lipoprotein cholesterol and apolipoprotein A-I levels in the Framingham Offspring Study. *J Lipid Res*. 1994;35:871-882.

5. Assmann G, Schulte H, von Eckardstein A, Huang Y. High-density lipoprotein cholesterol as a predictor of coronary heart disease risk. The PROCAM experience and pathophysiological implications for reverse cholesterol transport. *Atherosclerosis*. 1996;124(Suppl):S11-S20.

6. Robins SJ, Collins D, Wittes JT, et al; VA-HIT Study Group. Veterans Affairs High-Density Lipoprotein Intervention Trial. Relation of gemfibrozil treatment and lipid levels with major coronary events: VA-HIT: a randomized controlled trial. *JAMA*. 2001;285:1585-1591.

7. Canner PL, Furberg CD, McGovern ME. Benefits of niacin in patients with versus without the metabolic syndrome and healed myocardial infarction (from the Coronary Drug Project). *Am J Cardiol*. 2006;97:477-479.

8. Goldenberg I, Goldbourt U, Boyko V, Behar S, Reicher-Reiss H; BIP Study Group. Relation between on-treatment increments in serum high-density lipoprotein cholesterol levels and cardiac mortality in patients with coronary heart disease (from the Bezafibrate Infarction Prevention trial). *Am J Cardiol*. 2006;97:466-471.

9. Inazu A, Brown ML, Hesler CB, et al. Increased high-density lipoprotein levels caused by a common cholesteryl-ester transfer protein gene mutation. *N Engl J Med*. 1990;323:1234-1238.

10. Boekholdt SM, Kuivenhoven JA, Wareham NJ, et al. Plasma levels of cholesteryl ester transfer protein and the risk of future coronary artery disease in apparently healthy men and women: the prospective EPIC (European Prospective Investigation into Cancer and nutrition)-Norfolk population study. *Circulation*. 2004;110:1418-1423.

11. Stein EA, Stroes ESG, Steiner G, et al. Safety and tolerability of dalcetrapib. *Am J Cardiol*. 2009;104:82-91.

12. de Grooth GJ, Kuivenhoven JA, Stalenhoef AF, et al. Efficacy and safety of a novel cholesteryl ester transfer protein inhibitor, JTT-705, in humans: a randomized phase II dose-response study. *Circulation*. 2002;105:2159-2165.

13. Neuvonen PJ, Niemi M, Backman JT. Drug interactions with lipid-lowering drugs: mechanisms and clinical relevance. *Clin Pharmacol Ther*. 2006;80:565-581.

14. Derks M, Fowler S, Kuhlmann O. In vitro and in vivo assessment of the effect of dalcetrapib on a panel of CYP substrates. *Curr Med Res Opin*. 2009;25:891-902.

15. Derks M, Fowler S, Kuhlmann O. A single-center, open-label, one-sequence study of dalcetrapib coadministered with ketoconazole, and an in vitro study of the S-methyl metabolite of dalcetrapib. *Clin Ther*. 2009;31:586-599.

16. Stein EA, Roth EM, Rhyne JM, et al. Safety and tolerability of dalcetrapib (RO4607381/JTT-705): results from a 48-week trial. *Am J Cardiol*. 2009;104:82-91.

17. Kuivenhoven JA, de Grooth GJ, Kawamura H, et al. Effectiveness of inhibition of cholesteryl ester transfer protein by JTT-705 in combination with pravastatin in type II dyslipidemia. *Am J Cardiol*. 2005;95:1085-1088.

For reprints and permission queries, please visit SAGE's Web site at http://www.sagepub.com/journalsPermissions.nav.
