# Supplementary material for: Lipid-lowering effect of combined therapy with high-intensity statins and CETP inhibitors: a Systematic Review and meta-analysis
Source: Front Endocrinol (Lausanne). 2025 May 1;16:1512670. doi: 10.3389/fendo.2025.1512670 (PMC12078159; doi:10.3389/fendo.2025.1512670)
Supplement: Supplementary file 1 [file DataSheet1.zip › Raw Data/Raw Data/Original Documentation Fruit/Nicholls2022✔.pdf]

ARTICLES
https://doi.org/10.1038/s41591-022-01936-7

nature medicine

Check for updates

Lipid lowering effects of the CETP inhibitor obicetrapib in combination with high-intensity statins: a randomized phase 2 trial

Stephen J. Nicholls[1]✉, Marc Ditmarsch[2], John J. Kastelein[3], Scott P. Rigby[4], Douglas Kling[2], Danielle L. Curcio[2], Nicholas John Alp[5] and Michael H. Davidson[2]

Global guidelines for the management of high-cardiovascular-risk patients include aggressive goals for low-density lipoprotein cholesterol (LDL-C). Statin therapy alone is often insufficient to reach goals and nonstatin options have limitations. Here, we tested the lipid-lowering effects of the cholesteryl ester transfer protein (CETP) inhibitor drug obicetrapib in a randomized, double-blind, placebo-controlled trial in dyslipidaemic patients ($n = 120$, median LDL-C 88 mg dl^{-1}) with background high-intensity statin treatment (NCT04753606). Over the course of 8 weeks, treatment with 5 mg or 10 mg obicetrapib resulted in a significant decrease as compared with placebo in median LDL-C concentration (by up to 51%; $P < 0.0001$), the primary trial outcome. As compared with placebo, obicetrapib treatment also significantly ($P < 0.0001$) decreased apolipoprotein B (by up to 30%) and non-high-density lipoprotein cholesterol (non-HDL-C) concentration (by up to 44%), and significantly ($P < 0.0001$) increased HDL-C concentration (by up to 165%; the secondary trial outcomes) and had an acceptable safety profile. These results support the potential of obicetrapib to address an unmet medical need for high-cardiovascular-risk patients.

Elevated LDL-C is a major risk factor for atherosclerotic cardiovascular disease (ASCVD) and the cornerstone of risk-reduction strategies[1,2]. Statins are generally the drugs of first choice in treating dyslipidaemia but, despite treatment with high-intensity statins, a proportion of patients do not achieve acceptable LDL-C levels with statin monotherapy[3]. Recent results from GOULD, a prospective observational registry study of patients with ASCVD, showed that most had suboptimal LDL-C levels at baseline and just 17.1% had intensification of their lipid-lowering therapy after 2 years[4]. Furthermore, two thirds of these patients continued to have an LDL-C concentration >70 mg dl^{-1} (ref. [4]). Results from DA VINCI, a cross-sectional, observational study in Europe, showed similar substantial gaps between clinical guidelines and clinical practice for lipid management[5]. Several adjunctive LDL-C-lowering drug therapies are available, such as ezetimibe, bempedoic acid and proprotein convertase subtilisin kexin type 9 (PCSK9) inhibitors. However, these have several limitations, such as limited LDL-C lowering, potential safety concerns and reduced patient access. Additional therapies that substantially reduce LDL-C, and have favorable safety and patient acceptability profiles, including convenience of oral dosing, are urgently needed.

Cholesteryl ester transfer protein (CETP), a plasma glycoprotein produced in the liver and adipose tissue that circulates in the blood bound primarily to HDL, mediates the transfer of cholesteryl esters from HDL to apolipoprotein (Apo) B-containing particles, for example, very low-density lipoprotein (VLDL) and low-density lipoprotein (LDL) particles, in exchange for triglycerides (TGs)[6–8]. Inhibiting CETP raises HDL cholesterol (HDL-C) and, in the case of potent CETP inhibition, also lowers LDL-C[6–8]. Several CETP-inhibitor drugs have been developed, but, to date, none has received market authorization largely due to compound or study-related failures[9]. CETP-inhibiting therapies were originally developed based on the premise that increasing HDL-C levels would prevent cardiovascular events[6,10,11]. However, results from clinical trials and Mendelian randomization data have suggested that cardiovascular benefits are uniquely related to changes in the concentration of Apo-B-containing particles (including LDL particles)[6,12].

Obicetrapib is a selective CETP inhibitor undergoing clinical development for reducing both LDL-C and the incidence of major adverse cardiovascular events[3–15]. CETP inhibition by obicetrapib in patients with mild dyslipidaemia (TULIP), a study of 364 subjects conducted in Denmark and the Netherlands, found that a daily dose of 10 mg obicetrapib in combination with moderate-intensity statins (20 mg atorvastatin or 10 mg rosuvastatin) for 12 weeks resulted in an incremental LDL-C reduction of up to 50% compared with statin monotherapy[14].

In contrast to the results observed in TULIP, results from a Mendelian randomization analysis suggested that combined exposure to variants in the genes that encode the targets of CETP inhibitors and statins was associated with discordant reductions in LDL-C and Apo B levels, and that the corresponding risk reduction was proportional to Apo B but less proportional to LDL-C[12]. Thus, there was concern that the potential clinical benefit of obicetrapib, when specifically used in combination with high-intensity statins, might be attenuated[12,16]. Another concern relates to the fact that some data suggest that LDL-C lowering by CETP inhibitors cannot be reliably assessed by the Friedewald equation but instead requires preparative ultracentrifugation (PUC)[17].

The primary objective of this trial was to evaluate the efficacy of 8-week dosing of obicetrapib 5 mg and 10 mg, compared with placebo, as an adjunct to high-intensity statin therapy for decreasing LDL-C and Apo B. The second objective was to evaluate other lipoprotein lipid and apolipoprotein responses, safety and tolerability profiles, and plasma concentrations of obicetrapib during treatment

[1]Victorian Heart Institute, Monash University, Clayton, Victoria, Australia. [2]NewAmsterdam Pharma B.V., Naarden, Netherlands. [3]Academic Medical Center, Amsterdam, Netherlands. [4]Summit Research Group, Stow, Ohio, USA. [5]Medpace, Cincinnati, Ohio, USA. ✉e-mail: Stephen.Nicholls@monash.edu

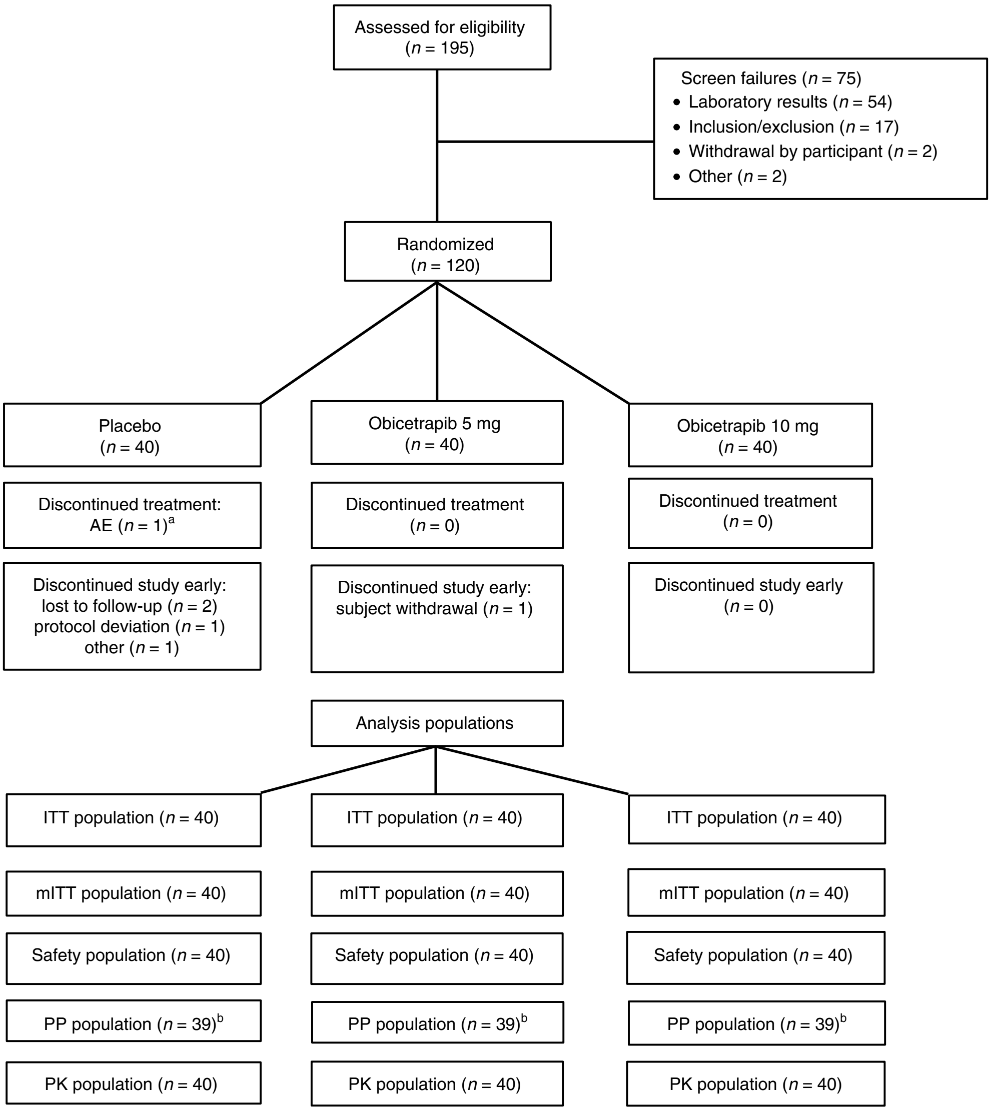

Fig. 1 | Patient flowchart. [a]AE was moderate arthralgia. [b]Subjects were excluded from the PP population because of either no week 8 LDL-C assessment ($n=1$ each in the placebo and obicetrapib 5 mg groups) or because the week 8 LDL-C assessment occurred more than 5 days after the obicetrapib dose ($n=1$ in the obicetrapib 10 mg group). AE, adverse event; ITT, intent to treat; PK, pharmacokinetic; PP, per protocol.

and for several weeks of follow up. Lastly, in this study we aimed to determine whether various quantitative measures of LDL-C levels produced differences in the treatment effects of CETP inhibition.

Results

Subjects. Of the 120 subjects randomized, 119 (99.2%) completed treatment; the modified intent-to-treat (mITT) population included all 120 subjects (Fig. 1). Subjects had a mean age of 61.8 years, were 55.8% male and 76.7% White, and had an average body mass index (BMI) of 31.1 kg m^{-2} (Table 1). All patients were treated with high-intensity statins. Most were taking atorvastatin 40 mg (54.2%), whereas 24.2%, 8.3% and 13.3% of subjects were taking atorvastatin 80 mg, rosuvastatin 20 mg and rosuvastatin 40 mg, respectively.

Lipoprotein lipids. LDL-C, Apo B, HDL-C and lipoprotein(a) (Lp(a)) concentrations at baseline and after 4 and 8 weeks of treatment (only after 8 weeks of treatment for Lp(a)) are shown in Fig. 2, and lipoprotein, lipid, apolipoprotein and Lp(a) concentrations at baseline and percent changes from baseline to the end of treatment are presented in Table 2. Obicetrapib 5 mg and 10 mg each significantly reduced LDL-C compared with placebo ($P<0.0001$). In the primary analysis, which used the Friedewald formula to calculate LDL-C, LDL-C was reduced from baseline by 42.9% and 45.7% for 5 mg and 10 mg obicetrapib, respectively, compared with 0.0% for placebo. Results for LDL-C measured by PUC (also called beta-quantification), which was conducted to evaluate potential discordant results using this 'gold standard' method for LDL-C determination, were comparable to those from the Friedewald formula: −41.5% and −50.8% for 5 mg and 10 mg obicetrapib, respectively, compared with −6.50% for placebo. In addition, we calculated the LDL-C reductions for the 5 mg and 10 mg doses of obicetrapib with the Martin–Hopkins equation, a recently published alternative to the Friedewald formula[18], and they were comparable again to the beta-quantification results: −42.5% and −49.2%, respectively. Additional sensitivity analyses including mixed model repeated measures (MMRM) with imputation and analysis of covarianace (ANCOVA), produced similar results, as did analysis of the per protocol (PP) population ($n=117$).

Obicetrapib 5 mg and 10 mg also significantly reduced Apo B by 24.4% and 29.8%, non-HDL-C by 38.9% and 44.4%, and Lp(a) by 33.8% and 56.5%, respectively, ($P<0.0001$) and significantly increased HDL-C by 135% and 165% and Apo A1 by 44.6% and

Table 1 | Demographic and baseline characteristics

Characteristic[a]	Placebo ($n=40$)	Obicetrapib 5 mg ($n=40$)	Obicetrapib 10 mg ($n=40$)
Age (yr)	61.3 ± 8.77	61.1 ± 8.13	62.9 ± 8.48
Sex, *n* (%)			
Male	19 (47.5)	23 (57.5)	25 (62.5)
Female[b]	21 (52.5)	17 (42.5)	15 (37.5)
Ethnicity, *n* (%)			
Hispanic or Latino	3 (7.5)	5 (12.5)	6 (15.0)
Not Hispanic or Latino	37 (92.5)	35 (87.5)	34 (85.0)
Race, *n* (%)			
White	32 (80.0)	30 (75.0)	30 (75.0)
Black/African American	7 (17.5)	10 (25.0)	5 (12.5)
Asian	1 (2.5)	0 (0.0)	5 (12.5)
BMI (kg m^{-2})	30.2 ± 5.05	32.2 ± 4.24	30.8 ± 4.53
LDL-C category, *n* (%)			
<100 mg dl^{-1}	25 (62.5)	25 (62.5)	24 (60.0)
≥100 mg dl^{-1}	15 (37.5)	15 (37.5)	16 (40.0)
Current statin therapy, *n* (%)			
Atorvastatin 40 mg	20 (50.0)	20 (50.0)	25 (62.5)
Atorvastatin 80 mg	8 (20.0)	10 (25.0)	11 (27.5)
Rosuvastatin 20 mg	2 (5.0)	5 (12.5)	3 (7.5)
Rosuvastatin 40 mg	10 (25.0)	5 (12.5)	1 (2.5)

[a]Values are mean ± standard deviation unless otherwise indicated. [b]Each of the obicetrapib treatment groups included one woman of childbearing potential.

47.8%, respectively, compared with placebo ($P<0.0001$). TG and VLDL-C (measured by PUC; beta-quantification) were both mildly but significantly reduced by obicetrapib 5 mg compared with placebo (−11.0% and −11.5%, respectively) ($P<0.05$), but the comparison of obicetrapib 10 mg with placebo was not significantly different (Table 2). A post-hoc analysis indicated that in individuals with baseline Apo B > 100 mg/dl, obicetrapib 10 mg yielded median Apo B reductions of 39% from baseline.

Safety. Treatment-emergent adverse events were reported by a total of 42 (35.0%) of the 120 subjects in the safety population: 15 subjects (37.5%) and 8 subjects (20.0%) in the obicetrapib 5 mg and 10 mg groups, respectively, compared with 19 subjects (47.5%) in the placebo group. The adverse events reported by at least two subjects in any treatment group are shown in Table 3. The most prevalent adverse events were gastrointestinal disorders (primarily nausea) and nervous system disorders (primarily headache). A majority of events were classified as mild or moderate in severity; 1 subject (2.5%) in the placebo group had a severe adverse event (Covid-19 pneumonia). Six events reported by 4 subjects (10.0%) in the placebo group, 2 events reported by 2 subjects (5.0%) in the obicetrapib 5 mg group and 1 event reported by 1 subject (2.5%) in the obicetrapib 10 mg group, were considered to be study-drug related. Two subjects (5.0%), both in the placebo group, had serious adverse events (1 had Covid-19 pneumonia (mentioned above) and 1 had a transient ischemic attack). Neither was considered related to the study drug. One subject (2.5%) in the placebo group had moderate arthralgia that led to discontinuation of the study drug but was not considered to be related to study drug. There were no signals in any laboratory parameters, that is, no clinically meaningful shifts in chemistry, hematology or urinalysis parameters, and no changes in vital signs in either obicetrapib treatment group compared with placebo.

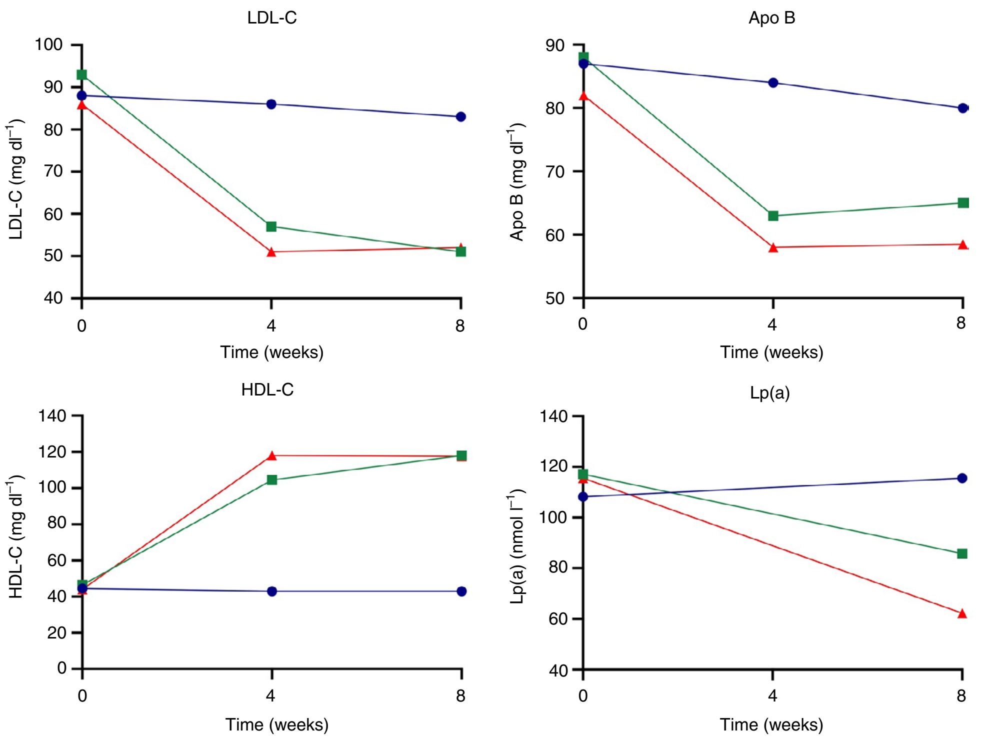

Fig. 2 | Median lipoprotein lipid concentrations. a–d, LDL-C concentrations measured by the Friedewald formula (**a**), Apo B (**b**), HDL-C (**c**) and Lp(a) (**d**) for the placebo (blue), obicetrapib 5 mg (green) and obicetrapib 10 mg (red) groups ($n=40$ each), administered on a background of high-intensity statin treatment at baseline and after 4 and 8 weeks of treatment (only after 8 weeks of treatment for Lp(a)).

Table 2 | Lipoprotein, lipid and apolipoprotein concentrations at baseline and percent changes from baseline to week 8

Lipid and time point	Placebo ($n=40$)[a]	Obicetrapib 5 mg ($n=40$)[a]	Obicetrapib 10 mg ($n=40$)
**LDL-C (Friedewald)**[b]			
Baseline (mg dl^{-1})			
Median (min, max)	87.5 (63, 210)	93.0 (57, 224)	86.0 (39, 211)
Week 8 change (%)			
Median (min, max)	0.00 (−57.9, 40.3)	−42.9 (−81.9, 72.4)	−45.7 (−78.0, 26.2)
LS mean ± s.e.	−5.00 ± 3.50	−38.0 ± 3.49	−44.2 ± 3.46
P value versus placebo		<0.0001	<0.0001
**LDL-C (PUC)**[c]			
Baseline (mg dl^{-1})			
Median (min, max)	90.0 (63, 204)	95.0 (54, 236)	88.0 (39, 207)
Week 8 change (%)			
Median (min, max)	−6.50 (−53.9, 31.6)	−41.5 (−71.2, 62.3)	−50.8 (−76.9, 15.6)
LS mean ± s.e.	−6.70 ± 3.26	−35.7 ± 3.31	−46.8 ± 3.23
P value versus placebo		<0.0001	<0.0001
**LDL-C (Martin–Hopkins)**[b]			
Baseline (mg dl^{-1})			
Median (min, max)	92.0 (69, 207)	95.0 (55, 229)	86.5 (35, 210)
Week 8 change (%)			
Median (min, max)	−2.90 (−55.3, 36.8)	−42.5 (−80.8, 74.4)	−49.2 (−77.0, 28.1)
LS mean ± s.e.	−4.94 ± 3.69	−39.6 ± 3.68	−46.9 ± 3.65
P value versus placebo		<0.0001	<0.0001
**Apo B**[b]			
Baseline (mg dl^{-1})			
Median (min, max)	87.0 (66, 136)	88.0 (53, 171)	82.0 (49, 161)
Week 8 change (%)			
Median (min, max)	−2.60 (−50.0, 28.4)	−24.4 (−58.5, 47.4)	−29.8 (−58.4, 13.0)
LS mean ± s.e.	−4.13 ± 2.59	−22.4 ± 2.58	−28.1 ± 2.56
P value versus placebo		<0.0001	<0.0001
**Non-HDL-C**[b]			
Baseline (mg dl^{-1})			
Median (min, max)	115 (87, 227)	119 (69, 276)	113 (53, 242)
Week 8 change (%)			
Median (min, max)	−3.50 (−50.3, 48.4)	−38.9 (−65.6, 66.3)	−44.4 (−70.2, 22.5)
LS mean ± s.e.	−3.83 ± 3.19	−34.4 ± 3.18	−39.9 ± 3.15
P value versus placebo		<0.0001	<0.0001
**HDL-C**[b]			
Baseline (mg dl^{-1})			
Median (min, max)	44.5 (19, 99)	46.5 (24, 79)	44.0 (25, 138)
Week 8 change (%)			
Median (min, max)	−4.90 (−30.3, 28.6)	135 (−26.4, 213)	165 (55.1, 286)
LS mean ± s.e.	−6.98 ± 6.62	122 ± 6.59	157 ± 6.54
P value versus placebo		<0.0001	<0.0001
**Apo A1**[b]			
Baseline (mg dl^{-1})			
Median (min, max)	152 (92, 235)	157 (101, 215)	151 (90, 324)
Week 8 change (%)			
Median (min, max)	0.00 (−28.9, 22.6)	44.6 (−10.0, 87.1)	47.8 (13.5, 106)
LS mean ± s.e.	−3.46 ± 2.96	41.1 ± 3.04	52.3 ± 3.04

Continued

Table 2 | Lipoprotein, lipid and apolipoprotein concentrations at baseline and percent changes from baseline to week 8 (continued)

Lipid and time point	Placebo ($n=40$)[a]	Obicetrapib 5 mg ($n=40$)[a]	Obicetrapib 10 mg ($n=40$)
P value versus placebo		<0.0001	<0.0001
**TG**[b]			
Baseline (mg dl^{-1})			
Median (min, max)	130 (45, 254)	128 (46, 478)	123 (41, 509)
Week 8 change (%)			
Median (min, max)	1.50 (−44.1, 139)	−11.0 (−53.3, 86.7)	−7.85 (−63.7, 129)
LS mean ± s.e.	8.88 ± 5.33	−6.63 ± 5.32	2.45 ± 5.27
P value versus placebo		0.0417	0.3925
**VLDL-C**[b]			
Baseline (mg dl^{-1})			
Median (min, max)	26.0 (9, 51)	25.5 (9, 59)	24.5 (8, 57)
Week 8 change (%)			
Median (min, max)	0.00 (−43.5, 139)	−11.5 (−53.5, 83.3)	−7.90 (−44.4, 125)
LS mean ± s.e.	9.84 ± 5.26	−6.02 ± 5.24	3.08 ± 5.19
P value versus placebo		0.0347	0.3620
**Lp(a)**[b]			
Baseline (nmol l^{-1})			
Median (min, max)	45.3 (2.9, 410)	89.4 (2.8, 354)	29.9 (2.8, 435)
Week 8 change (%)			
Median (min, max)	4.00 (−29.6, 45.5)	−33.8 (−84.6, 93.8)	−56.5 (−85.7, 18.3)
LS mean ± s.e.	5.06 ± 4.36	−30.9 ± 4.43	−42.0 ± 4.33
P value versus placebo		<0.0001	<0.0001

[a]For percent change values, $n=39$ for the placebo and obicetrapib 5 mg groups for all measurements, except for the 5 mg obicetrapib group, $n=38$ for LDL-C (PUC) and Lp(a) and $n=37$ for Apo A1. [b]LS mean ± s.e. and *P* values are from an MMRM model with treatment, visit and treatment-by-visit as factors and the baseline value as a covariate. Two-sided 95% CIs for each treatment group and for the pairwise comparisons of each dose of obicetrapib to the placebo group were determined. Adjustment for multiple comparisons was made using Dunnett's test for Friedewald-calculated LDL-C; no adjustment was made for multiplicity in testing the other endpoints and no imputation was made. Missing at random was assumed for all missing data at scheduled visits. [c]LS mean ± s.e. and *P* values are from an ANCOVA model, with treatment group as a factor and baseline LDL-C (PUC) as a covariate. Two-sided 95% CIs for each treatment group and for the pairwise comparisons of each dose of obicetrapib to the placebo group were determined; no adjustment was made for multiplicity in testing the other endpoints and no imputation was made. Missing at random was assumed for all missing data at scheduled visits. LS, least squares; max, maximum; min, minimum; s.e., standard error.

Pharmacokinetics. Pharmacokinetic (PK) assessment following end of treatment showed that the drug was nearly completely cleared from circulation by 4–8 weeks post-treatment. Plasma obicetrapib levels decreased by medians of 92%, 98% and 99% at 4, 8 and 15 weeks after the end of treatment, respectively, in the obicetrapib 5 mg group, and by 93%, 98% and 99%, respectively, in the obicetrapib 10 mg group.

Discussion

In this trial of subjects with dyslipidaemia (LDL-C > 70 mg dl^{-1}) receiving high-intensity statin therapy (40 mg or 80 mg atorvastatin or 20 mg or 40 mg rosuvastatin), 8 weeks of add-on treatment of obicetrapib 5 mg and 10 mg significantly reduced LDL-C, Apo B, non-HDL-C and Lp(a), and significantly increased HDL-C and Apo A1. Maximum LDL-C reductions were already present after 4 weeks of treatment. In the TULIP study, patients with mild dyslipidaemia who received 1 mg, 2.5 mg, 5 mg or 10 mg obicetrapib for 12 weeks had LDL-C reductions of 27%, 33%, 45% and 45%, respectively, and those who received 10 mg obicetrapib plus 20 mg atorvastatin or plus 10 mg rosuvastatin had LDL-C reductions of 68% and 63%, respectively[14]. However, that study design made it impossible to calculate the additional LDL-C lowering that might be achieved with a given dose of obicetrapib on top of statin and, furthermore, did not examine the combination of obicetrapib with high-intensity statins. Thus, the present results extend beyond previous findings for obicetrapib administered as monotherapy and in combination with moderate-intensity statins to demonstrate its lipid-altering efficacy at both 5 mg and 10 mg doses in combination with high-intensity statins.

Evidence from randomized clinical trials and Mendelian randomization studies of lipid-altering therapies indicates a causal relationship between lowering LDL-C and reduced risk of ASCVD[19]. A Mendelian randomization analysis by Ference et al[12] further examined the association between changes in lipoproteins and the risk of major cardiovascular events related to variants in the *CETP* gene alone and in combination with variants in the 3-hydroxy-3-methylglutaryl-coenzyme A reductase (*HMGCR*) gene. This analysis demonstrated that, when evaluated alone, the *CETP* score was associated with higher HDL-C, as well as lower LDL-C, concordantly lower Apo B and a correspondingly lower risk of major cardiovascular events, which was similar in magnitude to the relation between the *HMGCR* score and risk of major cardiovascular events per unit LDL-C (and Apo B) change. However, when the *CETP* score and the *HMGCR* score were combined, the *CETP* score was associated with the same LDL-C reduction, but an attenuated Apo B response and a corresponding attenuated nonsignificant cardiovascular risk. These results suggested that combined exposure to gene variants that reduced both CETP and HMG coenzyme A reductase was associated with discordant LDL-C and Apo B reductions, and that reducing risk of cardiovascular events was proportional to the effects on Apo B, and less so for LDL-C. The results from TULIP and ROSE contradict the supposition that combining obicetrapib with moderate and high-intensity statin might significantly attenuate Apo B lowering.

Table 3 | Adverse events occurring in at least two subjects for any treatment condition

System organ class preferred term[a]	Placebo ($n=40$)	Obicetrapib 5 mg ($n=40$)	Obicetrapib 10 mg ($n=40$)
Musculoskeletal and connective tissue disorders	4 (10.0)	4 (10.0)	4 (10.0)
Muscle spasms	2 (5.0)	0 (0.0)	0 (0.0)
General disorders and administration site conditions	4 (10.0)	3 (7.5)	2 (5.0)
Fatigue	2 (5.0)	2 (5.0)	1 (2.5)
Gastrointestinal disorders	4 (10.0)	3 (7.5)	0 (0.0)
Nausea	2 (5.0)	1 (2.5)	0 (0.0)
Nervous system disorders	3 (7.5)	2 (5.0)	2 (5.0)
Investigations	3 (7.5)	2 (5.0)	0 (0.0)
Respiratory, thoracic and mediastinal disorders	3 (7.5)	2 (5.0)	0 (0.0)
Infections and infestations	3 (7.5)	1 (2.5)	0 (0.0)
Injury, poisoning and procedural complications	1 (2.5)	3 (7.5)	1 (2.5)
Metabolism and nutrition disorders	0 (0.0)	2 (5.0)	0 (0.0)
Type 2 diabetes mellitus	0 (0.0)	2 (5.0)	0 (0.0)
Neoplasms benign, malignant and unspecified[b]	2 (5.0)	0 (0.0)	0 (0.0)
Basal-cell carcinoma	2 (5.0)	0 (0.0)	0 (0.0)
Vascular disorders	0 (0.0)	2 (5.0)	0 (0.0)
Hypertension	0 (0.0)	2 (5.0)	0 (0.0)

Data are presented as *n* (%). [a]'Preferred term' is a level in the Medical Dictionary for Regulatory Activities hierarchy; it is defined as a distinct descriptor (single medical concept) for a symptom, sign, disease diagnosis, therapeutic indication, investigation, surgical or medical procedure and medical social or family history characteristic. [b]Including cysts and polyps.

Previous turnover studies with stable isotopes have suggested that the Apo-B- and LDL-C-lowering effect of CETP inhibitors is the consequence of increased clearance of Apo-B-containing lipoproteins, whereas the Lp(a) lowering effect follows from a decreased synthesis of Apo(a)[20,21].

Cardiovascular outcomes trials of CETP inhibitors, with the exception of the Randomized Evaluation of the Effects of Anacetrapib through Lipid Modification (REVEAL) trial, failed to demonstrate clinical benefit. It is now understood that those failures were compound specific, not drug-class specific[9]. Dalcetrapib sub-optimally reduced LDL-C, torcetrapib had off-target adverse effects, and evacetrapib had an insufficient duration of follow up for the modest absolute LDL-C difference to have sufficient impact on cardiovascular events[6,9,16,22,23]. In contrast, the REVEAL trial of 30,449 patients with ASCVD showed that anacetrapib added to statin therapy reduced LDL-C (direct measurement method) by 41% relative to placebo at 2 years, and significantly reduced risk of a first major coronary event (rate ratio 0.91, 95% confidence interval (CI) 0.85, 0.97) during the median follow up of 4.1 years[9,24]. In a subgroup of 2,000 patients in REVEAL, LDL-C was also analyzed using the PUC method. Results showed that the relative LDL-C reduction versus placebo was in fact substantially less than that measured by the direct assay, 17% versus 41%, which was compatible with the relative Apo B difference of 18%. Direct enzymatic assays, as well as the Friedewald formula, tend to underestimate true LDL-C concentrations only when LDL-C is very low. This can lead to an overestimation of the percentage change from baseline LDL-C[17,25]. The ROSE trial included a comparison of LDL-C results obtained using the Friedewald equation with PUC and also with the Martin–Hopkins equation. LDL-C values were comparable with each method, that is, within ~3–4 mg dl^{-1} of each other in the range of LDL-C levels observed in ROSE, which is an important finding divergent from preconceptions.

A multiple, ascending dose, phase 1 study of obicetrapib 1 mg, 2.5 mg, 5 mg, 10 mg and 25 mg, showed near-total inhibition of CETP with the 25 mg dose (99.4%) with differentiated and less inhibition between the 2.5 mg, 5 mg and 10 mg doses[13]. CETP activity was inhibited by 90.9% and 97.6%, respectively, for the 5 mg and 10 mg doses. The increase in CETP inhibition between obicetrapib 5 mg and 10 mg resulted in greater changes from baseline for LDL-C, non-HDL-C, Apo B, HDL-C and Lp(a). The 5 mg dose versus the 10 mg dose reduced Apo B by 24.4% versus 29.75%, respectively, increased HDL-C by 135.4% versus 165%, respectively and, finally, reduced Lp(a) by 33.8% versus 56.5%, respectively.

There is a marked difference in the CETP inhibitory potential of obicetrapib compared to anacetrapib and evacetrapib. At equipotent dosages obicetrapib reduces CETP activity to a greater extent than both anacetrapib and evacetrapib[13,26–28]. This is also the case for the phase 3 dose of obicetrapib (10 mg) compared with that of anacetrapib (100 mg). There are no data available on CETP inhibition for the phase 3 dose of evacetrapib (130 mg); however, results from the phase 1 multiple-ascending dose study of 100 mg evacetrapib indicate that it is significantly less potent than obicetrapib. These differences in the capacity to inhibit CETP also translate into the ability to raise HDL-C; obicetrapib 10 mg raises HDL-C by 169.9% compared with placebo, while 100 mg anacetrapib and 130 mg evaceptrapib raised HDL-C levels by 104% and 131.6%, respectively[16,24].

A potential explanation for the potency of obicetrapib comes from a series of crystallography experiments showing that CETP inhibitors locate at the narrow *N*-terminal neck of the hydrophobic tunnel of CETP and can restrict the lipid flow through this tunnel[29]. Whereas these interactions between the CETP inhibitor and the opening of the tunnel are of a hydrophobic nature, there are also three polar residues in the center of the inhibitor-binding site, Gln-199, Ser-230 and His-232. The authors conclude that it might be possible to incorporate more hydrophilic groups into the structure of a CETP inhibitor that can then interact with these three polar amino acids to improve the binding and specificity of the compound while also improving the solubility of the CETP inhibitor. Indeed, by introducing hydrophilic structures into obicetrapib, it is the most polar of all CETP inhibitors and has a LogP of 4.9 versus 9.2 for anacetrapib and 7.9 for evacetrapib[30]. It also has better bioavailability and greater efficacy at lower doses. These biochemical differences between CETP inhibitors likely partly explain the large differences in potency.

A limitation of this trial includes the relatively homogeneous racial/ethnic composition of the population evaluated, making it difficult to generalize the results to the wider population. In this trial, obicetrapib therapy of 5 mg and 10 mg did not result in clinically important changes in laboratory parameters or vital signs but had an acceptable safety profile, confirming previous safety and tolerability findings[14]. Although a recent Mendelian randomization study claimed that CETP inhibition may 'cause' an increase in cases of age-related macular degeneration[31], those conclusions were contrary to available clinical evidence to date, including the results from the REVEAL trial of anacetrapib[24]. Analyses of obicetrapib blood concentrations demonstrated that steady-state conditions were already achieved by 4 weeks and decreased substantially after the end of treatment. As a result, unlike anacetrapib, which demonstrated accumulation in adipose tissue, the terminal half-life of obicetrapib was adequate for once-a-day chronic dosing.

In conclusion, this study demonstrated the efficacy of obicetrapib 5 mg and 10 mg as an adjunct to high-intensity statins for

robustly decreasing LDL-C, Apo B, non-HDL-C and Lp(a) and increasing HDL-C and Apo A1, compared with placebo, thus refuting the theory that such CETP-inhibitor-driven effects would be attenuated when used in combination with high-intensity statins. This trial also expanded the growing body of evidence to support the safety and tolerability of this specific CETP inhibitor[13,14]. In light of the unmet medical need for additional therapies to substantially reduce LDL-C in patients at high cardiovascular risk, the results from ROSE are promising. Additional phase 3 investigations including a cardiovascular outcomes trial are currently underway to further assess the safety and clinical benefits of obicetrapib.

Online content

Any methods, additional references, Nature Research reporting summaries, source data, extended data, supplementary information, acknowledgements, peer review information; details of author contributions and competing interests; and statements of data and code availability are available at https://doi.org/10.1038/s41591-022-01936-7.

Received: 19 December 2021; Accepted: 6 July 2022;
Published online: 11 August 2022

References

1. Arnett, D. K. et al. 2019 ACC/AHA guideline on the primary prevention of cardiovascular disease: a report of the American College of Cardiology/American Heart Association Task Force on Clinical Practice Guidelines. *Circulation* **140**, e596–e646 (2019).
2. Mach, F. et al. ESC Scientific Document Group. 2019 ESC/EAS guidelines for the management of dyslipidaemias: lipid modification to reduce cardiovascular risk. *Eur. Heart J.* **41**, 111–188 (2020).
3. Packard, C., Chapman, M. J., Sibartie, M., Laufs, U. & Masana, L. Intensive low-density lipoprotein cholesterol lowering in cardiovascular disease prevention: opportunities and challenges. *Heart* **107**, 1369–1375 (2021).
4. Cannon, C. P. et al. for the GOULD Investigators. Use of lipid-lowering therapies over 2 years in GOULD, a registry of patients with atherosclerotic cardiovascular disease in the US. *JAMA Cardiol.* **6**, 1060–1068 (2021).
5. Ray, K. K. et al. EU-wide cross-sectional observational study of lipid-modifying therapy use in secondary and primary care: the DA VINCI study. *Eur. J. Prev. Cardiol.* **28**, 1279–1289 (2021).
6. Tall, A. R. & Rader, D. J. Trials and tribulations of CETP inhibitors. *Circ. Res.* **122**, 106–112 (2018).
7. Shrestha, S., Wu, B. J., Guiney, L., Barter, P. J. & Rye, K. A. Cholesteryl ester transfer protein and its inhibitors. *J. Lipid Res.* **59**, 772–783 (2018).
8. Banderjee, S. & De, A. Pathophysiology and inhibition of cholesteryl ester transfer protein for prevention of cardiovascular diseases: an update. *Drug Discov. Today* **26**, 1759–1764 (2021).
9. Schmidt, A. F. et al. Cholesteryl ester transfer protein (CETP) as a drug target for cardiovascular disease. *Nat. Commun.* **12**, 5640 (2021).
10. Voight, B. F. et al. Plasma HDL cholesterol and risk of myocardial infarction: a Mendelian randomization study. *Lancet* **380**, 572–580 (2012).
11. Van Capelleveen, J. C. et al. Effects of the cholesteryl ester transfer protein inhibitor, TA-8995, on cholesterol efflux capacity and high-density lipoprotein particle subclasses. *J. Clin. Lipidol.* **10**, 1137–1144.e3 (2016).
12. Ference, B. A. et al. Association of genetic variants related to CETP inhibitors and statins with lipoprotein levels and cardiovascular risk. *JAMA* **318**, 947–956 (2017).
13. Ford, J. et al. Tolerability, pharmacokinetics and pharmacodynamics of TA-8995, a selective cholesteryl ester transfer protein (CETP) inhibitor, in healthy subjects. *Br. J. Clin. Pharmacol.* **78**, 498–508 (2014).
14. Hovingh, G. K. et al. Cholesterol ester transfer protein inhibition by TA-8995 in patients with mild dyslipidaemia (TULIP): a randomised, double-blind, placebo-controlled phase 2 trial. *Lancet* **386**, 452–460 (2015).
15. Nurmohamed, N. S., Navar, A. M. & Kastelein, J. J. P. New and emerging therapies for reduction of LDL-cholesterol and apolipoprotein B: JACC Focus Seminar 1/4. *J. Am. Coll. Cardiol.* **77**, 1564–1575 (2021).
16. Lincoff, A. M. et al., for the ACCELERATE Investigators. Evacetrapib and cardiovascular outcomes in high-risk vascular disease. *N. Engl. J. Med.* **376**, 1933–1942 (2017).
17. Davidson, M. et al. Measurement of LDL-C after treatment with CETP inhibitor anacetrapib. *J. Lipid Res.* **54**, 467–472 (2013).
18. Martin, S. S. et al. Comparison of a novel method vs. the Friedewald equation for estimating low-density lipoprotein cholesterol levels from the standard lipid profile. *JAMA* **310**, 2061–2068 (2013).
19. Ference, B. A. et al. Low-density lipoproteins cause atherosclerotic cardiovascular disease. 1. Evidence from genetic, epidemiologic, and clinical studies. A consensus statement from the European Atherosclerosis Society Consensus Panel. *Eur. Heart J.* **38**, 2459–2472 (2017).
20. Millar, J. S. et al. Anacetrapib lowers LDL by increasing ApoB clearance in mildly hypercholesterolemic subjects. *J. Clin. Invest.* **126**, 1603–1604 (2016).
21. Thomas, T. et al. CETP (cholesteryl ester transfer protein) inhibition with anacetrapib decreases production of lipoprotein(a) in mildly hypercholesterolemic subjects. *Arterioscler. Thromb. Vasc. Biol.* **37**, 1770–1775 (2017).
22. Barter, P. J. et al. for the ILLUMINATE Investigators. Effects of torcetrapib in patients at high risk for coronary events. *N. Engl. J. Med.* **357**, 2109–2122 (2007).
23. Schwartz, G. C. et al. Effects of dalcetrapib in patients with acute coronary syndrome. *N. Engl. J. Med.* **367**, 2089–2099 (2012).
24. HPS3-TIMI55-REVEAL Collaborative Group. Effects of anacetrapib in patients with atherosclerotic vascular disease. *N. Engl. J. Med.* **377**, 1217–1227 (2017).
25. Holmes, M. V. & Ala-Korpela, M. What is 'LDL cholesterol'? *Nat. Rev. Cardiol.* **16**, 197–198 (2019).
26. Krishna, R. et al. Multiple-dose pharmacodynamics and pharmacokinetics of anacetrapib, a potent cholesteryl ester transfer protein (CETP) inhibitor, in healthy subjects. *Clin. Pharmacol. Ther.* **84**, 679–683 (2008).
27. Krishna, R. et al. Single-dose pharmacokinetics and pharmacodynamics of anacetrapib, a potent cholesteryl ester transfer protein (CETP) inhibitor, in healthy subjects. *Br. J. Clin. Pharmacol.* **68**, 535–545 (2009).
28. Suico, J. G. et al. Effects of the cholesteryl ester transfer protein inhibitor evacetrapib on lipoproteins, apolipoproteins and 24-h ambulatory blood pressure in healthy adults. *J. Pharm. Pharmacol.* **66**, 1576–1585 (2014).
29. Liu, S. et al. Crystal structures of cholesteryl ester transfer protein in complex with inhibitors. *J. Am. Coll. Cardiol.* **287**, 37321–37329 (2012).
30. Nurmohamed, N.S. et al. Cholesteryl ester transfer protein inhibitors: from high-density lipoprotein cholesterol to low-density lipoprotein cholesterol lowering agents? *Cardiovasc. Res.* https://doi.org/10.1093/cvr/cvab350 (2021).
31. Nordestgaard, L. T. et al. Long-term benefits and harms associated with genetic cholesteryl ester transfer protein deficiency in the general population. *JAMA Cardiol.* **7**, 55–64 (2022).

Publisher's note Springer Nature remains neutral with regard to jurisdictional claims in published maps and institutional affiliations.

Springer Nature or its licensor holds exclusive rights to this article under a publishing agreement with the author(s) or other rightsholder(s); author self-archiving of the accepted manuscript version of this article is solely governed by the terms of such publishing agreement and applicable law.

© The Author(s), under exclusive licence to Springer Nature America, Inc. 2022

Methods

Study design. This placebo-controlled, double-blind, randomized, phase 2 dose-finding study of obicetrapib was conducted from February 2021 to August 2021 (6 months) at 17 clinical research sites in the United States (see list of Principal Investigators in the Supplementary Note 1) in accordance with Good Clinical Practice guidelines, the Declaration of Helsinki (2000) and the US 21 Code of Federal Regulations. The trial was registered at ClinicalTrials.gov with the identifier NCT04753606. The protocol was approved by Advarra Institutional Review Board (Columbia, Maryland). The protocol and the statistical analysis plan are provided in Supplementary Note 2. All subjects provided informed consent before their enrollment in the study. There were seven clinic visits during the trial: a screening visit where subjects provided informed consent, a randomization visit up to 2 weeks after screening where subject eligibility was confirmed and subjects were randomized to treatment, visits at the midpoint (week 4) and end of treatment (week 8), a safety and PK follow-up visit 4 weeks after the conclusion of treatment, and additional PK follow-up visits at 8 and 15 weeks after the conclusion of treatment.

Subjects were randomized, using an automated interactive response technology system, in a 1:1:1 ratio to the following treatment groups: 5 mg obicetrapib (one 5 mg obicetrapib tablet plus one placebo tablet), 10 mg obicetrapib (two 5 mg obicetrapib tablets) or placebo (two placebo tablets). To ensure similar distribution of LDL-C values across all treatment groups, subjects were stratified at randomization according to their screening LDL-C level of $\geq$100 or <100 mg dl^{-1}. Investigators, participants, the clinical research organization and the sponsor of the trial were blinded to all lipid results from randomization through database lock. Obicetrapib and placebo tablets were provided to the clinical research sites by New Amsterdam Pharma (Naarden, the Netherlands). Subjects were instructed to take the assigned study drug once daily throughout the 8-week treatment period at approximately the same time each morning, with food. On days when clinic visits were scheduled, study drugs were administered with food following the collection of the fasted blood samples. Participants were instructed to bring all their study-drug supply to the site at the clinic visits, where compliance was evaluated by counting unused tablets.

Subjects. Subjects included men and women 18–75 years of age with fasting LDL-C > 70 mg dl^{-1} and TG < 400 mg dl^{-1} at screening, while receiving high-intensity statin therapy (atorvastatin 40 mg or 80 mg or rosuvastatin 20 mg or 40 mg) at a stable dose for at least the prior 8 weeks. Women enrolled were not pregnant or breastfeeding, and, if of childbearing potential, had a negative urine pregnancy test and agreed to use an effective method of avoiding pregnancy throughout the trial. Volunteers were excluded from participation if they had a BMI $\geq$ 40 kg m^{-2}, clinically significant cardiovascular disease, glycated hemoglobin $\geq$10%, uncontrolled hypertension, active muscle disease or creatine kinase thrice the upper limit of normal, estimated glomerular filtration rate <60 ml min^{-1} (ref. [32]). hepatic dysfunction, anemia or a recent history of malignancy or alcohol or drug abuse. Individuals who had been treated with a PCSK9 inhibitor within 10 weeks prior to randomization or bempedoic acid within 2 weeks prior to randomization were also excluded.

Assessments. Blood samples were obtained under fasting conditions (~10 h, nothing by mouth except water and any essential medications) at each clinic visit throughout the trial. At screening, baseline, and 4 and 8 weeks of treatment, fasting samples were analyzed for a lipid profile by Medpace Reference Laboratory, Cincinnati, Ohio, using Beckman Coulter AU5800. For the primary analysis, LDL-C was calculated using the Friedewald calculation (total cholesterol minus HDL-C minus (TG/5)) at each time point, unless TG $\geq$ 400 mg dl^{-1} or LDL-C $\leq$ 50 mg dl^{-1}, in which case LDL-C was measured directly by preparative ultracentrifugation[33]. LDL-C was also measured by PUC at baseline and at the end of treatment in all subjects (Beckman Coulter AU5800), and was calculated using the Martin–Hopkins equation[18], a modified version of the Friedewald equation that uses a novel factor based on the patient's individual non-HDL-C and TG values, instead of the fixed ratio of TG/5. Additional components measured in the fasting lipid profile included HDL-C, non-HDL-C (total cholesterol minus HDL-C), VLDL-C, TG, Apo B, Apo A1 and Lp(a). Apo B and Apo A1 were measured using a Siemens nephelometric analyser. Lp(a) was measured using Roche c502. Fasting blood samples were analyzed at each clinic visit for safety chemistry (including glycated hemoglobin at screening) and hematology profiles. At randomization, a blood sample was collected pre-dose for PK analyses, and subsequent post-dose PK samples were collected once at approximately the same time at each remaining clinic visit for measurement of plasma obicetrapib concentrations. Chemical analyses of obicetrapib were performed by liquid chromatography (Shimadzu LC-30 AD)/mass spectrometry (AB Sciex Triple Quad 5500 System).

AEs were monitored from the first dose of study treatment through the completion of the trial. Vital signs, including body temperature, heart rate and triplicate systolic and diastolic blood pressure measurements were taken from screening through the safety follow-up visits, with participants in the supine position after at least 10 min rest prior to measurements. Body weight and height were measured at screening and used to calculate BMI. A single-standard 12-lead electrocardiogram was performed at screening, and a physical examination was performed at screening and the conclusion of the treatment period.

Statistics. A sample size of at least 108 evaluable participants (36 per treatment group) was determined to provide more than 90% power to detect a 30% difference in LDL-C reduction at week 8 (standard deviation of 15%) for each of the obicetrapib groups compared with placebo at a two-sided significance level of 0.025. A total of 120 subjects (n = 40 per treatment group) was randomized.

All statistical analyses were performed using SAS v.9.4 (SAS Institute, Cary, NC). The mITT population, defined as all participants in the ITT population who received at least one dose of any study drug and had a baseline value for the LDL-C assessment, was the primary population for the efficacy analyses. Efficacy was also analyzed in the ITT (all participants randomized) and PP populations (all participants in the mITT population who had a baseline value for the LDL-C assessment, had a week 8 LDL-C assessment, and did not experience a major protocol deviation with the potential to impact the primary efficacy endpoint) as supportive analyses. Safety analyses were performed in the safety population defined as all participants who received at least one dose of any study drug. PK analyses were performed in the PK population defined as all participants in the mITT population who had sufficient blood samples collected for valid estimation of PK parameters.

The primary efficacy analysis, percent change in Friedewald-calculated LDL-C from baseline to week 8, was performed using an MMRM approach, with SAS Proc Mixed. The analysis included fixed effects for treatment, visit and treatment-by-visit interaction, along with a covariate of the baseline value as a continuous covariate. The restricted maximum likelihood estimation approach was used with an unstructured covariance matrix. LS means, s.e. and two-sided 95% CIs for each treatment group and for the pairwise comparisons of each dose of obicetrapib with the placebo group were determined. Adjustment for multiple comparisons was made using Dunnett's test. No imputation of missing data was performed for the primary efficacy endpoint analysis. The Shapiro–Wilk test was used to evaluate normality. Three sensitivity analyses were also performed for the primary efficacy endpoint: MMRM with imputation, ANCOVA and ANCOVA using LDL-C by PUC. Similar MMRM models as described for the primary efficacy analyses were used to analyze the other efficacy endpoints (that is, percent change from baseline to week 8 in Apo B, non-HDL-C, HDL-C, Apo A1, TG, VLDL-C and Lp(a)). No adjustment was made for multiplicity in testing these other endpoints, and no imputation was made.

Reporting summary. Further information on research design is available in the Nature Research Reporting Summary linked to this article.

Data availability

The authors declare that all data supporting the findings of this analysis, collected by MedPace ClinTrack, are available within the article and its supplement. New Amsterdam Pharma is committed to sharing, with qualified external researchers, access to patient-level data and supporting clinical documents from eligible studies. These requests are reviewed and approved by an independent review and panel on the basis of scientific merit. All data provided are anonymized to respect the privacy of patients who have participated in the trial, in line with applicable laws and regulations.

References

32. CKD-EPI equations for glomerular filtration rate (GFR). *MDCalc* https://www.mdcalc.com/ckd-epi-equations-glomerular-filtration-rate-gfr (2020).
33. LDL calculated. *MDCalc* https://www.mdcalc.com/ldl-calculated (2020).

Acknowledgements

This trial was funded by New Amsterdam Pharma. We thank the contributions of the study patients, the dedicated study physicians and the New Amsterdam Pharma operations team.

Author contributions

The study was conceived by M.D, J.J.K., D.K. and M.H.D. S.P.R. and N.J.A. analyzed the data. M.D. wrote the first draft of the manuscript. All authors interpreted the results, contributed to writing and approved the final version for submission.

Competing interests

S.J.N. has received research support from AstraZeneca, New Amsterdam Pharma, Amgen, Anthera, Eli Lilly, Esperion, Novartis, Cerenis, The Medicines Company, Resverlogix, IntraReDx, Roche, Sanofi-Regeneron and LipoScience and is a consultant for AstraZeneca, Amarin, Akcea, Eli Lilly, Anthera, Omthera, Merck, Takeda, Resverlogix, Sanofi-Regeneron, CSL Behring, Esperion and Boehringer Ingelheim. M.D. is Vice President of Research & Development for New Amsterdam Pharma. J.J.K. is employed part-time as Chief Scientific Officer by New Amsterdam Pharma and is an Emeritus Professor of Medicine at the University of Amsterdam, The Netherlands. S.P.R. is employed by MedPace, Inc. D.K. is employed as Chief Operating Officer by New Amsterdam Pharma. D.L.C. is employed as Senior Director of Clinical Operations by

New Amsterdam Pharma. N.J.A. is employed by MedPace, Inc. M.H.D. is employed as Chief Executive Officer by New Amsterdam Pharma.

Additional information

Supplementary information The online version contains supplementary material available at https://doi.org/10.1038/s41591-022-01936-7.

Correspondence and requests for materials should be addressed to Stephen J. Nicholls.

Peer review information *Nature Medicine* thanks Gerald Watts and the other, anonymous, reviewer(s) for their contribution to the peer review of this work. Primary Handling Editor: Michael Basson, in collaboration with the *Nature Medicine* team.

Reprints and permissions information is available at www.nature.com/reprints.

nature portfolio

Corresponding author(s): Stephen J Nicholls

Last updated by author(s): Mar 30, 2022

Reporting Summary

Nature Portfolio wishes to improve the reproducibility of the work that we publish. This form provides structure for consistency and transparency in reporting. For further information on Nature Portfolio policies, see our Editorial Policies and the Editorial Policy Checklist.

Statistics

For all statistical analyses, confirm that the following items are present in the figure legend, table legend, main text, or Methods section.

n/a	Confirmed	
☐	☒	The exact sample size (*n*) for each experimental group/condition, given as a discrete number and unit of measurement
☐	☒	A statement on whether measurements were taken from distinct samples or whether the same sample was measured repeatedly
☐	☒	The statistical test(s) used AND whether they are one- or two-sided<br>*Only common tests should be described solely by name; describe more complex techniques in the Methods section.*
☐	☒	A description of all covariates tested
☐	☒	A description of any assumptions or corrections, such as tests of normality and adjustment for multiple comparisons
☐	☒	A full description of the statistical parameters including central tendency (e.g. means) or other basic estimates (e.g. regression coefficient) AND variation (e.g. standard deviation) or associated estimates of uncertainty (e.g. confidence intervals)
☒	☐	For null hypothesis testing, the test statistic (e.g. *F*, *t*, *r*) with confidence intervals, effect sizes, degrees of freedom and *P* value noted<br>*Give P values as exact values whenever suitable.*
☒	☐	For Bayesian analysis, information on the choice of priors and Markov chain Monte Carlo settings
☒	☐	For hierarchical and complex designs, identification of the appropriate level for tests and full reporting of outcomes
☒	☐	Estimates of effect sizes (e.g. Cohen's *d*, Pearson's *r*), indicating how they were calculated

Our web collection on statistics for biologists contains articles on many of the points above.

Software and code

Policy information about availability of computer code

Data collection	Medpace ClinTrack systems were used for data collection.
Data analysis	Statistical analyses were performed using SAS version 9.4 (SAS Institute Cary, NC).

For manuscripts utilizing custom algorithms or software that are central to the research but not yet described in published literature, software must be made available to editors and reviewers. We strongly encourage code deposition in a community repository (e.g. GitHub). See the Nature Portfolio guidelines for submitting code & software for further information.

Data

Policy information about availability of data

All manuscripts must include a data availability statement. This statement should provide the following information, where applicable:

- Accession codes, unique identifiers, or web links for publicly available datasets
- A description of any restrictions on data availability
- For clinical datasets or third party data, please ensure that the statement adheres to our policy

The authors declare that all data supporting the findings of this analysis are available within the article and its supplement. New Amsterdam Pharma is committed to sharing, with qualified external researchers, access to patient-level data and supporting clinical documents from eligible studies. These requests are reviewed and approved by an independent review panel on the basis of scientific merit. All data provided are anonymized to respect the privacy of patients who have participated in the trial, in line with applicable laws and regulations.

Field-specific reporting

Please select the one below that is the best fit for your research. If you are not sure, read the appropriate sections before making your selection.

☒ Life sciences ☐ Behavioural & social sciences ☐ Ecological, evolutionary & environmental sciences

For a reference copy of the document with all sections, see nature.com/documents/nr-reporting-summary-flat.pdf

Life sciences study design

All studies must disclose on these points even when the disclosure is negative.

Sample size	A sample size of at least 108 evaluable participants (36 per treatment group) was determined to provide more than 90% power to detect a 30% difference in LDL-C reduction at week 8 (standard deviation of 15%) for each of the obicetrapib groups compared to placebo at a 2-sided significance level of 0.025. A total of 120 subjects (n = 40 per treatment group) was randomized.
Data exclusions	The pre-defined analyses populations were an intent-to-treat (ITT) population (all participants randomized), a modified ITT population, defined as all participants in the ITT population who received at least 1 dose of any study drug and had a baseline baseline for LDL-C assessment (which was primary), and a per protocol population which included all participants in the modified ITT population who had a baseline LDL-C value, a week 8 LDL-C value, and did not experience a major protocol deviation with the potential to impact the primary efficacy endpoint.
Replication	The results from this trial are consistent with those from a previously completed trial of obicetrapib, administered alone and in combination with statin therapy, in patients with mild dyslipidemia (TULIP; NCT01970215).
Randomization	This was a randomized, controlled trial. Subjects were randomized using an automated interactive response technology system in a 1:1:1 ratio to one of three treatment groups.
Blinding	Investigators, participants, the clinical research organization, and the sponsor of the trial were all blinded to all lipid results from randomization through database lock.

Reporting for specific materials, systems and methods

We require information from authors about some types of materials, experimental systems and methods used in many studies. Here, indicate whether each material, system or method listed is relevant to your study. If you are not sure if a list item applies to your research, read the appropriate section before selecting a response.

Materials & experimental systems

n/a	Involved in the study	
☒	☐	Antibodies
☒	☐	Eukaryotic cell lines
☒	☐	Palaeontology and archaeology
☒	☐	Animals and other organisms
☐	☒	Human research participants
☐	☒	Clinical data
☒	☐	Dual use research of concern

Methods

n/a	Involved in the study	
☒	☐	ChIP-seq
☒	☐	Flow cytometry
☒	☐	MRI-based neuroimaging

Human research participants

Policy information about studies involving human research participants

Population characteristics	Subjects included men and women 18-75 years of age with fasting LDL-C >70 mg/dL and TG <400 mg/dL at screening, while receiving high-intensity statin therapy (atorvastatin 40 mg or 80 mg or rosuvastatin 20 mg or 40 mg) at a stable dose for at least the prior 8 weeks.
Recruitment	Participants were recruited, according to the entry criteria outlined in the protocol, through advertisements or review of clinical records at individual sites across the United States. The study was designed as a placebo-controlled, double-blind, randomized trial to reduce bias.
Ethics oversight	The protocol was approved by Advarra Institutional Review Board (Columbia, Maryland, USA).

Note that full information on the approval of the study protocol must also be provided in the manuscript.

Clinical data

Policy information about clinical studies

All manuscripts should comply with the ICMJE guidelines for publication of clinical research and a completed CONSORT checklist must be included with all submissions.

Clinical trial registration	NCT04753606
Study protocol	The protocol and statistical analysis plan for the study have been provided to the journal.
Data collection	The study was conducted from February 2021 to August 2021 (6 months) at 17 clinical research sites in the United States.
Outcomes	The primary efficacy endpoint was the percent change from baseline to week 8 in LDL-C for each obicetrapib group compared to the placebo group. The key secondary efficacy endpoints included the percent changes from baseline to week 8 in Apo B, non-HDL-C, and HDL-C for each obicetrapib group compared to the placebo group.
