# Supplementary figures and images for: Lipid-lowering effect of combined therapy with high-intensity statins and CETP inhibitors: a Systematic Review and meta-analysis
Source: Front Endocrinol (Lausanne). 2025 May 1;16:1512670. doi: 10.3389/fendo.2025.1512670 (PMC12078159; doi:10.3389/fendo.2025.1512670)

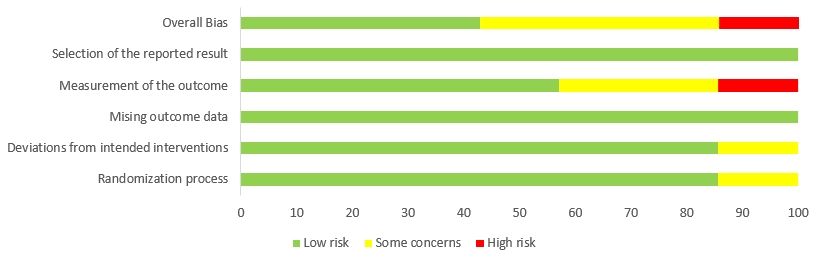

Supplement: Supplementary file 1 [file DataSheet1.zip › Raw Data/Raw Data/5. Risk of bias/1.jpg]

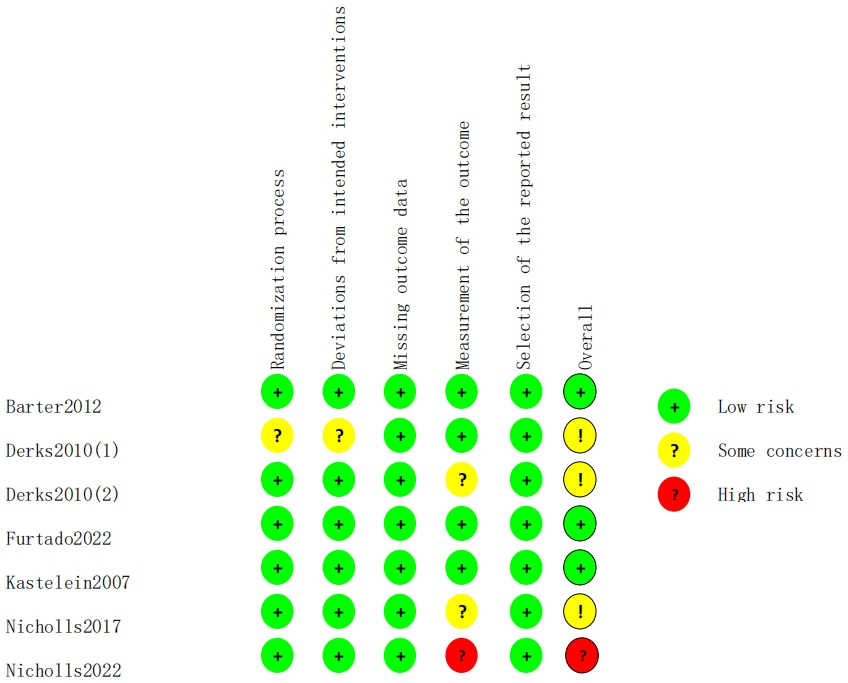

Supplement: Supplementary file 1 [file DataSheet1.zip › Raw Data/Raw Data/5. Risk of bias/2.jpg]

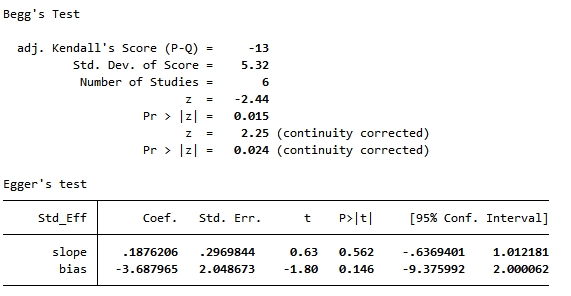

Supplement: Supplementary file 1 [file DataSheet1.zip › Raw Data/Raw Data/3. Publication bias/TG.jpg]

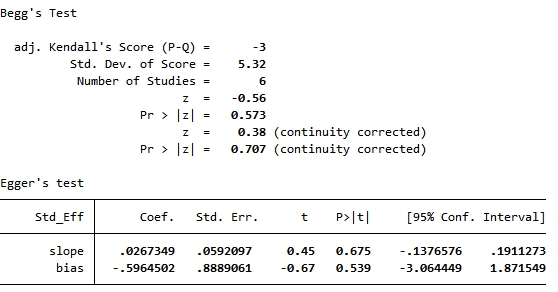

Supplement: Supplementary file 1 [file DataSheet1.zip › Raw Data/Raw Data/3. Publication bias/AE.jpg]

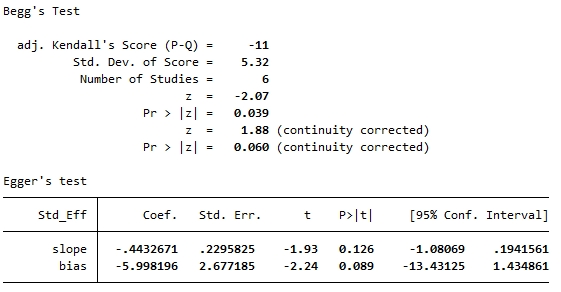

Supplement: Supplementary file 1 [file DataSheet1.zip › Raw Data/Raw Data/3. Publication bias/ApoB.png]

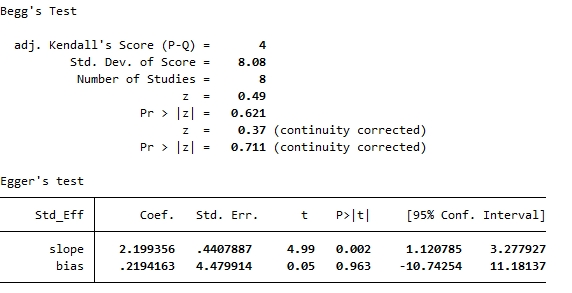

Supplement: Supplementary file 1 [file DataSheet1.zip › Raw Data/Raw Data/3. Publication bias/HDL-C.png]

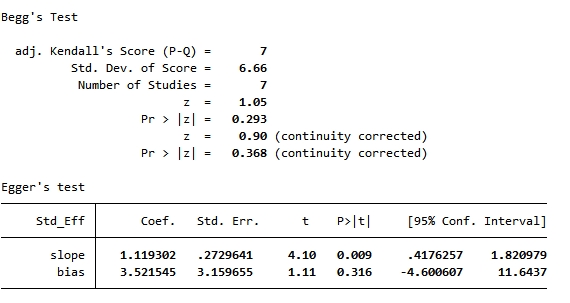

Supplement: Supplementary file 1 [file DataSheet1.zip › Raw Data/Raw Data/3. Publication bias/ApoAI.jpg]

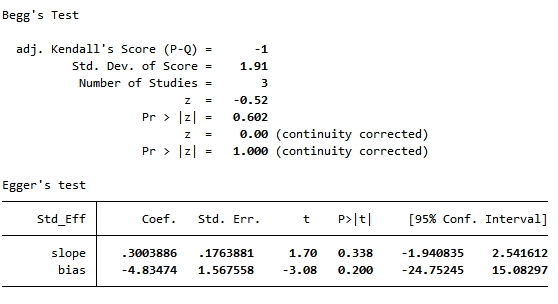

Supplement: Supplementary file 1 [file DataSheet1.zip › Raw Data/Raw Data/3. Publication bias/TC.jpg]

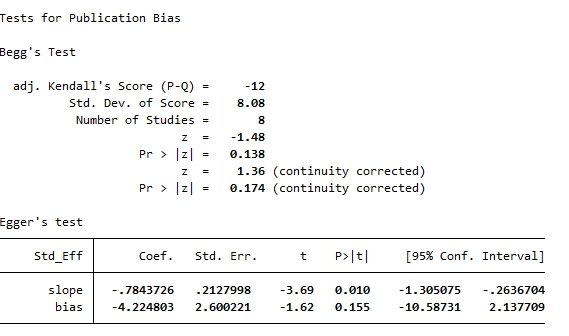

Supplement: Supplementary file 1 [file DataSheet1.zip › Raw Data/Raw Data/3. Publication bias/LDL.png]
