# Supplementary material for: Lipid-lowering effect of combined therapy with high-intensity statins and CETP inhibitors: a Systematic Review and meta-analysis
Source: Front Endocrinol (Lausanne). 2025 May 1;16:1512670. doi: 10.3389/fendo.2025.1512670 (PMC12078159; doi:10.3389/fendo.2025.1512670)
Supplement: Supplementary file 2 [file Table1.docx]

**Supplementary Materials 1:** Search strategy

**PubMed**

| Search number | Query | Results |
| --- | --- | --- |
| 1 | "Hydroxymethylglutaryl-CoA Reductase Inhibitors"[Mesh] | 35,624 |
| 2 | ((((((((((((((("Hydroxymethylglutaryl-CoA Reductase Inhibitors"[Mesh]) OR (Hydroxymethylglutaryl-CoA Reductase Inhibitors[Title/Abstract])) OR (Hydroxymethylglutaryl CoA Reductase Inhibitors[Title/Abstract])) OR (HMG-CoA Reductase Inhibitor[Title/Abstract])) OR (HMG CoA Reductase Inhibitor[Title/Abstract])) OR (Statin[Title/Abstract])) OR (HMG-CoA Statins[Title/Abstract])) OR (HMG-CoA Reductase Inhibitors[Title/Abstract])) OR (Hydroxymethylglutaryl-Coenzyme A Inhibitors[Title/Abstract])) OR (Hydroxymethylglutaryl-CoA Inhibitors[Title/Abstract])) OR (Hydroxymethylglutaryl-CoA Reductase Inhibitor[Title/Abstract])) OR (Hydroxymethylglutaryl CoA Reductase Inhibitor[Title/Abstract])) OR (hydroxymethylglutaryl coenzyme A reductase inhibitor[Title/Abstract])) OR (HMG CoA reductase inhibitor[Title/Abstract])) OR (hmg coenzyme a reductase inhibitor[Title/Abstract])) OR (statin (drug[Title/Abstract])) | 52,989 |
| 3 | "Cholesterol Ester Transfer Proteins"[Mesh] | 2,811 |
| 4 | (((((("Cholesterol Ester Transfer Proteins"[Mesh]) OR (Cholesterol Ester Transfer Proteins[Title/Abstract])) OR (Cholesterol Ester Transport Protein, CETP[Title/Abstract])) OR (Cholesteryl Ester Transfer Protein[Title/Abstract])) OR (Cholesterol Ester Transfer Protein[Title/Abstract])) OR (Cholesteryl Ester Exchange Protein[Title/Abstract])) OR (CETP[Title/Abstract]) | 4,300 |
| 5 | (((((((((((((((("Hydroxymethylglutaryl-CoA Reductase Inhibitors"[Mesh]) OR (Hydroxymethylglutaryl-CoA Reductase Inhibitors[Title/Abstract])) OR (Hydroxymethylglutaryl CoA Reductase Inhibitors[Title/Abstract])) OR (HMG-CoA Reductase Inhibitor[Title/Abstract])) OR (HMG CoA Reductase Inhibitor[Title/Abstract])) OR (Statin[Title/Abstract])) OR (HMG-CoA Statins[Title/Abstract])) OR (HMG-CoA Reductase Inhibitors[Title/Abstract])) OR (Hydroxymethylglutaryl-Coenzyme A Inhibitors[Title/Abstract])) OR (Hydroxymethylglutaryl-CoA Inhibitors[Title/Abstract])) OR (Hydroxymethylglutaryl-CoA Reductase Inhibitor[Title/Abstract])) OR (Hydroxymethylglutaryl CoA Reductase Inhibitor[Title/Abstract])) OR (hydroxymethylglutaryl coenzyme A reductase inhibitor[Title/Abstract])) OR (HMG CoA reductase inhibitor[Title/Abstract])) OR (hmg coenzyme a reductase inhibitor[Title/Abstract])) OR (statin (drug[Title/Abstract]))) AND ((((((("Cholesterol Ester Transfer Proteins"[Mesh]) OR (Cholesterol Ester Transfer Proteins[Title/Abstract])) OR (Cholesterol Ester Transport Protein, CETP[Title/Abstract])) OR (Cholesteryl Ester Transfer Protein[Title/Abstract])) OR (Cholesterol Ester Transfer Protein[Title/Abstract])) OR (Cholesteryl Ester Exchange Protein[Title/Abstract])) OR (CETP[Title/Abstract])) | 471 |

**Embase**

| No. | Query | Results | Date |
| --- | --- | --- | --- |
| #1 | 'hydroxymethylglutaryl coenzyme a reductase inhibitor'/exp | 199,984 | 11-May-24 |
| #2 | 'hydroxymethylglutaryl-coa reductase inhibitors'/exp OR 'hydroxymethylglutaryl-coa reductase inhibitors' OR ('hydroxymethylglutaryl coa' AND ('reductase'/exp OR reductase) AND ('inhibitors'/exp OR inhibitors)) OR 'hydroxymethylglutaryl coa reductase inhibitors':ab,ti OR 'hmg-coa reductase inhibitor':ab,ti OR statin:ab,ti OR 'hmg-coa statins':ab,ti OR 'hmg-coa reductase inhibitors':ab,ti OR 'hydroxymethylglutaryl-coenzyme a inhibitors':ab,ti OR 'hydroxymethylglutaryl-coa inhibitors':ab,ti OR 'hydroxymethylglutaryl-coa reductase inhibitor':ab,ti OR 'hydroxymethylglutaryl coa reductase inhibitor':ab,ti OR 'hydroxymethylglutaryl coenzyme a reductase inhibitor':ab,ti OR 'hmg coa reductase inhibitor':ab,ti OR 'hmg coenzyme a reductase inhibitor':ab,ti OR (statin:ab,ti AND drug:ab,ti) | 207,389 | 11-May-24 |
| #3 | #1 OR #2 | 207,389 | 11-May-24 |
| #4 | 'cholesterol ester transfer protein'/exp | 5,160 | 11-May-24 |
| #5 | 'cholesterol ester transfer proteins'/exp OR 'cholesterol ester transfer proteins' OR (('cholesterol'/exp OR cholesterol) AND ('ester'/exp OR ester) AND ('transfer'/exp OR transfer) AND ('proteins'/exp OR proteins)) OR 'cholesterol ester transport protein, cetp':ab,ti OR 'cholesteryl ester transfer protein':ab,ti OR 'cholesterol ester transfer protein':ab,ti OR 'cholesteryl ester exchange protein':ab,ti OR cetp:ab,ti | 6,672 | 11-May-24 |
| #6 | #4 OR #5 | 6,672 | 11-May-24 |
| #7 | #3 AND #6 | 1,440 | 11-May-24 |

**# Web of Science**

# Database: Web of Science Core Collection

# Entitlements:

- WOS.IC: 1993 to 2024

- WOS.CCR: 1985 to 2024

- WOS.SCI: 1975 to 2024

- WOS.AHCI: 1975 to 2024

- WOS.BHCI: 2005 to 2024

- WOS.BSCI: 2005 to 2024

- WOS.ESCI: 2019 to 2024

- WOS.ISTP: 1990 to 2024

- WOS.SSCI: 1965 to 2024

- WOS.ISSHP: 1990 to 2024

# Searches:

1:Hydroxymethylglutaryl-CoA Reductase Inhibitors (主题) OR Hydroxymethylglutaryl CoA Reductase Inhibitors (主题) OR HMG-CoA Reductase Inhibitor (主题) OR HMG CoA Reductase Inhibitor (主题) OR Statin (主题) OR HMG-CoA Statins (主题) OR HMG-CoA Reductase Inhibitors (主题) OR Hydroxymethylglutaryl-Coenzyme A Inhibitors (主题) OR Hydroxymethylglutaryl-CoA Inhibitors (主题) OR Hydroxymethylglutaryl-CoA Reductase Inhibitor (主题) OR Hydroxymethylglutaryl CoA Reductase Inhibitor (主题) OR hydroxymethylglutaryl coenzyme A reductase inhibitor (主题) OR HMG CoA reductase inhibitor (主题) OR hmg coenzyme a reductase inhibitor (主题) OR statin (drug) (主题) Date Run: Thu May 09 2024 17:11:45 GMT+0800 (中国标准时间) Results: 44,113

2: Cholesterol Ester Transfer Proteins (主题) OR Cholesterol Ester Transport Protein, CETP (主题) OR Cholesteryl Ester Transfer Protein (主题) OR Cholesterol Ester Transfer Protein (主题) OR Cholesteryl Ester Exchange Protein (主题) OR CETP (主题) Date Run: Thu May 09 2024 17:12:37 GMT+0800 (中国标准时间) Results: 6,732

3: #1 AND #2 Date Run: Thu May 09 2024 17:13:09 GMT+0800 (中国标准时间) Results: 522

**Cochrane LIbrary**

Search Name:

Date Run: 09/05/2024 10:57:48

Comment:

ID Search Hits

#1 MeSH descriptor: [Hydroxymethylglutaryl-CoA Reductase Inhibitors] explode all trees 5037

#2 (Hydroxymethylglutaryl-CoA Reductase Inhibitors):ti,ab,kw OR (Hydroxymethylglutaryl CoA Reductase Inhibitors):ti,ab,kw OR (HMG-CoA Reductase Inhibitor):ti,ab,kw OR (HMG CoA Reductase Inhibitor):ti,ab,kw OR (Statin):ti,ab,kw 8021

#3 (HMG-CoA Statins):ti,ab,kw OR (HMG-CoA Reductase Inhibitors):ti,ab,kw OR (Hydroxymethylglutaryl-Coenzyme A Inhibitors):ti,ab,kw OR (Hydroxymethylglutaryl-CoA Inhibitors):ti,ab,kw OR (Hydroxymethylglutaryl-CoA Reductase Inhibitor):ti,ab,kw 615

#4 (Hydroxymethylglutaryl CoA Reductase Inhibitor):ti,ab,kw OR (hydroxymethylglutaryl coenzyme A reductase inhibitor):ti,ab,kw OR (HMG CoA reductase inhibitor):ti,ab,kw OR (hmg coenzyme a reductase inhibitor):ti,ab,kw OR (statin (drug)):ti,ab,kw 6562

#5 #1 or #2 or #3 or #4 10985

#6 MeSH descriptor: [Cholesterol Ester Transfer Proteins] explode all trees 228

#7 (Cholesterol Ester Transfer Proteins):ti,ab,kw OR (Cholesterol Ester Transport Protein, CETP):ti,ab,kw OR (Cholesteryl Ester Transfer Protein):ti,ab,kw OR (Cholesterol Ester Transfer Protein):ti,ab,kw OR (Cholesteryl Ester Exchange Protein):ti,ab,kw 421

#8 (CETP):ti,ab,kw 324

#9 #6 or #7 or #8 489

#10 #5 and #9 119
