# Supplementary material for: Lipid-lowering effect of combined therapy with high-intensity statins and CETP inhibitors: a Systematic Review and meta-analysis
Source: Front Endocrinol (Lausanne). 2025 May 1;16:1512670. doi: 10.3389/fendo.2025.1512670 (PMC12078159; doi:10.3389/fendo.2025.1512670)
Supplement: Supplementary file 3 [file Table2.docx]

Supplementary Table S2. Basic characteristics of the included studies

| Study | Year | Region | Study type | Intervention | | Sample size | | Gender(M/F) | | Age | | Follow-up | Dosage/per day | Outcomes | Results |
| --- | --- | --- | --- | --- | --- | --- | --- | --- | --- | --- | --- | --- | --- | --- | --- |
|  |  |  |  | Experimental | Control | Experimental group | Control group | Experimental group | Control group | Experimental group | Control group |  |  |  |  |
| Barter | 2012 | Australia | RCT | torcetrapib plus atorvastatin | placebo plus atorvastatin | 1508 | 1559 | 1174/334 | 1212/347 | 60.6 ± 7.6 | 60.6 ± 7.6 | 3 months | torcetrapib 60mg atorvastatin 40mg | 1.HDL-C 2.LDL-C 3.ApoAI 4.ApoB | Torcetrapib increased all-cause mortality (ACM) and major cardiovascular events (MCVEs). Post-hoc analysis showed this harm was limited to the 10 mg atorvastatin subgroup, with no harm observed in higher atorvastatin doses(17). |
| Barter | 2012 | Australia | RCT | torcetrapib plus atorvastatin | placebo plus atorvastatin | 771 | 674 | 612/159 | 534/140 | 59.8 ± 7.6 | 60.4 ± 7.5 | 3 months | torcetrapib 60mg atorvastatin 80mg |  |  |
| Derks(1) | 2010 | Switzerland | Crossover study | dalcetrapib plus atorvastatin | atorvastatin | 26 | 26 | 26/0 | 26/0 | 33.9 ± 12.2 | 33.9 ± 12.2 | 7-14 days | dalcetrapib 900mg atorvastatin 40mg | 1.HDL-C 2.LDL-C 3.TC 4.TG 5.AEs | The combination of dalcetrapib and atorvastatin significantly increased HDL-C levels, with marked reductions in LDL-C, VLDL-C, and total cholesterol, while triglyceride levels showed comparable reductions to atorvastatin alone. The therapy was well tolerated, with headache being the most common but mild adverse event and no serious safety concerns, demonstrating both efficacy in lipid improvement and safety in concurrent use(18). |
| Derks(2) | 2010 | Switzerland | Crossover study | dalcetrapib plus rosuvastatin | dalcetrapib | 31 | 31 | 31/0 | 31/0 | 30.0 ± 1.93 | 30.0 ± 1.93 | 8 days | dalcetrapib 900mg rosuvastatin 40mg | 1.HDL-C 2.LDL-C 3.ApoAI 4.ApoB 5.TC 6.TG 7.AEs | Dalcetrapib, when administered alone or in combination with statins (pravastatin, rosuvastatin, or simvastatin), significantly increased HDL-C levels, with no compromise in efficacy when coadministered with statins. Greater reductions in LDL-C were observed with the combination of dalcetrapib and statins compared to statins alone. Dalcetrapib alone and in combination with simvastatin increased the concentration of large HDL particles while decreasing medium and small HDL particles. Triglyceride levels were not notably altered by dalcetrapib alone or in combination with statins(19). |
| Furtado | 2022 | USA | RCT | evacetrapib plus atorvastatin | placebo plus atorvastatin | 81 | 36 | 55/26 | 26/10 | 63±8 | 63±8 | 3 months | evacetrapib 130mg atorvastatin 40mg | 1.ApoAI | CETP inhibitors (evacetrapib and torcetrapib) significantly increased total HDL cholesterol and apoA1 but failed to reduce cardiovascular disease risk, likely because they increased dysfunctional HDL subspecies, particularly HDL containing apoC3, which is associated with higher coronary heart disease risk(20). |
| Kastelein | 2007 | Netherlands | RCT | torcetrapib plus atorvastatin | placebo plus atorvastatin | 450 | 454 | 214/236 | 232/222 | 46.8±12.0 | 45.2±12.9 | 24 months | torcetrapib 60mg atorvastatin 56.5mg | 1.HDL-C 2.LDL-C 3.TC 4.TG 5.AEs | Despite significantly increasing HDL cholesterol levels by 52% and reducing LDL cholesterol by 21%, torcetrapib failed to slow the progression of atherosclerosis compared to atorvastatin alone. In fact, it was associated with progression of disease in the common carotid segment. These findings suggest that raising HDL cholesterol with CETP inhibition, as with torcetrapib, may not confer cardiovascular benefits(21). |
| Nicholls | 2017 | Australia | RCT | evacetrapib plus atorvastatin | atorvastatin | 86 | 40 | NR | NR | NR | NR | 90 days | evacetrapib 130mg atorvastatin 40mg | 1.HDL-C 2.LDL-C 3.TG 4.ApoAI 5.ApoB 6.AEs | The ACCENTUATE trial compared the effects of adding the CETP inhibitor evacetrapib, increasing atorvastatin dose, or adding ezetimibe in patients with atherosclerotic cardiovascular disease (ASCVD) and/or diabetes already on atorvastatin 40 mg. Evacetrapib significantly reduced LDL-C (-33%) more than ezetimibe (-27%) or increasing statin dose (-6%), and increased HDL-C (+125%), apoA-I (+46%), and cholesterol efflux capacity (+35%). Despite these lipid improvements, evacetrapib failed to reduce cardiovascular events in the ACCELERATE trial, possibly due to adverse effects on novel atherogenic risk factors. This highlights the complexity of CETP inhibition and its unclear cardiovascular benefits despite favorable lipid changes(23). |
| Nicholls | 2022 | Australia | RCT | obicetrapib plus atorvastatin/ rosuvastatin | atorvastatin or rosuvastatin | 40 | 40 | 23/17 | 19/21 | 61.1 ± 8.13 | 61.3 ± 8.77 | 8 weeks | obicetrapib 5mg atorvastatin(40)80mg/ rosuvastatin20(40) | 1.HDL-C 2.LDL-C 3.TG 4.ApoAI 5.ApoB 6.AEs | Obicetrapib significantly reduced LDL-C by 42.9% (5 mg) and 45.7% (10 mg) compared to placebo (P < 0.0001). These reductions were consistent across different LDL-C measurement methods, including preparative ultracentrifugation (PUC) and the Martin–Hopkins equation, addressing concerns about the accuracy of LDL-C measurements in the context of CETP inhibition. In addition to LDL-C, obicetrapib also significantly increased HDL-C by 135% (5 mg) and 165% (10 mg) and reduced non-HDL-C by 38.9% (5 mg) and 44.4% (10 mg). Furthermore, lipoprotein(a) [Lp(a)], an independent cardiovascular risk factor, was reduced by 33.8% (5 mg) and 56.5% (10 mg)(22). |
| Nicholls | 2022 | Australia | RCT | obicetrapib plus atorvastatin/ rosuvastatin | atorvastatin or rosuvastatin | 40 | 40 | 25/15 | 19/21 | 62.9 ± 8.48 | 61.3 ± 8.77 | 8 weeks | obicetrapib 10mg atorvastatin(40)80mg/ rosuvastatin20(40) |  |  |

RCT, Randomized controlled trial; NR, Not reported; HDL-C, High-density lipoprotein cholesterol; LDL-C, Low-density lipoprotein cholesterol; TC, Total cholesterol; TG, Triglyceride; ApoAI, Apolipoprotein AI; ApoB, Apolipoprotein B; AEs, Adverse events.
